# Balancing Data on Deep Learning-Based Proteochemometric Activity Classification

**DOI:** 10.1021/acs.jcim.1c00086

**Published:** 2021-03-29

**Authors:** Angela Lopez-del Rio, Sergio Picart-Armada, Alexandre Perera-Lluna

**Affiliations:** † B2SLab, Departament d’Enginyeria de Sistemes, Automàtica i Informàtica Industrial, Universitat Politècnica de Catalunya, 08028 Barcelona, Spain; ‡ Department of Biomedical Engineering, Institut de Recerca Pediàtrica Hospital Sant Joan de Déu, 08950 Esplugues de Llobregat, Spain

## Abstract

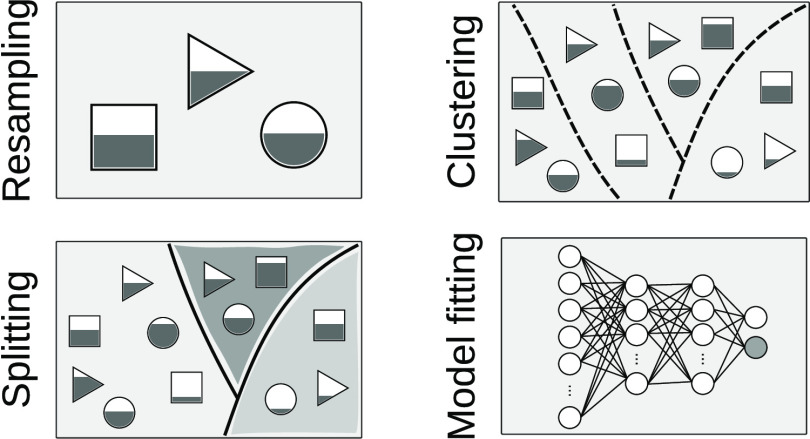

In silico analysis of biological activity data has become an essential technique in pharmaceutical development. Specifically, the so-called proteochemometric models aim to share information between targets in machine learning ligand–target activity prediction models. However, bioactivity data sets used in proteochemometric modeling are usually imbalanced, which could potentially affect the performance of the models. In this work, we explored the effect of different balancing strategies in deep learning proteochemometric target–compound activity classification models while controlling for the compound series bias through clustering. These strategies were (1) no_resampling, (2) resampling_after_clustering, (3) resampling_before_clustering, and (4) semi_resampling. These schemas were evaluated in kinases, GPCRs, nuclear receptors, and proteases from BindingDB. We observed that the predicted proportion of positives was driven by the actual data balance in the test set. Additionally, it was confirmed that data balance had an impact on the performance estimates of the proteochemometric model. We recommend a combination of data augmentation and clustering in the training set (semi_resampling) to mitigate the data imbalance effect in a realistic scenario. The code of this analysis is publicly available at https://github.com/b2slab/imbalance_pcm_benchmark.

## Introduction

The discovery, design, and bring-to-market of a novel small-molecule drug is a very challenging process and very expensive in terms of money, time, and effort.^1^ Computer-assisted drug design (CADD) methods can help improve and refine the identification of hits in the first steps of drug development, thus having a huge positive impact on the costs of the whole process.^2^ Traditionally, interactions between ligands and targets have been predicted in CADD through a quantitative structure–activity relationship (QSAR) approach.^3^ In QSAR, a target is fixed and only information from compounds is used for modeling and predicting binding for the said target. However, the compartmentalized nature of QSAR does not allow for discovering new cross-interactions between the ligand and targets for which no training data is available.^2^ Proteochemometric modeling (PCM) is an extension of QSAR, which overcomes this drawback by combining information of both ligand and protein descriptors on a supervised prediction model. PCM allows for the integration of different sources of data in one model and for the general prediction of which ligands will bind to which targets.^4^

Both PCM and QSAR usually apply machine learning (ML) techniques such as random forests, support vector machine, logistic regression, or partial least squares.^2,4^ Following the trends in other fields and the growing availability of data, deep learning (DL) has also been increasingly and successfully applied for bioactivity prediction,^5^ specially for QSAR modeling.^6^ The application of DL to PCM followed, taking advantage of public databases^7−9^ and improving the descriptor representation.^10,11^

However, an important issue for PCM and QSAR DL models is the amount and quality of data when compared to other fields of application, since increasing the number of data samples in drug discovery is expensive and, thus, often infeasible.^12^ This poses a problem since neural networks require a large quantity of training data to actually learn. While in other fields this problem is alleviated through data augmentation, i.e., an artificial increase of the number of observations of the training set to help the model generalize, this regularization technique is not yet commonly used in CADD. Some studies have considered different variants of the SMILES of each molecule as a way of data augmentation,^13,14^ but despite its proven benefits, its use is not widespread yet. This is partly due to the lack of consensus in the input representations, where alternatives to SMILES are often used.

Another factor highly affecting QSAR and PCM models is data imbalance, since the class definitions based on bioactivity data can result in highly skewed labels. In this regard, Zakharov et al.^15^ explored how data balancing affected self-consistent regression QSAR models using highly imbalanced PubChem bioassays. The study proposed a method including cost-sensitive learning and undersampling approaches to obtain more accurate predictions. Using the same data, Korkmaz explored how data balancing affected DL-based QSAR models.^16^ The study concluded that imbalance has indeed a negative impact on the performance of the models but that this impact could be alleviated by applying oversampling methods like SMOTE (synthetic minority oversampling technique)^17^ on the fingerprint representations of the molecules. Besides, oversampling methods could also serve the purpose of augmenting the original data set.

While the effect of data imbalance on model performance has been studied for shallow ML and DL QSAR, to our knowledge, there are no analogous studies yet for PCM. In PCM, modeling information between targets is shared, which may compensate those for which activity data is very imbalanced. However, it is still to be proved if this compensation does happen or if the results are actually dominated by the original imbalance of each target.

Recently, it has been shown that for the validation of PCM models, it is important to control the chemical series bias through clustering techniques to get more reliable performance estimates.^8,18^ This adds a complexity layer to the imbalance handling since clustering can affect the data balance in PCM. Since Korkmaz and Zakharov et al. did not consider the potential similarity between different compounds when validating their results,^15,16^ its impact on data balancing is yet to be tested.

In this paper, we study the effect of different balancing strategies in DL-based PCM target–compound activity classification models. While handling data imbalance, we also study how to integrate the compounds’ clustering in this process. We describe the behavior of model predictions and performance according to imbalance handling.

## Materials and Methods

### Data

We evaluated the different balancing models on the benchmark data set used in DeepAffinity.^19^ The original data set contains binding data from BindingDB,^20^ merged with the amino acid sequence information from UniRef^21^ and the SMILES representation of compounds from STITCH.^22^ The original data set consisted of IC50, *K*_i_, or *K*_d_ values from 829 033 compound–protein pairs. We classified the data-set proteins into the main protein families according to the release 2018_09 from Uniprot^23^ and focused our study on proteins of the kinase family. Our results were further validated on the G protein-coupled receptor (GPCR), nuclear receptor (NR), and protease (PR) families (separately). Binding activities were in the logarithm form, so a threshold of 6 was applied to have binary labels for classification (active/inactive). Table 1 summarizes the final data set we used in our analysis. The same descriptive table, but for GPCR, NR, and PR families, can be seen in Table S1 of the Supporting Information.

**Table 1:** Summary of the Kinase Sub-Data-Set

entity	number
compounds	84 643
targets	490
ligand–target pairs	129 997
actives	99 158
inactives	30 839

In Figures S1 and S2 of the Supporting Information, the proportion of actives/inactives for each protein of each of the studied protein families is represented in more detail.

### Descriptors

We represented compounds by their molecular fingerprints, in which structural information is represented by bits in a bit string. We used the fingerprints from PubChem^24^ provided in DeepAffinity.^19^ In these, basic substructures of compounds are encoded in a 1D binary vector with a length of 881 bits.

We represented proteins by raw amino acid sequences transformed to one-hot encoding. Each amino acid was represented by a binary vector of length 26. Protein sequences were then normalized to the maximum length of 1499. Those sequences shorter than 1499 were zero-padded. According to the recommendation of our previous work,^25^ we tuned the padding type and obtained the best results with prepadding (adding zeros to the beginning of the sequence).

### Validation Strategy

A splitting strategy based on compound clustering (of both actives and inactives) was applied to the bioactivity data, omitting target information. Clustering-based validation strategies have been used to avoid the compound series bias, making sure that there are no similar molecules in training, validation, and test sets.^18,26,27^ We followed the implementation of our previous study on cross-validation strategies in PCM,^8^ where *K*-means clustering with *k* = 100 was applied to the fingerprint description of the compounds. Data was divided into training, validation (for selecting the best epoch), and test (for evaluating the performance) sets with a proportion of 80/10/10%. This splitting was randomly performed 10 times (folds) to test the consistency of the results, thus training and testing each model in 10 different data partitions. As further explained in the next subsection, for some balancing strategies the clustering was applied before the resampling and for others it was applied afterward.

### Balancing Strategies

We chose an oversampling method to balance data since oversampling was shown to improve performance in the Korkmaz study of data imbalance in DL-based QSAR^16^ and in a systematic study of data imbalance with CNNs.^28^ Oversampling methods increase the number of samples in the minority class to create a balanced data set. Specifically, we used the SMOTE oversampling technique,^17^ which creates synthetic data points of the minority class similar to those available. Resampling with SMOTE was done on a per-protein basis so that each protein would be balanced. Some proteins had to be discarded in certain strategies since either there were only active or inactive ligands or the number of samples in the minority class was smaller than the number of neighbors used for constructing the synthetic samples (*k* = 5) and SMOTE was not applicable.

**Figure 1:**
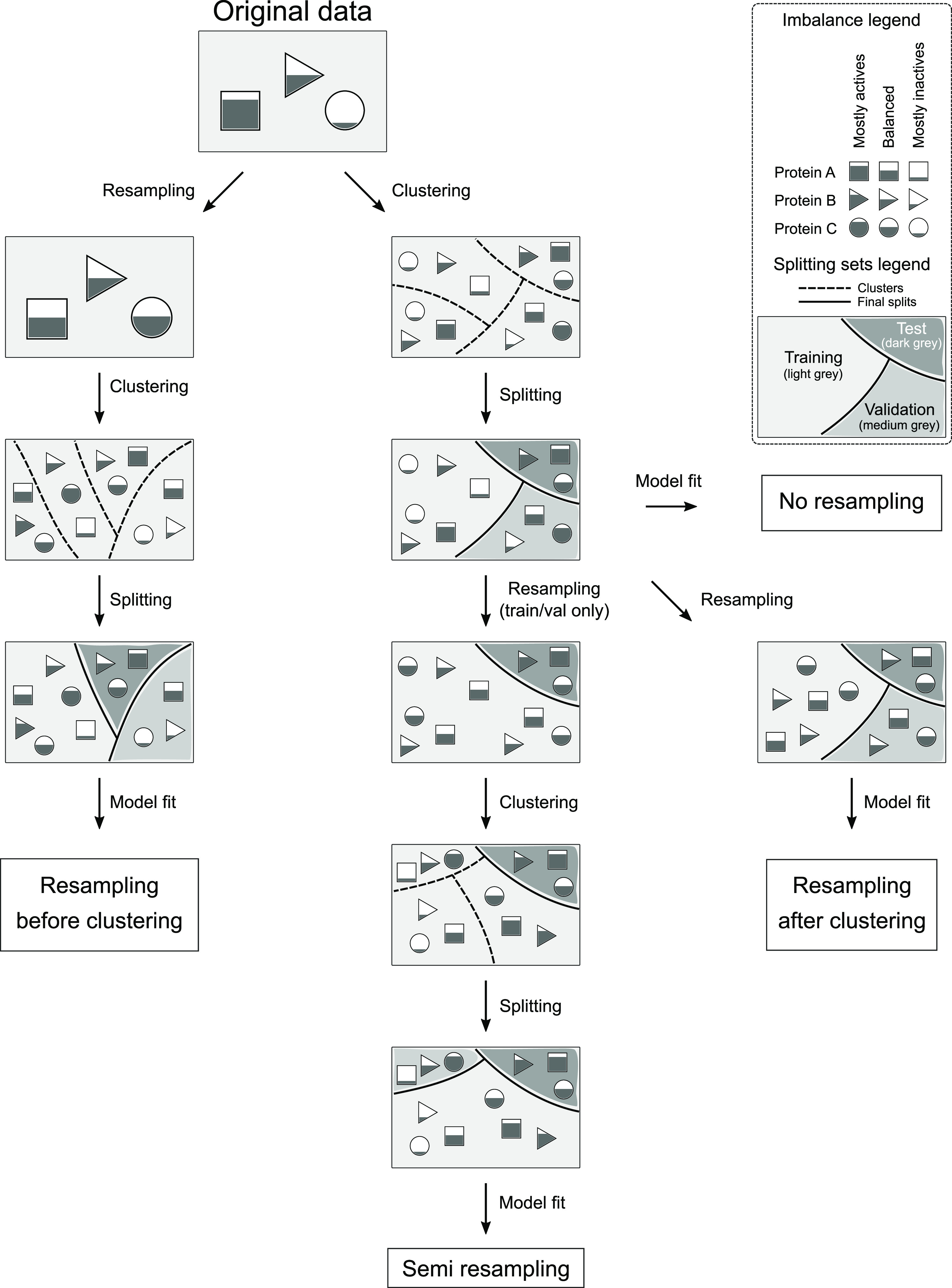
Description of the four balancing strategies that were applied to the bioactivity data. **Resampling**_**before**_**clustering**, where resampling per protein is applied prior to clustering and splitting; **resampling**_**after**_**clustering**, where data is first clustered and split and then each protein activity data in each set is resampled; **semi**_**resampling**, in which the splitting is performed and then the test set is kept without resampling but the training + validation set is resampled and clustered; and **no**_**resampling**, in which the imbalance of the original data is kept and clustering is applied prior to splitting. Dashed lines indicate clusters, and solid lines delineate the final splits; in the latter, training, validation, and test sets can be recognized by their shade intensity. Filled shapes illustrate the active ratios for each of the three example proteins, in every set or cluster.

Unlike Korkmaz, who applied data balancing methods to each training set,^16^ we tested four different combinations of balancing, data clustering, and splitting (see Figure 1): **no**_**resampling**, in which bioactivity data for each protein was taken as it was, and clustering was applied to perform the splitting; **resampling**_**after**_**clustering**, in which after clustering data and splitting it into training, validation, and test, each protein activity data in each set was resampled and attained a 50% active/inactive proportion; **resampling**_**before**_**clustering**, in which, contrary to the previous strategy, resampling was applied prior to clustering and splitting, so while the global protein-wise proportion of actives/inactives was 50%, it did not have to be 50% within each splitting set; and **semi**_**resampling**, in which the splitting performed in the *no*_*resampling* strategy was reused, the test set was kept without resampling but the training + validation set was resampled, re-clustered, and re-split into training and validation.

The overall number of resampling rounds, by strategy, was 0 in no_resampling, 30 in resampling_after_clustering (in each fold, one resampling in the training set, one in the validation set, and one in the test set), 1 in resampling_before_clustering (from which the 10 folds were calculated), and 10 in semi_resampling (in each fold, one resampling in the training and validation sets combined). In turn, each resampling round consisted of oversampling each one of the available proteins in the corresponding set. The total number of active and inactive protein–compound pairs in each strategy, splitting set, and protein family can be seen in Table S2 of the Supporting Information.

### Prediction Models

We built a DL model for studying the impact of different data balancing strategies in state-of-the-art PCM. Besides, a random prediction was generated to have an absolute, input-naïve baseline. Having predictions from a random baseline served two purposes: characterizing how well random predictions scored in each performance metric in a scenario with varying data imbalance and putting the performance of DL models in context.

#### Random Baseline

A random baseline was computed according to the active/inactive ratio of the training set for each strategy and each fold. Let *f*  be the fraction of actives in the training samples involving a protein and *n* be the number of samples to be predicted in the test set for that protein. The random baseline is obtained by first sampling ⌊*fn* + 0.5⌋ values from a uniform distribution in [0.5, 1] (actives) and *n* – ⌊*fn* + 0.5⌋ values from a uniform distribution in [0, 0.5] (inactives) and then concatenating both and shuffling. This procedure keeps the active/inactive balance by design while producing random activity predictions.

#### Deep Learning Model

We studied the impact of data balancing strategies on a DL model. We followed the Korkmaz strategy of selecting a simple, well-established architecture whose complexity issues would not be a confounder of the factor under study.^16^ We refrained from using long short-term memory networks since they have convergence issues when training sequences longer than 1000 elements.^29^ Model hyperparameters were tuned using the validation set, choosing the simplest working architecture. As in our previous work,^8^ the DL PCM model consisted of two analysis blocks. The amino acid sequence analysis block was a 1D convolutional neural network. The fingerprint analysis block consisted of a feed-forward neural network. Dropout was used in both branches to prevent overfitting.^30^ The representations built by the compound and target analysis blocks were then merged, and the information was passed through a softmax activation unit, which quantified the ligand–target pair activity probability. A schematic representation of the DL-based PCM model can be found in Figure S3 of the Supporting Information, along with further details on the optimized hyperparameters.

In the training process, the weights of the selected model were those from the epoch with the maximum accuracy (proportion of correct predictions) on the validation set. This process was run for each strategy and fold. Then, each selected model was used to predict on their corresponding test set.

### Characterization of Data Balance

The data balancing strategy had an impact on the actual data balance, defined as the proportion of active molecules for a protein.
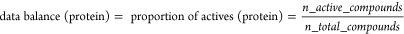
Thus, a comprehensive analysis of data balance was carried out to better understand and interpret performance results. For each of the balancing strategies, the original distribution of active ratios per protein was characterized. We also compared the original imbalance of the training and test sets for each strategy to explore possible trends and studied the effect that other covariates (the protein length and the number of interactions of each protein in its corresponding set and fold) might have on the original test set imbalance.

The next key question was to narrow down the factor driving the proportion of actives in the predicted data (as opposed to the original data). The main options under consideration were as follows: (1) a constant, global imbalance that the model would learn from the whole data set; (2) the protein-wise imbalance that the model would learn in the training set; and (3) a test-set-driven imbalance, based on its actual imbalance. To answer this, the test set predictions were binarized with a probability threshold of 0.5 and the proportion of predicted actives was computed by protein and also compared to the ratios of the original test and training sets.

### Performance Metrics

The resampling strategies were assessed with various performance metrics for binary classifiers and prioritizers. The selection was based on those used by Korkmaz:^16^ balanced accuracy, *F*_1_-score, the Matthews correlation coefficient (MCC), and area under the ROC curve (AUROC). All of them are insensitive to class imbalance. In the case of the *F*_1_-score, we used the macro-average, which is computed by averaging the *F*_1_-score for the active and inactive labels. Further details on the definition of these metrics can be found in the Supporting Information.

The performance metrics were computed on the predictions of each selected model in its corresponding test set. For each combination of resampling strategy, fold and protein, we computed the performance of (1) the random baseline and (2) the DL model. AUROC was computed from raw predicted probabilities, while the *F*_1_-score, balanced accuracy, and MCC were derived from the binarized predictions. We tested the significance of the differences between strategies by means of the nonparametric two-sided Wilcoxon test for paired samples.^31^

### Explanatory Models

Performance metrics and predicted ratios were further described through linear models built upon the different combinations of variables considered in this analysis. Our prior work in similar scopes had found them insightful since they allow for a statistical analysis of the contribution of each factor under study.^8,25,32^ Each of the data points used for fitting an explanatory linear model corresponded to a different protein. Simpler claims were investigated with Pearson’s *r* for linear correlation, using confidence intervals (CIs) and *p*-values for significance.

On the one hand, the predicted ratio of actives (*r*_pred_) was modeled through the quasibinomial logistic model^33^ in eq 1, stratified by strategy, to quantify the effect of different variables of interest.
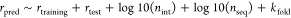
1

Specifically, the main variables of interest in this model were the actual ratios in the training (*r*_training_) and test (*r*_test_) sets, both numeric between 0 and 1. As additional covariates, the number of interactions (*n*_int_) and the sequence length (*n*_seq_) (both numerical) and the fold number (*k*_fold_, categorical) were also included. This model was not computed for the resampling_after_clustering strategy, since the data balance (and thus, the predicted active ratio) is enforced.

On the other hand, each performance metric was explained through the linear model described by eq 2.
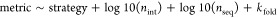
2

The response was the quantitative metric of interest in each case (one model per metric), while strategy was categorical (no_resampling, resampling_after_clustering, resampling_before_clustering, semi_resampling). The same covariates as in eq 1 were added.

However, before evaluating the DL model, the performance metrics of the baseline were characterized: the strategy variable was tested with a type 3 analysis of variance (ANOVA)^34^ to pinpoint the imbalance-sensitive and -insensitive metrics. Metrics were called imbalance-sensitive if the imbalance-aware random baseline exhibited different performances between resampling strategies.

The imbalance-insensitive metric models were fitted analogously to the baseline performance models (with eq 2). However, to address the pitfalls of the direct comparison of metrics whose baselines might differ, imbalance-sensitive performance metrics were defined and modeled as follows
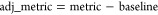
3

Thus, adjusted performance metrics were also described with eq 2 but changing the response to *adj*_*metric* of eq 3
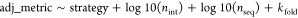
4

Note that while all of the metrics but MCC were non-negative, the adjusted metrics could show negative values when the performance of the DL model was lower than that of the baseline.

Reference categories for categorical variables were no_resampling for strategy and 0 for fold. Each term of the fitted model represents the difference between its specified category and the reference category of that variable.

### Implementation

We trained every DL model with an Adam optimizer^35^ (learning rate = 5 × 10^–4^, β_1_ = 0.1, β_2_ = 0.001, ϵ = 1 × 10^–8^; decay rate is defined as the learning rate/number of epochs) for 100 epochs, with a batch size of 128 for both training and validation. Both DL models and random baselines were implemented in Python 3.6.9. Specifically, DL models were implemented with the package Keras^36^ 2.3.1 using Tensorflow^37^ 2.1.0 as the backend and run on two NVIDIA GeForce GTX 1070 GPUs. SMOTE data balancing was applied using the imbalanced-learn Python package.^38^ The statistical processing of results was performed in R software (3.6.3).^39^

## Results

Unless stated otherwise, the results shown in this section refer to the kinase protein family.

### Characterization of the Original Data Balance

#### Distribution of the Active Ratio

**Figure 2:**
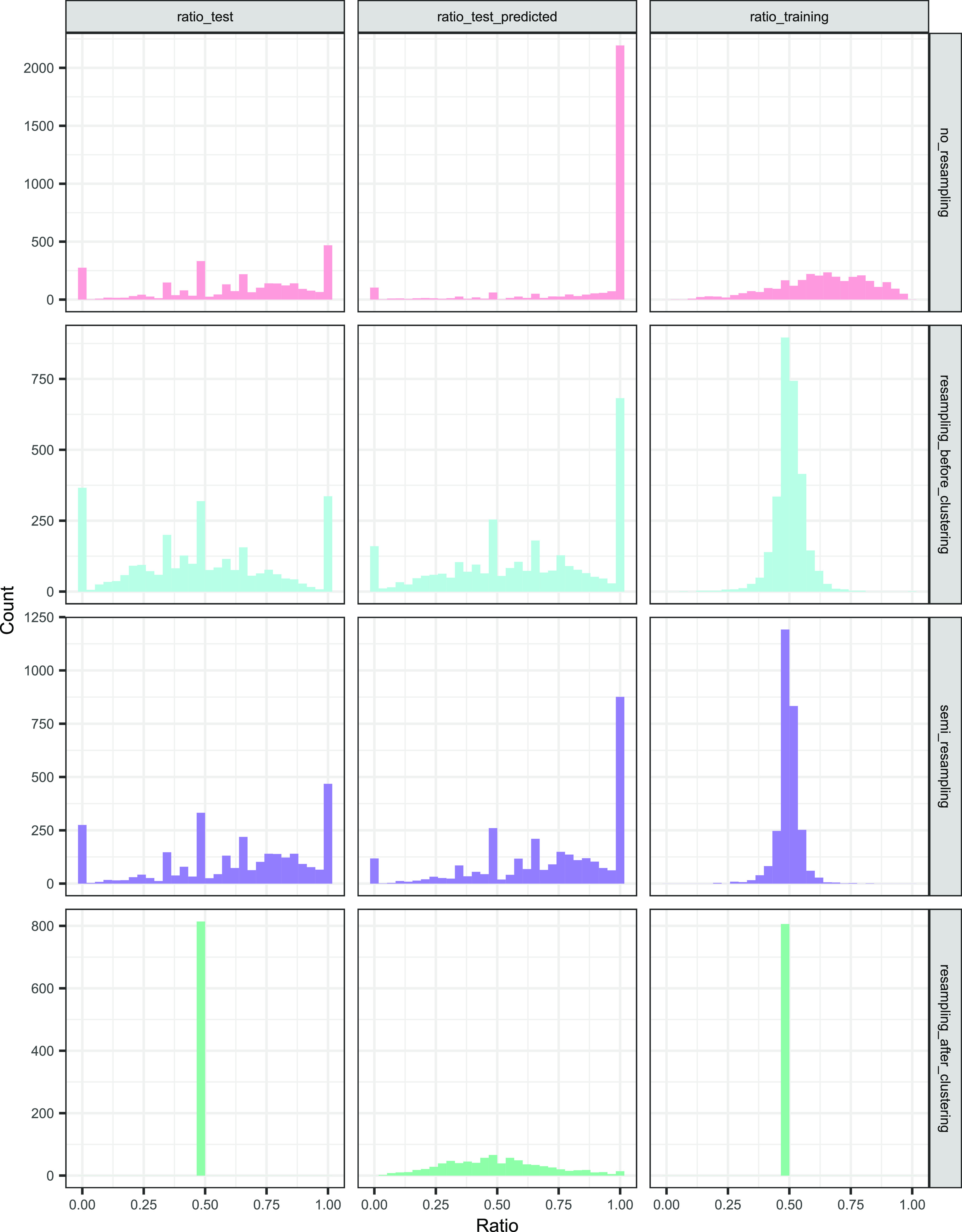
Histograms of the active ratios in the training set and in the test set (both original and predicted by the deep learning model), within each resampling strategy. Each histogram combines all of the folds.

Figure 2 displays the original distribution of the active ratio in the training and test sets. Test sets tended to magnify data imbalance, creating around 24% of the times extreme cases, i.e., all actives or all inactives, not present in the training set. Strategy-wise, no_resampling kept similar data distributions in training and test, resampling_before_clustering and semi_resampling led to a more balanced training set but an imbalanced test set, and resampling_after_clustering only kept totally balanced proteins in both training and test sets.

#### Training and Test Imbalance Comparison

**Figure 3:**
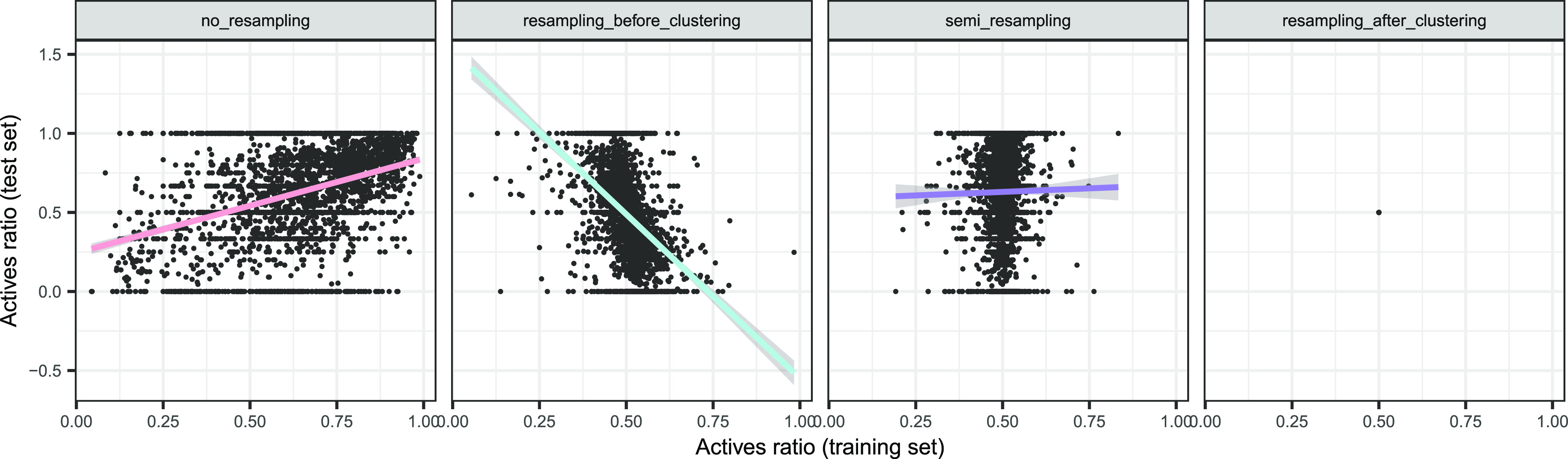
Comparison of the training and test original active ratios, by resampling strategy. Linear fit trends were added by strategy, and the shadowed areas indicated the 95% CI of the expected value. Each plot combines all of the folds.

Figure 3 reveals positive, negative, and null trends between the training and test protein balances, and Table S4 of the Supporting Information quantifies these correlations. No_resampling showed a positive correlation between both (Pearson’s *r* 95% CI: [0.338, 0.400], *p* < 10^–16^), i.e., proteins were prone to keeping their (im)balance in training and test sets. Resampling_before_clustering showed an inverse relationship (Pearson’s *r* 95% CI: [−0.457, −0.398], *p* < 10^–16^), which was expected since this strategy started from globally balanced proteins, and after the clustering, an imbalance in one direction in the training set entailed an inverse imbalance in the test set. Semi_resampling led to uncorrelated train and test balances (Pearson’s *r* 95% CI: [−0.024, 0.051], *p* = 0.48), expected since the training set was resampled, breaking any correlation with the test set balance. Resampling_after_clustering always kept balanced proteins, by design.

#### Other Covariates

The effect that the number of interactions for each protein in its corresponding set and fold, and the protein length (i.e., number of amino acids), had on the test set imbalance was investigated (Figures S5 and S6 and Tables S5 and S6 of the Supporting Information). Proteins with the greatest imbalance tended to be among those with the least interactions (Table S5: Pearson’s *r* 95% CI [−0.097, −0.026], *p* = 8.01 × 10^–4^ for no_resampling and semi_resampling; [−0.307, −0.240], *p* < 10^–16^ for resampling_before_clustering). The sequence length had no consistent effect on protein imbalance (Table S6: Pearson’s *r* 95% CI [−0.052, 0.020], *p* = 0.37 for no_resampling and semi_resampling; [−0.082, −0.009], *p* = 0.014 for resampling_before_clustering).

### Analysis of the Predicted Proportions of Active Compounds

Figure 2 represents the ratio of predicted actives by protein, and Table S7 of the Supporting Information summarizes the percentage of proteins with all actives or inactives (extreme cases). They show that the no_resampling strategy was inclined to predict everything as positives (71.6% of the time, compared to 3.5% for predicting all negatives). Resampling_before_clustering and semi_resampling alleviated the imbalance in the predictions but still retained a spike of proteins where all of the compounds were predicted as positives (23.4 and 29.1%) and negatives (5.5 and 4%). Resampling_after_clustering kept a wide and symmetric distribution of predicted actives, with only 1.2% predicted as all actives and 0% as all inactives.

Figure 2 also puts the ratio of predicted actives in context with the original training and test ratios: the distribution was more similar to that of the test proportions than to that of the training ones (except for resampling_after_clustering, since those proportions were constant).

**Figure 4:**
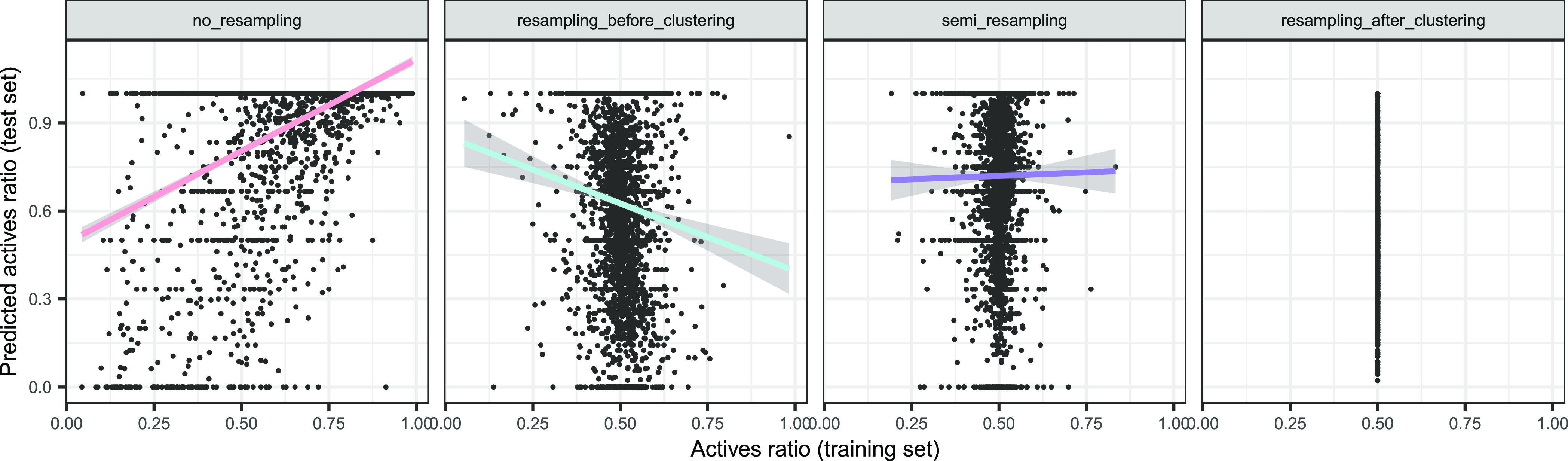
Predicted ratios, as a function of training ratios, by resampling strategy. Linear fit trends were added by strategy, and the shadowed areas indicated the 95% CI of the expected value. Each plot combines all of the folds.

Figure 4 puts the predicted ratios in the context of the training ratios, and Table S8 of the Supporting Information quantifies their correlations, elucidating a variety of trends: (1) no_resampling shows a positive trend between the training and the predicted ratio (Pearson’s *r* 95% CI: [0.440, 0.496], *p* < 10^–16^), but since the training and the test ratio are also positively correlated (Figure 3), the latter could be the one driving the predicted ratio of positives; (2) resampling_after_clustering had a constant training ratio, meaning that the predicted ratio was not explainable by differences in training ratios; (3) resampling_before_clustering showed instead a negative relation between the training and the predicted ratio (Pearson’s *r* 95% CI: [−0.130, −0.058], *p* = 3.77 × 10^–7^), but since the former and the test ratio also anticorrelated (Figure 3), the simplest explanation was that the test ratio drove the predicted test ratio; and (4) semi_resampling showed no apparent correlation between the predicted ratio and the training ratio (Pearson’s *r* 95% CI: [−0.029, 0.045], *p* = 0.68).

The models in eq 1 that describe the predicted ratio of actives for each balancing strategy are summarized in Tables S9 and S10 of the Supporting Information. For semi_resampling and resampling_before_clustering (Table S9), the original active ratio in the test set had a positive, significant effect on the predicted active ratio (β = 0.945 and 0.784, both *p* < 10^–16^). However, the original active ratio of the training set showed no evidence of affecting the predicted ratio (β = 0.197 and −0.446, *p* = 0.73 and 0.31). Conversely, for the no_resampling strategy (Table S10), both the original training (β = 8.312, *p* < 10^–16^) and test ratios (β = 1.102, *p* = 2.6 × 10^–9^) had positive, significant effects on the predicted active ratio. In the three models, the number of interactions per protein had a significant, negative effect (β = −0.391, −0.396, and −1.24, all *p* < 10^–16^), and some of the folds entailed significant variations of the predicted ratio.

### Performance Metrics

#### Baseline Performance

**Figure 5:**
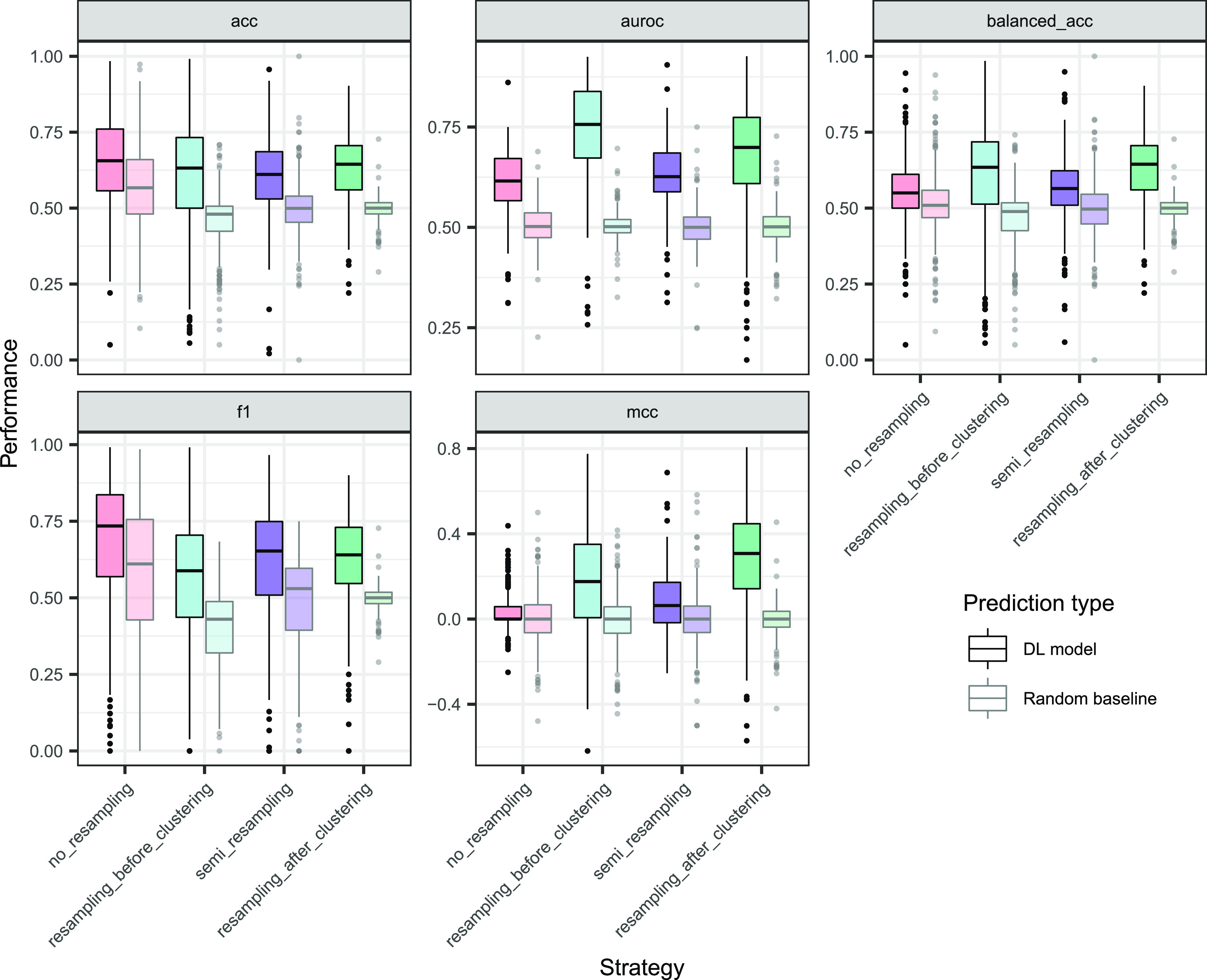
Absolute performance metrics for balancing strategies and their corresponding imbalance-aware random baselines. Data points correspond to proteins, averaged over folds.

Figure 5 shows a fold-averaged picture of the metrics by protein and by model type (DL or input-naïve baseline). Visual inspection suggested that the *F*_1_-score, accuracy, and possibly balanced accuracy were affected by the baseline data imbalance. To quantify this finding, the model in eq 2 was fitted to the baseline performance metrics. According to Table S12 of the Supporting Information, the strategy term was significant (type 3 ANOVA, *p* < 10^–16^, *p* < 10^–16^ and 5.61 × 10^–11^) for those three metrics and nonsignificant in AUROC and MCC (*p* = 0.91 and 0.82). Based on this, metrics were divided into two types: (1) imbalance-sensitive, if the baseline was different between strategies; and (2) imbalance-insensitive, if the baseline was constant.

#### Deep Learning Model

Figure 5 displays an overview of fold-averaged performances, where strategies are paired with their baselines. Undefined metrics in edge cases were excluded. This mainly affected AUROC, where the number of proteins with metrics dropped around 25% for semi_resampling, resampling_before_clustering, and no_resampling (Table S13 of the Supporting Information). Figure 5 brought the dilemma of direct strategy comparison with imbalance-sensitive metrics, which was especially apparent for the *F*_1_-score and its high baseline in no_resampling (quartiles: *Q*_1_ = 0.428, median of 0.611, *Q*_3_ = 0.756, Table S11 of the Supporting Information).

#### Absolute, Baseline-Naïve Performance

Absolute metric models (not accounting for baselines) were fitted following eq 2, analogous to the baseline performance models. The strategy term would always explain variance (type 3 ANOVA, *p*-values ranged between 2.89 × 10^–15^ and *p* < 10^–16^; see Table S14 in the Supporting Information). The models showed different behaviors in imbalance-sensitive and -insensitive metrics (Table S15 of the Supporting Information). Pairwise comparisons of the strategy term coefficients using Tukey’s method would point to two apparently conflicting scenarios (Figure S9 of the Supporting Information), further confirmed when prioritizing the strategies according to their expected performance through the linear models (Figure 7 and Table S16 of the Supporting Information): (a) no_resampling was suggested as the best strategy by accuracy and *F*_1_-score (95% CI of expected performances: [0.701, 0.723] and [0.754, 0.779]), but this was confounded by the fact that it also held the highest baselines; and (b) resampling_before_clustering and resampling_after_clustering kept the highest performance estimates in AUROC (95% CI [0.699, 0.724] and [0.670, 0.708]), MCC (95% CI [0.244, 0.268] and [0.296, 0.337]), and balanced accuracy (95% CI [0.619, 0.640] and [0.634, 0.670]).

#### Baseline-Adjusted Performance

**Figure 6:**
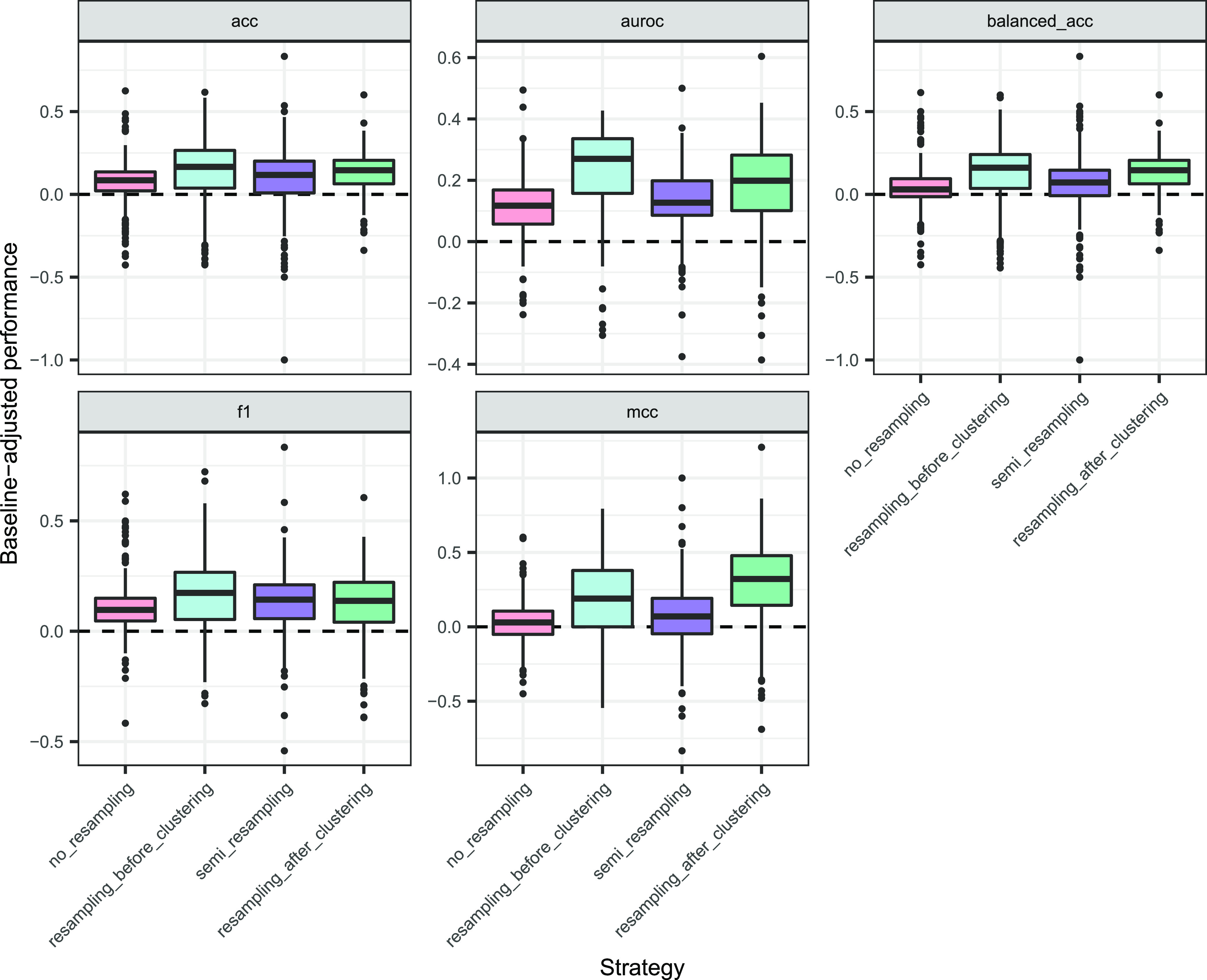
Baseline-adjusted performance metrics for balancing strategies. Data points correspond to proteins, averaged over folds. Values are positive when the DL model performs better than its paired imbalance-aware baseline, and negative otherwise.

A descriptive plot of the adjusted metrics (Figure 6) pointed to a different scenario than that of the absolute ones (Figure 5).

**Figure 7:**
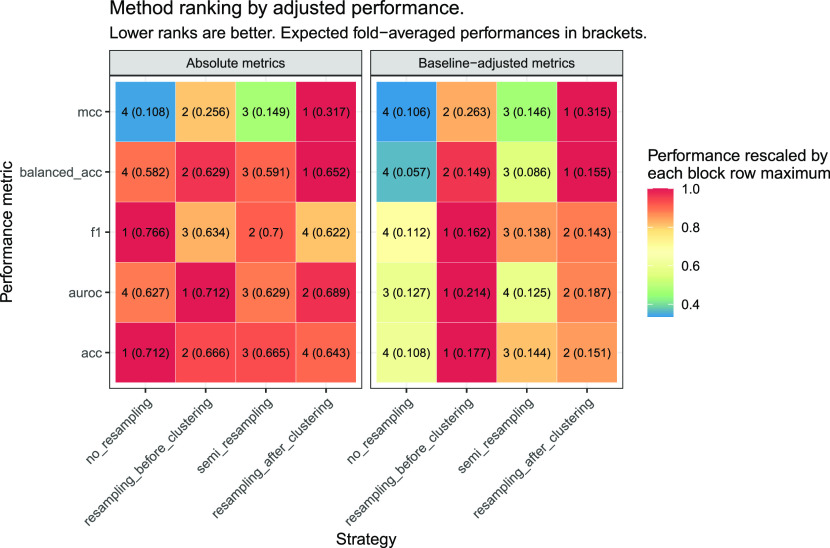
Resampling strategy ranking according to their absolute (left block) and baseline-adjusted performances (right block), estimated through the corresponding linear model of each metric. For baseline-adjusted metrics, only the improvement over the baseline is displayed. The ranking, ranging from 1 (best) to 4 (worst) in each row and block, was based on the expected performance, averaged over folds and indicated in parentheses. The color scale varies in the block row-wise between maximum (red) and 0 (blue).

Again, the strategy term was always significant (type 3 ANOVA, *p*-values ranged between 2.78 × 10^–9^ and *p* < 10^–16^; Table S17 of the Supporting Information). Baseline adjustment brought a unified behavior across the models (Table S18 of the Supporting Information), further confirmed in pairwise coefficient comparison (Tukey’s method, Figure S10 of the Supporting Information) and in their expected performance (Figure 7 and Table S19 of the Supporting Information): resampling_before_clustering and resampling_after_clustering had the highest performance estimates (expected improvements over baseline ranging from 0.149 to 0.263 and from 0.143 to 0.315 in all metrics), followed by semi_resampling (0.086–0.146) and finally by no_resampling (0.057–0.127).

### Further Validation with Other Protein Families

We repeated all of the previous analyses on three protein families to confirm whether the claims obtained for the kinase protein family could be generalized to other families. Those families were G protein-coupled receptors (GPCRs), nuclear receptors (NRs), and proteases (PRs). Appendices 3, 4, and 5 of the Supporting Information gather in detail the replication of the kinases analyses in GPCRs, NRs, and PRs. In general, the main observations and recommendations hold in GPCRs, NRs, and PRs.

Compared with kinases, GPCRs contained almost 60% more protein–compound pairs, PRs were roughly even, and NRs had about 20% of their interactions. GPCRs were more imbalanced toward the actives than kinases, while NRs and PRs kept more balanced active ratio distributions (Figure S1 of the Supporting Information).

The distributions of active ratios and the comparison between training and test set imbalances hold in GPCRs, NRs, and PRs. The only exception was semi_resampling in GPCRs, exhibiting a slightly positive correlation (Pearson’s *r* 95% CI [0.029, 0.105], *p* = 5.91 × 10^–4^) between training and test balances (see Table S20 of the Supporting Information) instead of no correlation. The effects of the number of interactions and the sequence length on the protein imbalance were also replicated on the GPCRs, NRs, and PRs.

Kinases, GPCRs, NRs, and PRs mainly agreed on the predicted active proportion analyses, except for the n_interaction coefficient, nonsignificant in the semi_resampling strategy in GPCRs, NRs, and PRs. Still, the semi_resampling model was always the clearest scenario to show that the predicted active proportions were driven by the actual proportions in the test set, rather than those in the training set.

Regarding performance, the explanatory linear models on GPCRs, NRs, and PRs also identified accuracy, *F*_1_, and balanced accuracy as sensitive to data imbalance.

The analysis of absolute metrics in GPCRs was analogous to that of kinases, while NRs and PRs showed some differences. Those mainly involved which metrics place no_resampling as the best strategy (accuracy, balanced accuracy, and *F*_1_-score for NRs; and also AUROC in PRs) and resampling_after_clustering not being suggested as the best strategy anymore.

As for adjusted performances, GPCRs showed essentially the same facts as kinases. In NRs and PRs, resampling_before_clustering still showed the best performance in general, but resampling_after_clustering lost its shared dominance with the former. This implied that augmenting the test set was not the largest performance drive anymore, which might be explained by the more moderate data imbalance in NRs and PRs as compared to kinases and GPCRs. On the other hand, our main recommendation remained unchanged, since semi_resampling still compared favorably to no_resampling, with less significant changes (especially in NRs with their sensibly reduced sample size), but always in favor of the former if present.

## Discussion

### Impact of Clustering in Final Imbalance Was Strategy-Dependent

This study is focused on the characterization of the data imbalance present in bioactivity data sets, as well as how to address it. Bioactivity data also poses the problem of chemical series, i.e., sets of similar molecules with similar activities, that result in inflated performance metrics when split between training and test sets. We addressed those via a clustering prior to the splitting, ensuring that similar molecules would belong to the same set.

The first observation was that clustering modified data imbalance in a strategy-dependent way. When the starting set was perfectly balanced (strategy resampling_before_clustering), clustering and splitting induced a degree of imbalance, particularly visible in the heavier tails of the active ratio distributions in the test set. Compared to training, the lower sample sizes in the test set may also cause extreme imbalances more often. On the other end, this effect was only moderate in no_resampling, where the distribution of the active ratio was similar in training and test, but that of the test had more extreme proteins with either all actives or all inactives.

Besides the overall changes in data imbalance, strategies differed in how the imbalance of a certain protein in the training set would translate to the test set. The positive trend in no_resampling suggests that existing data imbalances tended to persist after the clustering and splitting. The negative trend in resampling_before_clustering hints that, in the absence of imbalance, clustering will induce it. The flat trend in semi_resampling supports the fact that the imbalance induced with the clustering in the training set, which was balanced with SMOTE beforehand, is independent of the original imbalance in the data set (present in the test set).

### Predicted Active Proportion Was Driven by the Test Set rather than the Training

The original distribution of the active ratio in each of the balancing strategies affected the predicted ratio of actives by the models. Due to the lack of correlation between training and test ratios (Figure 3), the semi_resampling strategy was the ideal scenario to disentangle their effect on the predicted ratio of actives (see the model in Table S4 of the Supporting Information). Its additive model suggested that the original ratio of actives in the test explained the predicted proportions, rather than the training ratio. We also found that the number of interactions per protein was a relevant factor: the more the interactions, the less the proportion of actives, suggesting that the extreme cases with all predicted as actives tended to be proteins with few interactions.

Likewise, resampling_before_clustering showed a negative correlation between training and test ratios, also providing a reasonably good scenario to distinguish their effects (Table S4 from the Supporting Information). Its explanatory model confirmed both conclusions from the model in the semi_resampling strategy, with similar estimates (Table S9).

The explanatory model for the no_resampling strategy (Table S10 of the Supporting Information) suffered from the positive correlation between training and test ratios, which could be confounded. Both original training and test ratios showed a positive effect on the predicted fraction of actives. Although the estimate was larger and more significant for the training ratio coefficient, the confounding effect and the very skewed distribution of the predicted ratios deemed this model inconclusive.

### Imbalance-Sensitive Metrics Required Baseline Adjustment

The prediction task studied here posed a particular challenge: data imbalance happened on a protein basis, and the imbalance of certain proteins could be extreme (very low or high), moving away from the global ratio of actives. Each resampling strategy would lead to different protein-wise imbalance patterns. The baseline performance of some metrics (accuracy, *F*_1_-score, and balanced accuracy) was different between strategies, while it was constant for others (AUROC and MCC). The data-driven division into imbalance-sensitive and -insensitive metrics was an important step to understand the opposite conclusions reached within each metric type after direct performance comparison between strategies (Figure 7).

The direct comparison of resampling strategies with imbalance-sensitive metrics would be confounded by the imbalance-induced bias in the metrics and the protein-wise imbalance differences between strategies. We found that adjusting by the baseline metrics (see eq 4) brought an agreement in the conclusions obtained by both imbalance-sensitive and -insensitive metrics. In turn, the same conclusions were obtainable by direct comparison of imbalance-insensitive metrics. Because of this, our recommendation is to include imbalance-aware baselines and to adjust imbalance-sensitive metrics when used for model selection.

### Augmenting the Test Set Was the Largest Performance Drive

Our results showed that the largest impact on performance estimates was the application of data augmentation to the test set: resampling_before_clustering and resampling_after_clustering tended to outperform semi_resampling and no_resampling. However, augmenting the test set might not faithfully reflect new data anymore and could artificially inflate the performance estimates: models may specialize in discriminating between original and resampled data points instead of actives and inactives. Our validation with other protein families (NRs, PRs) suggested that this fact might not apply when the interaction data is more balanced.

### Resampling Improved Performance when Keeping the Original Test Set

On the other hand, semi_resampling outperformed no_resampling in four out of five metrics (Tukey’s method, *p* < 0.05, Figure S10 of the Supporting Information), which supported data augmentation usefulness even if the data balance in the test set differed from that of the training set. This was consistent with the observation that the main influence on the predicted active ratio in the test set was their actual ratios in the test set instead of the original ratios in the training set. Combined with the less skewed distributions of predicted active ratios of semi_resampling against no_resampling (Figure 2), we recommend semi_resampling for future studies.

### Using External Protein Family Data Sets for Validation Suggests Replicability of the Main Guidelines

The results obtained by the kinases and those of the GPCR, NR, and PR proteins, used as external validation sets for model fitting and evaluation, pointed to the same general picture with aligned conclusions. The differences could arise from changes in data imbalance (NRs and PRs were less imbalanced, while GPCRs were more) and number of protein–compound pairs (GPCRs had more interactions, while PRs had less). The variety of scenarios under consideration suggests that the guidelines for proteochemometric models of our study provide sensible defaults to more protein families.

### Similarities with Existing Literature

In this paper, we have confirmed that data balance has an impact on DL proteochemometric target–compound activity models. Zakharov et al. and Korkmaz arrived at a similar conclusion in a QSAR setting,^15,16^ the latter also using DNN models for classification. More specifically, Korkmaz stated that the higher the imbalance for a protein, the worse the model performance (measured by *F*_1_-score and MCC).

These studies achieved the best performances by controlling data balance by means of undersampling techniques (in the case of Zakharov) and oversampling techniques (in the case of Korkmaz). We chose SMOTE for data balancing, an oversampling technique, since the settings of the Korkmaz study were more aligned with ours and because DL models require a large quantity of training data. Specifically, in four out of five metrics, proteins with more interactions were better-predicted (Table S18 of the Supporting Information), which was also found in the Korkmaz paper.

Within our resampling strategies, semi_resampling was the most similar to the balancing process in the Korkmaz study, in which the training and validation sets were oversampled (per protein), while the test set was not.

### Dissimilarities with Existing Literature

Technical differences existed in the descriptors used in the three studies. Zakharov et al. used Quantitative Neighborhood of Atoms and biological descriptors, whereas Korkmaz used PaDEL software. We, on the other hand, used the fingerprints from PubChem. The fact that the overall messages are consistent suggests a degree of independence from the input encoding.

More importantly, the studies of Zakharov and Korkmaz did not take into account the control of the compound series bias. This step is necessary for obtaining realistic performance estimates in a real-world setting.^8,18^ Not only did we account for it, but we also investigated if the stage in which the compound series control was introduced, in combination with the data augmentation (before or after applying SMOTE), had an impact on the outcome.

Indeed, the order had an impact on the model performance and needed careful consideration. Resampling_before_clustering solved the global imbalance of the data set, but clustering after oversampling would lead again to a proteinwise imbalance. Analogously, semi_resampling resampled the training and validation sets, but imbalance returned after their clustering. On the contrary, resampling_after_clustering first corrected the problem of similar compounds and then augmented the data to reach a protein-wise balance.

### Limitations and Future Work

This study continues our incremental work on recommendations for DL models regarding input encoding^25^ and control of chemical series.^8^ While this study was limited to one architecture and four protein families, it provides a foundation to understand the basic behavior of PCM models, insights into how to adjust performance metrics for a proteinwise analysis, and a first step toward exploring more general questions. Those could include architecture-centric analyses to confirm if the same trends are observed when changing the layers or the model structure or using other protein families with a different distribution of active ratios to those studied in this analysis.

## Conclusions

Although the effect of data balance and resampling techniques had been analyzed for QSAR models, it had not been studied yet in the context of proteochemometric models, even if the bioactivity data sets used in this setting are usually imbalanced. In this paper, we have tested four different combinations of data oversampling (through SMOTE) and clustering for controlling compounds’ similarity. While the clustering avoids overly optimistic performance estimates, it could introduce more data imbalance (in the form of splittings having proteins with mostly active or inactive compounds). Despite this potential conflict between the resampling and the clustering, we found that resampling was useful to improve the model behavior and performance.

Some common performance metrics were affected by the data imbalance and yielded misleading trends. We included an imbalance-aware random baseline and defined baseline-adjusted metrics to overcome this issue, especially in *F*_1_-score and accuracy. After baseline adjustment, the metrics provided a unified picture: the largest impact on performance estimates came from the application of data augmentation to the test set (resampling_before_clustering and resampling_after_clustering outperformed semi_resampling and no_resampling). However, augmenting the test set may not reflect a realistic scenario.

On the other hand, semi_resampling outperformed no_resampling in four out of five adjusted metrics and provided a more equalized distribution of the predicted active ratio. This confirmed the data augmentation usefulness even if the data balance in the test set differed from that of the training set. This was consistent with the finding that the predicted proportion of positives of the proteochemometric model was explained by the actual data balance in the test set, rather than that of the training set. We also found that proteins with more interactions were better predicted.

Our recommendation is thus to use the semi_resampling strategy, i.e., clustering compounds to separate training and validation from test sets, resampling training and validation, and then clustering compounds again to definitely split training and validation sets. This was carried out on the kinase protein family and further confirmed on the GPCR, NR, and PR protein families. While we cannot extrapolate these results to all of the proteins and imbalance distributions, this sets a sensible starting point for improving proteochemometric modeling and remains consistent with the corresponding data imbalance studies on QSAR models.

## Supporting Information Available

Detailed explanation of the material, deep learning model architecture, performance metrics and complementary results of the analysis (explanatory model tables, significance tests, descriptive figures); four appendices with the complete analysis for kinases (2), GPCRs (3), NRs (4), and PRs (5); claims specific for each of the protein families are bolded (PDF)

## Notes

The bioactivity data used in our analysis is publicly available in the repository https://github.com/Shen-Lab/DeepAffinity.^19^ The code of this analysis is publicly available at https://github.com/b2slab/imbalance_pcm_benchmark.
