# Supplementary material for: Balancing Data on Deep Learning-Based Proteochemometric Activity Classification
Source: J Chem Inf Model. 2021 Mar 29;61(4):1657–69. doi: 10.1021/acs.jcim.1c00086 (PMC8594867; doi:10.1021/acs.jcim.1c00086)
Supplement: Supplementary file 1 — ci1c00086_si_001.pdf [file ci1c00086_si_001.pdf]

Supporting Information

Balancing data on deep learning-based proteochemometrics activity classification

Angela Lopez-del Rio,*[,†,‡] Sergio Picart-Armada,[†,‡] and Alexandre Perera-Lluna[†,‡]

†*B2SLab, Departament d'Enginyeria de Sistemes, Automàtica i Informàtica Industrial, Universitat Politècnica de Catalunya, 08028 Barcelona, Spain.*

‡*Department of Biomedical Engineering, Institut de Recerca Pediàtrica Hospital Sant Joan de Déu, Esplugues de Llobregat, 08950 Barcelona, Spain.*

E-mail: angela.lopez.del.rio@upc.edu

Appendix 1: supporting information

The Appendix 1 gathers all the tables and figures referenced from the Supporting Information through the main text.

Materials and methods

Table S1 summarizes the GPCRs, nuclear receptors and proteases subdatasets, used for validating the results obtained in the kinases family.

On the other hand, table S2 displays the number of active and inactive protein-compound pairs, averaged over folds, that were available in each set (training, validation, test) per strategy and protein family.

In Figure S1, an histogram with the proportion of actives respect to all the interactions per protein is shown for kinases, GPCRs, nuclear receptors and proteases. It can be seen

Table S1: Summary of the GPCRs, nuclear receptors (NR) and proteases (PR) subdatasets.

	GPCR	NR	PR
Entity		#	
Compounds	120,906	18,120	58,882
Targets	498	82	319
Ligand-target pairs	200,523	26,433	92,965
Actives	153,993	16,962	57,510
Inactives	46,530	9,471	35,455

Table S2: Total number of protein-compound pairs in each strategy and protein family, displayed in the actives/inactives format. Number of actives and inactives were averaged over the 10 folds.

protein_type	strategy	training	validation	test
kinases	no_resampling	75767/23511	10970/3429	10215/3045
kinases	resampling_after_clustering	76897/76897	9778/9778	8955/8955
kinases	resampling_before_clustering	78102/77361	9899/10456	10374/10558
kinases	semi_resampling	77951/77539	9893/10305	10215/3045
GPCRs	no_resampling	121449/36876	16315/4919	16228/4734
GPCRs	resampling_after_clustering	121097/121097	14355/14355	13930/13930
GPCRs	resampling_before_clustering	121108/120957	15613/15307	17235/17692
GPCRs	semi_resampling	122132/120912	15487/16708	16228/4734
nuclear_receptors	no_resampling	13333/7479	1824/997	1804/994
nuclear_receptors	resampling_after_clustering	14218/14218	1760/1760	1678/1678
nuclear_receptors	resampling_before_clustering	14123/14285	1912/1856	2074/1966
nuclear_receptors	semi_resampling	14372/14398	1798/1771	1804/994
proteases	no_resampling	44954/28098	6314/3586	6243/3770
proteases	resampling_after_clustering	50558/50558	6323/6323	6229/6229
proteases	resampling_before_clustering	50891/51481	6766/6778	7217/6616
proteases	semi_resampling	51091/51221	6604/6474	6243/3770

that for both kinases GPCRs (but specially for GPCRs) there are more actives per protein than inactives. There is a large number of GPCR proteins for which only active interactions are reported. For proteases the distribution is more even.

In Figure S2, the number of active and inactive interactions for each protein is represented for kinases, GPCRs, nuclear receptors and proteases. In all cases, there are many proteins for which there are only active or inactive interactions, thus the median is near 0. There are also many proteins (shown as outliers) with a large number of active interactions.

As stated in the main text, proteins whose sample size in a specific fold or strategy did

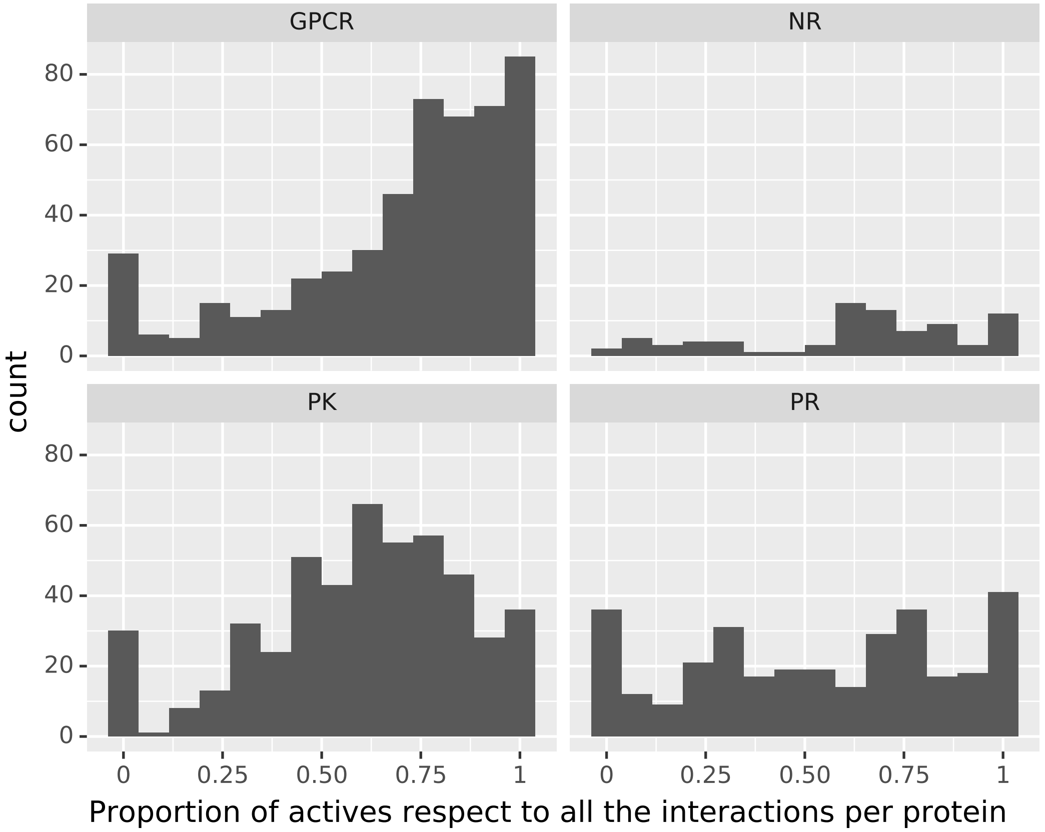

Figure S1: Histogram of proportion of actives respect to the total number of interactions for each protein for the protein kinases (PK), GPCRs, nuclear receptors (NR) and proteases (PR) families.

not allow SMOTE upsampling were excluded. Table S3 shows the number of protein kinases for which performance metrics were computed in each case.

Table S3: Number of proteins for which performance metrics were computed. The resampling after clustering was the most stringent strategy regarding eligible proteins, since the resampling was carried out after the clustering, which introduced more imbalance.

Strategy	Fold 0	Fold 1	Fold 2	Fold 3	Fold 4	Fold 5	Fold 6	Fold 7	Fold 8	Fold 9
no_resampling	288	282	295	303	305	305	293	307	294	301
resampling_before_clustering	271	295	284	274	298	286	301	307	302	293
semi_resampling	288	282	295	303	305	305	293	307	294	301
resampling_after_clustering	79	74	72	84	76	79	87	82	81	100

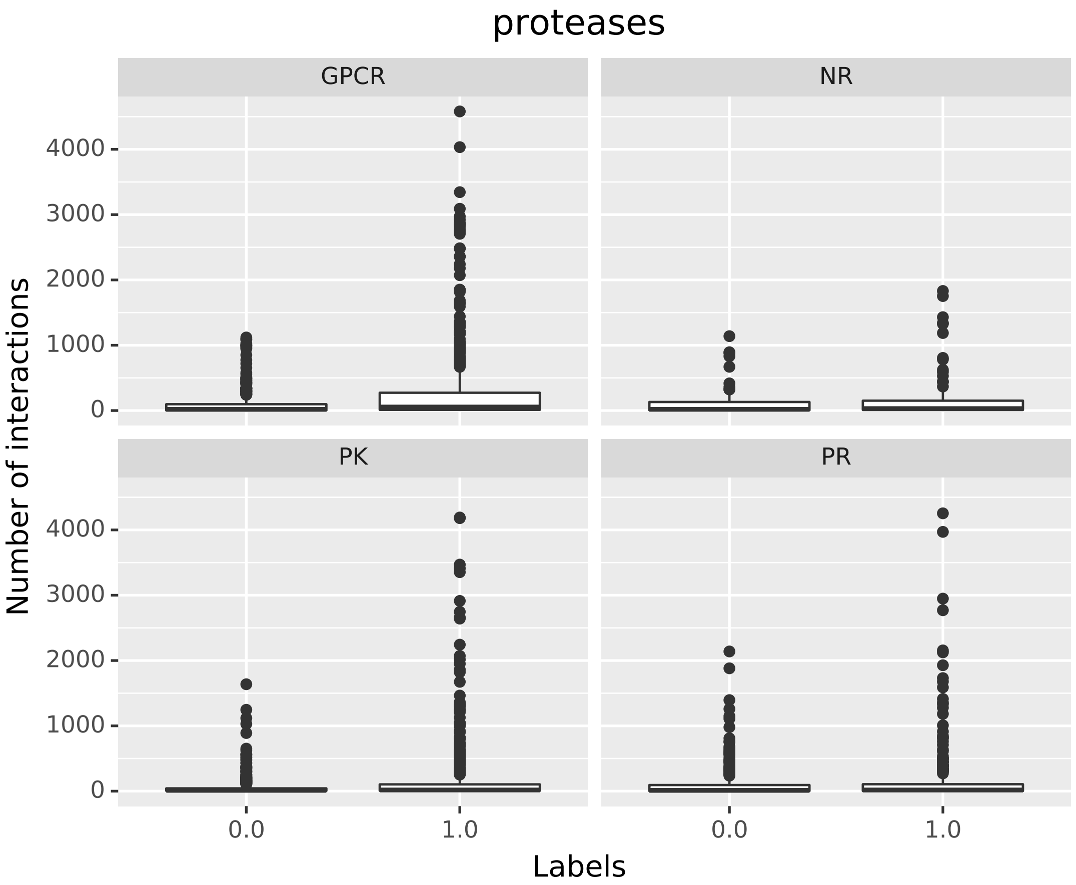

Figure S2: Number of active (1) and inactive (0) interactions per protein for the protein kinase (PK), GPCRs, nuclear receptors (NR) and proteases (PR) familie. Each data point is a protein from the corresponding family.

Model architecture

The schematic representation of the deep learning model is displayed in Figure S3. The selected parameters were drop_hid=0.4, n_1=50, f1=64 and k1=3 . As activation function, we used Rectified Linear Unit (ReLU) for the hidden layers,[1] and *softmax* for the output dense(2) layer.

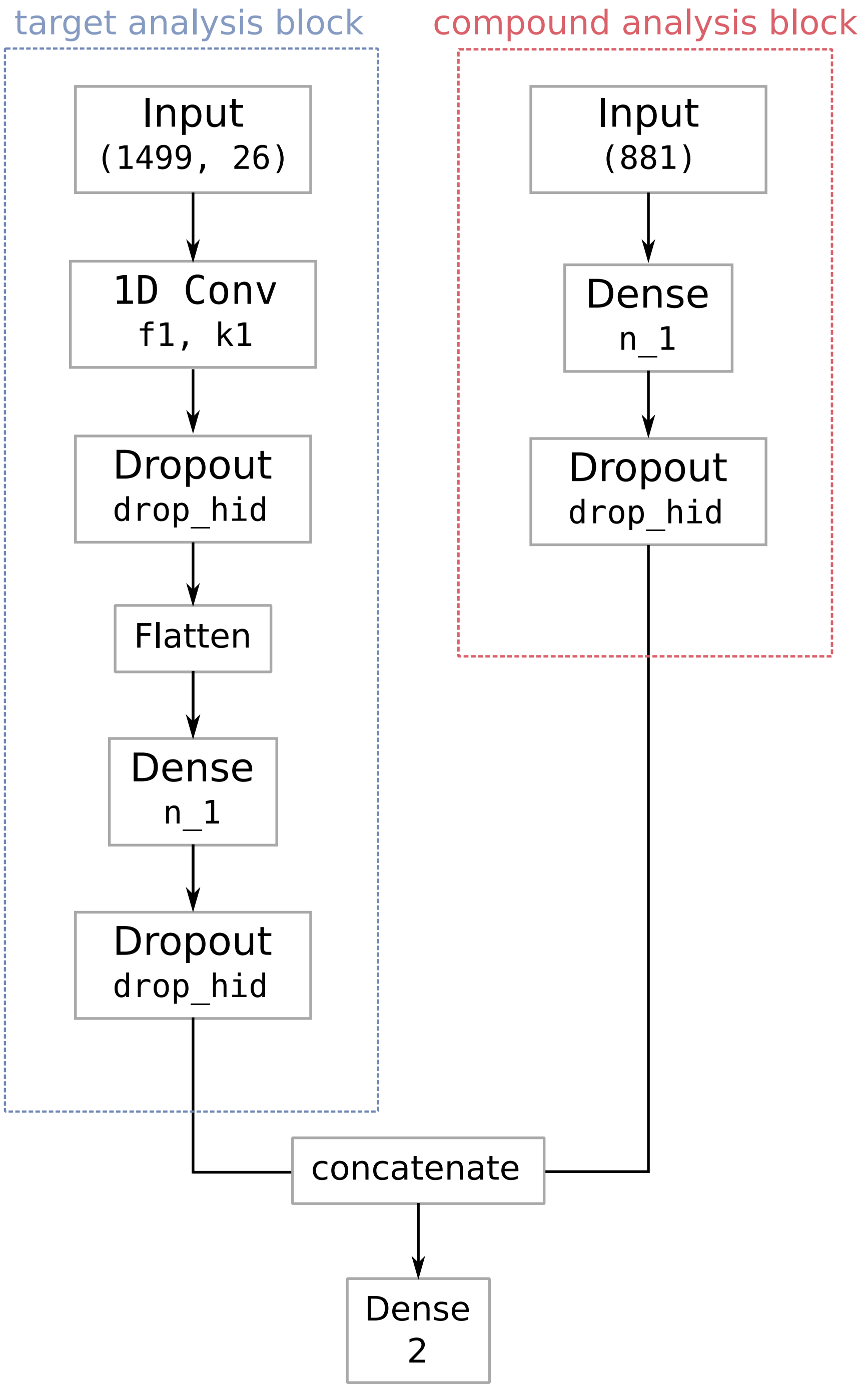

Figure S3: Schematic representation of the deep learning architecture. It is composed of two main blocks: the protein analysis block and the compound analysis block.

Performance metrics

The description of the metrics used for evaluating and comparing the performance of the different balancing strategies is shown below. Let TP be the number of true positive classified samples, TN the true negatives, FP the false positives and FN the false negatives:

$$Accuracy = \frac{(TP + TN)}{(TP + FP + TN + FN)}$$

.

Since accuracy is not a proper metric when the dataset is highly imbalanced, we used balanced accuracy, which is the average between sensitivity and specificity:

$$Balanced\ accuracy = \left[\frac{TP}{TP + FN} + \frac{TN}{TN + FP}\right]/2$$

If $precision = \frac{TP}{TP+FP}$ and $recall = \frac{TP}{TP+FN}$, then F1-score can be described as

$$F_1 = 2 \cdot \frac{precision \cdot recall}{precision + recall}$$

The macro F1-score calculates metrics for each label, and finds their unweighted mean. This does not take label imbalance into account.

The Matthews correlation coefficient (MCC) is alue between -1 and +1: +1 represents a perfect prediction, 0 an average random prediction and -1 an inverse prediction. This metric is not sensitive to imbalance, so it can be used even if the classes are of very different sizes. It can be calculated using the formula:

$$MCC = \frac{(TP \times TN - FP \times FN)}{\sqrt{(TP + FP)(TP + FN)(TN + FP)(TN + FN)}}$$

.

The Area Under the Curve (AUC) of the Receiver Operating Characteristic (ROC) curve measures performance in classification problems for different thresholds. The ROC curve is

a probability curve obtained by plotting the True Positive Rate (which is the same as the recall) on y-axis against the False Positive Rate (which is $\frac{FP}{TN+FP}$) on the x-axis. The AUC, which is the area under this curve, quantifies how capable the model is of distinguishing both classes.

Results

From now on, unless it is stated otherwise, all the figures and tables presented in the Results section of this Supplementary Material show results for the protein kinases dataset.

Description of data balance

Distributions of actives ratio

In Figure S4, the distributions of the proportion of active molecules for a protein for each strategy both on training and test sets is shown.

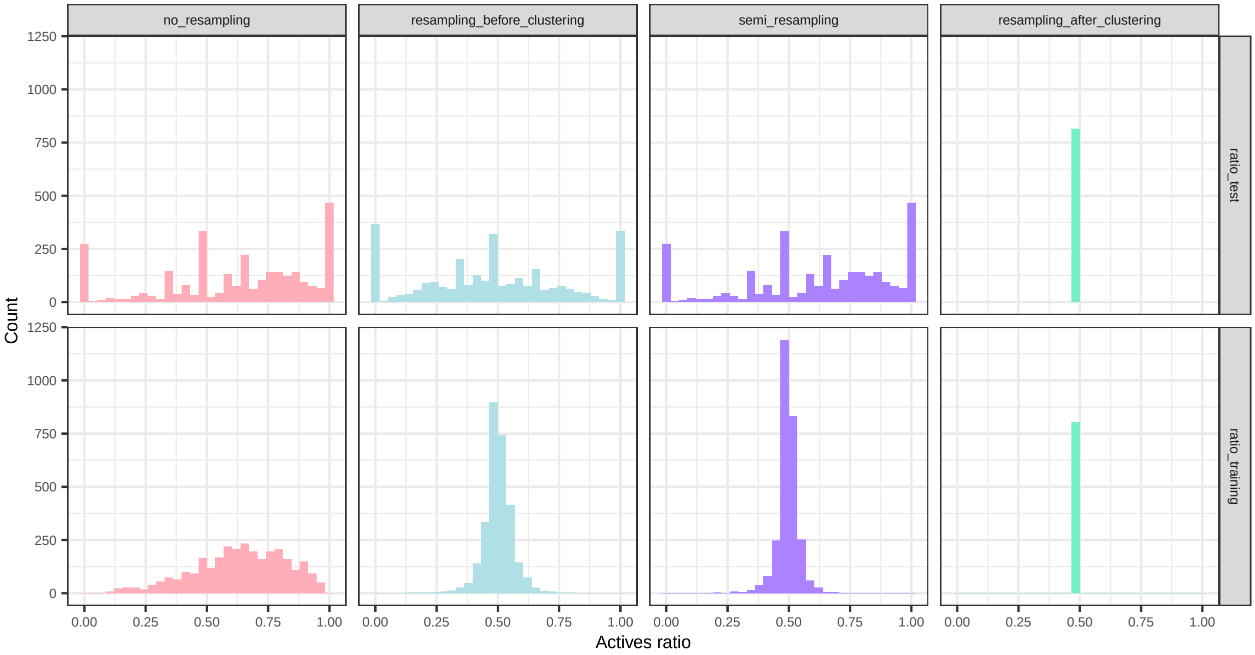

Figure S4: Distribution of the actives ratio in the training set and in the test set.

Training and test imbalance comparison

Table S4 shows the Pearson correlation estimate, 95% interval and p-value for each strategy (except resampling_after_clustering, where ratios are constant).

Table S4: Correlations between train and test active ratios. 95% confidence intervals and p-values are shown.

strategy	cor	ci_lower	ci_upper	alternative	pvalue
no_resampling	0.369	0.338	0.400	two.sided	1.15e-96
resampling_before_clustering	-0.428	-0.457	-0.398	two.sided	5.54e-130
semi_resampling	0.014	-0.024	0.051	two.sided	4.76e-01
resampling_after_clustering	NA	NA	NA	two.sided	NA

Other covariates

Figure S5 shows the effect of the number of interactions of each protein in its corresponding set and fold on the test set imbalance. Figure S6 shows the effect of the protein length on the test set imbalance.

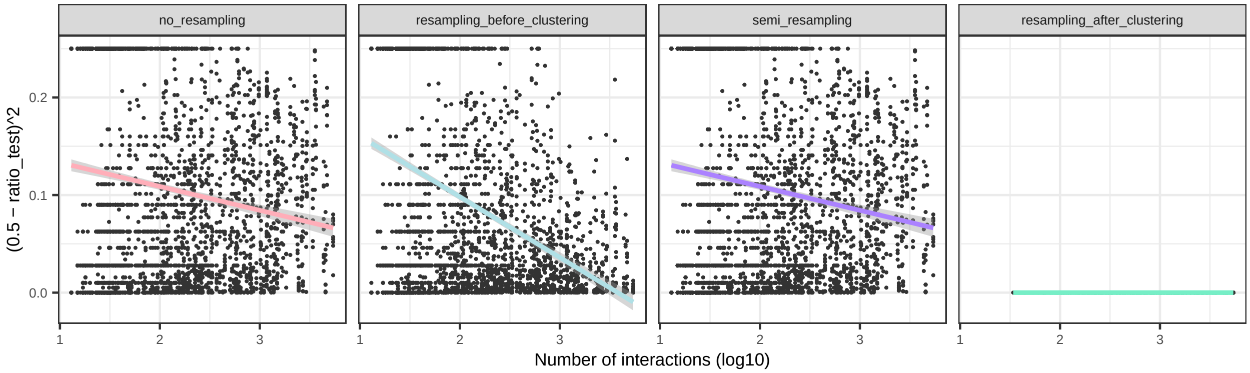

Figure S5: Data imbalance in the test set as a function of the number of available interactions for each protein. The imbalance was quantified as $(0.5 - ratio_test)^2$ in order to measure the deviation from a perfectly balanced protein, with an actives ratio of 0.5.

Pearson correlation estimates, 95% interval an p-value between imbalance and number of interactions for each strategy is shown in Table S5. The same information but for the correlations between imbalance and sequence length is in Table S6.

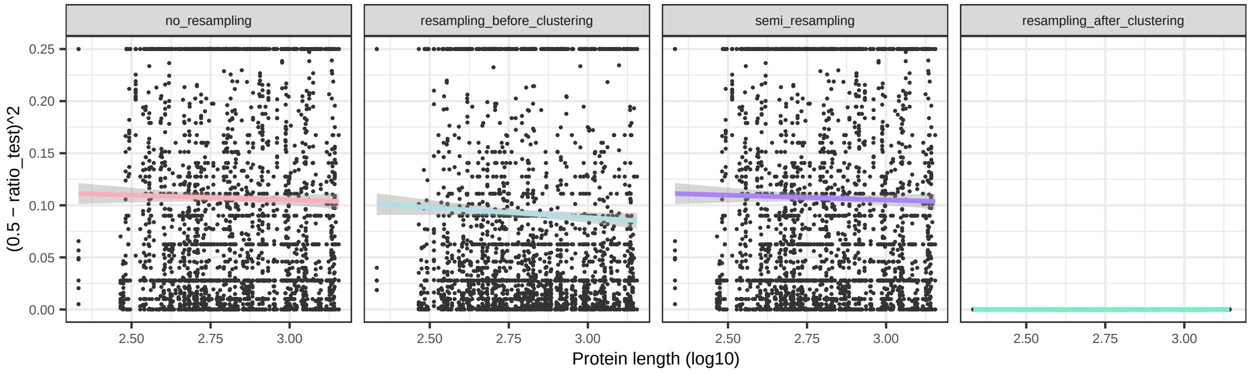

Figure S6: Data imbalance in the test set as a function of the protein length, in amino acids.

Table S5: Correlations between imbalance (as defined above) and number of interactions. 95% confidence intervals and p-values are shown.

strategy	cor	ci_lower	ci_upper	alternative	pvalue
no_resampling	-0.061	-0.097	-0.026	two.sided	8.01e-04
resampling_before_clustering	-0.274	-0.307	-0.240	two.sided	3.98e-51
semi_resampling	-0.061	-0.097	-0.026	two.sided	8.01e-04
resampling_after_clustering	NA	NA	NA	two.sided	NA

Table S6: Correlations between imbalance (as defined above) and sequence length. 95% confidence intervals and p-values are shown.

strategy	cor	ci_lower	ci_upper	alternative	pvalue
no_resampling	-0.016	-0.052	0.020	two.sided	3.73e-01
resampling_before_clustering	-0.046	-0.082	-0.009	two.sided	1.37e-02
semi_resampling	-0.016	-0.052	0.020	two.sided	3.73e-01
resampling_after_clustering	NA	NA	NA	two.sided	NA

Analysis of the predicted proportions

Pearson correlation estimates, 95% interval an p-value between training and predicted test active ratios for each strategy is shown in Table S8.

In Table S7 it is shown the percentage of extreme cases for each strategy.

The quasibinomial generalized linear models describing the predicted active ratio are depicted in Table S9 for the semi_resampling and resampling_before_clustering strategy and in Table S10 for the no_resampling.

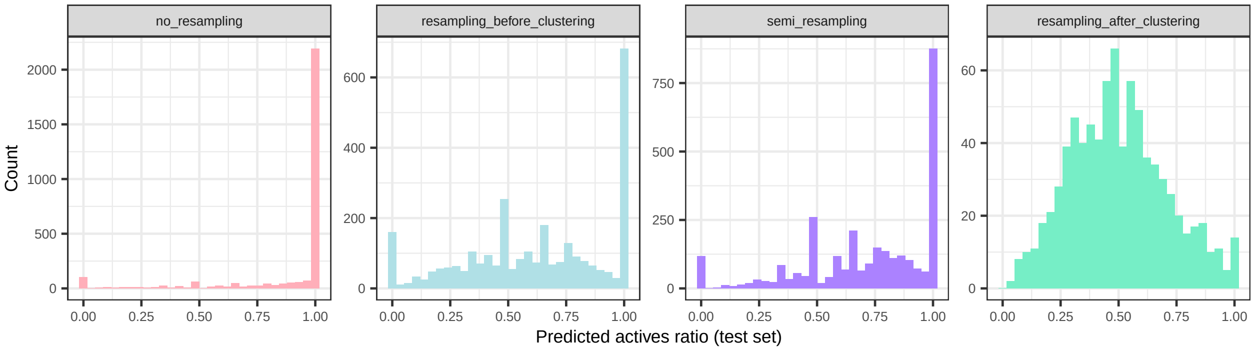

Figure S7: Ratios of the prediction values, after binarization.

Table S7: Percentage of extreme cases, i.e. proteins with all actives or inactives.

strategy	RatioSet	all_inactives	all_actives	all_extremes
no_resampling	ratio_test	9.2	15.5	24.7
no_resampling	ratio_test_predicted	3.5	71.6	75.1
no_resampling	ratio_training	0.0	0.0	0.0
resampling_before_clustering	ratio_test	12.5	11.5	24.0
resampling_before_clustering	ratio_test_predicted	5.5	23.4	28.9
resampling_before_clustering	ratio_training	0.0	0.0	0.0
semi_resampling	ratio_test	9.2	15.5	24.7
semi_resampling	ratio_test_predicted	4.0	29.1	33.1
semi_resampling	ratio_training	0.0	0.0	0.0
resampling_after_clustering	ratio_test	0.0	0.0	0.0
resampling_after_clustering	ratio_test_predicted	0.0	1.2	1.2
resampling_after_clustering	ratio_training	0.0	0.0	0.0

Table S8: Correlations between train and predicted test active ratios. 95% confidence intervals and p-values are shown.

strategy	cor	ci_lower	ci_upper	alternative	pvalue
no_resampling	0.469	0.440	0.496	two.sided	2.79e-162
resampling_before_clustering	-0.094	-0.130	-0.058	two.sided	3.77e-07
semi_resampling	0.008	-0.029	0.045	two.sided	6.78e-01
resampling_after_clustering	NA	NA	NA	two.sided	NA

Table S9: Explanatory models to describe the predicted active ratio for the semi_resampling and the resampling_before_clustering strategies. Significance and 95% confidence intervals are included.

	semi_resampling	resampling_before_clustering
	(1)	(2)
ratio_training	0.197 (−0.903, 1.298)<br>p = 0.725	−0.446 (−1.296, 0.405)<br>p = 0.305
ratio_test	0.945 (0.775, 1.114)*** p = 2.460e-27	0.784 (0.606, 0.963)*** p = 1.181e-17
log10(n_interactions)	−0.391 (−0.467, −0.314)*** p = 3.197e-23	−0.396 (−0.466, −0.325)*** p = 1.987e-27
log10(len_seq)	0.289 (0.023, 0.554)* p = 0.033	−0.033 (−0.293, 0.226)<br>p = 0.801
fold1	0.034 (−0.176, 0.245)<br>p = 0.748	0.071 (−0.138, 0.281)<br>p = 0.504
fold2	−0.651 (−0.852, −0.45)*** p = 2.348e-10	0.416 (0.196, 0.635)** p = 2.076e-04
fold3	0.982 (0.74, 1.224)*** p = 2.598e-15	−0.436 (−0.646, −0.226)** p = 4.738e-05
fold4	0.665 (0.44, 0.891)*** p = 8.708e-09	0.326 (0.114, 0.538)* p = 2.609e-03
fold5	0.023 (−0.187, 0.232)<br>p = 0.831	0.333 (0.118, 0.548)* p = 2.413e-03
fold6	−0.524 (−0.725, −0.323)*** p = 3.457e-07	0.021 (−0.186, 0.229)<br>p = 0.839
fold7	0.626 (0.402, 0.849)*** p = 4.506e-08	0.377 (0.165, 0.589)** p = 5.045e-04
fold8	0.504 (0.283, 0.725)** p = 8.130e-06	0.378 (0.165, 0.592)** p = 5.250e-04
fold9	−8.292e-03 (−0.215, 0.199)<br>p = 0.937	−0.73 (−0.938, −0.522)*** p = 7.301e-12
Constant	0.177 (−0.76, 1.113)<br>p = 0.712	1.229 (0.34, 2.118)* p = 6.796e-03
Observations	2783	2911
Note:		*p<0.05; **p<1.000e-03; ***p<1e-06

Table S10: Explanatory models to describe the predicted active ratio for the no_resampling strategy. Significance and 95% confidence intervals are included.

	no_resampling
ratio_training	8.312 (7.581, 9.042)***
	p = 0.000e+00
ratio_test	1.102 (0.741, 1.464)***
	p = 2.600e-09
log10(n_interactions)	−1.24 (−1.422, −1.058)***
	p = 1.330e-39
log10(len_seq)	−0.949 (−1.513, −0.386)**
	p = 9.683e-04
fold1	−0.505 (−0.931, −0.078)*
	p = 0.021
fold2	−0.034 (−0.478, 0.411)
	p = 0.881
fold3	1.063 (0.545, 1.58)**
	p = 5.828e-05
fold4	−0.805 (−1.21, −0.4)**
	p = 9.970e-05
fold5	0.917 (0.411, 1.423)**
	p = 3.862e-04
fold6	1.709 (1.097, 2.321)***
	p = 4.711e-08
fold7	0.169 (−0.278, 0.615)
	p = 0.459
fold8	0.116 (−0.341, 0.574)
	p = 0.618
fold9	0.886 (0.385, 1.387)**
	p = 5.365e-04
Constant	1.904 (0.269, 3.539)*
	p = 0.023
Observations	2973
Note:	*p<0.05; **p<1.000e-03; ***p<1e-06

Performance metrics

Baseline performance

Figure S8 shows a fold-averaged picture of the metrics by protein.

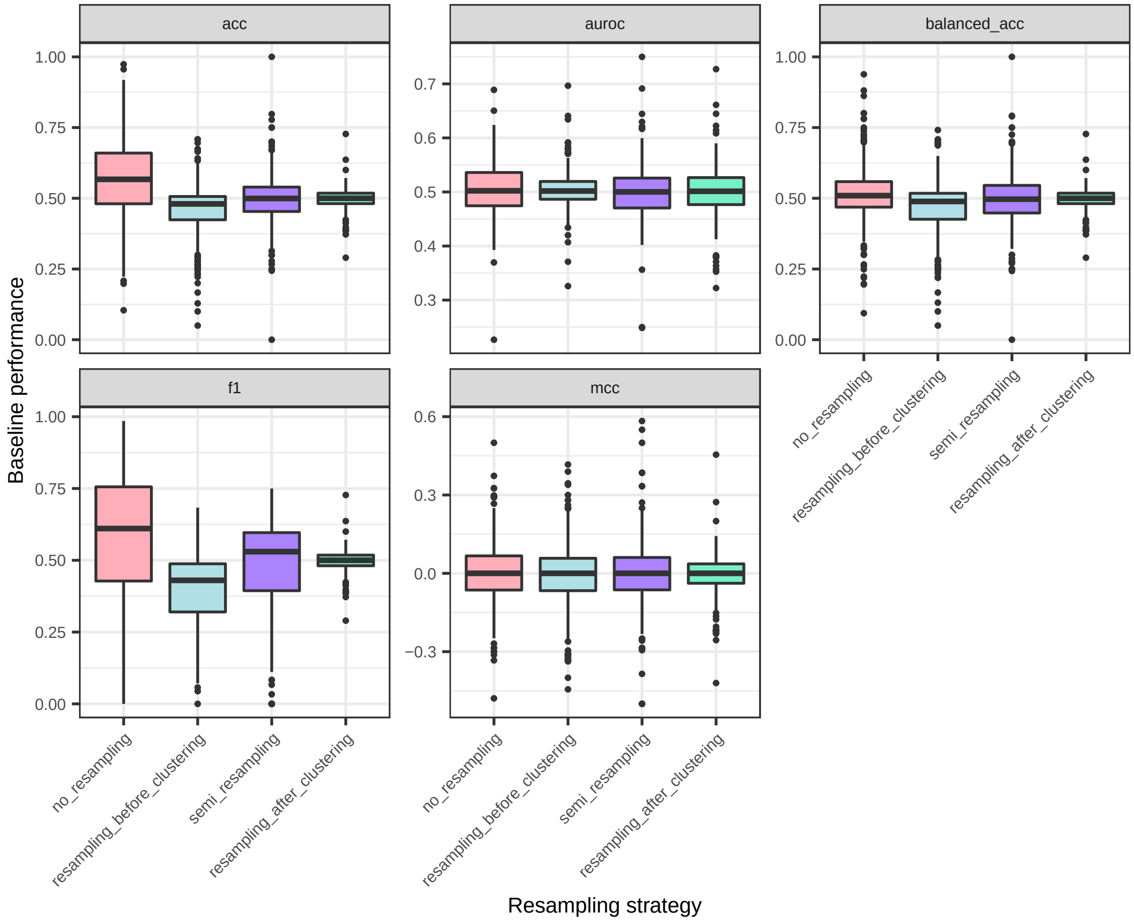

Figure S8: Performance metrics for imbalance-aware random baselines. Data points correspond to proteins, averaged over folds.

The quartiles for the baseline F1-scores are in Table S11.

Table S12 shows the type 3 ANOVA p-values of the strategy variable in the explanatory linear model of Equation 2 (main text) applied to the random baseline metrics.

Table S11: Quartiles for the baseline F1-scores.

strategy	Min.	1st Qu.	Median	Mean	3rd Qu.	Max.
no_resampling	0.000	0.428	0.611	0.569	0.756	0.985
resampling_before_clustering	0.000	0.320	0.430	0.392	0.488	0.683
semi_resampling	0.000	0.394	0.530	0.478	0.596	0.750
resampling_after_clustering	0.290	0.481	0.500	0.497	0.518	0.727

Table S12: ANOVA p-values for including the resampling strategy as a regressor. Significant p-values imply that differences exist between resampling strategies.

strategy	variable	Sum Sq	Df	F value	Pr(>F)
acc	strategy	22.2816465	3	1.36e+02	5.58e-86
auroc	strategy	0.0324079	3	1.79e-01	9.11e-01
balanced_acc	strategy	3.0761723	3	1.70e+01	5.61e-11
f1	strategy	53.2175335	3	2.36e+02	8.97e-148
mcc	strategy	0.1219509	3	3.03e-01	8.24e-01

Deep Learning model

Table S13 summarizes the number of proteins, added over folds, whose metrics were computable.

Table S13: Number of computable performance measures. AUROC was undefined for proteins with all actives or unactives in the test set, hence its lower counts.

strategy	acc	auroc	f1	balanced_acc	mcc
no_resampling	2973	2238	2973	2973	2973
resampling_before_clustering	2911	2211	2911	2911	2911
semi_resampling	2973	2238	2973	2973	2973
resampling_after_clustering	814	814	814	814	814

Absolute, baseline-naive performance The strategy covariate was always significant in a type 3 ANOVA (table S14). The explanatory linear models are summarized in S15.

Pairwise comparisons of the strategy coefficients using Tukey's method are shown in Figure S9.

Table S16 shows the expected absolute performances by metric and strategy.

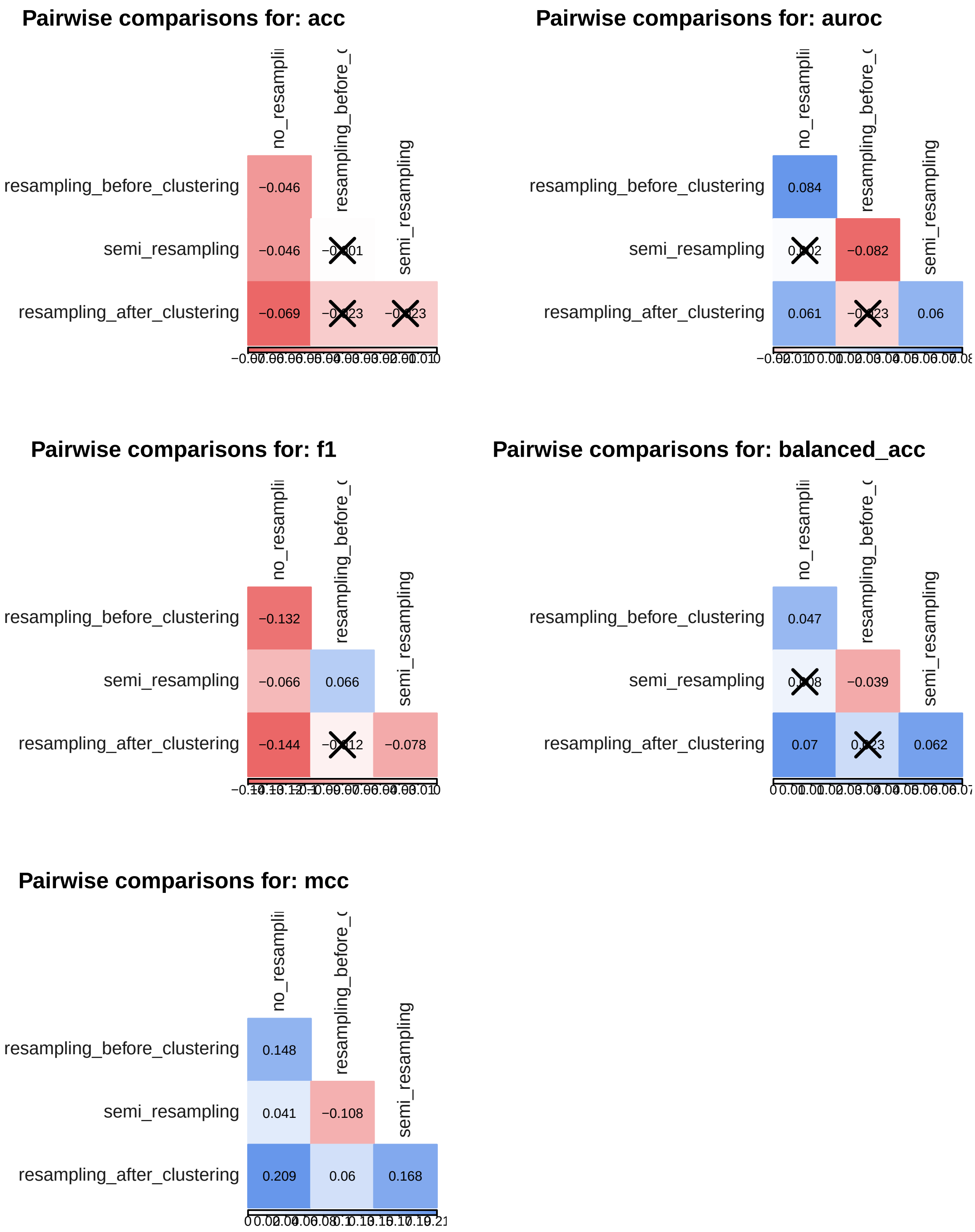

Figure S9: Pairwise comparison of strategy performance using Tukey method.

Table S14: ANOVA p-values for including the resampling strategy as a regressor in the performance models.

strategy	variable	Sum Sq	Df	F value	Pr(>F)
acc	strategy	5.170321	3	2.37e+01	2.89e-15
auroc	strategy	10.908150	3	4.78e+01	1.28e-30
f1	strategy	29.754722	3	1.09e+02	2.07e-69
balanced_acc	strategy	5.464791	3	2.72e+01	1.64e-17
mcc	strategy	48.559151	3	1.82e+02	7.97e-115

Table S15: Linear models to describe each performance metric. Standard deviations in parentheses.

	acc	auroc	f1	balanced_acc	mcc
	(1)	(2)	(3)	(4)	(5)
strategyresampling_before_clustering	−0.046***	0.084***	−0.132***	0.047***	0.148***
	(7.036e-03)	(8.279e-03)	(7.867e-03)	(6.749e-03)	(7.777e-03)
	p = 8.802e-11	p = 4.087e-24	p = 1.533e-62	p = 3.584e-12	p = 1.229e-79
strategysemi_resampling	−0.046***	1.799e-03	−0.066***	8.309e-03	0.041***
	(6.997e-03)	(8.241e-03)	(7.823e-03)	(6.712e-03)	(7.734e-03)
	p = 3.308e-11	p = 0.827	p = 3.896e-17	p = 0.216	p = 1.524e-07
strategyresampling_after_clustering	−0.069***	0.061***	−0.144***	0.07***	0.209***
	(0.011)	(0.012)	(0.013)	(0.011)	(0.012)
	p = 7.285e-10	p = 1.668e-07	p = 1.727e-30	p = 6.696e-11	p = 6.559e-63
log10(n_interactions)	0.098***	0.076***	0.123***	0.041***	0.114***
	(4.212e-03)	(4.958e-03)	(4.709e-03)	(4.040e-03)	(4.655e-03)
	p = 0.000e+00	p = 6.946e-52	p = 0.000e+00	p = 2.529e-24	p = 0.000e+00
log10(len_seq)	0.052**	0.024	0.053*	0.028*	0.043*
	(0.015)	(0.017)	(0.017)	(0.014)	(0.017)
	p = 5.105e-04	p = 0.157	p = 1.612e-03	p = 0.05	p = 9.744e-03
fold1	0.041*	−7.487e-03	0.036*	0.033*	−0.025
	(0.013)	(0.015)	(0.014)	(0.012)	(0.014)
	p = 1.037e-03	p = 0.617	p = 9.481e-03	p = 5.917e-03	p = 0.07
fold2	0.031*	−0.014	0.035*	0.018	−0.022
	(0.012)	(0.015)	(0.014)	(0.012)	(0.014)
	p = 0.014	p = 0.33	p = 0.012	p = 0.13	p = 0.103
fold3	0.057**	1.904e-03	0.073***	0.033*	1.973e-04
	(0.012)	(0.014)	(0.014)	(0.012)	(0.014)
	p = 5.280e-06	p = 0.896	p = 1.650e-07	p = 5.870e-03	p = 0.989
fold4	4.646e-03	−5.580e-03	0.023	−0.01	−7.461e-03
	(0.012)	(0.014)	(0.014)	(0.012)	(0.014)
	p = 0.707	p = 0.699	p = 0.097	p = 0.384	p = 0.585
fold5	0.035*	0.053**	0.081***	0.011	0.03*
	(0.012)	(0.014)	(0.014)	(0.012)	(0.014)
	p = 4.349e-03	p = 1.862e-04	p = 5.174e-09	p = 0.345	p = 0.03
fold6	0.016	−0.011	0.018	9.628e-03	6.239e-03
	(0.012)	(0.015)	(0.014)	(0.012)	(0.014)
	p = 0.208	p = 0.455	p = 0.182	p = 0.418	p = 0.648
fold7	0.027*	0.036*	0.066**	0.015	−2.417e-03
	(0.012)	(0.014)	(0.014)	(0.012)	(0.014)
	p = 0.031	p = 0.011	p = 1.775e-06	p = 0.198	p = 0.859
fold8	0.021	1.533e-03	0.056**	−1.289e-03	−0.024
	(0.012)	(0.014)	(0.014)	(0.012)	(0.014)
	p = 0.096	p = 0.915	p = 5.076e-05	p = 0.914	p = 0.085
fold9	0.025*	0.015	0.021	0.014	0.018
	(0.012)	(0.014)	(0.014)	(0.012)	(0.014)
	p = 0.045	p = 0.307	p = 0.134	p = 0.244	p = 0.193
Constant	0.274***	0.347***	0.243**	0.378***	−0.318***
	(0.045)	(0.051)	(0.05)	(0.043)	(0.049)
	p = 7.742e-10	p = 1.421e-11	p = 1.039e-06	p = 1.020e-18	p = 1.102e-10
Observations	9671	7501	9671	9671	9671
R^2	0.063	0.059	0.096	0.027	0.134
Adjusted R^2	0.062	0.057	0.095	0.026	0.133

Note: *p<0.05; **p<1.000e-03; ***p<1e-06

Table S16: Expected absolute performances, by metric and strategy, with 95% confidence intervals.

metric	strategy	emmean	SE	lower.CL	upper.CL
acc	no_resampling	0.712	5.614e-03	0.701	0.723
acc	resampling_before_clustering	0.666	5.618e-03	0.655	0.677
acc	semi_resampling	0.665	5.614e-03	0.654	0.676
acc	resampling_after_clustering	0.643	9.511e-03	0.624	0.662
auroc	no_resampling	0.627	6.280e-03	0.615	0.640
auroc	resampling_before_clustering	0.712	6.257e-03	0.699	0.724
auroc	semi_resampling	0.629	6.280e-03	0.617	0.641
auroc	resampling_after_clustering	0.689	9.737e-03	0.670	0.708
f1	no_resampling	0.766	6.277e-03	0.754	0.779
f1	resampling_before_clustering	0.634	6.282e-03	0.622	0.646
f1	semi_resampling	0.700	6.277e-03	0.688	0.713
f1	resampling_after_clustering	0.622	1.063e-02	0.601	0.643
balanced_acc	no_resampling	0.582	5.385e-03	0.572	0.593
balanced_acc	resampling_before_clustering	0.629	5.389e-03	0.619	0.640
balanced_acc	semi_resampling	0.591	5.385e-03	0.580	0.601
balanced_acc	resampling_after_clustering	0.652	9.123e-03	0.634	0.670
mcc	no_resampling	0.108	6.205e-03	0.096	0.120
mcc	resampling_before_clustering	0.256	6.210e-03	0.244	0.268
mcc	semi_resampling	0.149	6.205e-03	0.136	0.161
mcc	resampling_after_clustering	0.317	1.051e-02	0.296	0.337

Baseline-adjusted performance In this case, the strategy covariate was also always significant in a type 3 ANOVA (Table S17). The explanatory linear models for the adjusted metrics are summarized in S18.

Table S17: ANOVA p-values for including the resampling strategy as a regressor in the adjusted performance models.

strategy	variable	Sum Sq	Df	F value	Pr(>F)
acc	strategy	7.228605	3	2.09e+01	1.79e-13
auroc	strategy	12.123911	3	2.96e+01	4.81e-19
f1	strategy	3.665732	3	1.43e+01	2.78e-09
balanced_acc	strategy	15.297979	3	4.28e+01	1.82e-27
mcc	strategy	52.471976	3	7.77e+01	1.29e-49

Pairwise comparisons of the strategy coefficients using Tukey's method for the adjusted metrics are shown in Figure S10.

Expected adjusted performances by metric and strategy are in Table S19.

Table S18: Linear models to describe each adjusted performance metric. Standard deviations in parentheses.

	acc	auroc	f1	balanced_acc	mcc
	(1)	(2)	(3)	(4)	(5)
strategyresampling_before_clustering	0.07***	0.087***	0.05***	0.093***	0.157***
	(8.861e-03)	(0.011)	(7.628e-03)	(9.002e-03)	(0.012)
	p = 3.431e-15	p = 3.742e-15	p = 6.971e-11	p = 1.129e-24	p = 1.213e-36
strategysemi_resampling	0.037**	−2.411e-03	0.026**	0.029*	0.041*
	(8.966e-03)	(0.011)	(7.719e-03)	(9.108e-03)	(0.013)
	p = 4.267e-05	p = 0.829	p = 7.937e-04	p = 1.332e-03	p = 1.190e-03
strategyresampling_after_clustering	0.043*	0.06**	0.03*	0.098***	0.209***
	(0.014)	(0.016)	(0.012)	(0.014)	(0.02)
	p = 2.204e-03	p = 1.412e-04	p = 0.013	p = 9.770e-12	p = 5.834e-26
log10(n_interactions)	0.053***	0.079***	4.567e-03	0.026**	0.118***
	(5.390e-03)	(6.740e-03)	(4.640e-03)	(5.475e-03)	(7.529e-03)
	p = 2.346e-22	p = 3.209e-31	p = 0.325	p = 2.408e-06	p = 9.075e-55
log10(len_seq)	0.046*	0.018	0.021	0.02	0.025
	(0.019)	(0.023)	(0.016)	(0.019)	(0.027)
	p = 0.016	p = 0.439	p = 0.2	p = 0.294	p = 0.35
fold1	0.029	−6.058e-03	7.826e-03	0.021	−0.023
	(0.016)	(0.02)	(0.014)	(0.016)	(0.022)
	p = 0.071	p = 0.763	p = 0.567	p = 0.183	p = 0.296
fold2	0.024	−0.026	−8.380e-03	0.014	−0.044*
	(0.016)	(0.02)	(0.014)	(0.016)	(0.022)
	p = 0.126	p = 0.193	p = 0.539	p = 0.388	p = 0.046
fold3	0.049*	−3.319e-03	0.027*	0.029	−0.022
	(0.016)	(0.02)	(0.014)	(0.016)	(0.022)
	p = 1.775e-03	p = 0.865	p = 0.046	p = 0.073	p = 0.317
fold4	9.957e-03	1.882e-03	0.013	−7.284e-04	3.653e-03
	(0.016)	(0.019)	(0.014)	(0.016)	(0.022)
	p = 0.527	p = 0.923	p = 0.343	p = 0.964	p = 0.868
fold5	0.024	0.057*	0.024	9.004e-03	0.025
	(0.016)	(0.019)	(0.014)	(0.016)	(0.022)
	p = 0.134	p = 3.174e-03	p = 0.079	p = 0.575	p = 0.255
fold6	2.762e-03	−0.023	3.872e-03	7.173e-04	−6.218e-03
	(0.016)	(0.02)	(0.014)	(0.016)	(0.022)
	p = 0.861	p = 0.248	p = 0.775	p = 0.964	p = 0.777
fold7	0.022	0.032	0.033*	0.013	−0.011
	(0.016)	(0.019)	(0.013)	(0.016)	(0.022)
	p = 0.159	p = 0.099	p = 0.015	p = 0.406	p = 0.601
fold8	7.448e-03	−3.576e-04	5.280e-03	−0.012	−0.033
	(0.016)	(0.019)	(0.014)	(0.016)	(0.022)
	p = 0.636	p = 0.985	p = 0.697	p = 0.457	p = 0.132
fold9	0.019	7.796e-03	−9.248e-03	9.900e-03	−2.422e-03
	(0.016)	(0.019)	(0.013)	(0.016)	(0.022)
	p = 0.216	p = 0.684	p = 0.493	p = 0.534	p = 0.912
Constant	−0.184*	−0.141*	0.03	−0.08	−0.273**
	(0.057)	(0.069)	(0.049)	(0.057)	(0.079)
	p = 1.143e-03	p = 0.041	p = 0.534	p = 0.166	p = 5.493e-04
Observations	9473	7387	9473	9473	9473
R^2	0.02	0.036	7.025e-03	0.019	0.061
Adjusted R^2	0.019	0.035	5.555e-03	0.017	0.06

Note: *p<0.05; **p<1.000e-03; ***p<1e-06

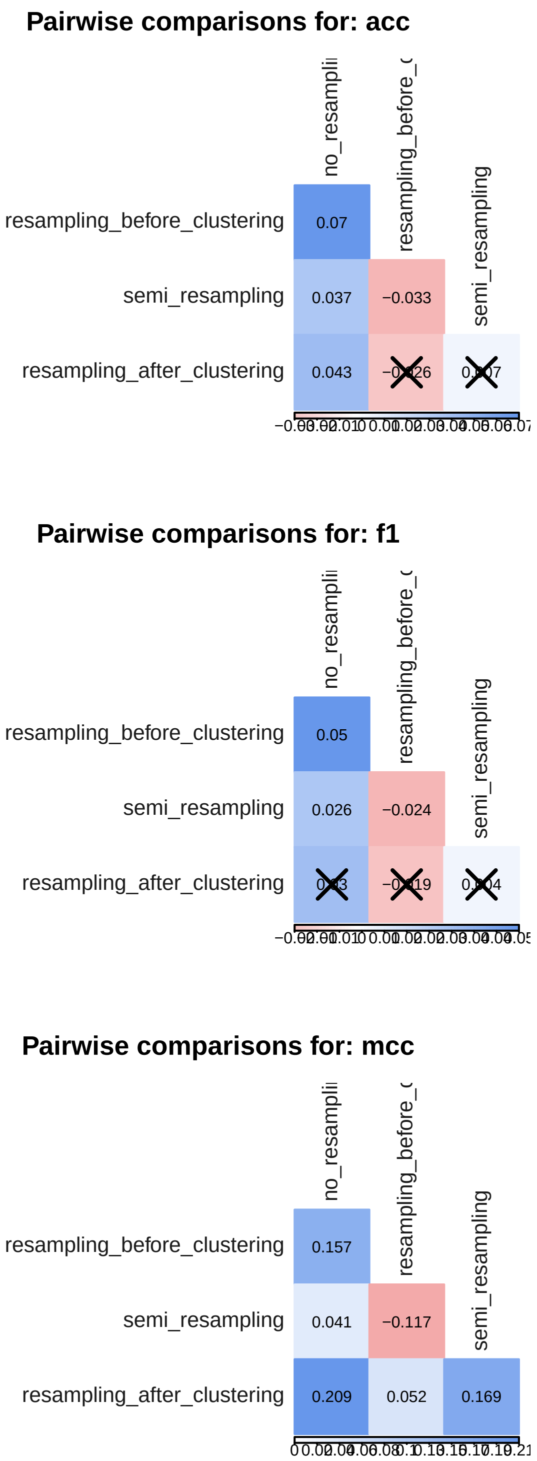

Figure S10: Pairwise comparison of strategy adjusted performance using Tukey method.

Table S19: Expected adjusted performances, by metric and strategy, with 95% confidence intervals.

metric	strategy	emmean	SE	lower.CL	upper.CL
acc	no_resampling	0.108	7.116e-03	0.094	0.122
acc	resampling_before_clustering	0.177	7.119e-03	0.164	0.191
acc	semi_resampling	0.144	7.180e-03	0.130	0.158
acc	resampling_after_clustering	0.151	1.204e-02	0.127	0.175
auroc	no_resampling	0.127	8.448e-03	0.110	0.144
auroc	resampling_before_clustering	0.214	8.413e-03	0.198	0.231
auroc	semi_resampling	0.125	8.528e-03	0.108	0.141
auroc	resampling_after_clustering	0.187	1.311e-02	0.161	0.213
f1	no_resampling	0.112	6.126e-03	0.100	0.124
f1	resampling_before_clustering	0.162	6.128e-03	0.150	0.174
f1	semi_resampling	0.138	6.181e-03	0.126	0.150
f1	resampling_after_clustering	0.143	1.036e-02	0.122	0.163
balanced_acc	no_resampling	0.057	7.229e-03	0.043	0.071
balanced_acc	resampling_before_clustering	0.149	7.232e-03	0.135	0.163
balanced_acc	semi_resampling	0.086	7.294e-03	0.072	0.100
balanced_acc	resampling_after_clustering	0.155	1.223e-02	0.131	0.179
mcc	no_resampling	0.106	9.940e-03	0.086	0.125
mcc	resampling_before_clustering	0.263	9.944e-03	0.243	0.282
mcc	semi_resampling	0.146	1.003e-02	0.127	0.166
mcc	resampling_after_clustering	0.315	1.682e-02	0.282	0.348

Further validation for other protein families

Table S20 shows the correlations between training and sets sets active ratios for GPCRs.

Table S20: Correlations between train and test active ratios. 95% confidence intervals and p-values are shown.

strategy	cor	ci_lower	ci_upper	alternative	pvalue
no_resampling	0.594	0.572	0.616	two.sided	6.73e-320
resampling_before_clustering	-0.386	-0.417	-0.354	two.sided	4.80e-98
semi_resampling	0.067	0.029	0.105	two.sided	5.91e-04
resampling_after_clustering	NA	NA	NA	two.sided	NA

Table S21 shows the linear models describing the predicted active ratio for the semi_resampling and the resampling_after_clustering strategies.

Table S21: Linear models to describe the predicted active ratio for the semi_resampling and the resampling_before_clustering strategies. Significance and 95% confidence intervals are included.

	semi_resampling	resampling_before_clustering
	(1)	(2)
ratio_training	−0.023 (−0.252, 0.206)<br>p = 0.843	0.295 (0.142, 0.447)**<br>p = 1.544e-04
ratio_test	0.252 (0.213, 0.29)***<br>p = 5.436e-36	0.346 (0.305, 0.388)***<br>p = 7.190e-58
log10(n_interactions)	−0.011 (−0.032, 0.01)<br>p = 0.302	−0.024 (−0.043, −4.597e-03)*<br>p = 0.015
log10(len_seq)	−0.137 (−0.288, 0.014)<br>p = 0.076	−0.17 (−0.316, −0.025)*<br>p = 0.021
fold1	0.017 (−0.032, 0.066)<br>p = 0.497	−0.123 (−0.17, −0.076)***<br>p = 2.947e-07
fold2	−0.019 (−0.068, 0.031)<br>p = 0.458	0.052 (5.878e-03, 0.099)*<br>p = 0.027
fold3	−0.033 (−0.082, 0.017)<br>p = 0.202	0.038 (−8.623e-03, 0.085)<br>p = 0.11
fold4	−0.01 (−0.06, 0.039)<br>p = 0.68	0.017 (−0.03, 0.064)<br>p = 0.469
fold5	0.035 (−0.014, 0.084)<br>p = 0.158	−0.085 (−0.132, −0.037)**<br>p = 5.370e-04
fold6	−0.055 (−0.105, −6.206e-03)*<br>p = 0.027	−0.025 (−0.073, 0.022)<br>p = 0.295
fold7	−0.171 (−0.219, −0.122)***<br>p = 8.958e-12	1.423e-04 (−0.046, 0.047)<br>p = 0.995
fold8	−0.2 (−0.249, −0.151)***<br>p = 2.301e-15	−0.038 (−0.085, 9.298e-03)<br>p = 0.116
fold9	−0.148 (−0.197, −0.099)***<br>p = 4.477e-09	0.038 (−9.078e-03, 0.084)<br>p = 0.115
Constant	0.907 (0.495, 1.318)**<br>p = 1.665e-05	0.724 (0.338, 1.11)**<br>p = 2.446e-04
Observations	2620	2741
R^2	0.123	0.12
Adjusted R^2	0.118	0.116
Residual Std. Error	0.287 (df = 2606)	0.28 (df = 2727)
F Statistic	28.011*** (df = 13; 2606)	28.722*** (df = 13; 2727)
Note:		*p<0.05; **p<1.000e-03; ***p<1e-06

Table S22 summarizes the linear models describing each adjusted performance metric.

Table S22: Linear models to describe each adjusted performance metric. Standard deviations in parentheses.

	acc	auroc	f1	balanced_acc	mcc
	(1)	(2)	(3)	(4)	(5)
strategyresampling_before_clustering	0.111***	0.114***	0.063***	0.148***	0.202***
	(6.797e-03)	(8.306e-03)	(6.172e-03)	(6.726e-03)	(8.543e-03)
	p = 4.459e-59	p = 1.574e-42	p = 1.135e-24	p = 0.000e+00	p = 0.000e+00
strategysemi_resampling	0.053***	0.011	0.015*	0.068***	0.061***
	(6.905e-03)	(8.479e-03)	(6.270e-03)	(6.833e-03)	(8.678e-03)
	p = 1.885e-14	p = 0.188	p = 0.019	p = 5.502e-23	p = 1.966e-12
strategyresampling_after_clustering	0.057***	0.098***	0.042***	0.12***	0.235***
	(9.236e-03)	(0.01)	(8.387e-03)	(9.140e-03)	(0.012)
	p = 6.266e-10	p = 2.754e-21	p = 6.864e-07	p = 3.564e-39	p = 1.186e-89
log10(n_interactions)	0.04***	0.038***	0.014**	0.014*	0.052***
	(4.375e-03)	(6.018e-03)	(3.972e-03)	(4.329e-03)	(5.498e-03)
	p = 5.057e-20	p = 1.732e-10	p = 3.523e-04	p = 1.485e-03	p = 2.617e-21
log10(len_seq)	−0.091*	−0.034	−1.940e-03	−0.093*	−0.027
	(0.035)	(0.042)	(0.031)	(0.034)	(0.043)
	p = 8.579e-03	p = 0.419	p = 0.951	p = 6.524e-03	p = 0.541
fold1	0.024*	2.277e-03	−0.011	0.014	0.012
	(0.012)	(0.014)	(0.011)	(0.011)	(0.015)
	p = 0.036	p = 0.869	p = 0.297	p = 0.215	p = 0.43
fold2	7.466e-03	0.016	9.307e-03	5.819e-03	0.012
	(0.012)	(0.014)	(0.011)	(0.011)	(0.015)
	p = 0.52	p = 0.26	p = 0.377	p = 0.612	p = 0.401
fold3	0.012	−6.461e-03	−6.538e-03	8.988e-03	−3.534e-03
	(0.012)	(0.014)	(0.011)	(0.012)	(0.015)
	p = 0.322	p = 0.648	p = 0.541	p = 0.441	p = 0.811
fold4	0.015	−8.950e-03	8.932e-03	0.014	3.821e-03
	(0.012)	(0.014)	(0.011)	(0.012)	(0.015)
	p = 0.2	p = 0.53	p = 0.404	p = 0.244	p = 0.797
fold5	6.087e-03	−0.012	−0.018	4.073e-03	−0.012
	(0.012)	(0.014)	(0.011)	(0.012)	(0.015)
	p = 0.604	p = 0.398	p = 0.087	p = 0.726	p = 0.426
fold6	0.017	3.990e-04	−0.017	0.014	0.011
	(0.012)	(0.014)	(0.011)	(0.012)	(0.015)
	p = 0.15	p = 0.977	p = 0.108	p = 0.239	p = 0.474
fold7	9.271e-04	3.367e-03	−0.026*	8.568e-03	0.01
	(0.012)	(0.014)	(0.011)	(0.012)	(0.015)
	p = 0.936	p = 0.809	p = 0.015	p = 0.456	p = 0.484
fold8	0.016	0.011	−0.029*	0.017	0.018
	(0.012)	(0.014)	(0.011)	(0.012)	(0.015)
	p = 0.176	p = 0.424	p = 6.887e-03	p = 0.131	p = 0.213
fold9	7.169e-03	0.011	−0.016	9.026e-03	0.011
	(0.012)	(0.014)	(0.011)	(0.012)	(0.015)
	p = 0.538	p = 0.435	p = 0.131	p = 0.433	p = 0.47
Constant	0.184*	0.077	0.049	0.205*	−0.041
	(0.09)	(0.111)	(0.082)	(0.089)	(0.114)
	p = 0.041	p = 0.486	p = 0.552	p = 0.022	p = 0.719
Observations	9902	7958	9902	9902	9902
R^2	0.042	0.04	0.017	0.056	0.096
Adjusted R^2	0.04	0.039	0.016	0.055	0.095

Note: *p<0.05; **p<1.000e-03; ***p<1e-06

References

(1) Lecun, Y.; Bengio, Y.; Hinton, G. Deep learning. *Nature* **2015**, *521*, 436–444.

Appendix 2: model predictions and performance (kinases)

Angela Lopez-del Rio Sergio Picart-Armada Alexandre Perera-Lluna

05/12/2020

Contents

1 Overview 1

2 Description of data balance 2
2.1 Distributions of the actives ratio . . . 2
2.2 Comparing training and test imbalance . . . 2
2.3 Other covariates . . . 2

3 Linear models on predicted proportions 5
3.1 Distributions of the predicted ratios . . . 5
3.2 Predicted ratios against training ratios . . . 6
3.3 Linear models . . . 6
3.4 Conclusions . . . 8

4 Description of baseline performance 11
4.1 Descriptive plot . . . 11
4.2 Linear models . . . 11

5 Description of deep learning model performance 12
5.1 Absolute, baseline-naive performance . . . 12
5.2 Baseline-adjusted performance . . . 18

6 Reproducibility 23

1 Overview

This supplement describes the behaviour of the proteochemometrics (PCM) deep learning model to predict protein-compound bioactivity. Specifically, this includes the descriptive statistics of data imbalance: the proportion of actives per protein in the training and test sets during the model fitting and the predicted proportion of actives. The model performance per protein was also described, pinpointing the most influential factors and characterising the proteins with the most extreme performances.

Four strategies (no_resampling, resampling_before_clustering, semi_resampling, resampling_after_clustering) were considered. For each of those, 10 folds of repeated holdout were run, and 5 performance metrics were computed: acc, auroc, f1, balanced_acc, mcc. This led to a total of 9671 values of performance. Since some strategies involved the upsampling method SMOTE, proteins whose sample sizes did not allow upsampling were excluded (table 1).

Table 1: Number of proteins for which performance metrics were computed. The resampling after clustering was the most stringent strategy regarding eligible proteins, since the resampling was carried out after the clustering, which introduced more imbalance.

Strategy	Fold 0	Fold 1	Fold 2	Fold 3	Fold 4	Fold 5	Fold 6	Fold 7	Fold 8	Fold 9
no_resampling	288	282	295	303	305	305	293	307	294	301
resampling_before_clustering	271	295	284	274	298	286	301	307	302	293
semi_resampling	288	282	295	303	305	305	293	307	294	301
resampling_after_clustering	79	74	72	84	76	79	87	82	81	100

2 Description of data balance

The data balancing strategy had an impact on the actual data balance, defined as the proportion of active molecules for a protein. Furthermore, specific trends were observed in the original data in the training and test sets, as well as in the values predicted by the deep learning model.

2.1 Distributions of the actives ratio

The histograms in figure 1 revealed trends:

- `no_resampling` keeps similar data imbalance in training and test.
- `resampling_before_clustering` and `semi_resampling` lead to a more balanced training set, but not so much for the test set.
- `resampling_after_clustering` kept balanced proteins in both training and test sets.

In addition, test sets with imbalance tended to magnify it and create extreme cases (all actives or all inactives), probably due to the combination of the clustering and the lower sample sizes in the test sets compared to training.

2.2 Comparing training and test imbalance

Figure 2 revealed both positive, negative and null trends between the training and test set protein balances.

- `no_resampling` showed a positive relation between both, i.e. proteins were prone to keep their (im)balance in train and test.
- `resampling_before_clustering` showed an inverse relationship instead. This was expected since this strategy started from globally balanced proteins, and after the clustering, an imbalance in one direction in the training set entailed an inverse imbalance in the test set.
- `semi_resampling` led to independent train and test balances, expected since the train set was resampled, breaking any correlation with the test set balance.
- `resampling_after_clustering` always kept balanced proteins, by design.

Table 2 displays the Pearson correlation estimate, 95% confidence interval and p-value for each strategy (except `resampling_after_clustering`, where ratios are constant), further confirming the claims above.

2.3 Other covariates

The effect of the number of interactions of each protein in its corresponding set and fold (figure 3) and the protein length in amino acids (figure 4) on the test set imbalance was investigated:

- Proteins with greatest imbalance (i.e. where `(0.5 - ratio_test)^2` was greatest) tended to be among those with the least interactions. Linear correlations were significant (table 3).

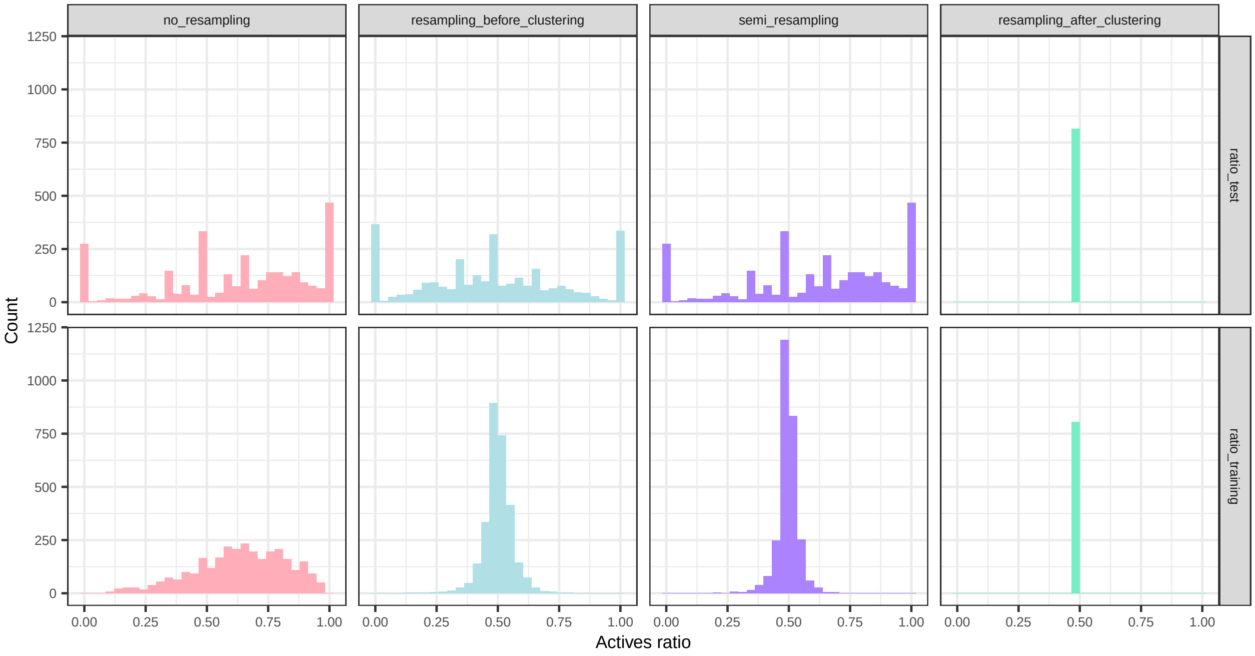

Figure 1: Distributios of the actives ratio in the training set and in the test set (both original and predicted by the deep learning model).

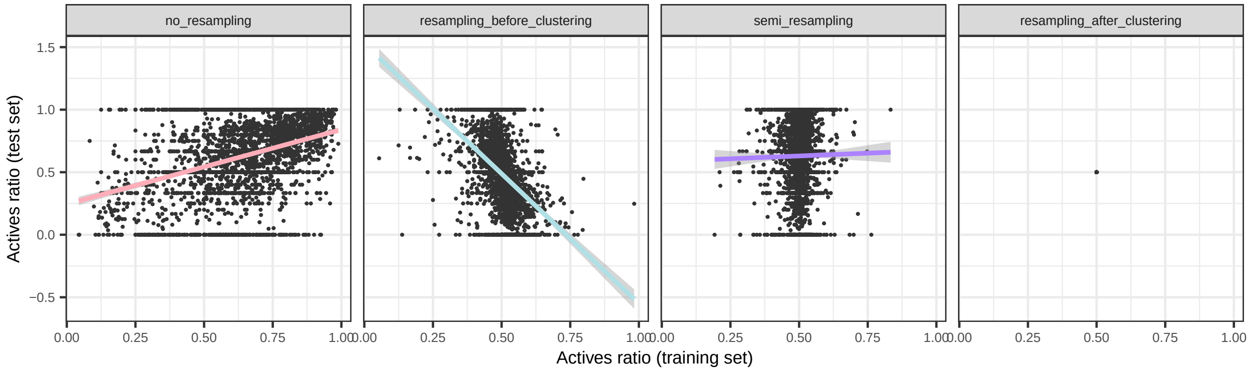

Figure 2: Comparison of the training and test ratios, by resampling strategy. A linear fit line was added per strategy.

Table 2: Correlations between train and test active ratios. 95% confidence intervals and p-values are shown.

strategy	cor	ci_lower	ci_upper	alternative	pvalue
no_resampling	0.369	0.338	0.400	two.sided	1.15e-96
resampling_before_clustering	-0.428	-0.457	-0.398	two.sided	5.54e-130
semi_resampling	0.014	-0.024	0.051	two.sided	4.76e-01
resampling_after_clustering	NA	NA	NA	two.sided	NA

- The sequence length had no obvious effect on the protein imbalance. Linear correlations were not significant (`no_resampling`, `semi_resampling`) or significant but low (`resampling_before_clustering`).

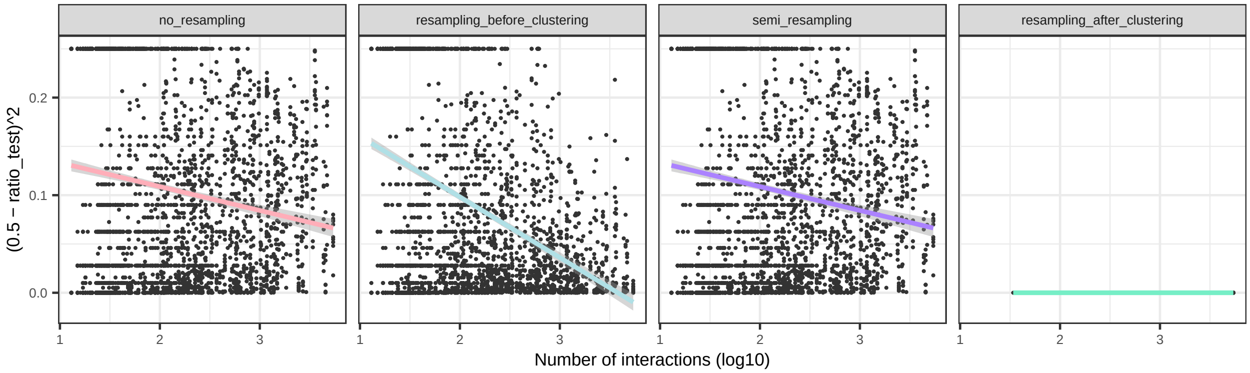

Figure 3: Data imbalance in the test set as a function of the number of available interactions for each protein.

Table 3: Correlations between imbalance (as defined above) and number of interactions. 95% confidence intervals and p-values are shown.

strategy	cor	ci_lower	ci_upper	alternative	pvalue
no_resampling	-0.061	-0.097	-0.026	two.sided	8.01e-04
resampling_before_clustering	-0.274	-0.307	-0.240	two.sided	3.98e-51
semi_resampling	-0.061	-0.097	-0.026	two.sided	8.01e-04
resampling_after_clustering	NA	NA	NA	two.sided	NA

Table 4: Correlations between imbalance (as defined above) and sequence length. 95% confidence intervals and p-values are shown.

strategy	cor	ci_lower	ci_upper	alternative	pvalue
no_resampling	-0.016	-0.052	0.020	two.sided	3.73e-01
resampling_before_clustering	-0.046	-0.082	-0.009	two.sided	1.37e-02
semi_resampling	-0.016	-0.052	0.020	two.sided	3.73e-01
resampling_after_clustering	NA	NA	NA	two.sided	NA

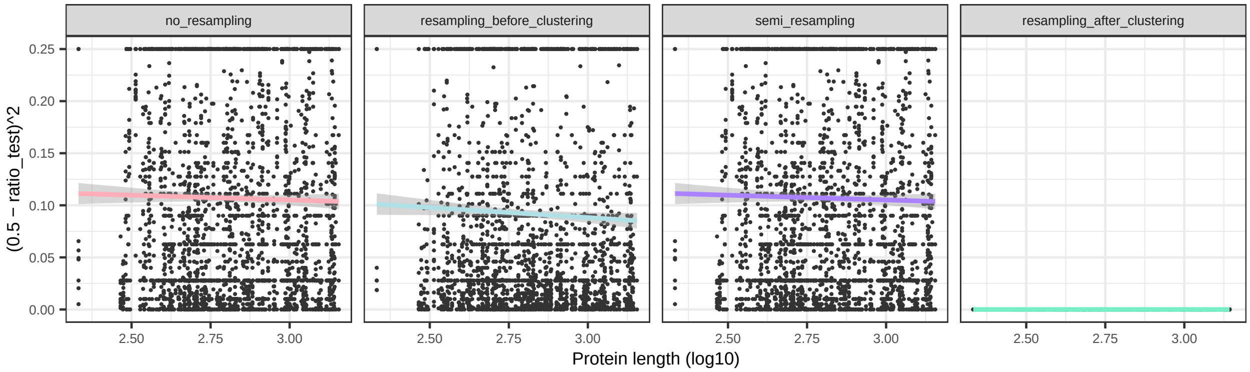

Figure 4: Data imbalance in the test set as a function of the protein length, in amino acids.

3 Linear models on predicted proportions

The next key question was to narrow down the factor driving the predicted proportion of actives. The main options under consideration were:

1. A constant, global imbalance that the model would learn from the whole dataset.
2. The protein-wise imbalance that the model would learn in the training set.
3. A test set-driven imbalance, based on its actual imbalance.

3.1 Distributions of the predicted ratios

After the model predictions in the test set were binarized (actives were those whose probabilities exceeded 0.5), the ratio of predicted actives was computed by protein. This ratio, shown in figure 5, suggested that:

- `no_resampling` was noticeably biased to predict everything as positives.
- `resampling_before_clustering` and `semi_resampling` alleviated the imbalance in the predictions, but still retained a spike of proteins where all the compounds were predicted as positives.
- `resampling_after_clustering` kept a wide and symmetric distribution of predicted actives.

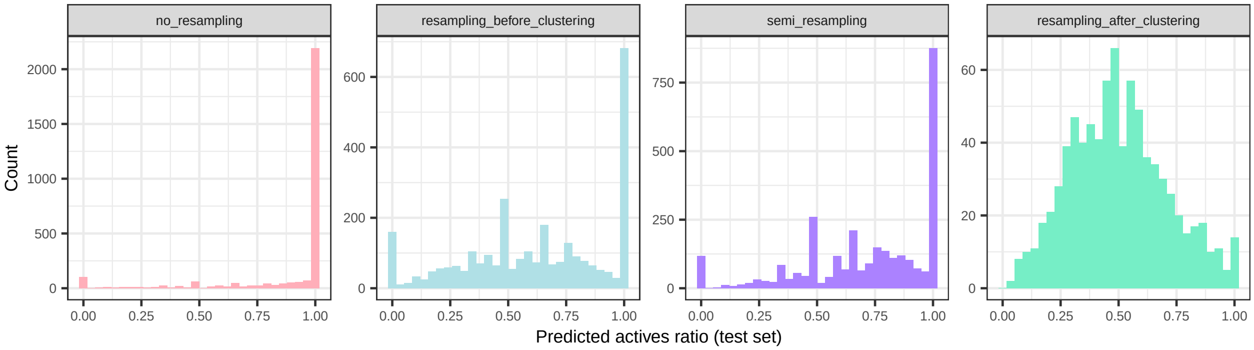

Figure 5: Ratios of the prediction values, after binarization.

Now, representing together (1) the original training and test ratios, and (2) the predicted ratios in test (figure 6) eased a general qualitative assessment: the distribution was most resemblant to that of the test proportions to that of the training ones (except `resampling_after_clustering`, since those proportions are constant). Table 5 displays how `no_resampling` was highly inclined to predict all positives, `resampling_before_clustering`

and `semi_resampling` alleviated this phenomenon, and `resampling_after_clustering` was essentially balanced.

Table 5: Percentage of extreme cases, i.e. proteins with all actives or inactives.

strategy	RatioSet	all_inactives	all_actives	all_extremes
no_resampling	ratio_test	9.2	15.5	24.7
no_resampling	ratio_test_predicted	3.5	71.6	75.1
no_resampling	ratio_training	0.0	0.0	0.0
resampling_before_clustering	ratio_test	12.5	11.5	24.0
resampling_before_clustering	ratio_test_predicted	5.5	23.4	28.9
resampling_before_clustering	ratio_training	0.0	0.0	0.0
semi_resampling	ratio_test	9.2	15.5	24.7
semi_resampling	ratio_test_predicted	4.0	29.1	33.1
semi_resampling	ratio_training	0.0	0.0	0.0
resampling_after_clustering	ratio_test	0.0	0.0	0.0
resampling_after_clustering	ratio_test_predicted	0.0	1.2	1.2
resampling_after_clustering	ratio_training	0.0	0.0	0.0

3.2 Predicted ratios against training ratios

Figure 7 puts the predicted ratios in context of the training ratios, elucidating a variety of trends:

- `no_resampling`: positive trend between the training and the predicted ratio, but since the training and the test ratio also positively correlated (figure 2), the latter could be the one driving the predicted ratio of positives.
- `resampling_after_clustering` had a constant training ratio, meaning that the predicted ratio was not explainable by differences in training ratios.
- `resampling_before_clustering` showed instead a negative relation between the training and the predicted ratio. But since the former and the test ratio also anticorrelated (figure 2, the simplest explanation was that the test ratio drove the predicted test ratio.
- `semi_resampling` showed no apparent correlation between the predicted ratio and the training ratio.

The significance of the linear correlation backs up all the claims above (table 6).

Table 6: Correlations between train and predicted test active ratios. 95% confidence intervals and p-values are shown.

strategy	cor	ci_lower	ci_upper	alternative	pvalue
no_resampling	0.469	0.440	0.496	two.sided	2.79e-162
resampling_before_clustering	-0.094	-0.130	-0.058	two.sided	3.77e-07
semi_resampling	0.008	-0.029	0.045	two.sided	6.78e-01
resampling_after_clustering	NA	NA	NA	two.sided	NA

3.3 Linear models

The predicted ratio of actives r_{pred} was modelled through the following quasibinomial generalized linear models, stratified by strategy:

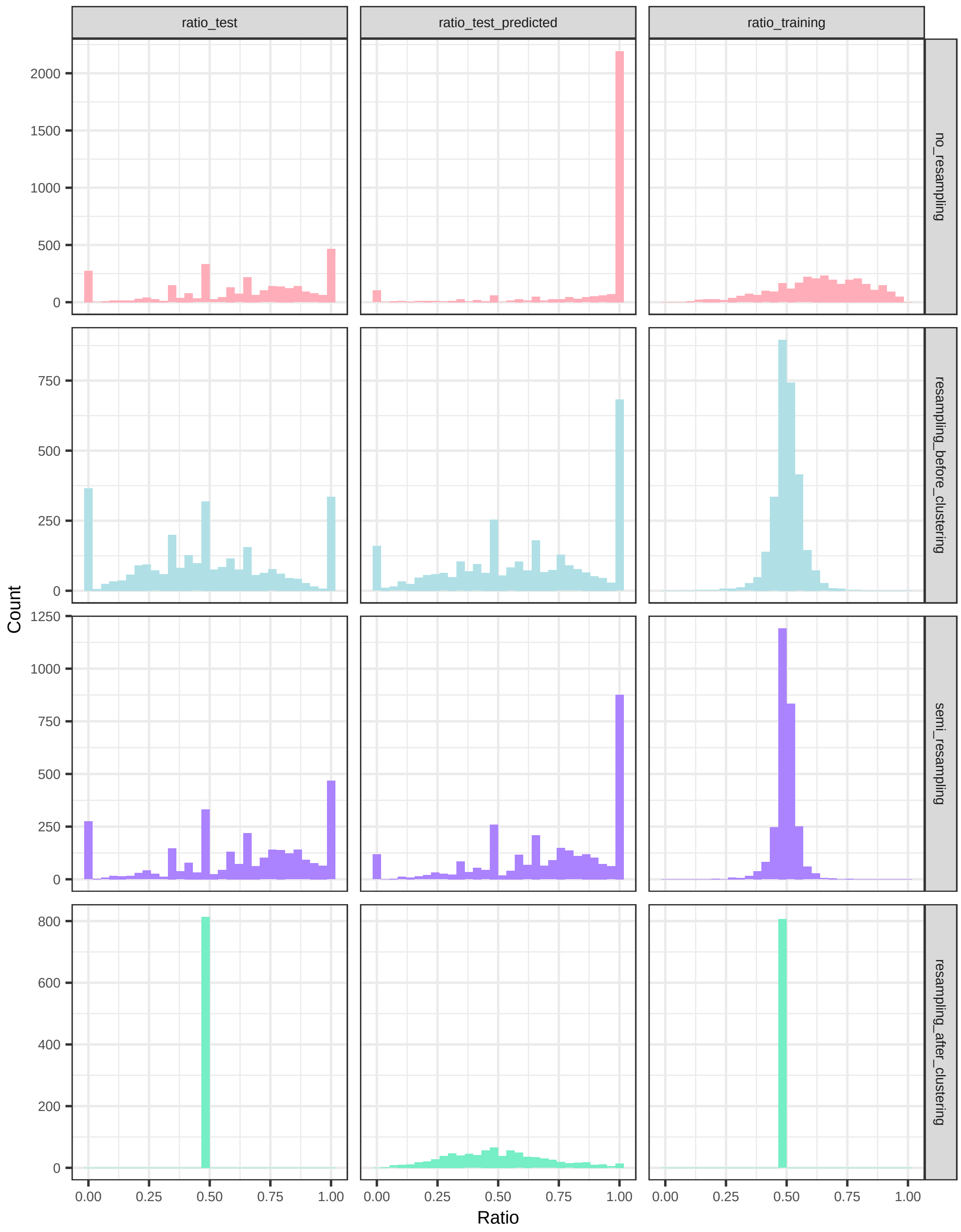

Figure 6: Distributios of the actives ratio in the training set and in the test set (both original and predicted by the deep learning model).

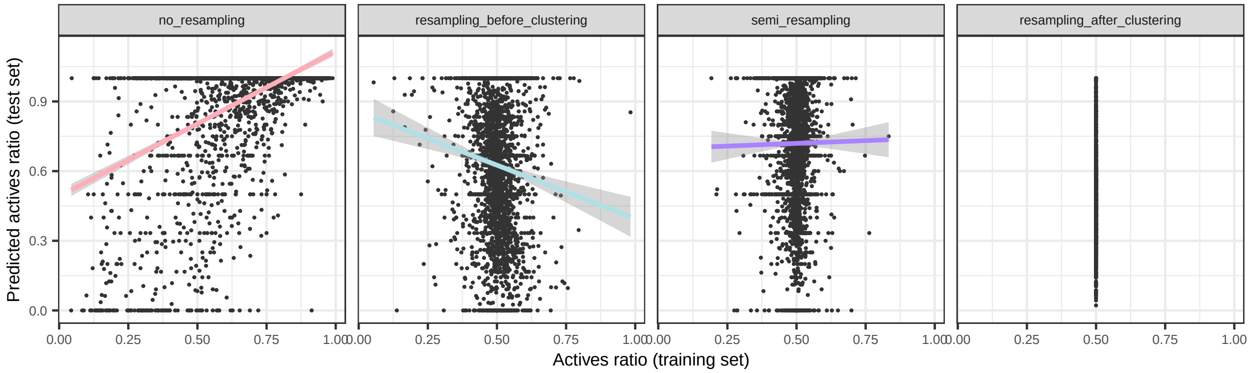

Figure 7: Predicted ratios, as a function of training ratios.

$$r_{pred} \sim r_{training} + r_{test} + \log10(n_{int}) + \log10(n_{seq}) + k_{fold}$$

The main variables of interest are the actual ratios in the training $r_{training}$ and in test r_{test}, both numeric between 0 and 1. As additional covariates, the number of interactions n_{int} and the sequence length n_{seq} (numerical) and the fold number k_{fold} (categorical) were also included.

3.3.1 In semi_resampling or resampling_before_clustering

Due to the lack of correlation between training and test ratios (figure 2), the `semi_resampling` strategy is the **ideal scenario** to disentangle their effects on the predicted ratio of actives (see model in table 7). This additive model suggests:

- The **test_ratio is driving the predicted proportions**, rather than the training ratio.
- A relevant factor is **n_interactions**: the more interactions, the less active proportion, suggesting that the extreme cases with all predicted as actives tend to be proteins with few interactions.

Table 7 also shows the additive model for `resampling_before_clustering`. This strategy showed negative correlation between training and test ratios, also providing a reasonably good scenario to distinguish their effects.

- This model **confirms both conclusions** from the model in the `semi_resampling` strategy, with similar estimates.

3.3.2 In no_resampling

The explanatory linear model under the `no_resampling` strategy (table 8) suffers from the positive correlation between training and test ratios, which can be confounded.

- Both `training_ratio` and `test_ratio` show a positive effect on the predicted fraction of actives.
- Although the estimate is larger and more significant for `training_ratio`, the confounding effect and the very skewed distribution of the predicted ratios deems this model inconclusive.

3.4 Conclusions

- Data imbalance exists in all strategies but in `resampling_after_clustering`, where balance is enforced.
- The correlation between a protein's ratio in train and test is positive in `no_resampling`, negative in `resampling_before_clustering` and null in `semi_resampling` and `resampling_after_clustering`.

Table 7: Linear models to describe the predicted active ratio for the semi_resampling and the resampling_before_clustering strategies. Significance and 95% confidence intervals are included.

	semi_resampling	resampling_before_clustering
	(1)	(2)
ratio_training	0.197 (−0.903, 1.298)	−0.446 (−1.296, 0.405)
	p = 0.725	p = 0.305
ratio_test	0.945 (0.775, 1.114)***	0.784 (0.606, 0.963)***
	p = 2.460e-27	p = 1.181e-17
log10(n_interactions)	−0.391 (−0.467, −0.314)***	−0.396 (−0.466, −0.325)***
	p = 3.197e-23	p = 1.987e-27
log10(len_seq)	0.289 (0.023, 0.554)*	−0.033 (−0.293, 0.226)
	p = 0.033	p = 0.801
fold1	0.034 (−0.176, 0.245)	0.071 (−0.138, 0.281)
	p = 0.748	p = 0.504
fold2	−0.651 (−0.852, −0.45)***	0.416 (0.196, 0.635)**
	p = 2.348e-10	p = 2.076e-04
fold3	0.982 (0.74, 1.224)***	−0.436 (−0.646, −0.226)**
	p = 2.598e-15	p = 4.738e-05
fold4	0.665 (0.44, 0.891)***	0.326 (0.114, 0.538)*
	p = 8.708e-09	p = 2.609e-03
fold5	0.023 (−0.187, 0.232)	0.333 (0.118, 0.548)*
	p = 0.831	p = 2.413e-03
fold6	−0.524 (−0.725, −0.323)***	0.021 (−0.186, 0.229)
	p = 3.457e-07	p = 0.839
fold7	0.626 (0.402, 0.849)***	0.377 (0.165, 0.589)**
	p = 4.506e-08	p = 5.045e-04
fold8	0.504 (0.283, 0.725)**	0.378 (0.165, 0.592)**
	p = 8.130e-06	p = 5.250e-04
fold9	−8.292e-03 (−0.215, 0.199)	−0.73 (−0.938, −0.522)***
	p = 0.937	p = 7.301e-12
Constant	0.177 (−0.76, 1.113)	1.229 (0.34, 2.118)*
	p = 0.712	p = 6.796e-03
Observations	2783	2911
Note:		*p<0.05; **p<1.000e-03; ***p<1e-06

Table 8: Linear models to describe the predicted active ratio for the no_resampling strategy. Significance and 95% confidence intervals are included.

	no_resampling
ratio_training	8.312 (7.581, 9.042)***
	p = 0.000e+00
ratio_test	1.102 (0.741, 1.464)***
	p = 2.600e-09
log10(n_interactions)	−1.24 (−1.422, −1.058)***
	p = 1.330e-39
log10(len_seq)	−0.949 (−1.513, −0.386)**
	p = 9.683e-04
fold1	−0.505 (−0.931, −0.078)*
	p = 0.021
fold2	−0.034 (−0.478, 0.411)
	p = 0.881
fold3	1.063 (0.545, 1.58)**
	p = 5.828e-05
fold4	−0.805 (−1.21, −0.4)**
	p = 9.970e-05
fold5	0.917 (0.411, 1.423)**
	p = 3.862e-04
fold6	1.709 (1.097, 2.321)***
	p = 4.711e-08
fold7	0.169 (−0.278, 0.615)
	p = 0.459
fold8	0.116 (−0.341, 0.574)
	p = 0.618
fold9	0.886 (0.385, 1.387)**
	p = 5.365e-04
Constant	1.904 (0.269, 3.539)*
	p = 0.023
Observations	2973
Note:	*p<0.05; **p<1.000e-03; ***p<1e-06

- The main factor driving the ratio of actives in the model predictions, per protein, is the actual ratio of positives in the test set. Their distributions are resemblant, and linear models confirm the association.

4 Description of baseline performance

Before evaluating the deep learning model, the performance metrics of the baselines were characterised, in order to pinpoint imbalance-sensitive and insensitive metrics. Metrics were called imbalance-sensitive if the imbalance-aware random baseline exhibited different performances between resampling strategies.

4.1 Descriptive plot

Figure 8 shows a fold-averaged picture of the metrics by protein. Visual inspection suggested that accuracy, F1 and possibly balanced accuracy were affected by the data imbalance. F1 is the most apparent case, see the quartiles in table 9.

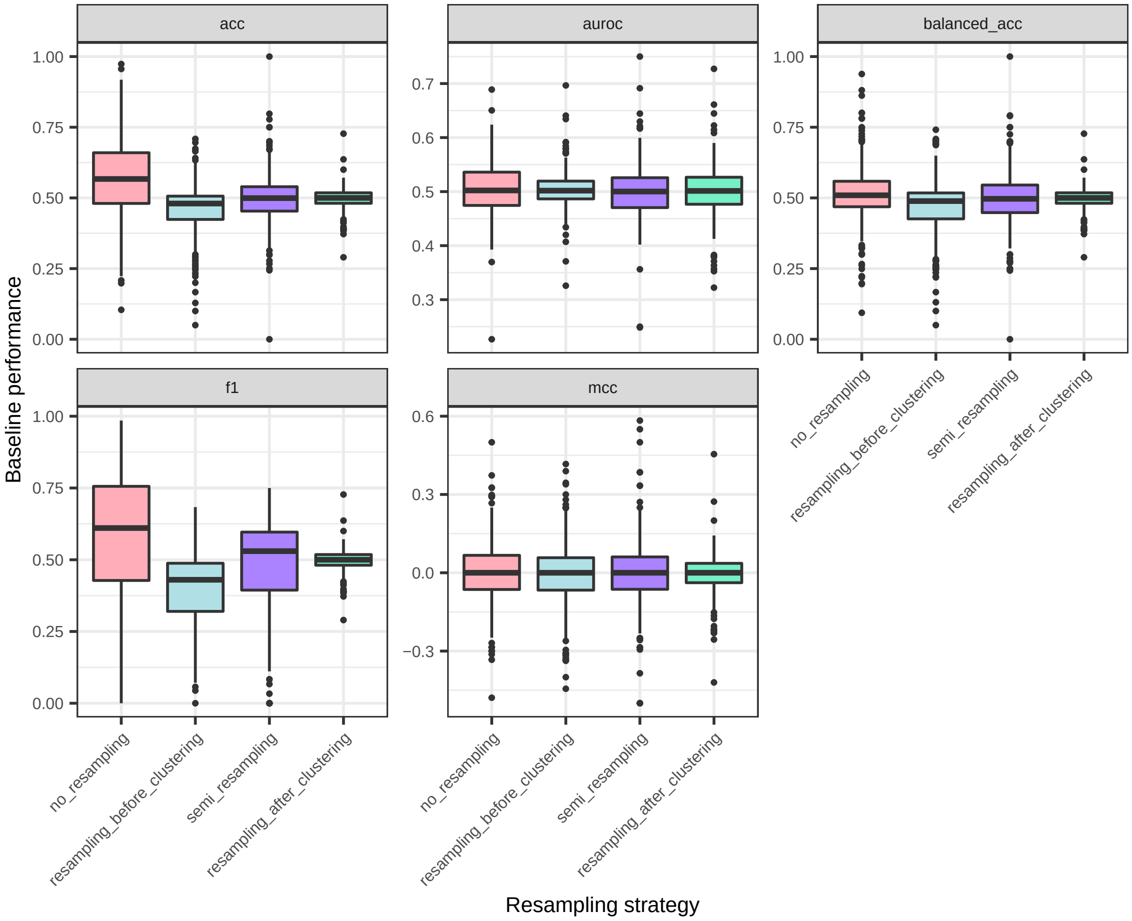

Figure 8: Performance metrics for imbalance-aware random baselines. Data points correspond to proteins, averaged over folds.

4.2 Linear models

Formally, each performance metric was described with the following linear model:

Table 9: Quartiles for the baseline F1-scores.

strategy	Min.	1st Qu.	Median	Mean	3rd Qu.	Max.
no_resampling	0.000	0.428	0.611	0.569	0.756	0.985
resampling_before_clustering	0.000	0.320	0.430	0.392	0.488	0.683
semi_resampling	0.000	0.394	0.530	0.478	0.596	0.750
resampling_after_clustering	0.290	0.481	0.500	0.497	0.518	0.727

$$\text{metric} \sim \text{strategy} + \log10(n_{int}) + \log10(n_{seq}) + k_{fold}$$

The response was the quantitative metric of interest (one model per metric), while `strategy` was categorical with the following possibilities: `no_resampling`, `resampling_after_clustering`, `resampling_before_clustering`, `semi_resampling`. Additional covariates included the number of interactions n_{int} and the sequence length n_{seq} (numerical) and the fold number k_{fold} (categorical). The `strategy` variable was tested with a type 3 ANOVA, being significant with $p < 0.05$ for `acc`, `f1` and `balanced_acc` (table 10).

Table 10: ANOVA p-values for including the resampling strategy as a regressor. Significant p-values imply that differences exist between resampling strategies.

strategy	variable	Sum Sq	Df	F value	Pr(>F)
acc	strategy	22.2816465	3	1.36e+02	5.58e-86
auroc	strategy	0.0324079	3	1.79e-01	9.11e-01
balanced_acc	strategy	3.0761723	3	1.70e+01	5.61e-11
f1	strategy	53.2175335	3	2.36e+02	8.97e-148
mcc	strategy	0.1219509	3	3.03e-01	8.24e-01

Based on this, metrics were divided in two types:

- Those where the baseline was different between strategies, i.e. imbalance-sensitive: `acc`, `f1` and `balanced_acc`. Therefore, before comparing strategies, the baseline performance needed to be accounted for.
- Those where the baseline was constant, i.e. imbalance-insensitive: `auroc`, `mcc`. Here we could compare strategies directly.

5 Description of deep learning model performance

An overview of fold-averaged performances is displayed in figure 9, where strategies are paired with their baselines. This illustrates the issue of direct strategy comparison with imbalance-sensitive metrics, which was especially visible for the F1-score. Some metrics are undefined in edge cases (e.g. AUROC when only actives or only inactives are available); table 11 summarizes the number of proteins, added over folds, whose metrics were computable.

5.1 Absolute, baseline-naive performance

Anologous to the baseline performance models, absolute metric models (not accounting for baselines) were fitted:

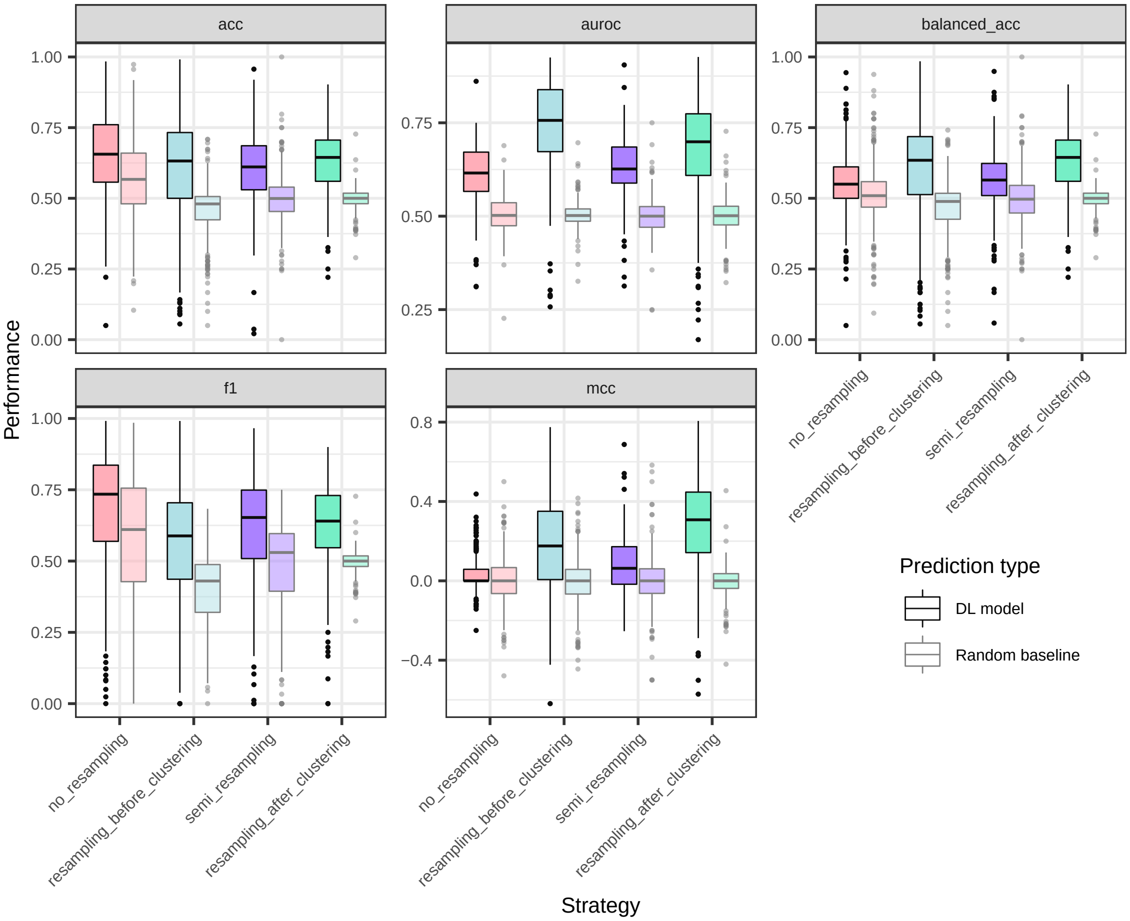

Figure 9: Performance metrics for balancing strategies and their corresponding imbalance-aware random baselines. Data points correspond to proteins, averaged over folds.

Table 11: Number of computable performance measures. AUROC was undefined for proteins with all actives or unactives in the test set, hence its lower counts.

strategy	acc	auroc	f1	balanced_acc	mcc
no_resampling	2973	2238	2973	2973	2973
resampling_before_clustering	2911	2211	2911	2911	2911
semi_resampling	2973	2238	2973	2973	2973
resampling_after_clustering	814	814	814	814	814

$$\text{metric} \sim \text{strategy} + \log 10(n_{int}) + \log 10(n_{seq}) + k_{fold}$$

The `strategy` covariate was always significant in a type 3 ANOVA (table 12). The models, summarized in 13, showed different behaviour in imbalance-sensitive and insensitive metrics. Pairwise comparisons of the strategy coefficients using Tukey's method would point to two different pictures (figure 10), further confirmed when prioritizing the strategies according to their expected performance through the linear models (table 14 and figure 11):

- Accuracy and F1-score suggested that `no_resampling` was the best strategy, but this was confounded by the fact that it also held the highest baselines.
- AUROC, MCC and balanced accuracy showed instead that `resampling_before_clustering` and `resampling_after_clustering` held the highest performance estimates.

Table 12: ANOVA p-values for including the resampling strategy as a regressor in the performance models.

strategy	variable	Sum Sq	Df	F value	Pr(>F)
acc	strategy	5.170321	3	2.37e+01	2.89e-15
auroc	strategy	10.908150	3	4.78e+01	1.28e-30
f1	strategy	29.754722	3	1.09e+02	2.07e-69
balanced_acc	strategy	5.464791	3	2.72e+01	1.64e-17
mcc	strategy	48.559151	3	1.82e+02	7.97e-115

Table 13: Linear models to describe each performance metric. Standard deviations in parentheses.

	acc	auroc	f1	balanced_acc	mcc
	(1)	(2)	(3)	(4)	(5)
strategyresampling_before_clustering	-0.046^{***}	0.084^{***}	-0.132^{***}	0.047^{***}	0.148^{***}
	(7.036e-03)	(8.279e-03)	(7.867e-03)	(6.749e-03)	(7.777e-03)
	p = 8.802e-11	p = 4.087e-24	p = 1.533e-62	p = 3.584e-12	p = 1.229e-79
strategysemi_resampling	-0.046^{***}	1.799e-03	-0.066^{***}	8.309e-03	0.041^{***}
	(6.997e-03)	(8.241e-03)	(7.823e-03)	(6.712e-03)	(7.734e-03)
	p = 3.308e-11	p = 0.827	p = 3.896e-17	p = 0.216	p = 1.524e-07
strategyresampling_after_clustering	-0.069^{***}	0.061^{***}	-0.144^{***}	0.07^{***}	0.209^{***}
	(0.011)	(0.012)	(0.013)	(0.011)	(0.012)
	p = 7.285e-10	p = 1.668e-07	p = 1.727e-30	p = 6.696e-11	p = 6.559e-63
log10(n_interactions)	0.098^{***}	0.076^{***}	0.123^{***}	0.041^{***}	0.114^{***}
	(4.212e-03)	(4.958e-03)	(4.709e-03)	(4.040e-03)	(4.655e-03)
	p = 0.000e+00	p = 6.946e-52	p = 0.000e+00	p = 2.529e-24	p = 0.000e+00
log10(len_seq)	0.052^{**}	0.024	0.053^{*}	0.028^{*}	0.043^{*}
	(0.015)	(0.017)	(0.017)	(0.014)	(0.017)
	p = 5.105e-04	p = 0.157	p = 1.612e-03	p = 0.05	p = 9.744e-03
fold1	0.041^{*}	−7.487e-03	0.036^{*}	0.033^{*}	−0.025
	(0.013)	(0.015)	(0.014)	(0.012)	(0.014)
	p = 1.037e-03	p = 0.617	p = 9.481e-03	p = 5.917e-03	p = 0.07
fold2	0.031^{*}	−0.014	0.035^{*}	0.018	−0.022
	(0.012)	(0.015)	(0.014)	(0.012)	(0.014)
	p = 0.014	p = 0.33	p = 0.012	p = 0.13	p = 0.103
fold3	0.057^{**}	1.904e-03	0.073^{***}	0.033^{*}	1.973e-04
	(0.012)	(0.014)	(0.014)	(0.012)	(0.014)
	p = 5.280e-06	p = 0.896	p = 1.650e-07	p = 5.870e-03	p = 0.989
fold4	4.646e-03	−5.580e-03	0.023	−0.01	−7.461e-03
	(0.012)	(0.014)	(0.014)	(0.012)	(0.014)
	p = 0.707	p = 0.699	p = 0.097	p = 0.384	p = 0.585
fold5	0.035^{*}	0.053^{**}	0.081^{***}	0.011	0.03^{*}
	(0.012)	(0.014)	(0.014)	(0.012)	(0.014)
	p = 4.349e-03	p = 1.862e-04	p = 5.174e-09	p = 0.345	p = 0.03
fold6	0.016	−0.011	0.018	9.628e-03	6.239e-03
	(0.012)	(0.015)	(0.014)	(0.012)	(0.014)
	p = 0.208	p = 0.455	p = 0.182	p = 0.418	p = 0.648
fold7	0.027^{*}	0.036^{*}	0.066^{**}	0.015	−2.417e-03
	(0.012)	(0.014)	(0.014)	(0.012)	(0.014)
	p = 0.031	p = 0.011	p = 1.775e-06	p = 0.198	p = 0.859
fold8	0.021	1.533e-03	0.056^{**}	−1.289e-03	−0.024
	(0.012)	(0.014)	(0.014)	(0.012)	(0.014)
	p = 0.096	p = 0.915	p = 5.076e-05	p = 0.914	p = 0.085
fold9	0.025^{*}	0.015	0.021	0.014	0.018
	(0.012)	(0.014)	(0.014)	(0.012)	(0.014)
	p = 0.045	p = 0.307	p = 0.134	p = 0.244	p = 0.193
Constant	0.274^{***}	0.347^{***}	0.243^{**}	0.378^{***}	-0.318^{***}
	(0.045)	(0.051)	(0.05)	(0.043)	(0.049)
	p = 7.742e-10	p = 1.421e-11	p = 1.039e-06	p = 1.020e-18	p = 1.102e-10
Observations	9671	7501	9671	9671	9671
R^2	0.063	0.059	0.096	0.027	0.134
Adjusted R^2	0.062	0.057	0.095	0.026	0.133

Note: *p<0.05; **p<1.000e-03; ***p<1e-06

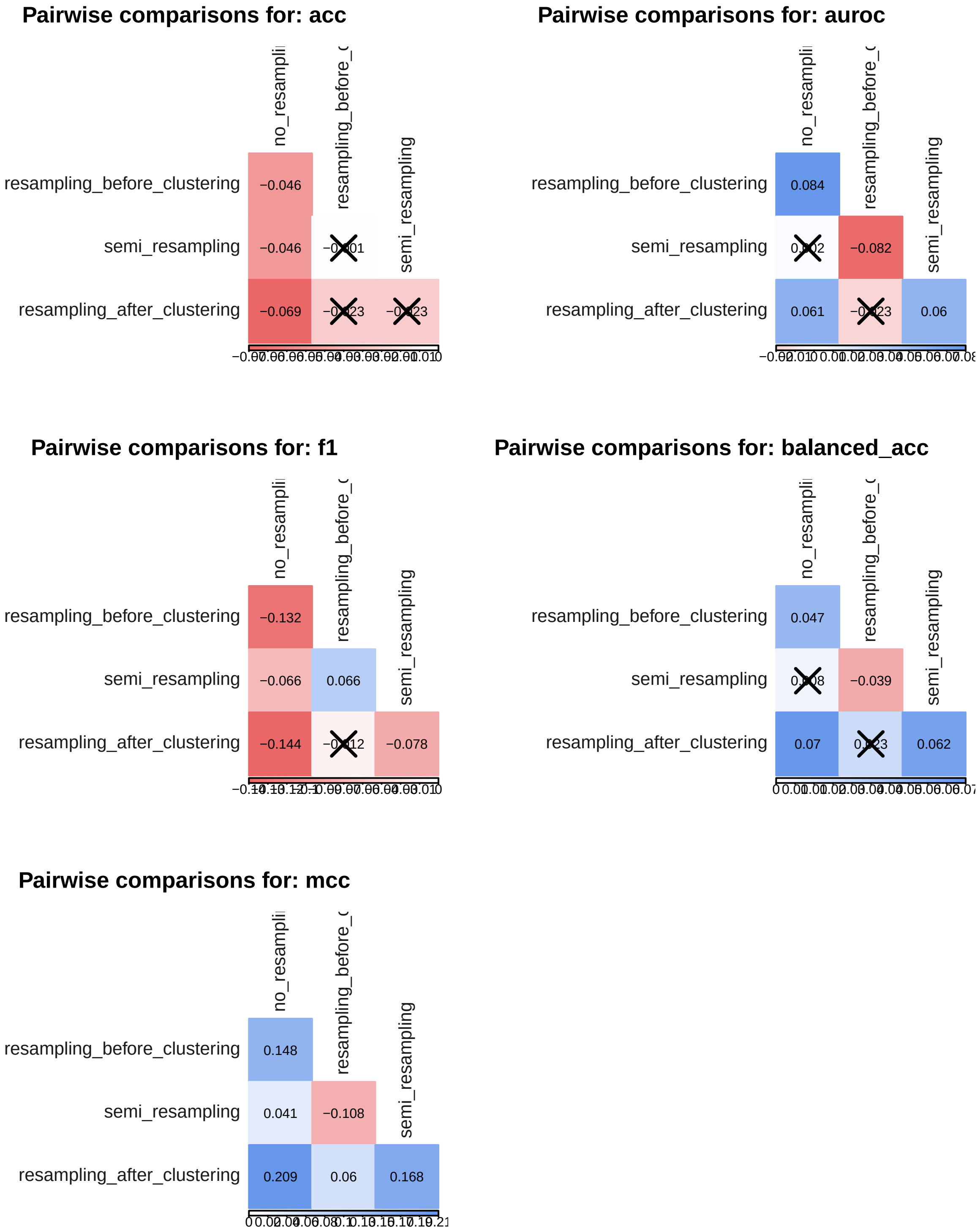

Figure 10: Pairwise comparison of strategy performance using Tukey method.

Table 14: Expected absolute performances, by metric and strategy, with 95% confidence intervals.

metric	strategy	emmean	SE	lower.CL	upper.CL
acc	no_resampling	0.712	5.614e-03	0.701	0.723
acc	resampling_before_clustering	0.666	5.618e-03	0.655	0.677
acc	semi_resampling	0.665	5.614e-03	0.654	0.676
acc	resampling_after_clustering	0.643	9.511e-03	0.624	0.662
auroc	no_resampling	0.627	6.280e-03	0.615	0.640
auroc	resampling_before_clustering	0.712	6.257e-03	0.699	0.724
auroc	semi_resampling	0.629	6.280e-03	0.617	0.641
auroc	resampling_after_clustering	0.689	9.737e-03	0.670	0.708
f1	no_resampling	0.766	6.277e-03	0.754	0.779
f1	resampling_before_clustering	0.634	6.282e-03	0.622	0.646
f1	semi_resampling	0.700	6.277e-03	0.688	0.713
f1	resampling_after_clustering	0.622	1.063e-02	0.601	0.643
balanced_acc	no_resampling	0.582	5.385e-03	0.572	0.593
balanced_acc	resampling_before_clustering	0.629	5.389e-03	0.619	0.640
balanced_acc	semi_resampling	0.591	5.385e-03	0.580	0.601
balanced_acc	resampling_after_clustering	0.652	9.123e-03	0.634	0.670
mcc	no_resampling	0.108	6.205e-03	0.096	0.120
mcc	resampling_before_clustering	0.256	6.210e-03	0.244	0.268
mcc	semi_resampling	0.149	6.205e-03	0.136	0.161
mcc	resampling_after_clustering	0.317	1.051e-02	0.296	0.337

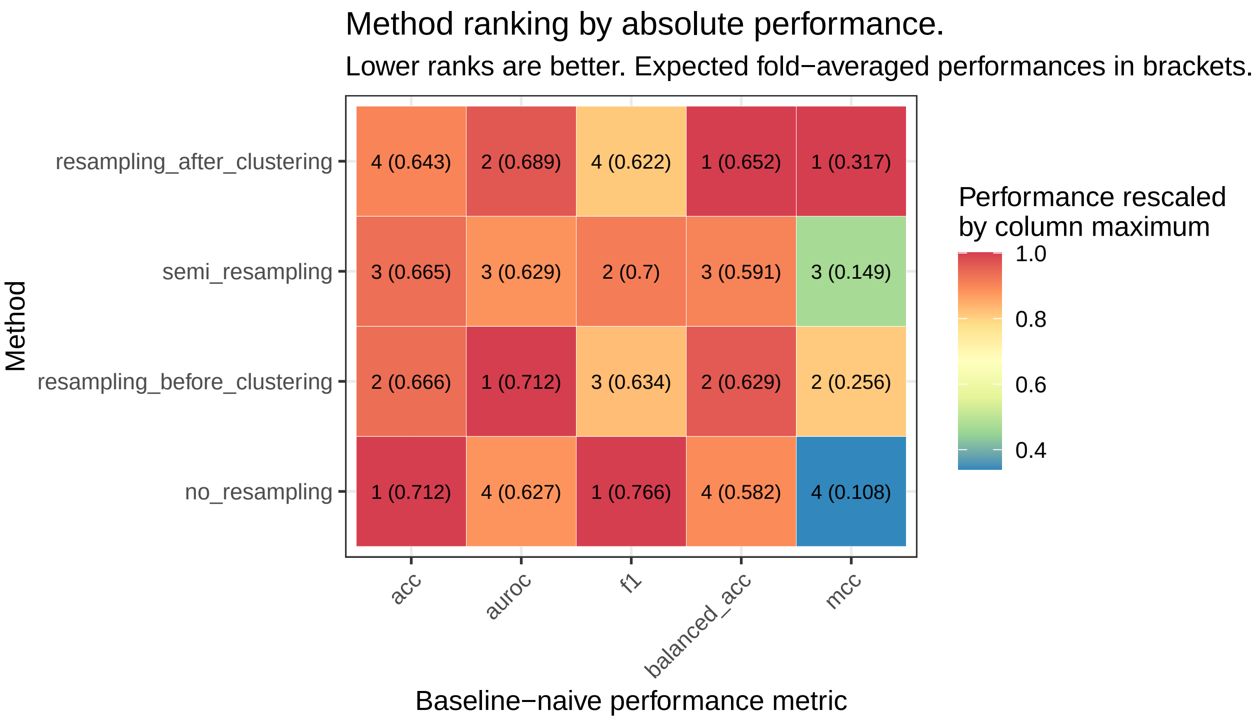

Figure 11: Method ranking according to the linear model predicted performances for each metric. Baseline metrics were ignored.

5.2 Baseline-adjusted performance

To address the pitfalls of the direct comparison of metrics whose baselines may differ, baseline-adjusted performance metrics were defined and modelled analogously. Specifically:

$$\text{adj\_metric} = \text{metric} - \text{baseline}$$

A descriptive plot of the the adjusted metrics (figure 12) pointed to a scenario diffent than that of unadjusted ones (figure 9).

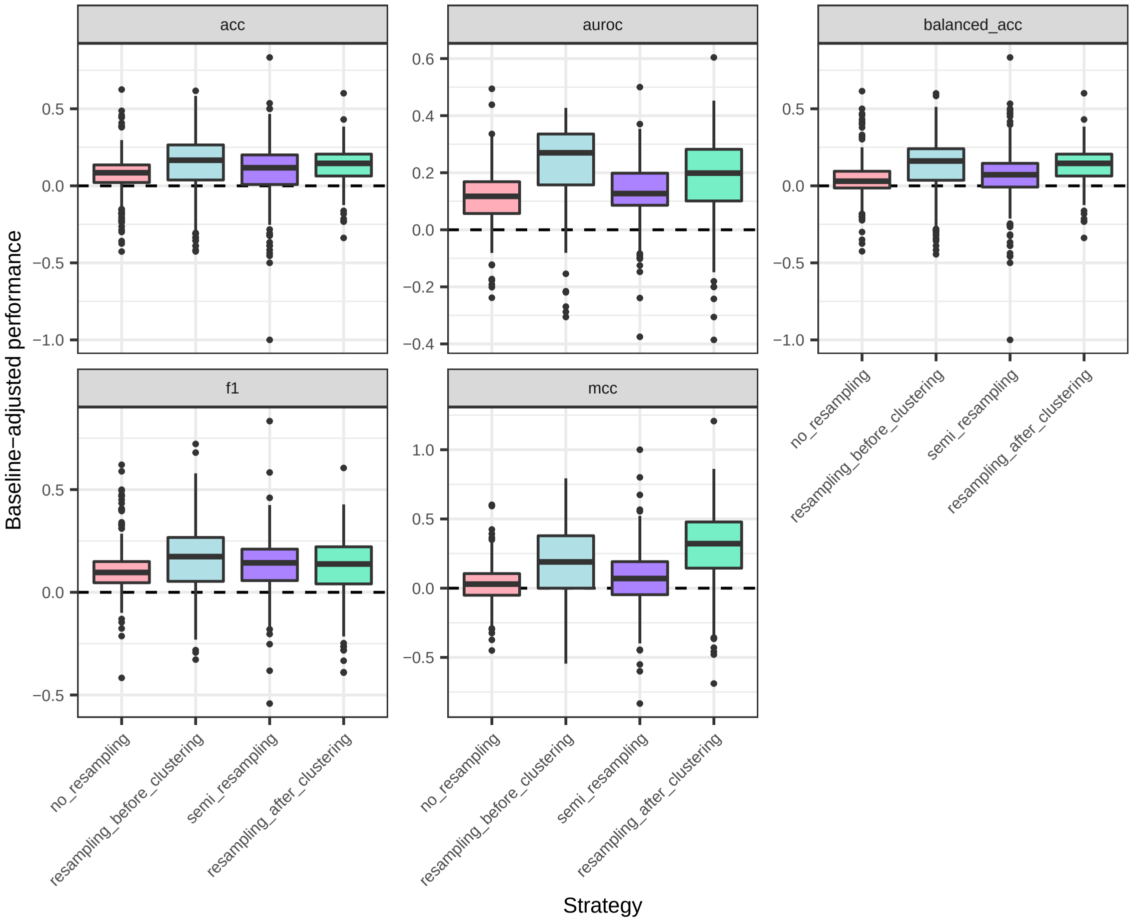

Figure 12: Baseline-adjusted performance metrics for balancing strategies. Data points correspond to proteins, averaged over folds.

Adjusted performance metrics were described with the following linear model:

$$\text{adj\_metric} \sim \text{strategy} + \log10(n_{int}) + \log10(n_{seq}) + k_{fold}$$

Note that while all metrics but `mcc` were non-negative, the adjusted metrics could show negative values when the performance of the DL model was lower than that of the baseline.

Again, `strategy` covariate was always significant in a type 3 ANOVA (table 15). Baseline adjustment brought a uniform behaviour across the models (table 16), further confirmed in pairwise coefficient comparison (Tukey's method, figure 13) and in their expected performance (table 17 and figure 14):

- `resampling_before_clustering` and `resampling_after_clustering` had the highest performance estimates, followed by `semi_resampling` and finally by `no_resampling`.
- This picture was analogous to that of the non-adjusted performance metrics that were imbalance-insensitive (AUROC, MCC).

Table 15: ANOVA p-values for including the resampling strategy as a regressor in the adjusted performance models.

strategy	variable	Sum Sq	Df	F value	Pr(>F)
acc	strategy	7.228605	3	2.09e+01	1.79e-13
auroc	strategy	12.123911	3	2.96e+01	4.81e-19
f1	strategy	3.665732	3	1.43e+01	2.78e-09
balanced_acc	strategy	15.297979	3	4.28e+01	1.82e-27
mcc	strategy	52.471976	3	7.77e+01	1.29e-49

Conclusions drawn from the baseline-adjusted performance analysis:

- The largest impact in performance estimates was the application of **data augmentation to the test set**: `resampling_before_clustering` and `resampling_after_clustering` tended to outperform `semi_resampling` and `no_resampling` (Tukey's method, $p < 0.05$, figure 13). However, augmenting the test set might not faithfully reflect new data anymore, and could **artificially inflate the peformance estimates**.
- `semi_resampling` outperformed `no_resampling` in four out of five metrics (Tukey's method, $p < 0.05$, figure 13), which **supports data augmentation usefulness** even if the data balance in the test set differs from that of the training set. This was consistent with the observation that the predicted proportion of positives of the PCM model was mainly driven by the actual data balance in the test set, rather than that of the training set. Combined with the healthier distributions of predicted active ratios of `semi_resampling` against `no_resampling`, this made a case in favour of the former.
- In four out of five metrics, **proteins with more interactions were better predicted** (table 16).

Table 16: Linear models to describe each adjusted performance metric. Standard deviations in parentheses.

	acc	auroc	f1	balanced_acc	mcc
	(1)	(2)	(3)	(4)	(5)
strategyresampling_before_clustering	0.07^{***}	0.087^{***}	0.05^{***}	0.093^{***}	0.157^{***}
	(8.861e-03)	(0.011)	(7.628e-03)	(9.002e-03)	(0.012)
	p = 3.431e-15	p = 3.742e-15	p = 6.971e-11	p = 1.129e-24	p = 1.213e-36
strategysemi_resampling	0.037^{**}	−2.411e-03	0.026^{**}	0.029^{*}	0.041^{*}
	(8.966e-03)	(0.011)	(7.719e-03)	(9.108e-03)	(0.013)
	p = 4.267e-05	p = 0.829	p = 7.937e-04	p = 1.332e-03	p = 1.190e-03
strategyresampling_after_clustering	0.043^{*}	0.06^{**}	0.03^{*}	0.098^{***}	0.209^{***}
	(0.014)	(0.016)	(0.012)	(0.014)	(0.02)
	p = 2.204e-03	p = 1.412e-04	p = 0.013	p = 9.770e-12	p = 5.834e-26
log10(n_interactions)	0.053^{***}	0.079^{***}	4.567e-03	0.026^{**}	0.118^{***}
	(5.390e-03)	(6.740e-03)	(4.640e-03)	(5.475e-03)	(7.529e-03)
	p = 2.346e-22	p = 3.209e-31	p = 0.325	p = 2.408e-06	p = 9.075e-55
log10(len_seq)	0.046^{*}	0.018	0.021	0.02	0.025
	(0.019)	(0.023)	(0.016)	(0.019)	(0.027)
	p = 0.016	p = 0.439	p = 0.2	p = 0.294	p = 0.35
fold1	0.029	−6.058e-03	7.826e-03	0.021	−0.023
	(0.016)	(0.02)	(0.014)	(0.016)	(0.022)
	p = 0.071	p = 0.763	p = 0.567	p = 0.183	p = 0.296
fold2	0.024	−0.026	−8.380e-03	0.014	-0.044^{*}
	(0.016)	(0.02)	(0.014)	(0.016)	(0.022)
	p = 0.126	p = 0.193	p = 0.539	p = 0.388	p = 0.046
fold3	0.049^{*}	−3.319e-03	0.027^{*}	0.029	−0.022
	(0.016)	(0.02)	(0.014)	(0.016)	(0.022)
	p = 1.775e-03	p = 0.865	p = 0.046	p = 0.073	p = 0.317
fold4	9.957e-03	1.882e-03	0.013	−7.284e-04	3.653e-03
	(0.016)	(0.019)	(0.014)	(0.016)	(0.022)
	p = 0.527	p = 0.923	p = 0.343	p = 0.964	p = 0.868
fold5	0.024	0.057^{*}	0.024	9.004e-03	0.025
	(0.016)	(0.019)	(0.014)	(0.016)	(0.022)
	p = 0.134	p = 3.174e-03	p = 0.079	p = 0.575	p = 0.255
fold6	2.762e-03	−0.023	3.872e-03	7.173e-04	−6.218e-03
	(0.016)	(0.02)	(0.014)	(0.016)	(0.022)
	p = 0.861	p = 0.248	p = 0.775	p = 0.964	p = 0.777
fold7	0.022	0.032	0.033^{*}	0.013	−0.011
	(0.016)	(0.019)	(0.013)	(0.016)	(0.022)
	p = 0.159	p = 0.099	p = 0.015	p = 0.406	p = 0.601
fold8	7.448e-03	−3.576e-04	5.280e-03	−0.012	−0.033
	(0.016)	(0.019)	(0.014)	(0.016)	(0.022)
	p = 0.636	p = 0.985	p = 0.697	p = 0.457	p = 0.132
fold9	0.019	7.796e-03	−9.248e-03	9.900e-03	−2.422e-03
	(0.016)	(0.019)	(0.013)	(0.016)	(0.022)
	p = 0.216	p = 0.684	p = 0.493	p = 0.534	p = 0.912
Constant	-0.184^{*}	-0.141^{*}	0.03	−0.08	-0.273^{**}
	(0.057)	(0.069)	(0.049)	(0.057)	(0.079)
	p = 1.143e-03	p = 0.041	p = 0.534	p = 0.166	p = 5.493e-04
Observations	9473	7387	9473	9473	9473
R^2	0.02	0.036	7.025e-03	0.019	0.061
Adjusted R^2	0.019	0.035	5.555e-03	0.017	0.06

Note: *p<0.05; **p<1.000e-03; ***p<1e-06

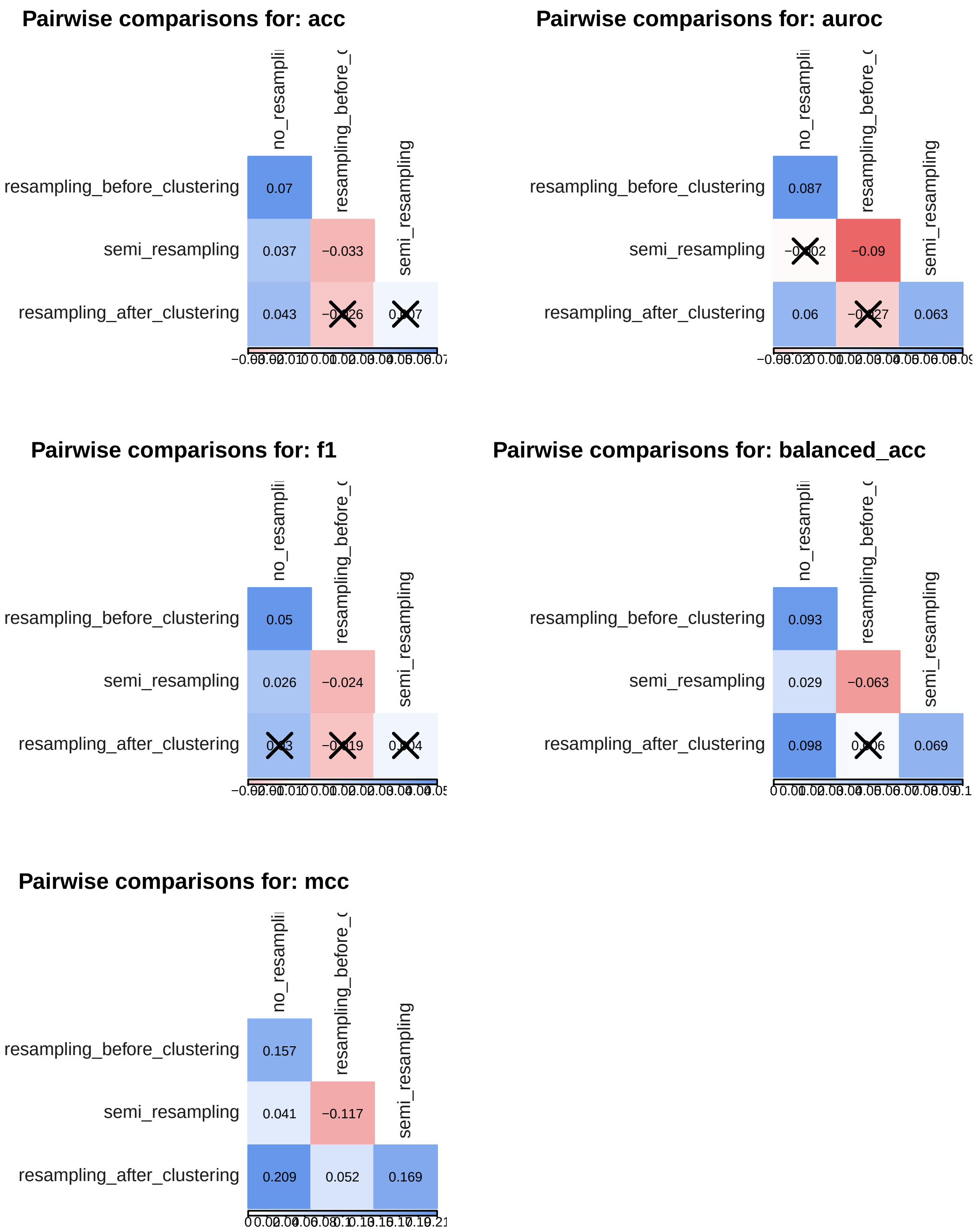

Figure 13: Pairwise comparison of strategy adjusted performance using Tukey method.

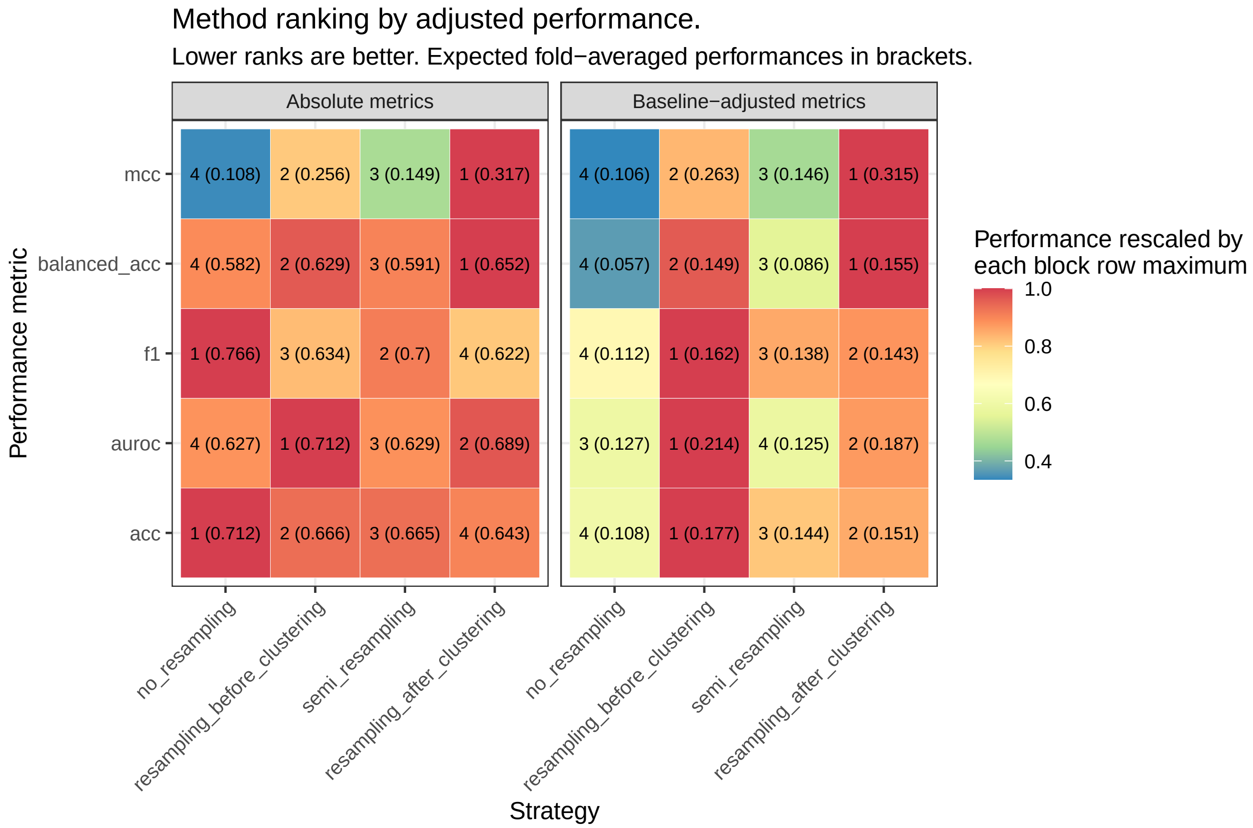

Figure 14: Method ranking according to the linear model predicted adjusted performances for each metric. Baseline metrics were taken into account in the adjustment. For a direct comparison, the same ranking using the absolute metrics was kept side by side.

Table 17: Expected adjusted performances, by metric and strategy, with 95% confidence intervals.

metric	strategy	emmean	SE	lower.CL	upper.CL
acc	no_resampling	0.108	7.116e-03	0.094	0.122
acc	resampling_before_clustering	0.177	7.119e-03	0.164	0.191
acc	semi_resampling	0.144	7.180e-03	0.130	0.158
acc	resampling_after_clustering	0.151	1.204e-02	0.127	0.175
auroc	no_resampling	0.127	8.448e-03	0.110	0.144
auroc	resampling_before_clustering	0.214	8.413e-03	0.198	0.231
auroc	semi_resampling	0.125	8.528e-03	0.108	0.141
auroc	resampling_after_clustering	0.187	1.311e-02	0.161	0.213
f1	no_resampling	0.112	6.126e-03	0.100	0.124
f1	resampling_before_clustering	0.162	6.128e-03	0.150	0.174
f1	semi_resampling	0.138	6.181e-03	0.126	0.150
f1	resampling_after_clustering	0.143	1.036e-02	0.122	0.163
balanced_acc	no_resampling	0.057	7.229e-03	0.043	0.071
balanced_acc	resampling_before_clustering	0.149	7.232e-03	0.135	0.163
balanced_acc	semi_resampling	0.086	7.294e-03	0.072	0.100
balanced_acc	resampling_after_clustering	0.155	1.223e-02	0.131	0.179
mcc	no_resampling	0.106	9.940e-03	0.086	0.125
mcc	resampling_before_clustering	0.263	9.944e-03	0.243	0.282
mcc	semi_resampling	0.146	1.003e-02	0.127	0.166
mcc	resampling_after_clustering	0.315	1.682e-02	0.282	0.348

6 Reproducibility

- R version 3.6.3 (2020-02-29), `x86_64-pc-linux-gnu`
- Locale: `LC_CTYPE=en_US.UTF-8`, `LC_NUMERIC=C`, `LC_TIME=es_ES.UTF-8`, `LC_COLLATE=en_US.UTF-8`, `LC_MONETARY=es_ES.UTF-8`, `LC_MESSAGES=en_US.UTF-8`, `LC_PAPER=es_ES.UTF-8`, `LC_NAME=C`, `LC_ADDRESS=C`, `LC_TELEPHONE=C`, `LC_MEASUREMENT=es_ES.UTF-8`, `LC_IDENTIFICATION=C`
- Running under: `Ubuntu 16.04.7 LTS`
- Matrix products: default
- BLAS: `/usr/lib/atlas-base/atlas/libblas.so.3.0`
- LAPACK: `/usr/lib/atlas-base/atlas/liblapack.so.3.0`
- Base packages: base, datasets, graphics, grDevices, methods, stats, utils
- Other packages: corrplot 0.84, dplyr 1.0.5, forcats 0.5.1, ggplot2 3.3.3, gsubfn 0.7, kableExtra 1.3.4, magrittr 2.0.1, proto 1.0.0, purrr 0.3.4, readr 1.4.0, rmarkdown 2.7, stargazer 5.2.2, stringr 1.4.0, tibble 3.1.0, tidyr 1.1.3, tidyverse 1.3.0
- Loaded via a namespace (and not attached): abind 1.4-5, assertthat 0.2.1, backports 1.2.1, bookdown 0.21, broom 0.7.5, car 3.0-10, carData 3.0-4, cellranger 1.1.0, cli 2.3.1, codetools 0.2-16, colorspace 2.0-0, compiler 3.6.3, crayon 1.4.1, curl 4.3, data.table 1.14.0, DBI 1.1.1, dbplyr 2.1.0, digest 0.6.27, ellipsis 0.3.1, emmeans 1.5.4, estimability 1.3, evaluate 0.14, fansi 0.4.2, farver 2.1.0, foreign 0.8-76, fs 1.5.0, generics 0.1.0, glue 1.4.2, grid 3.6.3, gtable 0.3.0, haven 2.3.1, highr 0.8, hms 1.0.0, htmltools 0.5.1.1, httr 1.4.2, jsonlite 1.7.2, knitr 1.31, labeling 0.4.2, lattice 0.20-41, lifecycle 1.0.0, lubridate 1.7.10, MASS 7.3-53, Matrix 1.2-18, mgcv 1.8-33, modelr 0.1.8, multcomp 1.4-16, munsell 0.5.0, mvtnorm 1.1-1, nlme 3.1-149, openxlsx 4.2.3, pillar 1.5.1,

pkgconfig 2.0.3, plyr 1.8.6, ps 1.6.0, R6 2.5.0, RColorBrewer 1.1-2, Rcpp 1.0.6, readxl 1.3.1, reprex 1.0.0, reshape2 1.4.4, rio 0.5.26, rlang 0.4.10, rstudioapi 0.13, rvest 0.3.6, sandwich 3.0-0, scales 1.1.1, splines 3.6.3, stringi 1.5.3, survival 3.2-7, svglite 2.0.0, systemfonts 1.0.1, tcltk 3.6.3, TH.data 1.0-10, tidyselect 1.1.0, tools 3.6.3, utf8 1.1.4, vctrs 0.3.6, viridisLite 0.3.0, webshot 0.5.2, withr 2.4.1, xfun 0.21, xml2 1.3.2, xtable 1.8-4, yaml 2.2.1, zip 2.1.1, zoo 1.8-8

Appendix 3: model predictions and performance (GPCRs)

Angela Lopez-del Rio   Sergio Picart-Armada   Alexandre Perera-Lluna

06/01/2021

Contents

1 Overview 1

2 Description of data balance 2
2.1 Distributions of the actives ratio 2
2.2 Comparing training and test imbalance 2
2.3 Other covariates 4

3 Linear models on predicted proportions 4
3.1 Distributions of the predicted ratios 5
3.2 Predicted ratios against training ratios 7
3.3 Linear models 8
3.4 Conclusions 8

4 Description of baseline performance 11
4.1 Descriptive plot 11
4.2 Linear models 12

5 Description of deep learning model performance 12
5.1 Absolute, baseline-naive performance 14
5.2 Baseline-adjusted performance 18

6 Reproducibility 23

1 Overview

This supplement describes the behaviour of the proteochemometrics (PCM) deep learning model to predict protein-compound bioactivity for **GPCRs**. Specifically, this includes the descriptive statistics of data imbalance: the proportion of actives per protein in the training and test sets during the model fitting and the predicted proportion of actives. The model performance per protein was also described, pinpointing the most influential factors and characterising the proteins with the most extreme performances.

Four strategies (no_resampling, resampling_before_clustering, semi_resampling, resampling_after_clustering) were considered. For each of those, 10 folds of repeated holdout were run, and 5 performance metrics were computed: acc, auroc, f1, balanced_acc, mcc. This led to a total of 10772 values of performance. Since some strategies involved the upsampling method SMOTE, proteins whose sample sizes did not allow upsampling were excluded (table 1).

Table 1: Number of proteins for which performance metrics were computed. The resampling after clustering was the most stringent strategy regarding eligible proteins, since the resampling was carried out after the clustering, which introduced more imbalance.

Strategy	Fold 0	Fold 1	Fold 2	Fold 3	Fold 4	Fold 5	Fold 6	Fold 7	Fold 8	Fold 9
no_resampling	347	356	359	320	332	331	342	336	341	350
resampling_before_clustering	274	275	284	277	274	256	264	286	271	280
semi_resampling	347	356	359	320	332	331	342	336	341	350
resampling_after_clustering	135	120	125	123	102	116	118	120	122	122

2 Description of data balance

The data balancing strategy had an impact on the actual data balance, defined as the proportion of active molecules for a protein. Furthermore, specific trends were observed in the original data in the training and test sets, as well as in the values predicted by the deep learning model.

2.1 Distributions of the actives ratio

The histograms in figure 1 revealed trends:

- `no_resampling` **keeps similar data imbalance in training and test.**
- `resampling_before_clustering` and `semi_resampling` **lead to a more balanced training set, but not so much for the test set.**
- `resampling_after_clustering` **kept balanced proteins** in both training and test sets.

In addition, **test sets with imbalance tended to magnify** it and create extreme cases (all actives or all inactives), probably due to the combination of the clustering and the lower sample sizes in the test sets compared to training.

2.2 Comparing training and test imbalance

Figure 2 revealed both positive, negative and null trends between the training and test set protein balances.

- `no_resampling` showed a **positive relation** between both, i.e. proteins were prone to keep their (im)balance in train and test.
- `resampling_before_clustering` showed an **inverse relationship** instead. This was expected since this strategy started from globally balanced proteins, and after the clustering, an imbalance in one direction in the training set entailed an inverse imbalance in the test set.
- `semi_resampling` led to **a slight positive correlation, but weaker than no resampling**.
- `resampling_after_clustering` always **kept balanced proteins**, by design.

Table 2 displays the Pearson correlation estimate, 95% confidence interval and p-value for each strategy (except `resampling_after_clustering`, where ratios are constant), further confirming the claims above.

Table 2: Correlations between train and test active ratios. 95% confidence intervals and p-values are shown.

strategy	cor	ci_lower	ci_upper	alternative	pvalue
no_resampling	0.594	0.572	0.616	two.sided	6.73e-320
resampling_before_clustering	-0.386	-0.417	-0.354	two.sided	4.80e-98
semi_resampling	0.067	0.029	0.105	two.sided	5.91e-04
resampling_after_clustering	NA	NA	NA	two.sided	NA

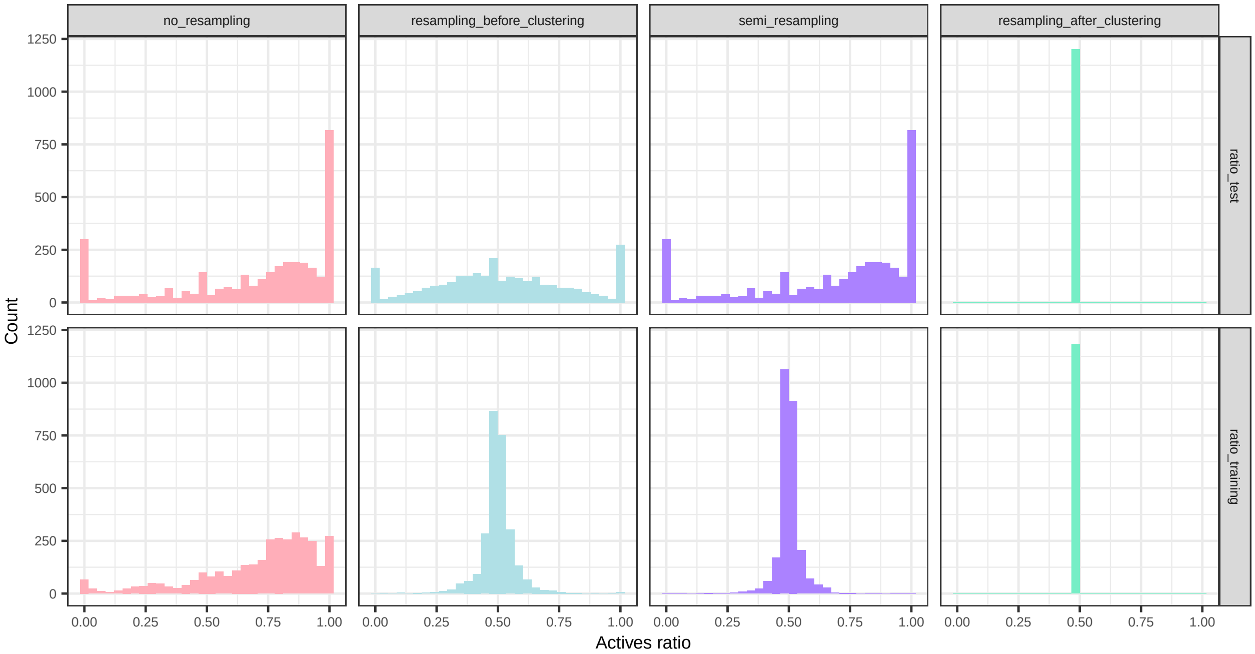

Figure 1: Distributios of the actives ratio in the training set and in the test set (both original and predicted by the deep learning model).

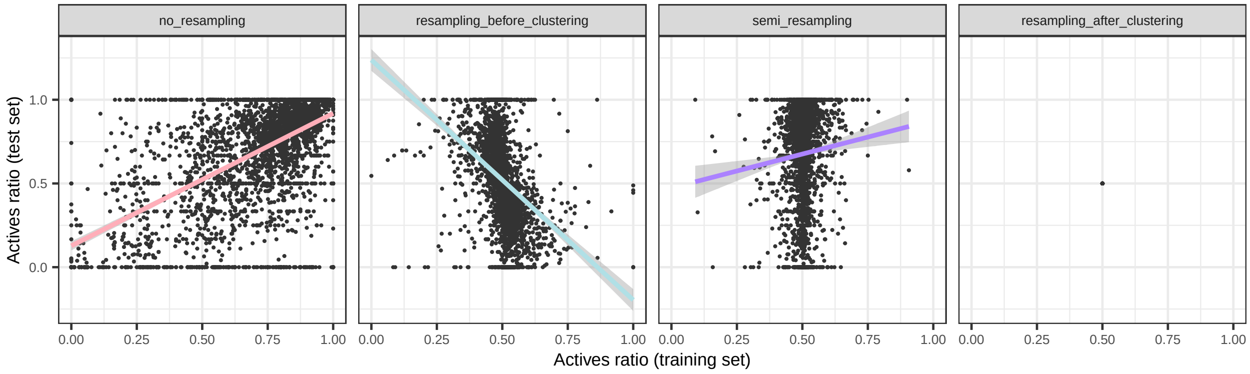

Figure 2: Comparison of the training and test ratios, by resampling strategy. A linear fit line was added per strategy.

2.3 Other covariates

The effect of the number of interactions of each protein in its corresponding set and fold (figure 3) and the protein length in amino acids (figure 4) on the test set imbalance was investigated:

- **Proteins with greatest imbalance** (i.e. where `(0.5 - ratio_test)^2` was greatest) **tended to be among those with the least interactions. Linear correlations were significant** (table 3).
- **The sequence length had no obvious effect on the protein imbalance. Linear correlations were not significant** (`no_resampling`, `semi_resampling`) or significant but low (`resampling_before_clustering`).

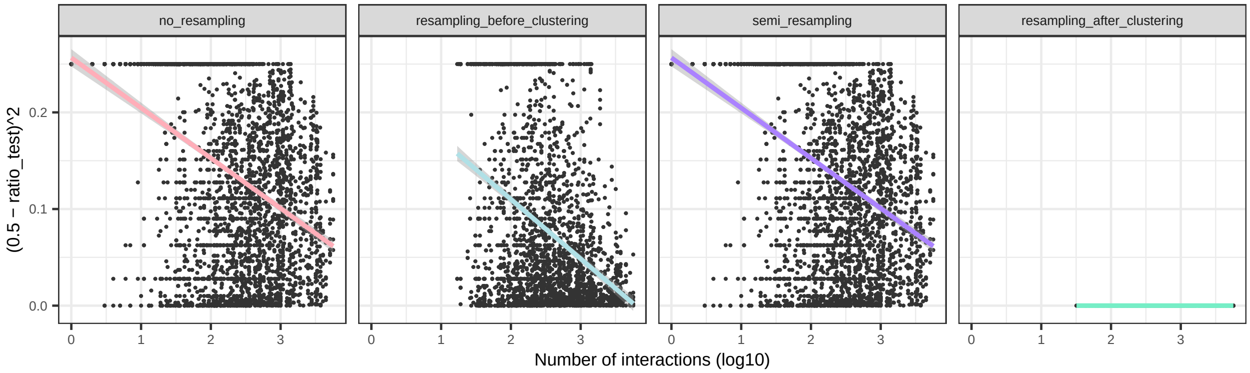

Figure 3: Data imbalance in the test set as a function of the number of available interactions for each protein.

Table 3: Correlations between imbalance (as defined above) and number of interactions. 95% confidence intervals and p-values are shown.

strategy	cor	ci_lower	ci_upper	alternative	pvalue
no_resampling	-0.246	-0.277	-0.214	two.sided	3.72e-48
resampling_before_clustering	-0.305	-0.338	-0.270	two.sided	5.43e-60
semi_resampling	-0.246	-0.277	-0.214	two.sided	3.72e-48
resampling_after_clustering	NA	NA	NA	two.sided	NA

Table 4: Correlations between imbalance (as defined above) and sequence length. 95% confidence intervals and p-values are shown.

strategy	cor	ci_lower	ci_upper	alternative	pvalue
no_resampling	-0.028	-0.062	0.005	two.sided	9.83e-02
resampling_before_clustering	-0.059	-0.097	-0.022	two.sided	1.90e-03
semi_resampling	-0.028	-0.062	0.005	two.sided	9.83e-02
resampling_after_clustering	NA	NA	NA	two.sided	NA

3 Linear models on predicted proportions

The next key question was to narrow down the factor driving the predicted proportion of actives. The main options under consideration were:

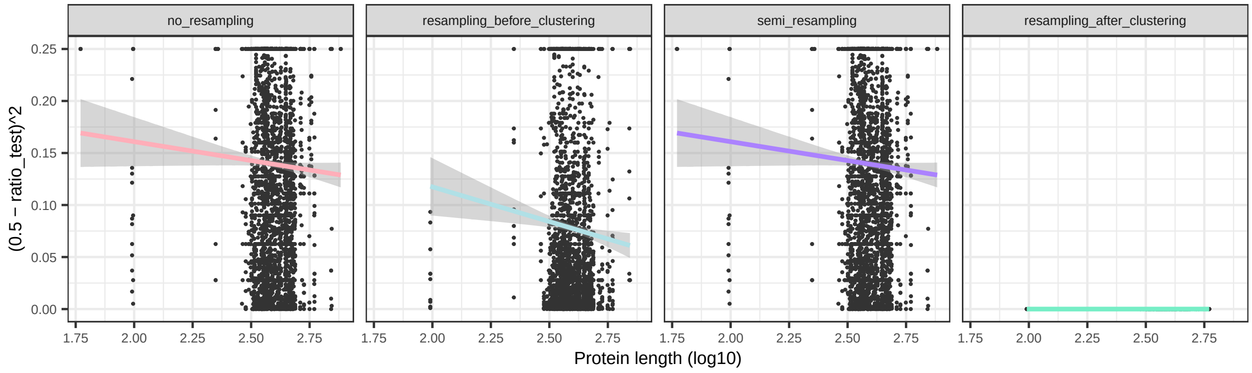

Figure 4: Data imbalance in the test set as a function of the protein length, in amino acids.

1. A constant, global imbalance that the model would learn from the whole dataset.
2. The protein-wise imbalance that the model would learn in the training set.
3. A test set-driven imbalance, based on its actual imbalance.

3.1 Distributions of the predicted ratios

After the model predictions in the test set were binarized (actives were those whose probabilities exceeded 0.5), the ratio of predicted actives was computed by protein. This ratio, shown in figure 5, suggested that:

- `no_resampling` was noticeably **biased to predict everything as positives**.
- `resampling_before_clustering` and `semi_resampling` **alleviated the imbalance in the predictions, but still retained a spike of proteins where all the compounds were predicted as positives**.
- `resampling_after_clustering` **kept a wide and symmetric distribution of predicted actives**.

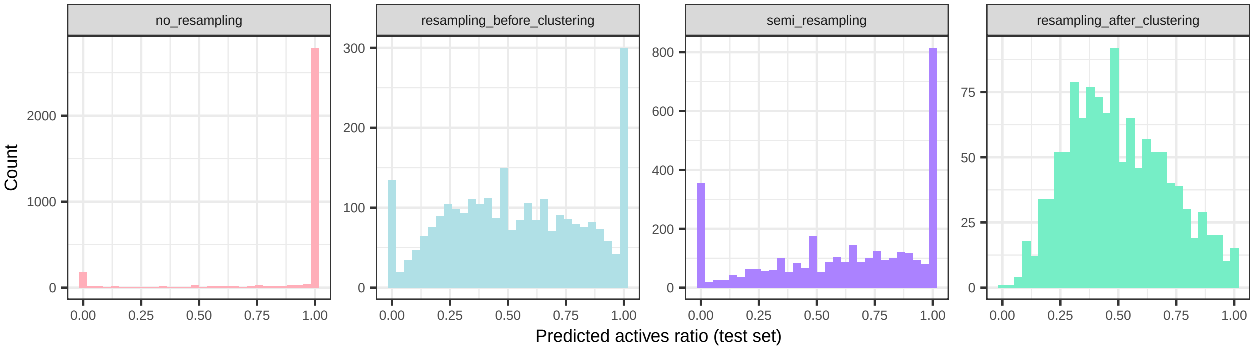

Figure 5: Ratios of the prediction values, after binarization.

Now, representing together (1) the original training and test ratios, and (2) the predicted ratios in test (figure 6) eased a general qualitative assessment: **the distribution was most resemblant to that of the test proportions to that of the training ones** (except `resampling_after_clustering`, since those proportions are constant). Table 5 displays how `no_resampling` was **highly inclined to predict all positives**, `resampling_before_clustering` and `semi_resampling` **alleviated this phenomenon**, and `resampling_after_clustering` was **essentially balanced**.

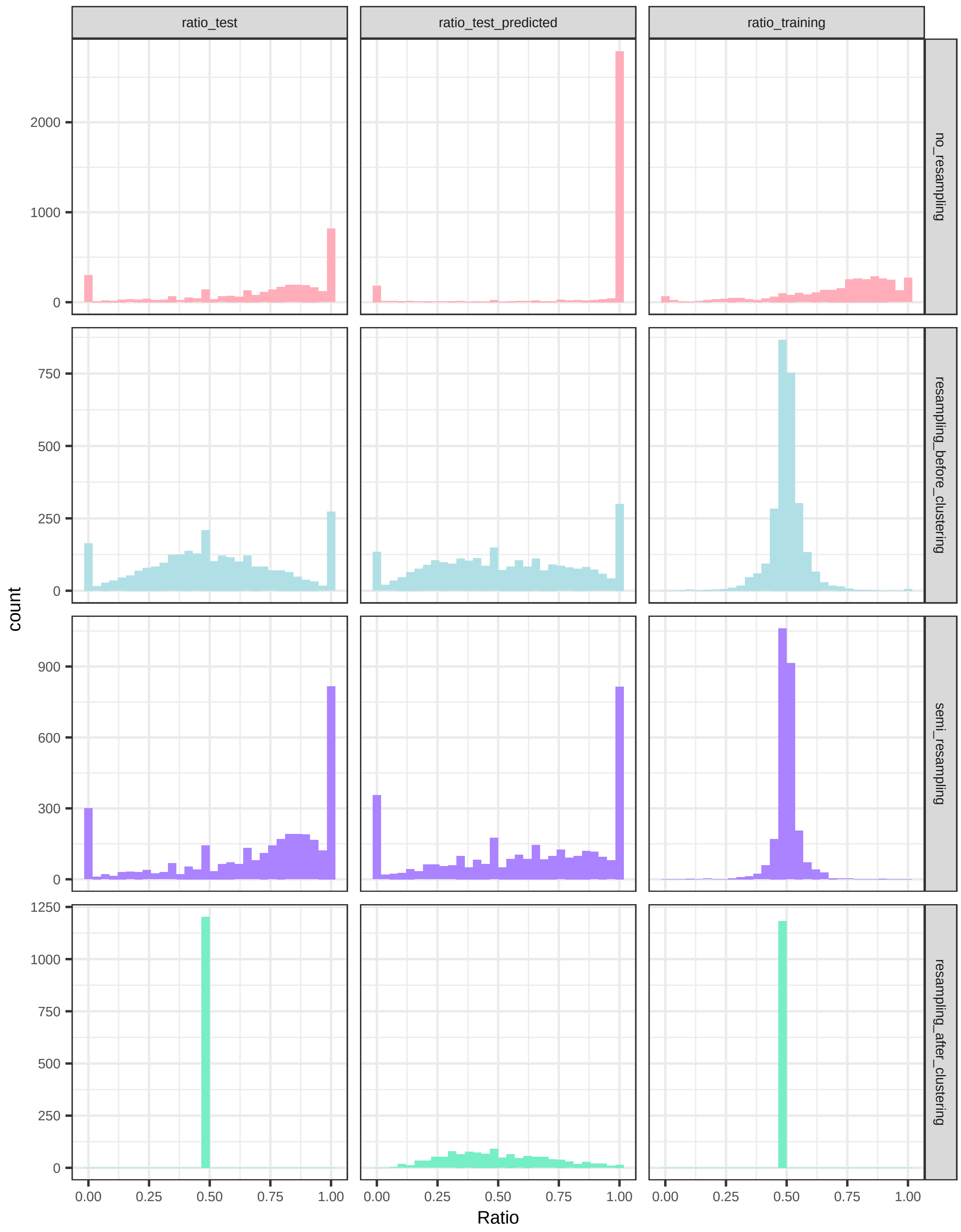

Figure 6: Distributios of the actives ratio in the training set and in the test set (both original and predicted by the deep learning model).

Table 5: Percentage of extreme cases, i.e. proteins with all actives or inactives.

strategy	RatioSet	all_inactives	all_actives	all_extremes
no_resampling	ratio_test	8.8	23.3	32.0
no_resampling	ratio_test_predicted	5.3	80.7	86.0
no_resampling	ratio_training	1.8	7.6	9.4
resampling_before_clustering	ratio_test	5.8	9.7	15.6
resampling_before_clustering	ratio_test_predicted	4.7	10.7	15.4
resampling_before_clustering	ratio_training	0.0	0.2	0.3
semi_resampling	ratio_test	8.8	23.3	32.0
semi_resampling	ratio_test_predicted	10.4	23.3	33.7
semi_resampling	ratio_training	0.0	0.0	0.0
resampling_after_clustering	ratio_test	0.0	0.0	0.0
resampling_after_clustering	ratio_test_predicted	0.1	1.1	1.2
resampling_after_clustering	ratio_training	0.0	0.0	0.0

3.2 Predicted ratios against training ratios

Figure 7 puts the predicted ratios in context of the training ratios, elucidating a variety of trends:

- `no_resampling`: **positive trend between the training and the predicted ratio**, but since the training and the test ratio also positively correlated (figure 2), the latter could be the one driving the predicted ratio of positives.
- `resampling_after_clustering` had a **constant training ratio**, meaning that the predicted ratio was not explainable by differences in training ratios.
- `resampling_before_clustering` showed instead a **negative relation between the training and the predicted ratio**. But since the former and the test ratio also anticorrelated (figure 2, the simplest explanation was that the test ratio drove the predicted test ratio.
- `semi_resampling` showed **no apparent correlation** between the predicted ratio and the training ratio.

The significance of the linear correlation backs up all the claims above (table 6).

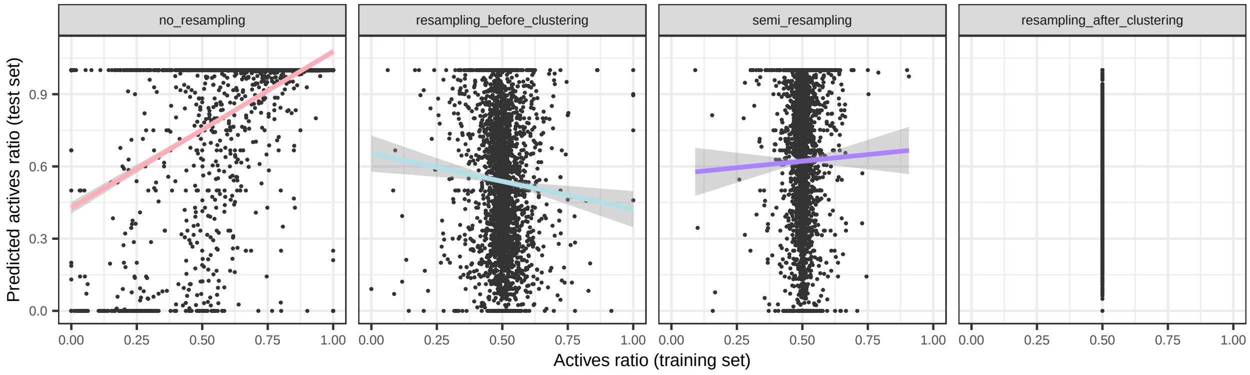

Figure 7: Predicted ratios, as a function of training ratios.

Table 6: Correlations between train and predicted test active ratios. 95% confidence intervals and p-values are shown.

strategy	cor	ci_lower	ci_upper	alternative	pvalue
no_resampling	0.561	0.538	0.584	two.sided	4.11e-278
resampling_before_clustering	-0.058	-0.095	-0.021	two.sided	2.37e-03
semi_resampling	0.017	-0.021	0.056	two.sided	3.76e-01
resampling_after_clustering	NA	NA	NA	two.sided	NA

3.3 Linear models

The predicted ratio of actives r_{pred} was modelled through the following quasibinomial generalized linear models, stratified by strategy:

$$r_{pred} \sim r_{training} + r_{test} + \log10(n_{int}) + \log10(n_{seq}) + k_{fold}$$

The main variables of interest are the actual ratios in the training $r_{training}$ and in test r_{test}, both numeric between 0 and 1. As additional covariates, the number of interactions n_{int} and the sequence length n_{seq} (numerical) and the fold number k_{fold} (categorical) were also included.

3.3.1 In semi_resampling or resampling_before_clustering

Due to the only slight correlation between training and test ratios (figure 2), the `semi_resampling` strategy is the **ideal scenario to disentangle their effects** on the predicted ratio of actives (see model in table 7). This additive model suggests:

- The **test ratio is driving the predicted proportions, rather than the training ratio**.
- `n_interactions`: **the term is not significant in GPCRs**.

Table 7 also shows the additive model for `resampling_before_clustering`. This strategy showed negative correlation between training and test ratios, also providing a reasonably good scenario to distinguish their effects.

- **This model confirms both conclusions from the model** in the `semi_resampling` strategy. **The ratio in training is also significant for GPCRs, but lower significance and estimate than the ratio in test; number of interactions slightly significant now.**

3.3.2 In no_resampling

The explanatory linear model under the `no_resampling` strategy (table 8) **suffers from the positive correlation between training and test ratios, which can be confounded**.

- Both `training_ratio` and `test_ratio` show a **positive effect** on the predicted fraction of actives.
- Although the **estimate is larger and more significant** for `training_ratio`, the **confounding effect and the very skewed distribution of the predicted ratios deems this model inconclusive**.

3.4 Conclusions

- Data imbalance exists in all strategies but in `resampling_after_clustering`, where balance is enforced.

Table 7: Linear models to describe the predicted active ratio for the semi_resampling and the resampling_before_clustering strategies. Significance and 95% confidence intervals are included.

	semi_resampling	resampling_before_clustering
	(1)	(2)
ratio_training	−0.106 (−1.147, 0.935)<br>p = 0.842	1.269 (0.613, 1.926)** p = 1.553e-04
ratio_test	1.093 (0.92, 1.266)***<br>p = 3.309e-34	1.456 (1.274, 1.637)***<br>p = 1.501e-53
log10(n_interactions)	−0.055 (−0.15, 0.04)<br>p = 0.259	−0.102 (−0.183, −0.021)*<br>p = 0.013
log10(len_seq)	−0.625 (−1.315, 0.066)<br>p = 0.076	−0.728 (−1.347, −0.109)*<br>p = 0.021
fold1	0.082 (−0.145, 0.309)<br>p = 0.477	−0.512 (−0.711, −0.313)***<br>p = 4.829e-07
fold2	−0.085 (−0.309, 0.139)<br>p = 0.455	0.219 (0.022, 0.417)*<br>p = 0.03
fold3	−0.145 (−0.371, 0.081)<br>p = 0.209	0.162 (−0.037, 0.362)<br>p = 0.11
fold4	−0.044 (−0.27, 0.183)<br>p = 0.706	0.073 (−0.125, 0.271)<br>p = 0.471
fold5	0.172 (−0.055, 0.4)<br>p = 0.138	−0.354 (−0.557, −0.152)**<br>p = 6.138e-04
fold6	−0.249 (−0.471, −0.027)*<br>p = 0.028	−0.106 (−0.307, 0.095)<br>p = 0.3
fold7	−0.732 (−0.949, −0.515)***<br>p = 4.372e-11	−1.350e-03 (−0.197, 0.195)<br>p = 0.989
fold8	−0.851 (−1.069, −0.633)***<br>p = 2.873e-14	−0.156 (−0.355, 0.042)<br>p = 0.123
fold9	−0.638 (−0.858, −0.419)***<br>p = 1.286e-08	0.157 (−0.041, 0.356)<br>p = 0.12
Constant	1.848 (−0.032, 3.727)<br>p = 0.054	0.961 (−0.685, 2.606)<br>p = 0.253
Observations	2620	2741
Note:		*p<0.05; **p<1.000e-03; ***p<1e-06

Table 8: Linear models to describe the predicted active ratio for the no_resampling strategy. Significance and 95% confidence intervals are included.

	no_resampling
ratio_training	6.695 (5.63, 7.76)***
	p = 3.913e-34
ratio_test	1.336 (0.663, 2.009)**
	p = 1.011e-04
log10(n_interactions)	−1.147 (−1.487, −0.806)***
	p = 4.808e-11
log10(len_seq)	3.459 (1.24, 5.679)*
	p = 2.273e-03
fold1	−1.024 (−1.928, −0.12)*
	p = 0.026
fold2	−0.455 (−1.381, 0.47)
	p = 0.335
fold3	−0.879 (−1.795, 0.037)
	p = 0.06
fold4	−0.221 (−1.209, 0.768)
	p = 0.662
fold5	−1.02 (−1.923, −0.118)*
	p = 0.027
fold6	−1.615 (−2.481, −0.749)**
	p = 2.620e-04
fold7	−0.879 (−1.791, 0.034)
	p = 0.059
fold8	−0.403 (−1.363, 0.557)
	p = 0.411
fold9	−0.469 (−1.392, 0.454)
	p = 0.319
Constant	−7.999 (−13.868, −2.13)*
	p = 7.593e-03
Observations	3359
Note:	*p<0.05; **p<1.000e-03; ***p<1e-06

- The correlation between a protein's ratio in train and test is positive in `no_resampling`, negative in `resampling_before_clustering` and null in `semi_resampling` and `resampling_after_clustering`.
- The main factor driving the ratio of actives in the model predictions, per protein, is the actual ratio of positives in the test set. Their distributions are resemblant, and linear models confirm the association.

All of them apply to GPCRs.

4 Description of baseline performance

Before evaluating the deep learning model, the performance metrics of the baselines were characterised, in order to pinpoint imbalance-sensitive and insensitive metrics. Metrics were called imbalance-sensitive if the imbalance-aware random baseline exhibited different performances between resampling strategies.

4.1 Descriptive plot

Figure 8 shows a fold-averaged picture of the metrics by protein. **Visual inspection suggested that accuracy, F1 and possibly balanced accuracy were affected by the data imbalance**. **F1 is the most apparent case**, see the quartiles in table 9.

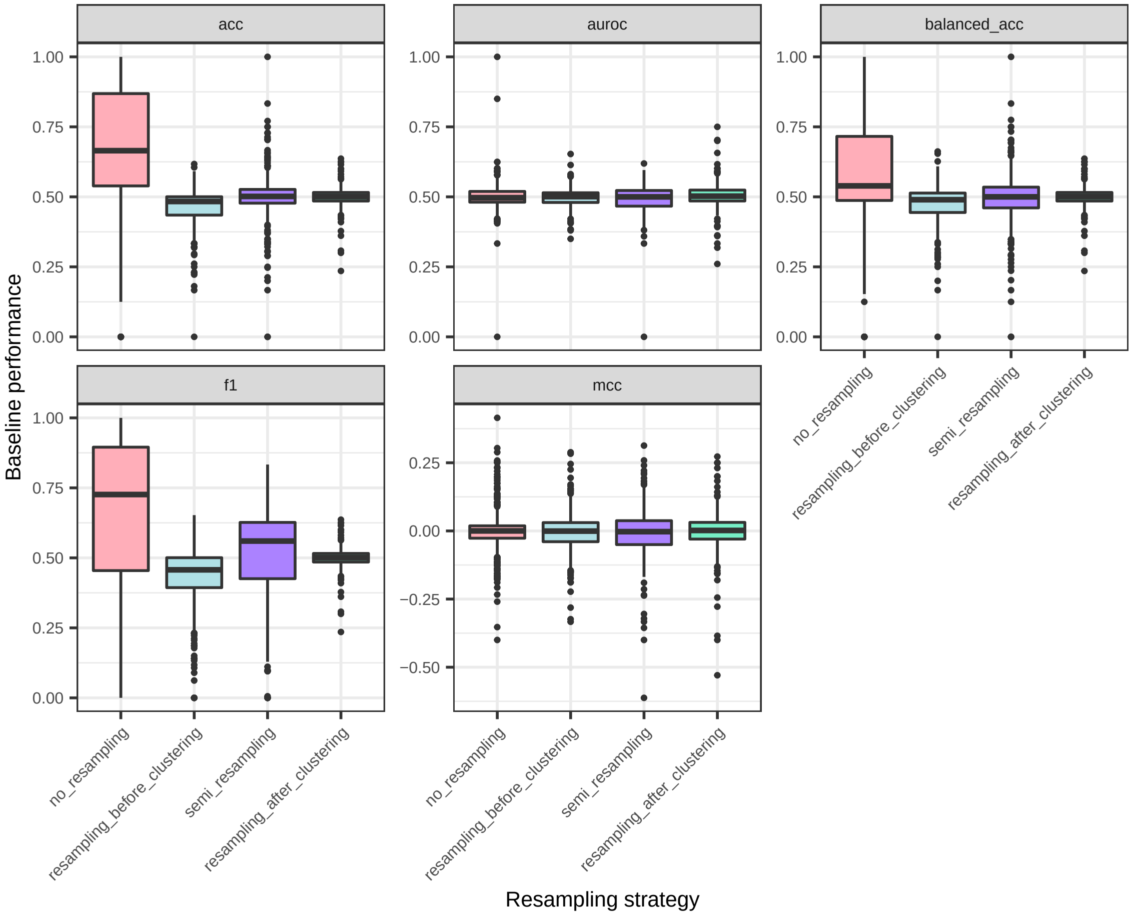

Figure 8: Performance metrics for imbalance-aware random baselines. Data points correspond to proteins, averaged over folds.

Table 9: Quartiles for the baseline F1-scores.

strategy	Min.	1st Qu.	Median	Mean	3rd Qu.	Max.
no_resampling	0.000	0.454	0.726	0.647	0.895	1.000
resampling_before_clustering	0.000	0.394	0.457	0.430	0.501	0.653
semi_resampling	0.000	0.426	0.560	0.506	0.627	0.833
resampling_after_clustering	0.235	0.485	0.501	0.498	0.516	0.636

4.2 Linear models

Formally, each performance metric was described with the following linear model:

$$\text{metric} \sim \text{strategy} + \log10(n_{int}) + \log10(n_{seq}) + k_{fold}$$

The response was the quantitative metric of interest (one model per metric), while `strategy` was categorical with the following possibilities: `no_resampling`, `resampling_after_clustering`, `resampling_before_clustering`, `semi_resampling`. Additional covariates included the number of interactions n_{int} and the sequence length n_{seq} (numerical) and the fold number k_{fold} (categorical). The `strategy` variable was tested with a type 3 ANOVA, being **significant** with $p < 0.05$ for `acc`, `f1` and `balanced_acc` (table 10).

Table 10: ANOVA p-values for including the resampling strategy as a regressor. Significant p-values imply that differences exist between resampling strategies.

strategy	variable	Sum Sq	Df	F value	Pr(>F)
acc	strategy	79.2893867	3	7.37e+02	0.00e+00
auroc	strategy	0.0060204	3	6.74e-02	9.77e-01
balanced_acc	strategy	15.7514288	3	1.28e+02	1.81e-81
f1	strategy	100.8229851	3	5.60e+02	0.00e+00
mcc	strategy	0.0338470	3	2.34e-01	8.73e-01

Based on this, metrics were divided in two types:

- Those where the baseline was different between strategies, i.e. imbalance-sensitive: `acc`, `f1` and `balanced_acc`. Therefore, before comparing strategies, the baseline performance needed to be accounted for.
- Those where the baseline was constant, i.e. imbalance-insensitive: `auroc`, `mcc`. Here we could compare strategies directly.

All applies to GPCRs as well.

5 Description of deep learning model performance

An overview of fold-averaged performances is displayed in figure 9, where strategies are paired with their baselines. This illustrates the **issue of direct strategy comparison** with imbalance-sensitive metrics, which was especially visible for the F1-score. Some metrics are undefined in edge cases (e.g. AUROC when only actives or only inactives are available); table 11 summarizes the number of proteins, added over folds, whose metrics were computable.

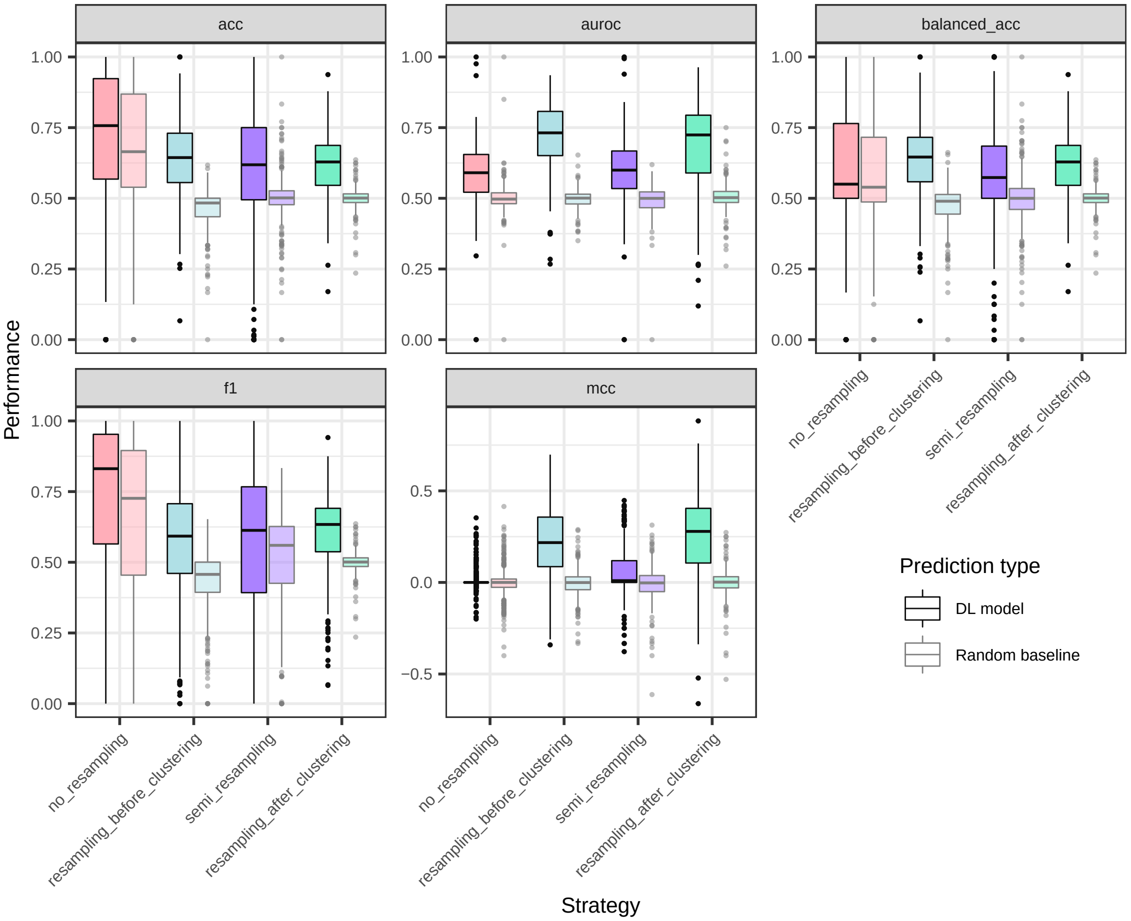

Figure 9: Performance metrics for balancing strategies and their corresponding imbalance-aware random baselines. Data points correspond to proteins, averaged over folds.

Table 11: Number of computable performance measures. AUROC was undefined for proteins with all actives or unactives in the test set, hence its lower counts.

strategy	acc	auroc	f1	balanced_acc	mcc
no_resampling	3414	2320	3414	3414	3414
resampling_before_clustering	2741	2314	2741	2741	2741
semi_resampling	3414	2320	3414	3414	3414
resampling_after_clustering	1203	1203	1203	1203	1203

5.1 Absolute, baseline-naive performance

Anologous to the baseline performance models, absolute metric models (not accounting for baselines) were fitted:

$$\text{metric} \sim \text{strategy} + \log10(n_{int}) + \log10(n_{seq}) + k_{fold}$$

The `strategy` covariate was **always significant** in a type 3 ANOVA (table 12). The models, summarized in 13, showed **different behaviour in imbalance-sensitive and insensitive metrics**. Pairwise comparisons of the strategy coefficients using Tukey's method would point to **two different pictures** (figure 10), further confirmed when prioritizing the strategies according to their expected performance through the linear models (table 14 and figure 11):

- Accuracy and F1-score suggested that `no_resampling` was the **best strategy**, but this was confounded by the fact that it also held the **highest baselines**.
- AUROC, MCC and balanced accuracy showed instead that `resampling_before_clustering` and `resampling_after_clustering` held the **highest performance estimates**.

Table 12: ANOVA p-values for including the resampling strategy as a regressor in the performance models.

strategy	variable	Sum Sq	Df	F value	Pr(>F)
acc	strategy	26.661749	3	1.26e+02	2.90e-80
auroc	strategy	21.408275	3	1.39e+02	1.40e-87
f1	strategy	61.491300	3	2.10e+02	2.20e-132
balanced_acc	strategy	7.868812	3	4.27e+01	2.08e-27
mcc	strategy	82.398075	3	4.78e+02	1.23e-291

Table 13: Linear models to describe each performance metric. Standard deviations in parentheses.

	acc	auroc	f1	balanced_acc	mcc
	(1)	(2)	(3)	(4)	(5)
strategyresampling_before_clustering	−0.096***	0.114***	−0.182***	0.051***	0.2***
	(6.883e-03)	(6.673e-03)	(8.100e-03)	(6.427e-03)	(6.214e-03)
	p = 4.956e-44	p = 8.545e-65	p = 0.000e+00	p = 1.253e-15	p = 0.000e+00
strategysemi_resampling	−0.112***	0.013*	−0.14***	−0.01	0.057***
	(6.426e-03)	(6.662e-03)	(7.562e-03)	(6.000e-03)	(5.801e-03)
	p = 3.801e-67	p = 0.048	p = 7.533e-76	p = 0.083	p = 1.271e-22
strategyresampling_after_clustering	−0.116***	0.101***	−0.171***	0.054***	0.232***
	(9.213e-03)	(8.193e-03)	(0.011)	(8.602e-03)	(8.317e-03)
	p = 3.303e-36	p = 1.520e-34	p = 1.286e-55	p = 4.246e-10	p = 0.000e+00
log10(n_interactions)	0.029***	0.046***	0.081***	−0.025***	0.054***
	(3.725e-03)	(4.573e-03)	(4.383e-03)	(3.478e-03)	(3.362e-03)
	p = 6.242e-15	p = 2.260e-23	p = 1.546e-75	p = 2.961e-13	p = 3.935e-58
log10(len_seq)	−0.084*	−0.054	−0.019	−0.088*	−0.019
	(0.033)	(0.033)	(0.039)	(0.031)	(0.03)
	p = 0.011	p = 0.108	p = 0.624	p = 4.142e-03	p = 0.519
fold1	0.014	0.012	−7.494e-03	3.971e-03	0.013
	(0.011)	(0.011)	(0.013)	(0.011)	(0.01)
	p = 0.203	p = 0.276	p = 0.573	p = 0.707	p = 0.195
fold2	0.015	0.021	9.361e-03	0.011	0.014
	(0.011)	(0.011)	(0.013)	(0.011)	(0.01)
	p = 0.193	p = 0.05	p = 0.479	p = 0.275	p = 0.176
fold3	0.011	5.610e-03	−5.323e-03	9.151e-03	2.785e-03
	(0.011)	(0.011)	(0.014)	(0.011)	(0.01)
	p = 0.329	p = 0.617	p = 0.694	p = 0.393	p = 0.788
fold4	0.034*	3.539e-03	0.017	0.028*	0.014
	(0.011)	(0.011)	(0.014)	(0.011)	(0.01)
	p = 3.126e-03	p = 0.754	p = 0.206	p = 8.064e-03	p = 0.163
fold5	0.016	−1.805e-03	−0.011	9.616e-03	−3.018e-03
	(0.011)	(0.011)	(0.014)	(0.011)	(0.01)
	p = 0.171	p = 0.871	p = 0.437	p = 0.37	p = 0.771
fold6	0.017	0.014	−0.019	0.014	0.021*
	(0.011)	(0.011)	(0.013)	(0.011)	(0.01)
	p = 0.128	p = 0.199	p = 0.146	p = 0.183	p = 0.044
fold7	2.239e-03	0.011	−0.025	8.648e-03	0.019
	(0.011)	(0.011)	(0.013)	(0.011)	(0.01)
	p = 0.844	p = 0.301	p = 0.057	p = 0.415	p = 0.071
fold8	0.015	0.024*	−0.025	0.015	0.026*
	(0.011)	(0.011)	(0.013)	(0.011)	(0.01)
	p = 0.19	p = 0.03	p = 0.059	p = 0.159	p = 0.012
fold9	−4.569e-04	0.014	−0.033*	5.402e-03	0.01
	(0.011)	(0.011)	(0.013)	(0.011)	(0.01)
	p = 0.968	p = 0.198	p = 0.013	p = 0.609	p = 0.323
Constant	0.868***	0.596***	0.609***	0.868***	−0.072
	(0.085)	(0.087)	(0.101)	(0.08)	(0.077)
	p = 3.968e-24	p = 8.565e-12	p = 1.434e-09	p = 2.065e-27	p = 0.352
Observations	10772	8157	10772	10772	10772
R^2	0.039	0.067	0.076	0.015	0.169
Adjusted R^2	0.037	0.065	0.075	0.014	0.168

Note: *p<0.05; **p<1.000e-03; ***p<1e-06

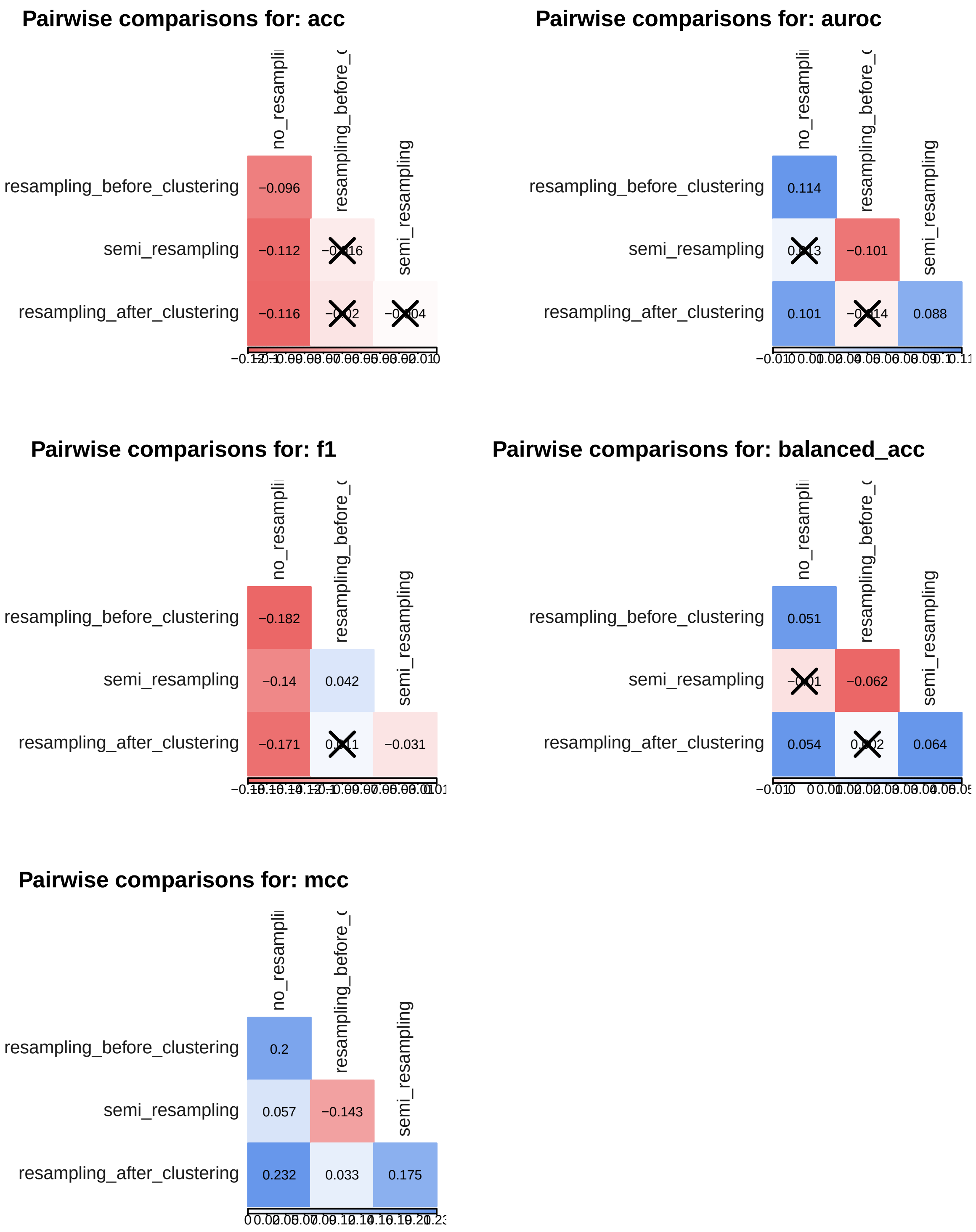

Figure 10: Pairwise comparison of strategy performance using Tukey method.

Table 14: Expected absolute performances, by metric and strategy, with 95% confidence intervals.

metric	strategy	emmean	SE	lower.CL	upper.CL
acc	no_resampling	0.746	5.030e-03	0.736	0.755
acc	resampling_before_clustering	0.649	5.202e-03	0.639	0.659
acc	semi_resampling	0.633	5.030e-03	0.624	0.643
acc	resampling_after_clustering	0.629	7.660e-03	0.614	0.644
auroc	no_resampling	0.597	4.861e-03	0.588	0.607
auroc	resampling_before_clustering	0.711	4.831e-03	0.702	0.721
auroc	semi_resampling	0.610	4.861e-03	0.601	0.620
auroc	resampling_after_clustering	0.698	6.550e-03	0.685	0.711
f1	no_resampling	0.780	5.919e-03	0.769	0.792
f1	resampling_before_clustering	0.598	6.121e-03	0.586	0.610
f1	semi_resampling	0.640	5.919e-03	0.628	0.652
f1	resampling_after_clustering	0.609	9.013e-03	0.591	0.627
balanced_acc	no_resampling	0.579	4.697e-03	0.569	0.588
balanced_acc	resampling_before_clustering	0.630	4.857e-03	0.621	0.640
balanced_acc	semi_resampling	0.568	4.697e-03	0.559	0.577
balanced_acc	resampling_after_clustering	0.632	7.152e-03	0.618	0.646
mcc	no_resampling	0.044	4.541e-03	0.035	0.053
mcc	resampling_before_clustering	0.244	4.696e-03	0.235	0.253
mcc	semi_resampling	0.101	4.541e-03	0.092	0.110
mcc	resampling_after_clustering	0.277	6.915e-03	0.263	0.290

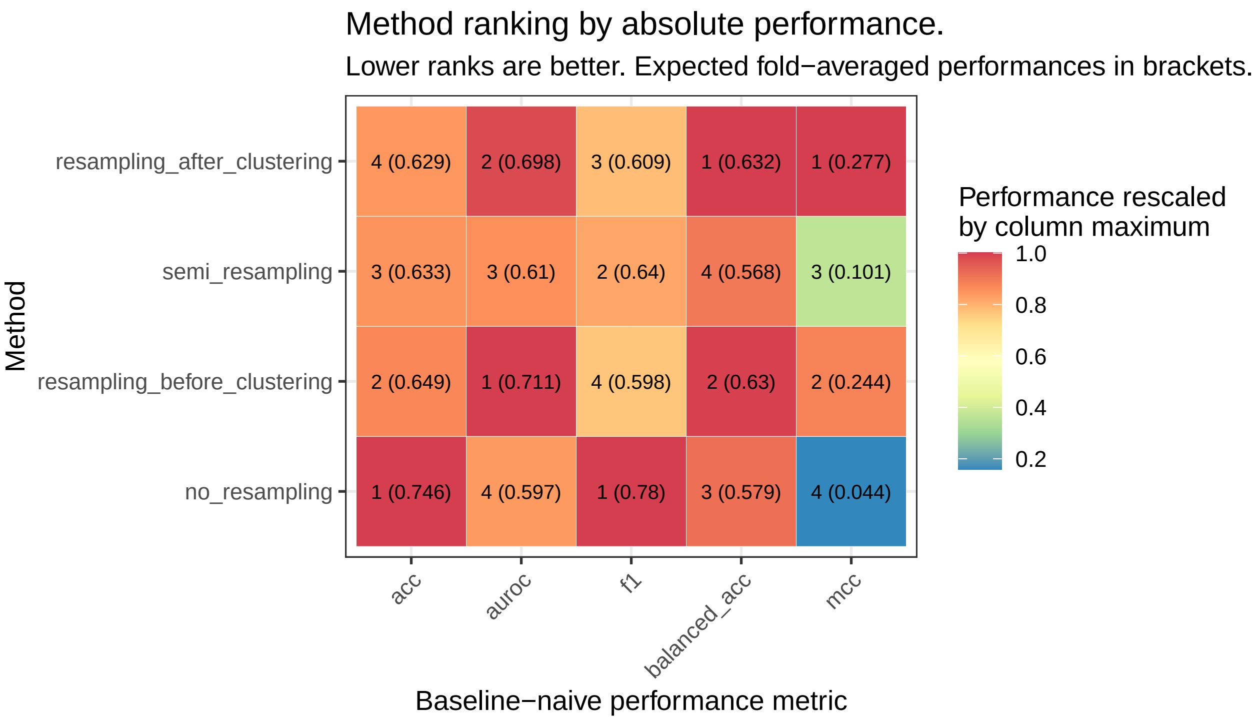

Figure 11: Method ranking according to the linear model predicted performances for each metric. Baseline metrics were ignored.

5.2 Baseline-adjusted performance

To address the pitfalls of the direct comparison of metrics whose baselines may differ, baseline-adjusted performance metrics were defined and modelled analogously. Specifically:

$$\text{adj\_metric} = \text{metric} - \text{baseline}$$

A descriptive plot of the the adjusted metrics (figure 12) pointed to a **scenario diffent than that of unadjusted ones** (figure 9).

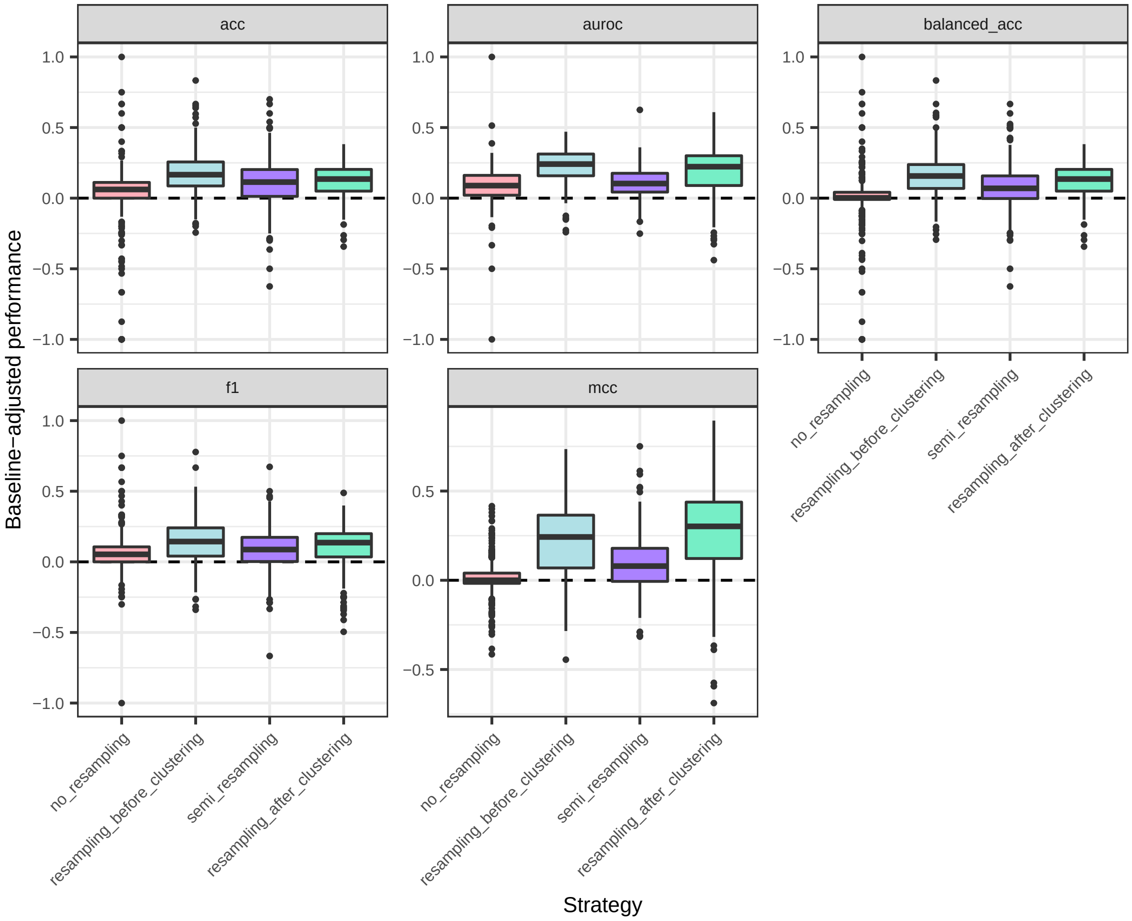

Figure 12: Baseline-adjusted performance metrics for balancing strategies. Data points correspond to proteins, averaged over folds.

Adjusted performance metrics were described with the following linear model:

$$\text{adj\_metric} \sim \text{strategy} + \log10(n_{int}) + \log10(n_{seq}) + k_{fold}$$

Note that while all metrics but `mcc` were non-negative, the adjusted metrics could show negative values when the performance of the DL model was lower than that of the baseline.

Again, `strategy` covariate was **always significant** in a type 3 ANOVA (table 15). **Baseline adjustment brought a uniform behaviour across the models** (table 16), further confirmed in pairwise coefficient comparison (Tukey's method, figure 13) and in their expected performance (table 17 and figure 14):

- `resampling_before_clustering` and `resampling_after_clustering` had the **highest performance estimates, followed by** `semi_resampling` and finally by `no_resampling`.
- This **picture was analogous to that of the non-adjusted performance metrics that were imbalance-insensitive** (AUROC, MCC).

Table 15: ANOVA p-values for including the resampling strategy as a regressor in the adjusted performance models.

strategy	variable	Sum Sq	Df	F value	Pr(>F)
acc	strategy	18.137218	3	8.88e+01	1.12e-56
auroc	strategy	21.118128	3	8.83e+01	3.59e-56
f1	strategy	6.556575	3	3.89e+01	5.40e-25
balanced_acc	strategy	34.369289	3	1.72e+02	1.50e-108
mcc	strategy	83.131344	3	2.58e+02	6.13e-161

Conclusions drawn from the baseline-adjusted performance analysis:

- The largest impact in performance estimates was the application of **data augmentation to the test set**: `resampling_before_clustering` and `resampling_after_clustering` tended to outperform `semi_resampling` and `no_resampling` (Tukey's method, $p < 0.05$, figure 13). However, augmenting the test set might not faithfully reflect new data anymore, and could **artificially inflate the peformance estimates**.
- `semi_resampling` outperformed `no_resampling` in **three out of five, being the other two non-significant** (Tukey's method, $p < 0.05$, figure 13), which **supports data augmentation usefulness** even if the data balance in the test set differs from that of the training set. This was **consistent with the observation that the predicted proportion of positives of the PCM model was mainly driven by the actual data balance in the test set, rather than that of the training set. Combined with the healthier distributions of predicted active ratios** of `semi_resampling` against `no_resampling`, this made a **case in favour of the former**.
- In **five** out of five metrics, **proteins with more interactions were better predicted** (table 16).

Table 16: Linear models to describe each adjusted performance metric. Standard deviations in parentheses.

	acc	auroc	f1	balanced_acc	mcc
	(1)	(2)	(3)	(4)	(5)
strategyresampling_before_clustering	0.111^{***}	0.114^{***}	0.063^{***}	0.148^{***}	0.202^{***}
	(6.797e-03)	(8.306e-03)	(6.172e-03)	(6.726e-03)	(8.543e-03)
	p = 4.459e-59	p = 1.574e-42	p = 1.135e-24	p = 0.000e+00	p = 0.000e+00
strategysemi_resampling	0.053^{***}	0.011	0.015^{*}	0.068^{***}	0.061^{***}
	(6.905e-03)	(8.479e-03)	(6.270e-03)	(6.833e-03)	(8.678e-03)
	p = 1.885e-14	p = 0.188	p = 0.019	p = 5.502e-23	p = 1.966e-12
strategyresampling_after_clustering	0.057^{***}	0.098^{***}	0.042^{***}	0.12^{***}	0.235^{***}
	(9.236e-03)	(0.01)	(8.387e-03)	(9.140e-03)	(0.012)
	p = 6.266e-10	p = 2.754e-21	p = 6.864e-07	p = 3.564e-39	p = 1.186e-89
log10(n_interactions)	0.04^{***}	0.038^{***}	0.014^{**}	0.014^{*}	0.052^{***}
	(4.375e-03)	(6.018e-03)	(3.972e-03)	(4.329e-03)	(5.498e-03)
	p = 5.057e-20	p = 1.732e-10	p = 3.523e-04	p = 1.485e-03	p = 2.617e-21
log10(len_seq)	-0.091^{*}	−0.034	−1.940e-03	-0.093^{*}	−0.027
	(0.035)	(0.042)	(0.031)	(0.034)	(0.043)
	p = 8.579e-03	p = 0.419	p = 0.951	p = 6.524e-03	p = 0.541
fold1	0.024^{*}	2.277e-03	−0.011	0.014	0.012
	(0.012)	(0.014)	(0.011)	(0.011)	(0.015)
	p = 0.036	p = 0.869	p = 0.297	p = 0.215	p = 0.43
fold2	7.466e-03	0.016	9.307e-03	5.819e-03	0.012
	(0.012)	(0.014)	(0.011)	(0.011)	(0.015)
	p = 0.52	p = 0.26	p = 0.377	p = 0.612	p = 0.401
fold3	0.012	−6.461e-03	−6.538e-03	8.988e-03	−3.534e-03
	(0.012)	(0.014)	(0.011)	(0.012)	(0.015)
	p = 0.322	p = 0.648	p = 0.541	p = 0.441	p = 0.811
fold4	0.015	−8.950e-03	8.932e-03	0.014	3.821e-03
	(0.012)	(0.014)	(0.011)	(0.012)	(0.015)
	p = 0.2	p = 0.53	p = 0.404	p = 0.244	p = 0.797
fold5	6.087e-03	−0.012	−0.018	4.073e-03	−0.012
	(0.012)	(0.014)	(0.011)	(0.012)	(0.015)
	p = 0.604	p = 0.398	p = 0.087	p = 0.726	p = 0.426
fold6	0.017	3.990e-04	−0.017	0.014	0.011
	(0.012)	(0.014)	(0.011)	(0.012)	(0.015)
	p = 0.15	p = 0.977	p = 0.108	p = 0.239	p = 0.474
fold7	9.271e-04	3.367e-03	-0.026^{*}	8.568e-03	0.01
	(0.012)	(0.014)	(0.011)	(0.012)	(0.015)
	p = 0.936	p = 0.809	p = 0.015	p = 0.456	p = 0.484
fold8	0.016	0.011	-0.029^{*}	0.017	0.018
	(0.012)	(0.014)	(0.011)	(0.012)	(0.015)
	p = 0.176	p = 0.424	p = 6.887e-03	p = 0.131	p = 0.213
fold9	7.169e-03	0.011	−0.016	9.026e-03	0.011
	(0.012)	(0.014)	(0.011)	(0.012)	(0.015)
	p = 0.538	p = 0.435	p = 0.131	p = 0.433	p = 0.47
Constant	0.184^{*}	0.077	0.049	0.205^{*}	−0.041
	(0.09)	(0.111)	(0.082)	(0.089)	(0.114)
	p = 0.041	p = 0.486	p = 0.552	p = 0.022	p = 0.719
Observations	9902	7958	9902	9902	9902
R^2	0.042	0.04	0.017	0.056	0.096
Adjusted R^2	0.04	0.039	0.016	0.055	0.095

Note: $^{*}p<0.05$; $^{**}p<1.000e-03$; $^{***}p<1e-06$

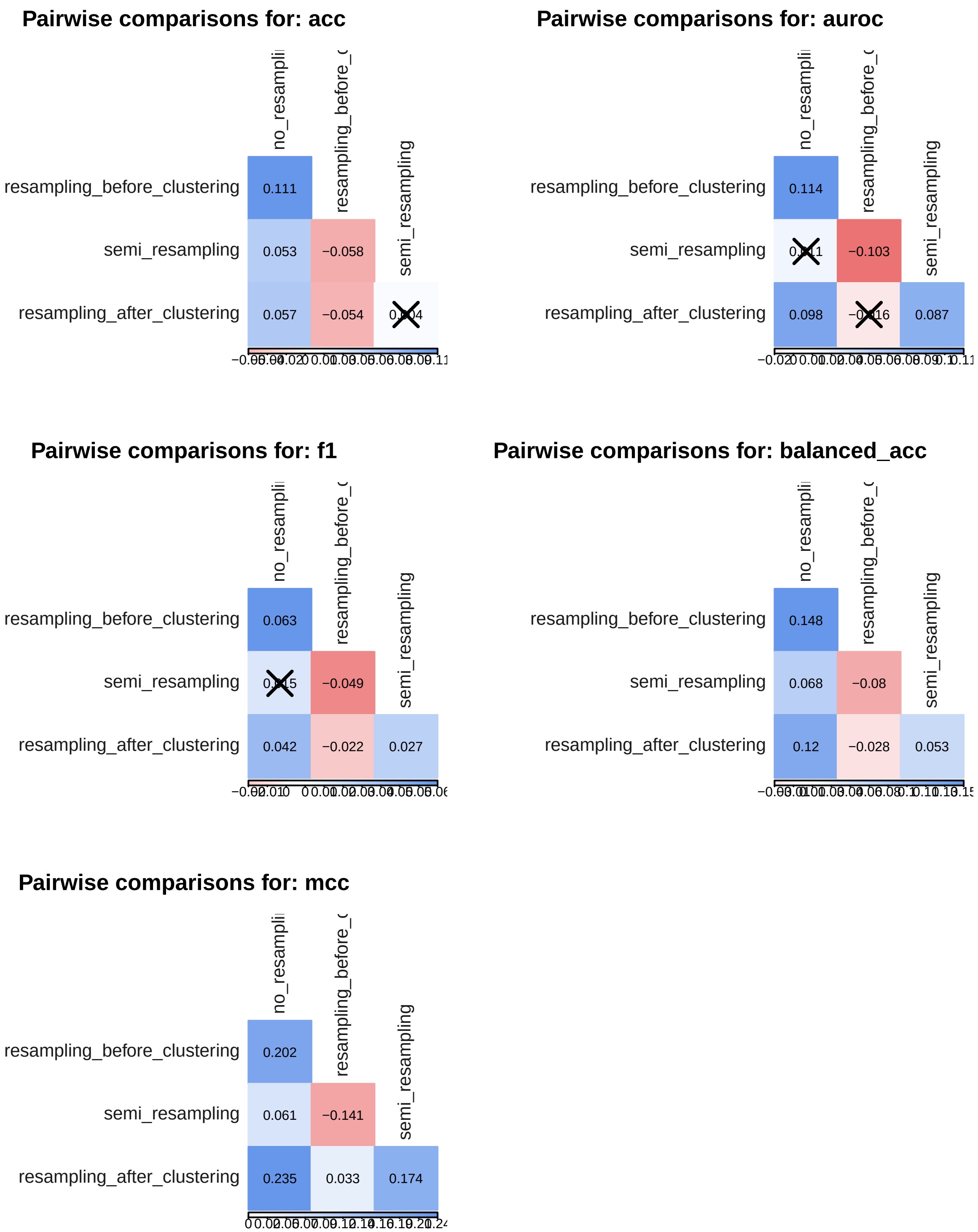

Figure 13: Pairwise comparison of strategy adjusted performance using Tukey method.

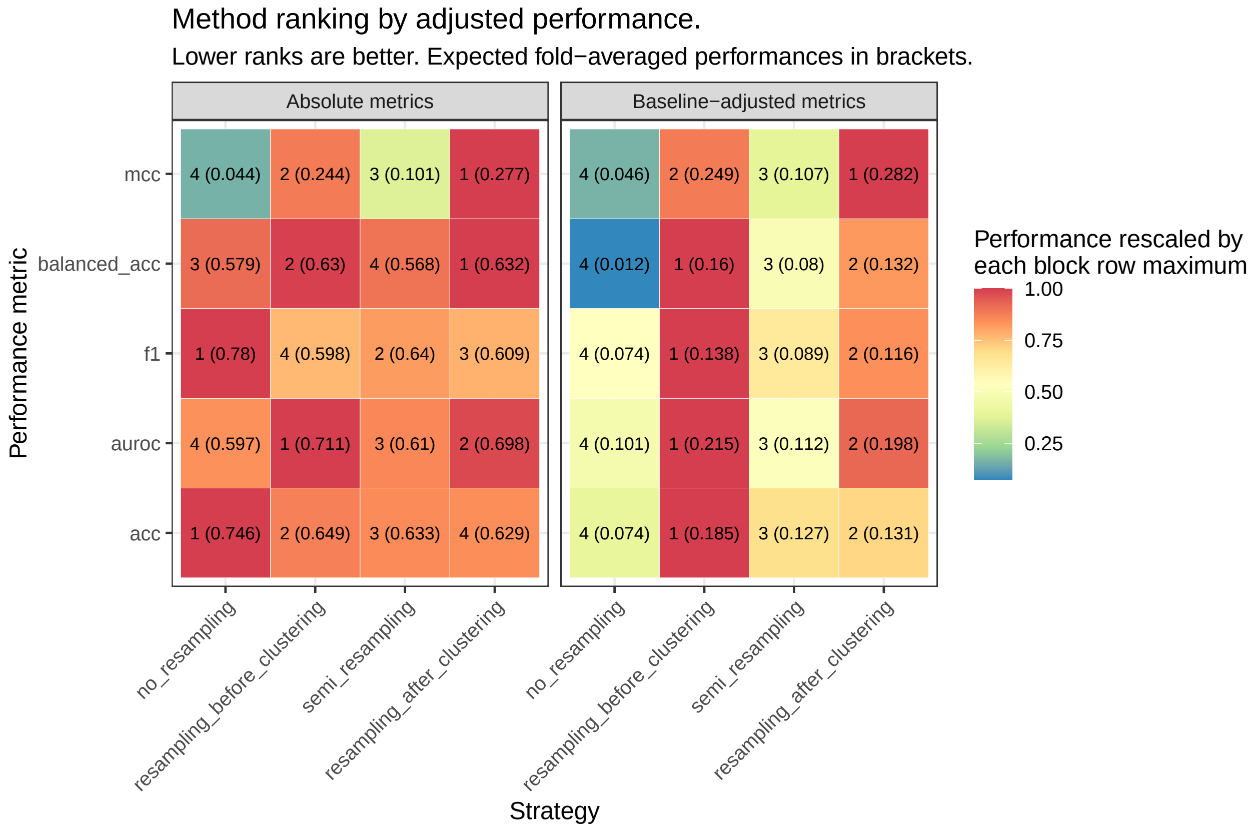

Figure 14: Method ranking according to the linear model predicted adjusted performances for each metric. Baseline metrics were taken into account in the adjustment. For a direct comparison, the same ranking using the absolute metrics was kept side by side.

Table 17: Expected adjusted performances, by metric and strategy, with 95% confidence intervals.

metric	strategy	emmean	SE	lower.CL	upper.CL
acc	no_resampling	0.074	5.172e-03	0.064	0.084
acc	resampling_before_clustering	0.185	5.210e-03	0.175	0.195
acc	semi_resampling	0.127	5.278e-03	0.117	0.137
acc	resampling_after_clustering	0.131	7.595e-03	0.116	0.146
auroc	no_resampling	0.101	6.130e-03	0.089	0.113
auroc	resampling_before_clustering	0.215	6.079e-03	0.203	0.227
auroc	semi_resampling	0.112	6.232e-03	0.100	0.124
auroc	resampling_after_clustering	0.198	8.222e-03	0.182	0.214
f1	no_resampling	0.074	4.696e-03	0.065	0.083
f1	resampling_before_clustering	0.138	4.731e-03	0.128	0.147
f1	semi_resampling	0.089	4.792e-03	0.079	0.098
f1	resampling_after_clustering	0.116	6.897e-03	0.102	0.129
balanced_acc	no_resampling	0.012	5.118e-03	0.002	0.022
balanced_acc	resampling_before_clustering	0.160	5.156e-03	0.150	0.170
balanced_acc	semi_resampling	0.080	5.223e-03	0.069	0.090
balanced_acc	resampling_after_clustering	0.132	7.516e-03	0.117	0.147
mcc	no_resampling	0.046	6.500e-03	0.033	0.059
mcc	resampling_before_clustering	0.249	6.548e-03	0.236	0.261
mcc	semi_resampling	0.107	6.633e-03	0.094	0.120
mcc	resampling_after_clustering	0.282	9.546e-03	0.263	0.300

6 Reproducibility

- R version 3.6.3 (2020-02-29), `x86_64-pc-linux-gnu`
- Locale: `LC_CTYPE=en_US.UTF-8`, `LC_NUMERIC=C`, `LC_TIME=es_ES.UTF-8`, `LC_COLLATE=en_US.UTF-8`, `LC_MONETARY=es_ES.UTF-8`, `LC_MESSAGES=en_US.UTF-8`, `LC_PAPER=es_ES.UTF-8`, `LC_NAME=C`, `LC_ADDRESS=C`, `LC_TELEPHONE=C`, `LC_MEASUREMENT=es_ES.UTF-8`, `LC_IDENTIFICATION=C`
- Running under: `Ubuntu 16.04.7 LTS`
- Matrix products: default
- BLAS: `/usr/lib/atlas-base/atlas/libblas.so.3.0`
- LAPACK: `/usr/lib/atlas-base/atlas/liblapack.so.3.0`
- Base packages: base, datasets, graphics, grDevices, methods, stats, utils
- Other packages: corrplot 0.84, dplyr 1.0.5, forcats 0.5.1, ggplot2 3.3.3, gsubfn 0.7, kableExtra 1.3.4, magrittr 2.0.1, proto 1.0.0, purrr 0.3.4, readr 1.4.0, stargazer 5.2.2, stringr 1.4.0, tibble 3.1.0, tidyr 1.1.3, tidyverse 1.3.0
- Loaded via a namespace (and not attached): abind 1.4-5, assertthat 0.2.1, backports 1.2.1, bookdown 0.21, broom 0.7.5, car 3.0-10, carData 3.0-4, cellranger 1.1.0, cli 2.3.1, codetools 0.2-16, colorspace 2.0-0, compiler 3.6.3, crayon 1.4.1, curl 4.3, data.table 1.14.0, DBI 1.1.1, dbplyr 2.1.0, digest 0.6.27, ellipsis 0.3.1, emmeans 1.5.4, estimability 1.3, evaluate 0.14, fansi 0.4.2, farver 2.1.0, foreign 0.8-76, fs 1.5.0, generics 0.1.0, glue 1.4.2, grid 3.6.3, gtable 0.3.0, haven 2.3.1, highr 0.8, hms 1.0.0, htmltools 0.5.1.1, httr 1.4.2, jsonlite 1.7.2, knitr 1.31, labeling 0.4.2, lattice 0.20-41, lifecycle 1.0.0, lubridate 1.7.10, MASS 7.3-53, Matrix 1.2-18, mgcv 1.8-33, modelr 0.1.8, multcomp 1.4-16, munsell 0.5.0, mvtnorm 1.1-1, nlme 3.1-149, openxlsx 4.2.3, pillar 1.5.1,

pkgconfig 2.0.3, plyr 1.8.6, R6 2.5.0, RColorBrewer 1.1-2, Rcpp 1.0.6, readxl 1.3.1, reprex 1.0.0, reshape2 1.4.4, rio 0.5.26, rlang 0.4.10, rmarkdown 2.7, rstudioapi 0.13, rvest 0.3.6, sandwich 3.0-0, scales 1.1.1, splines 3.6.3, stringi 1.5.3, survival 3.2-7, svglite 2.0.0, systemfonts 1.0.1, tcltk 3.6.3, TH.data 1.0-10, tidyselect 1.1.0, tools 3.6.3, utf8 1.1.4, vctrs 0.3.6, viridisLite 0.3.0, webshot 0.5.2, withr 2.4.1, xfun 0.21, xml2 1.3.2, xtable 1.8-4, yaml 2.2.1, zip 2.1.1, zoo 1.8-8

Appendix 4: model predictions and performance (NRs)

Angela Lopez-del Rio   Sergio Picart-Armada   Alexandre Perera-Lluna

27/02/2021

Contents

1 Overview 1

2 Description of data balance 2
2.1 Distributions of the actives ratio . . . 2
2.2 Comparing training and test imbalance . . . 2
2.3 Other covariates . . . 2

3 Linear models on predicted proportions 5
3.1 Distributions of the predicted ratios . . . 5
3.2 Predicted ratios against training ratios . . . 6
3.3 Linear models . . . 8
3.4 Conclusions . . . 11

4 Description of baseline performance 11
4.1 Descriptive plot . . . 11
4.2 Linear models . . . 11

5 Description of deep learning model performance 13
5.1 Absolute, baseline-naive performance . . . 13
5.2 Baseline-adjusted performance . . . 19

6 Reproducibility 24

1 Overview

This supplement describes the behaviour of the proteochemometrics (PCM) deep learning model to predict protein-compound bioactivity for **NRs**. Specifically, this includes the descriptive statistics of data imbalance: the proportion of actives per protein in the training and test sets during the model fitting and the predicted proportion of actives. The model performance per protein was also described, pinpointing the most influential factors and characterising the proteins with the most extreme performances.

Four strategies (no_resampling, resampling_before_clustering, semi_resampling, resampling_after_clustering) were considered. For each of those, 10 folds of repeated holdout were run, and 5 performance metrics were computed: acc, auroc, f1, balanced_acc, mcc. This led to a total of 1808 values of performance. Since some strategies involved the upsampling method SMOTE, proteins whose sample sizes did not allow upsampling were excluded (table 1).

Table 1: Number of proteins for which performance metrics were computed. The resampling after clustering was the most stringent strategy regarding eligible proteins, since the resampling was carried out after the clustering, which introduced more imbalance.

Strategy	Fold 0	Fold 1	Fold 2	Fold 3	Fold 4	Fold 5	Fold 6	Fold 7	Fold 8	Fold 9
no_resampling	56	69	51	65	55	44	68	56	51	67
resampling_before_clustering	44	46	48	47	40	46	45	45	46	46
semi_resampling	56	69	51	65	55	44	68	56	51	67
resampling_after_clustering	19	20	19	25	16	17	20	14	21	20

2 Description of data balance

The data balancing strategy had an impact on the actual data balance, defined as the proportion of active molecules for a protein. Furthermore, specific trends were observed in the original data in the training and test sets, as well as in the values predicted by the deep learning model.

2.1 Distributions of the actives ratio

The histograms in figure 1 revealed trends:

- `no_resampling` **keeps similar data imbalance in training and test.**
- `resampling_before_clustering` and `semi_resampling` **lead to a more balanced training set, but not so much for the test set.**
- `resampling_after_clustering` **kept balanced proteins** in both training and test sets.

In addition, **test sets with imbalance tended to magnify** it and create extreme cases (all actives or all inactives), probably due to the combination of the clustering and the lower sample sizes in the test sets compared to training.

2.2 Comparing training and test imbalance

Figure 2 revealed both positive, negative and null trends between the training and test set protein balances.

- `no_resampling` showed a **positive relation** between both, i.e. proteins were prone to keep their (im)balance in train and test.
- `resampling_before_clustering` showed an **inverse relationship** instead. This was expected since this strategy started from globally balanced proteins, and after the clustering, an imbalance in one direction in the training set entailed an inverse imbalance in the test set.
- `semi_resampling` led to **independent train and test balances, expected since the train set was resampled, breaking any correlation with the test set balance.**
- `resampling_after_clustering` always **kept balanced proteins**, by design.

Table 2 displays the Pearson correlation estimate, 95% confidence interval and p-value for each strategy (except `resampling_after_clustering`, where ratios are constant), further confirming the claims above.

2.3 Other covariates

The effect of the number of interactions of each protein in its corresponding set and fold (figure 3) and the protein length in amino acids (figure 4) on the test set imbalance was investigated:

- **Proteins with greatest imbalance** (i.e. where `(0.5 - ratio_test)^2` was greatest) **tended to be among those with the least interactions. Linear correlations were significant** (table 3).

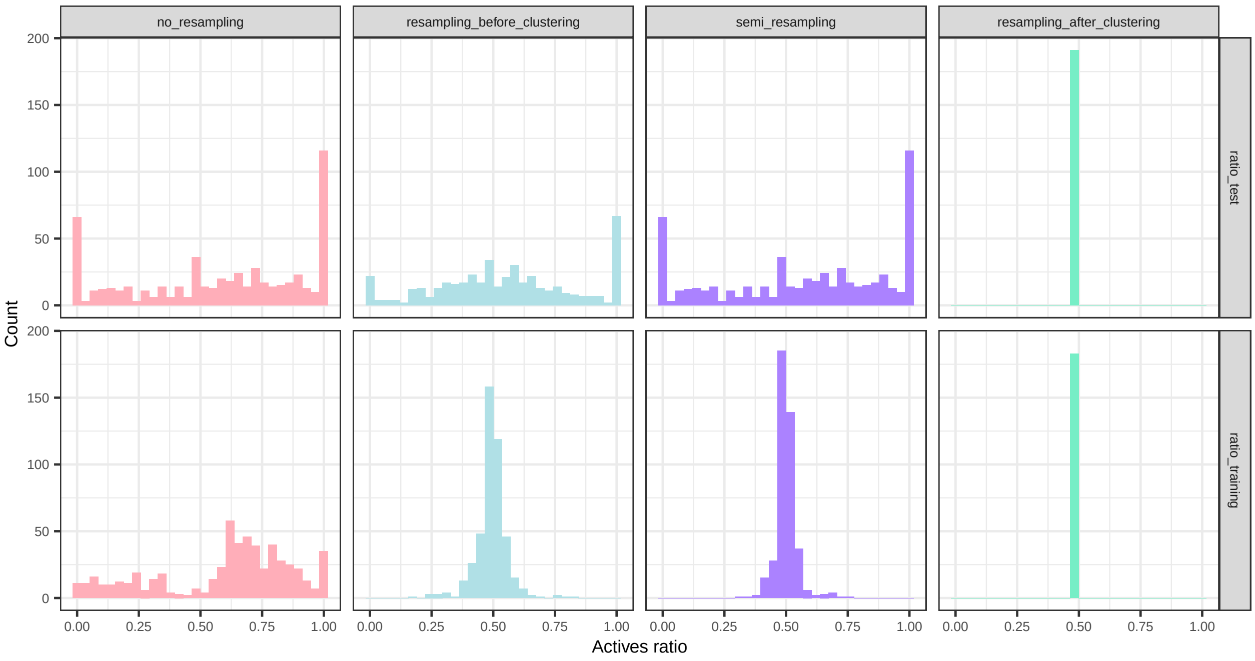

Figure 1: Distributios of the actives ratio in the training set and in the test set (both original and predicted by the deep learning model).

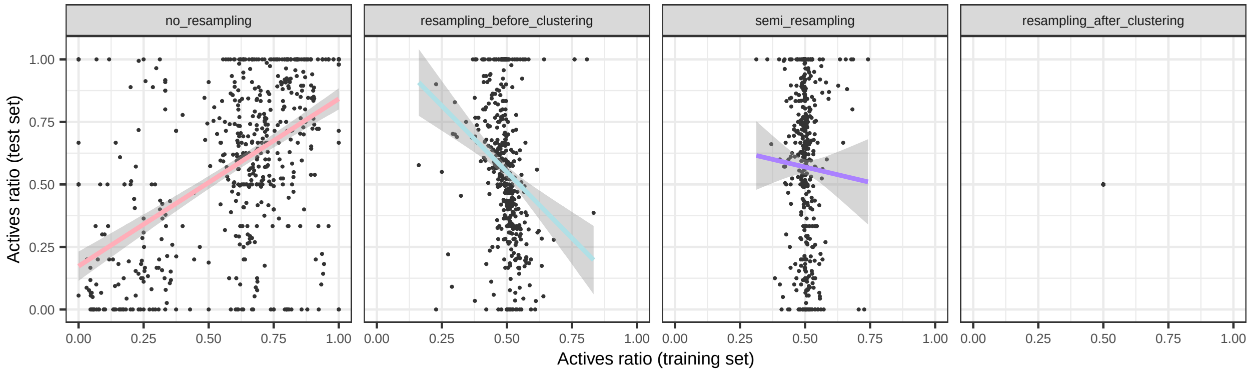

Figure 2: Comparison of the training and test ratios, by resampling strategy. A linear fit line was added per strategy.

Table 2: Correlations between train and test active ratios. 95% confidence intervals and p-values are shown.

strategy	cor	ci_lower	ci_upper	alternative	pvalue
no_resampling	0.531	0.470	0.588	two.sided	6.53e-43
resampling_before_clustering	-0.241	-0.326	-0.152	two.sided	2.16e-07
semi_resampling	-0.033	-0.128	0.062	two.sided	4.92e-01
resampling_after_clustering	NA	NA	NA	two.sided	NA

- **The sequence length had no obvious effect on the protein imbalance. Linear correlations were not significant** (`resampling_before_clustering`) or significant but low (`no_resampling`, `semi_resampling`).

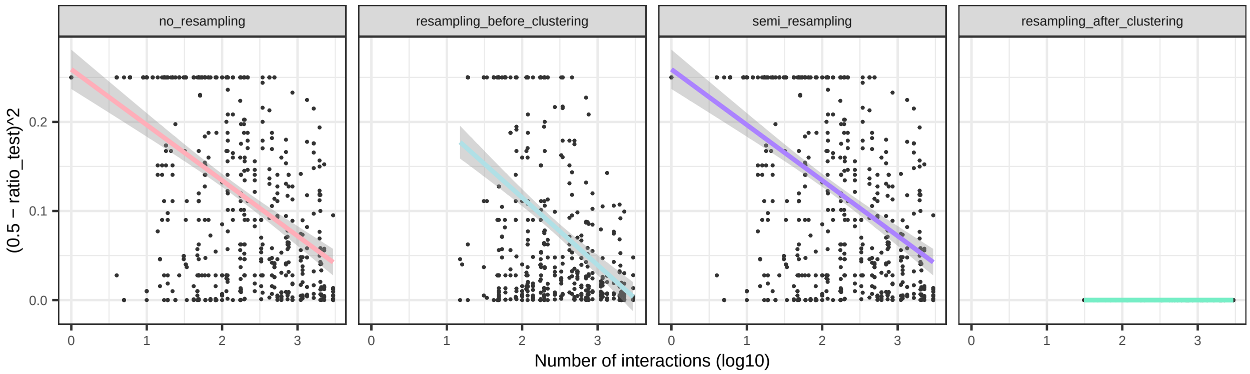

Figure 3: Data imbalance in the test set as a function of the number of available interactions for each protein.

Table 3: Correlations between imbalance (as defined above) and number of interactions. 95% confidence intervals and p-values are shown.

strategy	cor	ci_lower	ci_upper	alternative	pvalue
no_resampling	-0.396	-0.462	-0.325	two.sided	2.89e-23
resampling_before_clustering	-0.395	-0.470	-0.314	two.sided	2.28e-18
semi_resampling	-0.396	-0.462	-0.325	two.sided	2.89e-23
resampling_after_clustering	NA	NA	NA	two.sided	NA

Table 4: Correlations between imbalance (as defined above) and sequence length. 95% confidence intervals and p-values are shown.

strategy	cor	ci_lower	ci_upper	alternative	pvalue
no_resampling	-0.091	-0.171	-0.010	two.sided	2.81e-02
resampling_before_clustering	-0.030	-0.122	0.062	two.sided	5.18e-01
semi_resampling	-0.091	-0.171	-0.010	two.sided	2.81e-02
resampling_after_clustering	NA	NA	NA	two.sided	NA

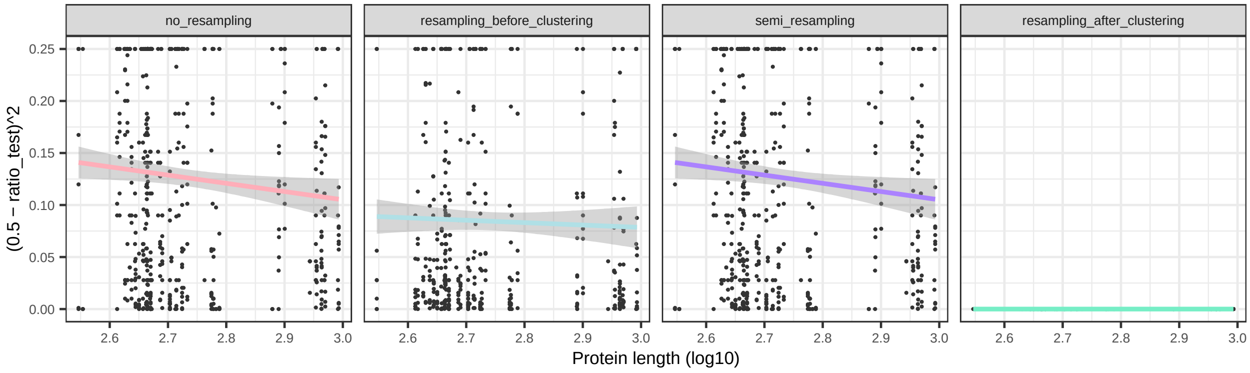

Figure 4: Data imbalance in the test set as a function of the protein length, in amino acids.

3 Linear models on predicted proportions

The next key question was to narrow down the factor driving the predicted proportion of actives. The main options under consideration were:

1. A constant, global imbalance that the model would learn from the whole dataset.
2. The protein-wise imbalance that the model would learn in the training set.
3. A test set-driven imbalance, based on its actual imbalance.

3.1 Distributions of the predicted ratios

After the model predictions in the test set were binarized (actives were those whose probabilities exceeded 0.5), the ratio of predicted actives was computed by protein. This ratio, shown in figure 5, suggested that:

- `no_resampling` was noticeably **biased to predict everything as positives**.
- `resampling_before_clustering` and `semi_resampling` **alleviated the imbalance in the predictions, but still retained a spike of proteins where all the compounds were predicted as positives**.
- `resampling_after_clustering` **kept a wide and symmetric distribution of predicted actives**.

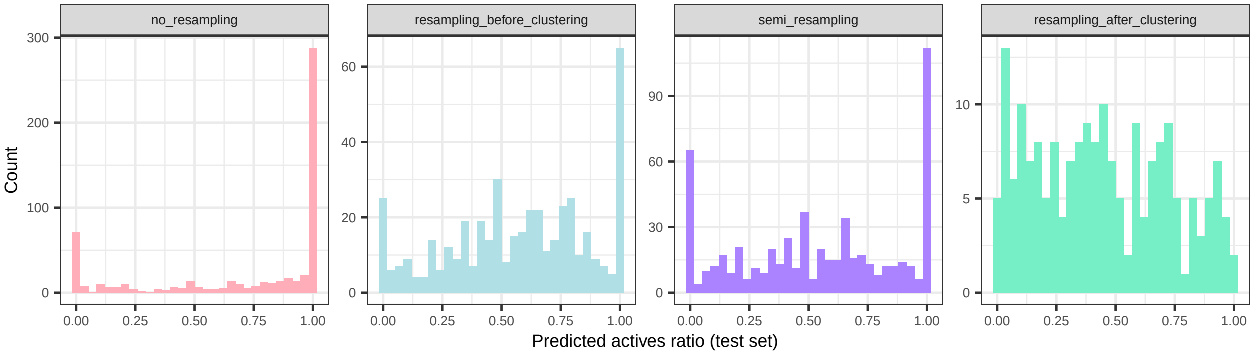

Figure 5: Ratios of the prediction values, after binarization.

Now, representing together (1) the original training and test ratios, and (2) the predicted ratios in test (figure 6) eased a general qualitative assessment: **the distribution was most resemblant to that of the test proportions to that of the training ones** (except `resampling_after_clustering`, since those

proportions are constant). Table 5 displays how `no_resampling` was **highly inclined to predict all positives**, `resampling_before_clustering` and `semi_resampling` **alleviated this phenomenon**, and `resampling_after_clustering` was **essentially balanced**.

Table 5: Percentage of extreme cases, i.e. proteins with all actives or inactives.

strategy	RatioSet	all_inactives	all_actives	all_extremes
no_resampling	ratio_test	11.3	19.6	30.9
no_resampling	ratio_test_predicted	12.2	47.6	59.8
no_resampling	ratio_training	1.8	6.1	7.9
resampling_before_clustering	ratio_test	4.9	14.8	19.6
resampling_before_clustering	ratio_test_predicted	5.5	14.1	19.6
resampling_before_clustering	ratio_training	0.0	0.0	0.0
semi_resampling	ratio_test	11.3	19.6	30.9
semi_resampling	ratio_test_predicted	11.2	19.2	30.4
semi_resampling	ratio_training	0.0	0.0	0.0
resampling_after_clustering	ratio_test	0.0	0.0	0.0
resampling_after_clustering	ratio_test_predicted	1.6	0.5	2.1
resampling_after_clustering	ratio_training	0.0	0.0	0.0

3.2 Predicted ratios against training ratios

Figure 7 puts the predicted ratios in context of the training ratios, elucidating a variety of trends:

- `no_resampling`: **positive trend between the training and the predicted ratio**, but since the training and the test ratio also positively correlated (figure 2), the latter could be the one driving the predicted ratio of positives.
- `resampling_after_clustering` had a **constant training ratio**, meaning that the predicted ratio was not explainable by differences in training ratios.
- `resampling_before_clustering` showed instead a **negative relation between the training and the predicted ratio**. But since the former and the test ratio also anticorrelated (figure 2, the simplest explanation was that the test ratio drove the predicted test ratio.
- `semi_resampling` showed **no apparent correlation** between the predicted ratio and the training ratio.

The significance of the linear correlation backs up all the claims above (table 6).

Table 6: Correlations between train and predicted test active ratios. 95% confidence intervals and p-values are shown.

strategy	cor	ci_lower	ci_upper	alternative	pvalue
no_resampling	0.705	0.661	0.744	two.sided	5.22e-87
resampling_before_clustering	-0.136	-0.226	-0.045	two.sided	3.73e-03
semi_resampling	0.063	-0.032	0.158	two.sided	1.92e-01
resampling_after_clustering	NA	NA	NA	two.sided	NA

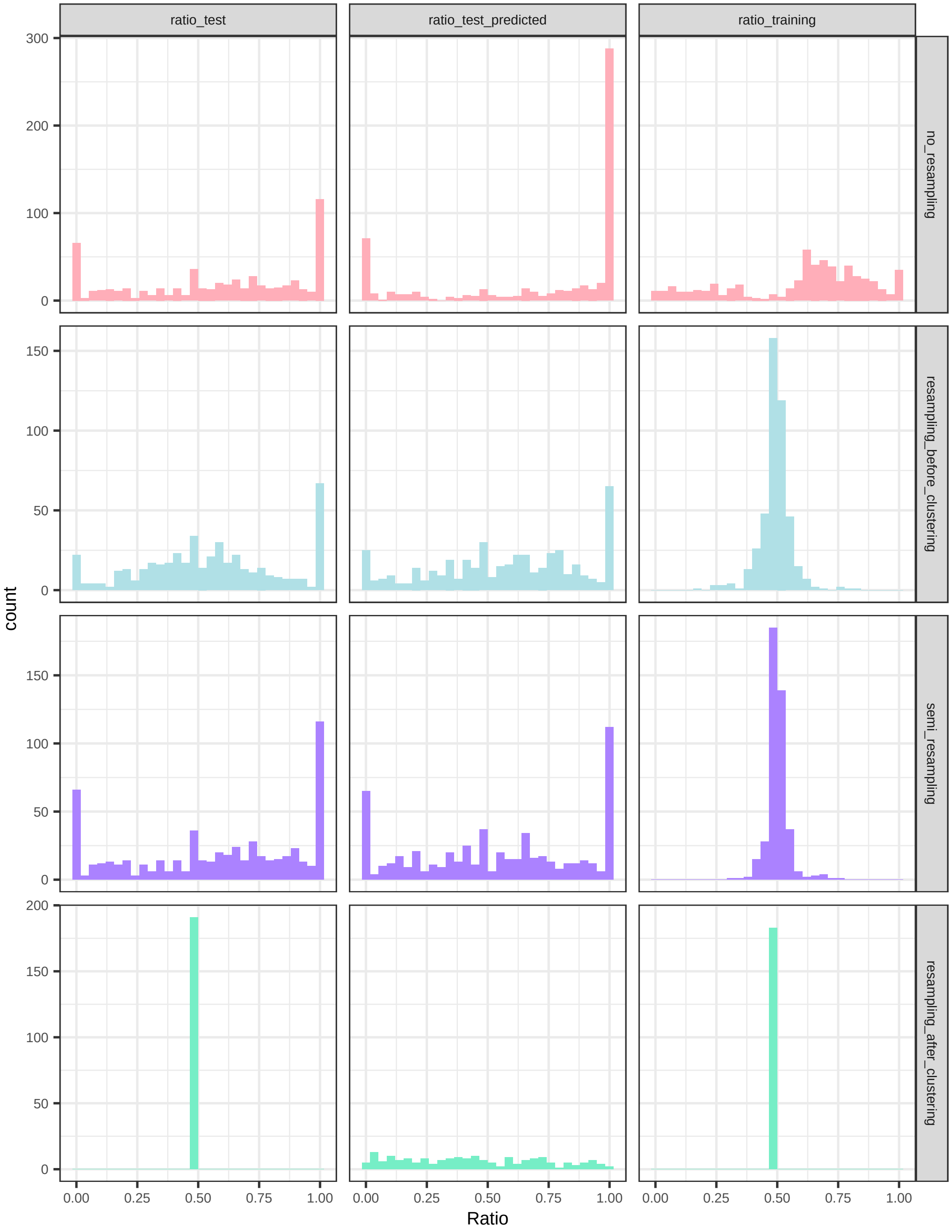

Figure 6: Distributios of the actives ratio in the training set and in the test set (both original and predicted by the deep learning model).

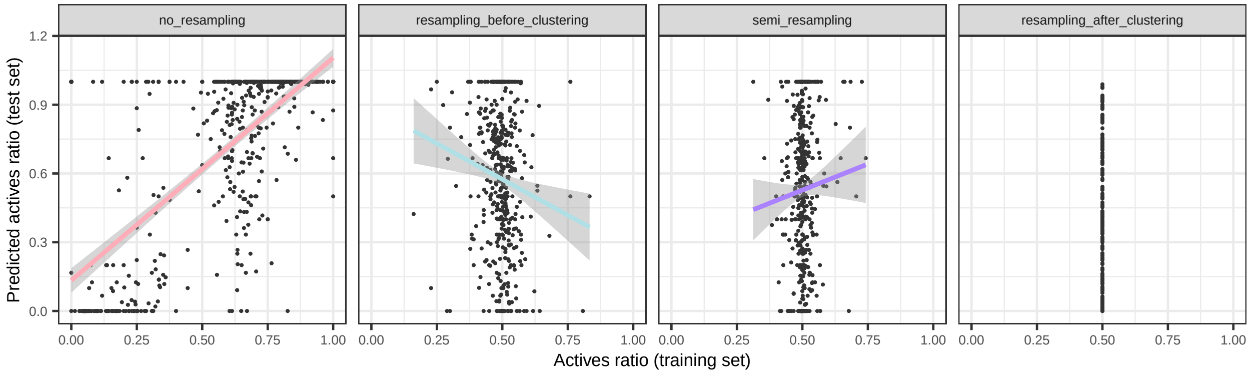

Figure 7: Predicted ratios, as a function of training ratios.

3.3 Linear models

The predicted ratio of actives r_{pred} was modelled through the following quasibinomial generalized linear models, stratified by strategy:

$$r_{pred} \sim r_{training} + r_{test} + \log10(n_{int}) + \log10(n_{seq}) + k_{fold}$$

The main variables of interest are the actual ratios in the training $r_{training}$ and in test r_{test}, both numeric between 0 and 1. As additional covariates, the number of interactions n_{int} and the sequence length n_{seq} (numerical) and the fold number k_{fold} (categorical) were also included.

3.3.1 In semi_resampling or resampling_before_clustering

Due to the lack of correlation between training and test ratios (figure 2), the `semi_resampling` strategy is the **ideal scenario to disentangle their effects** on the predicted ratio of actives (see model in table 7). This additive model suggests:

- The **test ratio is driving the predicted proportions, rather than the training ratio**.
- `n_interactions`: **the term is not significant in NRs**.

Table 7 also shows the additive model for `resampling_before_clustering`. This strategy showed negative correlation between training and test ratios, also providing a reasonably good scenario to distinguish their effects.

- **This model confirms both conclusions from the model** in the `semi_resampling` strategy, with **similar estimates.**

3.3.2 In no_resampling

The explanatory linear model under the `no_resampling` strategy (table 8) **suffers from the positive correlation between training and test ratios, which can be confounded**.

- Both `training_ratio` and `test_ratio` show a **positive effect** on the predicted fraction of actives.
- Although the **estimate is larger and more significant** for `training_ratio`, the **confounding effect and the very skewed distribution of the predicted ratios deems this model inconclusive**.

Table 7: Linear models to describe the predicted active ratio for the semi_resampling and the resampling_before_clustering strategies. Significance and 95% confidence intervals are included.

	semi_resampling	resampling_before_clustering
	(1)	(2)
ratio_training	2.479 (−0.348, 5.307)	−1.249 (−3.03, 0.533)
	p = 0.086	p = 0.17
ratio_test	1.183 (0.798, 1.567)***	1.148 (0.722, 1.575)***
	p = 3.689e-09	p = 2.076e-07
log10(n_interactions)	−0.035 (−0.251, 0.18)	−0.104 (−0.308, 0.101)
	p = 0.748	p = 0.322
log10(len_seq)	2.467 (1.431, 3.504)**	1.306 (0.36, 2.251)*
	p = 4.167e-06	p = 7.074e-03
fold1	0.818 (0.292, 1.344)*	1.257 (0.731, 1.783)**
	p = 2.461e-03	p = 3.795e-06
fold2	0.104 (−0.412, 0.621)	0.356 (−0.131, 0.843)
	p = 0.692	p = 0.153
fold3	−0.315 (−0.84, 0.21)	0.906 (0.4, 1.412)**
	p = 0.24	p = 4.989e-04
fold4	0.359 (−0.153, 0.871)	0.347 (−0.169, 0.864)
	p = 0.17	p = 0.188
fold5	0.468 (−0.057, 0.992)	0.623 (0.126, 1.119)*
	p = 0.081	p = 0.014
fold6	0.373 (−0.129, 0.875)	0.305 (−0.197, 0.808)
	p = 0.146	p = 0.235
fold7	−0.186 (−0.686, 0.314)	0.776 (0.272, 1.28)*
	p = 0.466	p = 2.717e-03
fold8	0.51 (1.837e-03, 1.018)*	0.675 (0.174, 1.176)*
	p = 0.05	p = 8.564e-03
fold9	0.812 (0.288, 1.337)*	−0.069 (−0.564, 0.427)
	p = 2.557e-03	p = 0.786
Constant	−8.753 (−11.857, −5.65)***	−3.532 (−6.296, −0.769)*
	p = 5.744e-08	p = 0.013
Observations	425	451
Note:		*p<0.05; **p<1.000e-03; ***p<1e-06

Table 8: Linear models to describe the predicted active ratio for the no_resampling strategy. Significance and 95% confidence intervals are included.

	no_resampling
ratio_training	5.556 (4.673, 6.44)***
	p = 4.823e-31
ratio_test	2.888 (2.242, 3.534)***
	p = 2.271e-17
log10(n_interactions)	−0.834 (−1.101, −0.568)***
	p = 1.616e-09
log10(len_seq)	1.202 (−0.471, 2.874)
	p = 0.16
fold1	0.24 (−0.591, 1.072)
	p = 0.571
fold2	−0.254 (−1.075, 0.568)
	p = 0.545
fold3	−1.286 (−2.06, −0.511)*
	p = 1.206e-03
fold4	0.322 (−0.603, 1.247)
	p = 0.495
fold5	0.077 (−0.791, 0.945)
	p = 0.862
fold6	−0.053 (−0.883, 0.778)
	p = 0.901
fold7	−0.632 (−1.465, 0.201)
	p = 0.138
fold8	−0.34 (−1.183, 0.503)
	p = 0.43
fold9	−0.384 (−1.186, 0.417)
	p = 0.348
Constant	−4.575 (−9.19, 0.039)
	p = 0.052
Observations	571
Note:	*p<0.05; **p<1.000e-03; ***p<1e-06

3.4 Conclusions

- Data imbalance exists in all strategies but in `resampling_after_clustering`, where balance is enforced.
- The correlation between a protein's ratio in train and test is positive in `no_resampling`, negative in `resampling_before_clustering` and null in `semi_resampling` and `resampling_after_clustering`.
- The main factor driving the ratio of actives in the model predictions, per protein, is the actual ratio of positives in the test set. Their distributions are resemblant, and linear models confirm the association.

All of them apply to NRs.

4 Description of baseline performance

Before evaluating the deep learning model, the performance metrics of the baselines were characterised, in order to pinpoint imbalance-sensitive and insensitive metrics. Metrics were called imbalance-sensitive if the imbalance-aware random baseline exhibited different performances between resampling strategies.

4.1 Descriptive plot

Figure 8 shows a fold-averaged picture of the metrics by protein. **Visual inspection suggested that accuracy, F1 and possibly balanced accuracy were affected by the data imbalance**. **F1 is the most apparent case**, see the quartiles in table 9.

Table 9: Quartiles for the baseline F1-scores.

strategy	Min.	1st Qu.	Median	Mean	3rd Qu.	Max.
no_resampling	0.000	0.333	0.601	0.540	0.792	1.000
resampling_before_clustering	0.125	0.403	0.465	0.443	0.493	0.597
semi_resampling	0.000	0.335	0.512	0.446	0.566	0.751
resampling_after_clustering	0.376	0.472	0.497	0.497	0.509	0.625

4.2 Linear models

Formally, each performance metric was described with the following linear model:

$$\text{metric} \sim \text{strategy} + \log10(n_{int}) + \log10(n_{seq}) + k_{fold}$$

The response was the quantitative metric of interest (one model per metric), while `strategy` was categorical with the following possibilities: `no_resampling`, `resampling_after_clustering`, `resampling_before_clustering`, `semi_resampling`. Additional covariates included the number of interactions n_{int} and the sequence length n_{seq} (numerical) and the fold number k_{fold} (categorical). The `strategy` variable was tested with a type 3 ANOVA, being **significant** with $p < 0.05$ for `acc`, `f1` and `balanced_acc` (table 10).

Based on this, metrics were divided in two types:

- Those where the baseline was different between strategies, i.e. imbalance-sensitive: `acc`, `f1` and `balanced_acc`. Therefore, before comparing strategies, the baseline performance needed to be accounted for.
- Those where the baseline was constant, i.e. imbalance-insensitive: `auroc`, `mcc`. Here we could compare strategies directly.

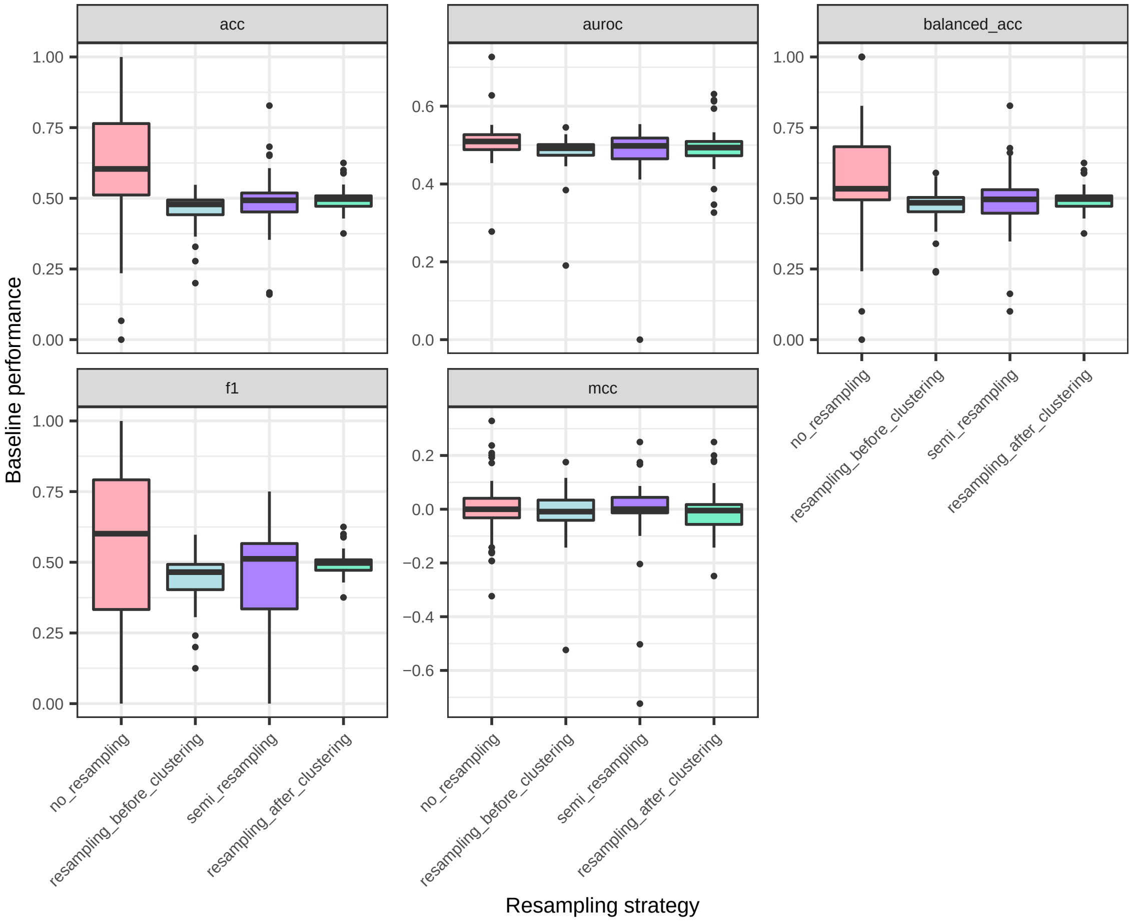

Figure 8: Performance metrics for imbalance-aware random baselines. Data points correspond to proteins, averaged over folds.

Table 10: ANOVA p-values for including the resampling strategy as a regressor. Significant p-values imply that differences exist between resampling strategies.

strategy	variable	Sum Sq	Df	F value	Pr(>F)
acc	strategy	6.9011780	3	5.57e+01	2.74e-34
auroc	strategy	0.0137418	3	1.38e-01	9.38e-01
balanced_acc	strategy	2.1662131	3	1.59e+01	3.33e-10
f1	strategy	3.3010337	3	1.53e+01	7.73e-10
mcc	strategy	0.0466933	3	2.74e-01	8.44e-01

All applies to NRs as well.

5 Description of deep learning model performance

An overview of fold-averaged performances is displayed in figure 9, where strategies are paired with their baselines. This illustrates the **issue of direct strategy comparison** with imbalance-sensitive metrics, which was especially visible for the F1-score. Some metrics are undefined in edge cases (e.g. AUROC when only actives or only inactives are available); table 11 summarizes the number of proteins, added over folds, whose metrics were computable.

Table 11: Number of computable performance measures. AUROC was undefined for proteins with all actives or unactives in the test set, hence its lower counts.

strategy	acc	auroc	f1	balanced_acc	mcc
no_resampling	582	402	582	582	582
resampling_before_clustering	453	364	453	453	453
semi_resampling	582	402	582	582	582
resampling_after_clustering	191	191	191	191	191

5.1 Absolute, baseline-naive performance

Anologous to the baseline performance models, absolute metric models (not accounting for baselines) were fitted:

$$\text{metric} \sim \text{strategy} + \log10(n_{int}) + \log10(n_{seq}) + k_{fold}$$

The `strategy` covariate was **always significant** in a type 3 ANOVA (table 12). The models, summarized in 13, showed **different behaviour in imbalance-sensitive and insensitive metrics**. Pairwise comparisons of the strategy coefficients using Tukey's method would point to **two different pictures** (figure 10), further confirmed when prioritizing the strategies according to their expected performance through the linear models (table 14 and figure 11):

- **Accuracy, balanced accuracy and F1-score** suggested that `no_resampling` was the **best strategy**, but this was confounded by the fact that it also held the **highest baselines** .
- **AUROC and MCC** and showed instead that `resampling_before_clustering` held the **highest performance estimates**.

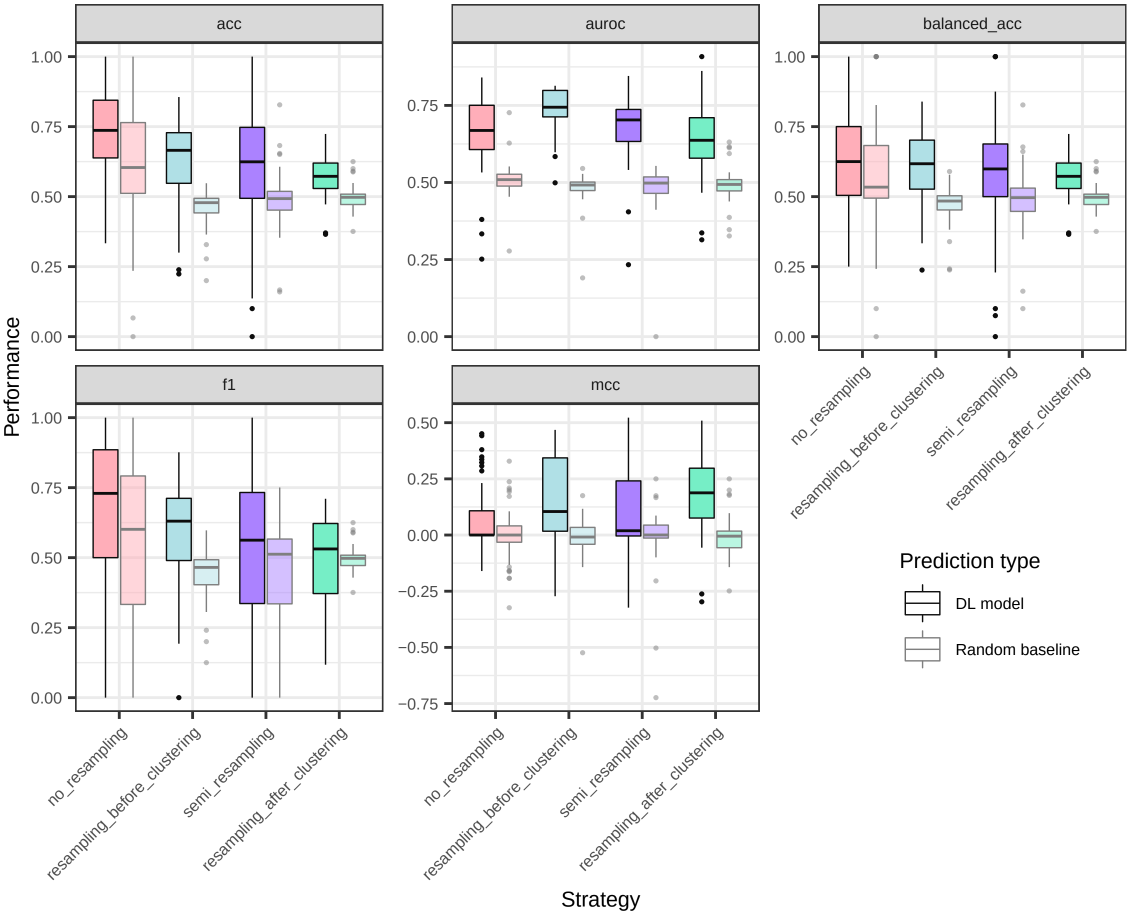

Figure 9: Performance metrics for balancing strategies and their corresponding imbalance-aware random baselines. Data points correspond to proteins, averaged over folds.

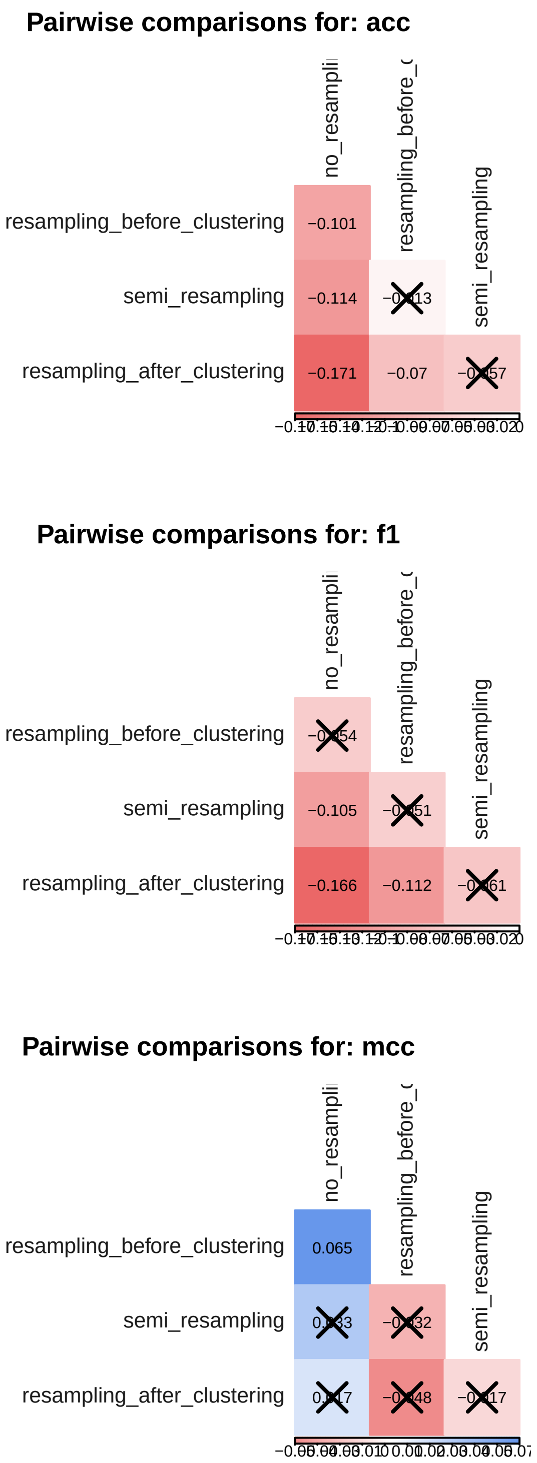

Figure 10: Pairwise comparison of strategy performance using Tukey method.

Table 12: ANOVA p-values for including the resampling strategy as a regressor in the performance models.

strategy	variable	Sum Sq	Df	F value	Pr(>F)
acc	strategy	5.8906886	3	3.01e+01	5.94e-19
auroc	strategy	0.6372396	3	3.79e+00	1.01e-02
f1	strategy	5.2009857	3	1.54e+01	6.59e-10
balanced_acc	strategy	0.8804841	3	4.74e+00	2.70e-03
mcc	strategy	1.1138090	3	5.16e+00	1.50e-03

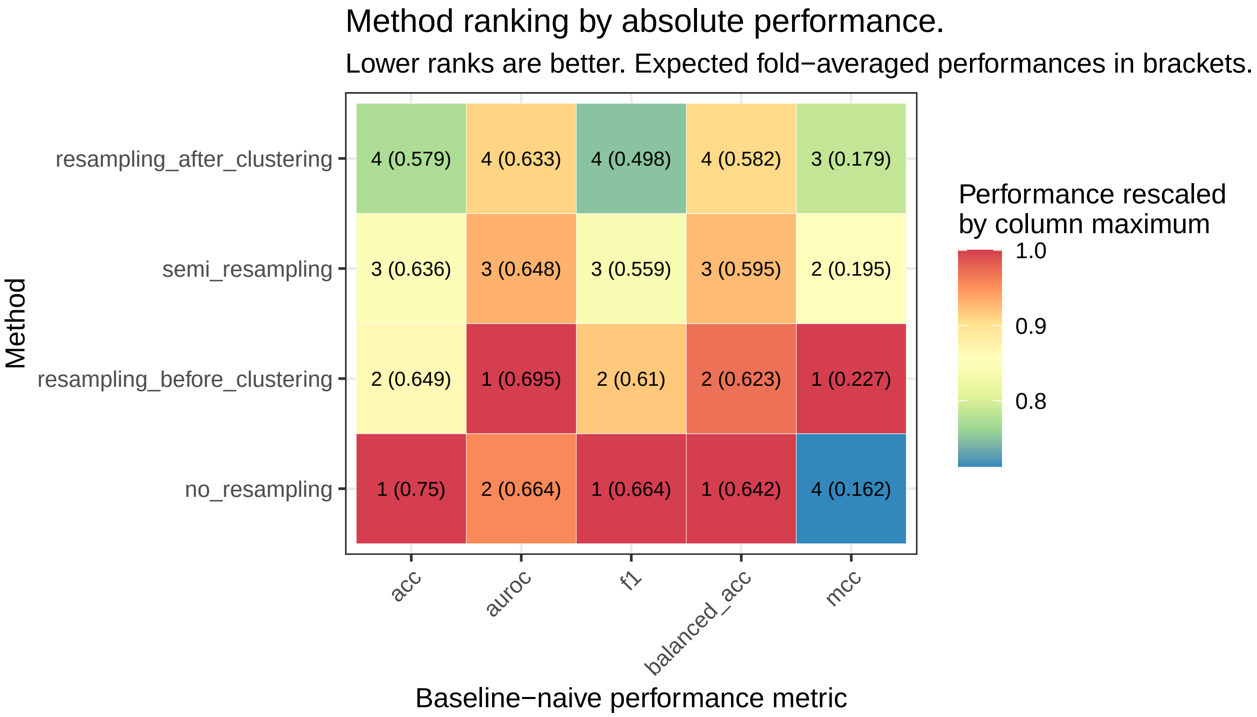

Figure 11: Method ranking according to the linear model predicted performances for each metric. Baseline metrics were ignored.

Table 13: Linear models to describe each performance metric. Standard deviations in parentheses.

	acc	auroc	f1	balanced_acc	mcc
	(1)	(2)	(3)	(4)	(5)
strategyresampling_before_clustering	-0.101^{***}	0.032	-0.054^{*}	-0.019	0.065^{**}
	(0.016)	(0.017)	(0.021)	(0.016)	(0.017)
	p = 6.966e-10	p = 0.067	p = 0.011	p = 0.23	p = 1.307e-04
strategysemi_resampling	-0.114^{***}	-0.016	-0.105^{***}	-0.047^{*}	0.033^{*}
	(0.015)	(0.017)	(0.02)	(0.015)	(0.016)
	p = 4.598e-14	p = 0.335	p = 1.075e-07	p = 1.207e-03	p = 0.034
strategyresampling_after_clustering	-0.171^{***}	-0.03	-0.166^{***}	-0.06^{*}	0.017
	(0.022)	(0.021)	(0.029)	(0.022)	(0.023)
	p = 3.743e-14	p = 0.156	p = 1.699e-08	p = 6.135e-03	p = 0.475
log10(n_interactions)	0.035^{**}	0.114^{***}	0.057^{**}	0.015	0.131^{***}
	(9.064e-03)	(0.011)	(0.012)	(8.828e-03)	(9.513e-03)
	p = 1.451e-04	p = 1.867e-25	p = 1.952e-06	p = 0.088	p = 5.040e-41
log10(len_seq)	-0.047	0.178^{**}	0.258^{**}	-0.077	0.074
	(0.051)	(0.053)	(0.066)	(0.049)	(0.053)
	p = 0.348	p = 7.888e-04	p = 1.033e-04	p = 0.118	p = 0.163
fold1	0.091^{**}	7.686e-03	0.174^{***}	0.048	0.025
	(0.026)	(0.028)	(0.035)	(0.026)	(0.028)
	p = 5.510e-04	p = 0.782	p = 5.102e-07	p = 0.063	p = 0.361
fold2	-4.926e-03	0.028	0.022	-0.019	0.03
	(0.028)	(0.029)	(0.036)	(0.027)	(0.029)
	p = 0.858	p = 0.326	p = 0.546	p = 0.478	p = 0.295
fold3	0.021	0.041	-0.013	0.016	0.036
	(0.026)	(0.028)	(0.035)	(0.026)	(0.028)
	p = 0.43	p = 0.148	p = 0.704	p = 0.535	p = 0.2
fold4	0.068^{*}	-1.440e-03	0.143^{**}	0.055^{*}	-0.02
	(0.028)	(0.031)	(0.036)	(0.027)	(0.029)
	p = 0.014	p = 0.962	p = 8.640e-05	p = 0.042	p = 0.496
fold5	0.017	-0.026	-0.013	0.013	-0.03
	(0.028)	(0.03)	(0.037)	(0.028)	(0.03)
	p = 0.542	p = 0.39	p = 0.731	p = 0.641	p = 0.308
fold6	0.061^{*}	0.019	0.137^{**}	0.033	0.024
	(0.026)	(0.028)	(0.035)	(0.026)	(0.028)
	p = 0.02	p = 0.49	p = 8.201e-05	p = 0.195	p = 0.379
fold7	0.045	0.032	0.066	0.033	0.026
	(0.027)	(0.03)	(0.036)	(0.027)	(0.029)
	p = 0.1	p = 0.285	p = 0.067	p = 0.223	p = 0.364
fold8	0.09^{*}	0.036	0.1^{*}	0.073^{*}	0.059^{*}
	(0.028)	(0.029)	(0.036)	(0.027)	(0.029)
	p = 1.066e-03	p = 0.224	p = 5.932e-03	p = 6.518e-03	p = 0.043
fold9	0.097^{**}	0.047	0.143^{**}	0.074^{*}	0.075^{*}
	(0.027)	(0.028)	(0.035)	(0.026)	(0.028)
	p = 2.477e-04	p = 0.091	p = 3.981e-05	p = 4.380e-03	p = 6.852e-03
Constant	0.738^{***}	-0.155	-0.28	0.781^{***}	-0.423^{*}
	(0.139)	(0.145)	(0.183)	(0.136)	(0.146)
	p = 1.291e-07	p = 0.286	p = 0.125	p = 9.610e-09	p = 3.832e-03
Observations	1808	1359	1808	1808	1808
R^2	0.068	0.107	0.078	0.023	0.129
Adjusted R^2	0.061	0.098	0.071	0.015	0.122

Note: *p<0.05; **p<1.000e-03; ***p<1e-06

Table 14: Expected absolute performances, by metric and strategy, with 95% confidence intervals.

metric	strategy	emmean	SE	lower.CL	upper.CL
acc	no_resampling	0.750	1.189e-02	0.727	0.773
acc	resampling_before_clustering	0.649	1.238e-02	0.625	0.674
acc	semi_resampling	0.636	1.189e-02	0.613	0.659
acc	resampling_after_clustering	0.579	1.857e-02	0.543	0.616
auroc	no_resampling	0.664	1.237e-02	0.640	0.688
auroc	resampling_before_clustering	0.695	1.263e-02	0.671	0.720
auroc	semi_resampling	0.648	1.237e-02	0.623	0.672
auroc	resampling_after_clustering	0.633	1.723e-02	0.600	0.667
f1	no_resampling	0.664	1.560e-02	0.633	0.695
f1	resampling_before_clustering	0.610	1.624e-02	0.578	0.642
f1	semi_resampling	0.559	1.560e-02	0.528	0.590
f1	resampling_after_clustering	0.498	2.435e-02	0.450	0.546
balanced_acc	no_resampling	0.642	1.158e-02	0.619	0.665
balanced_acc	resampling_before_clustering	0.623	1.206e-02	0.600	0.647
balanced_acc	semi_resampling	0.595	1.158e-02	0.572	0.618
balanced_acc	resampling_after_clustering	0.582	1.808e-02	0.547	0.618
mcc	no_resampling	0.162	1.248e-02	0.138	0.187
mcc	resampling_before_clustering	0.227	1.300e-02	0.202	0.253
mcc	semi_resampling	0.195	1.248e-02	0.171	0.220
mcc	resampling_after_clustering	0.179	1.949e-02	0.141	0.217

5.2 Baseline-adjusted performance

To address the pitfalls of the direct comparison of metrics whose baselines may differ, baseline-adjusted performance metrics were defined and modelled analogously. Specifically:

$$\text{adj\_metric} = \text{metric} - \text{baseline}$$

A descriptive plot of the the adjusted metrics (figure 12) pointed to a **scenario diffent than that of unadjusted ones** (figure 9).

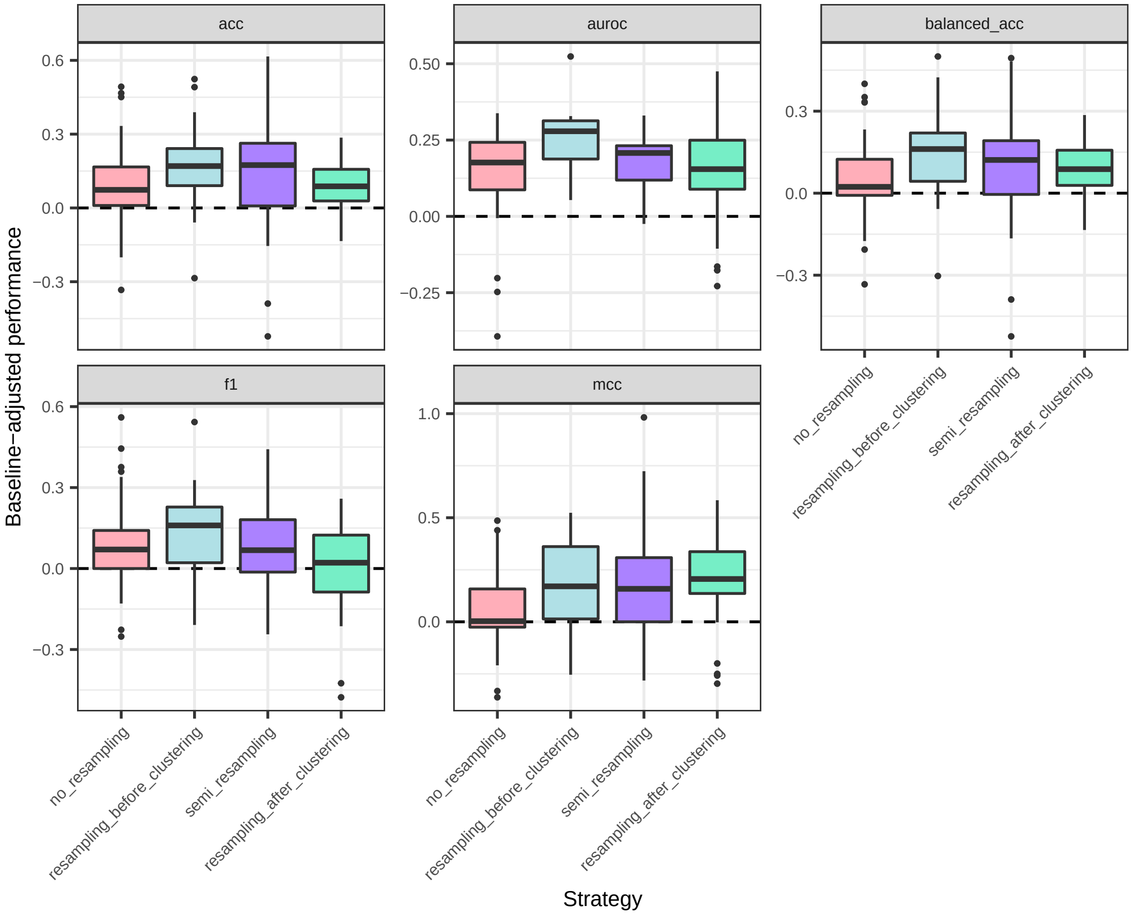

Figure 12: Baseline-adjusted performance metrics for balancing strategies. Data points correspond to proteins, averaged over folds.

Adjusted performance metrics were described with the following linear model:

$$\text{adj\_metric} \sim \text{strategy} + \log10(n_{int}) + \log10(n_{seq}) + k_{fold}$$

Note that while all metrics but `mcc` were non-negative, the adjusted metrics could show negative values when the performance of the DL model was lower than that of the baseline.

Again, `strategy` covariate was **always significant except for AUROC in NRs** in a type 3 ANOVA (table 15). **Baseline adjustment brought a uniform behaviour across the models** (table 16), further confirmed in pairwise coefficient comparison (Tukey's method, figure 13) and in their expected performance (table 17 and figure 14):

- **In NRs**, `resampling_before_clustering` **was the best performer, followed by a technical tie between** `no_resampling` and `semi_resampling`, and `resampling_after_clustering`.
- **In NRs the statistical significance is lower, but the general picture in the imbalance-insensitve non-adjusted metrics remains in the adjusted metrics: resampling before clustering being the best performer and no resampling being sensibly penalised** .

Table 15: ANOVA p-values for including the resampling strategy as a regressor in the adjusted performance models.

strategy	variable	Sum Sq	Df	F value	Pr(>F)
acc	strategy	1.4366294	3	6.41e+00	2.57e-04
auroc	strategy	0.2707975	3	1.14e+00	3.31e-01
f1	strategy	2.2855264	3	1.03e+01	1.05e-06
balanced_acc	strategy	1.4034456	3	6.11e+00	3.96e-04
mcc	strategy	1.4303470	3	3.57e+00	1.36e-02

Conclusions drawn from the baseline-adjusted performance analysis:

- **In NRs, resampling after clustering and semi resampling are technically tied, so data augmentation in the test set is not the largest performance drive**.
- **In NRs, the almost six-fold reduction in number of samples compared to kinases probably limited the statistical power when comparing semi resampling and no resampling**. Tukey's method gave no significant differences at $p < 0.05$. However, the model coefficient of semi resampling was significantly greater than the reference level (no resampling) in MCC ($p = 0.033$), but not in balanced accuracy ($p = 0.067$) or accuracy ($p = 0.111$), despite the positive estimates}, see table 16. This was **consistent with the observation that the predicted proportion of positives of the PCM model was mainly driven by the actual data balance in the test set, rather than that of the training set. Combined with the healthier distributions of predicted active ratios** of `semi_resampling` against `no_resampling`, this made a **case in favour of the former**.
- In **five** out of five metrics, **proteins with more interactions were better predicted** (table 16).

Table 16: Linear models to describe each adjusted performance metric. Standard deviations in parentheses.

	acc	auroc	f1	balanced_acc	mcc
	(1)	(2)	(3)	(4)	(5)
strategyresampling_before_clustering	0.056*	0.027	0.05*	0.072**	0.074*
	(0.017)	(0.021)	(0.017)	(0.018)	(0.023)
	p = 1.474e-03	p = 0.182	p = 4.337e-03	p = 4.886e-05	p = 1.637e-03
strategysemi_resampling	0.028	6.049e-03	−3.269e-03	0.033	0.051*
	(0.018)	(0.021)	(0.018)	(0.018)	(0.024)
	p = 0.111	p = 0.774	p = 0.854	p = 0.067	p = 0.033
strategyresampling_after_clustering	−0.038	−0.017	−0.084**	6.519e-03	0.033
	(0.025)	(0.026)	(0.025)	(0.025)	(0.033)
	p = 0.121	p = 0.516	p = 6.993e-04	p = 0.795	p = 0.323
log10(n_interactions)	0.037*	0.093***	0.025*	0.035*	0.137***
	(0.011)	(0.014)	(0.011)	(0.011)	(0.015)
	p = 1.227e-03	p = 1.128e-10	p = 0.026	p = 2.006e-03	p = 4.000e-19
log10(len_seq)	−4.762e-03	0.133*	0.098	0.023	0.059
	(0.056)	(0.065)	(0.056)	(0.057)	(0.076)
	p = 0.933	p = 0.039	p = 0.082	p = 0.691	p = 0.434
fold1	0.099**	0.024	0.139**	0.068*	0.059
	(0.03)	(0.034)	(0.03)	(0.03)	(0.04)
	p = 8.824e-04	p = 0.484	p = 2.766e-06	p = 0.024	p = 0.138
fold2	−1.428e-04	8.102e-03	0.028	−0.015	0.037
	(0.03)	(0.035)	(0.03)	(0.031)	(0.041)
	p = 0.996	p = 0.817	p = 0.349	p = 0.633	p = 0.369
fold3	0.046	0.028	0.03	0.046	0.054
	(0.03)	(0.035)	(0.03)	(0.03)	(0.04)
	p = 0.125	p = 0.425	p = 0.307	p = 0.128	p = 0.178
fold4	0.054	−0.021	0.071*	0.038	−0.012
	(0.031)	(0.037)	(0.031)	(0.031)	(0.041)
	p = 0.078	p = 0.574	p = 0.021	p = 0.222	p = 0.767
fold5	0.035	−0.06	0.048	0.031	−0.032
	(0.031)	(0.036)	(0.031)	(0.032)	(0.042)
	p = 0.259	p = 0.099	p = 0.12	p = 0.329	p = 0.438
fold6	0.05	0.019	0.081*	0.036	0.044
	(0.03)	(0.035)	(0.03)	(0.03)	(0.04)
	p = 0.092	p = 0.591	p = 6.661e-03	p = 0.235	p = 0.275
fold7	0.037	4.122e-03	0.05	0.026	0.045
	(0.03)	(0.036)	(0.03)	(0.031)	(0.041)
	p = 0.219	p = 0.909	p = 0.1	p = 0.398	p = 0.263
fold8	0.092*	0.036	0.109**	0.091*	0.095*
	(0.03)	(0.035)	(0.03)	(0.031)	(0.041)
	p = 2.382e-03	p = 0.303	p = 3.287e-04	p = 3.253e-03	p = 0.02
fold9	0.083*	0.049	0.093*	0.07*	0.1*
	(0.03)	(0.034)	(0.03)	(0.03)	(0.04)
	p = 5.452e-03	p = 0.151	p = 1.774e-03	p = 0.022	p = 0.012
Constant	−0.014	−0.471*	−0.31*	−0.121	−0.422*
	(0.155)	(0.177)	(0.155)	(0.157)	(0.207)
	p = 0.928	p = 7.935e-03	p = 0.045	p = 0.441	p = 0.042
Observations	1630	1277	1630	1630	1630
R^2	0.031	0.049	0.042	0.03	0.077
Adjusted R^2	0.022	0.039	0.033	0.022	0.069

Note: *p<0.05; **p<1.000e-03; ***p<1e-06

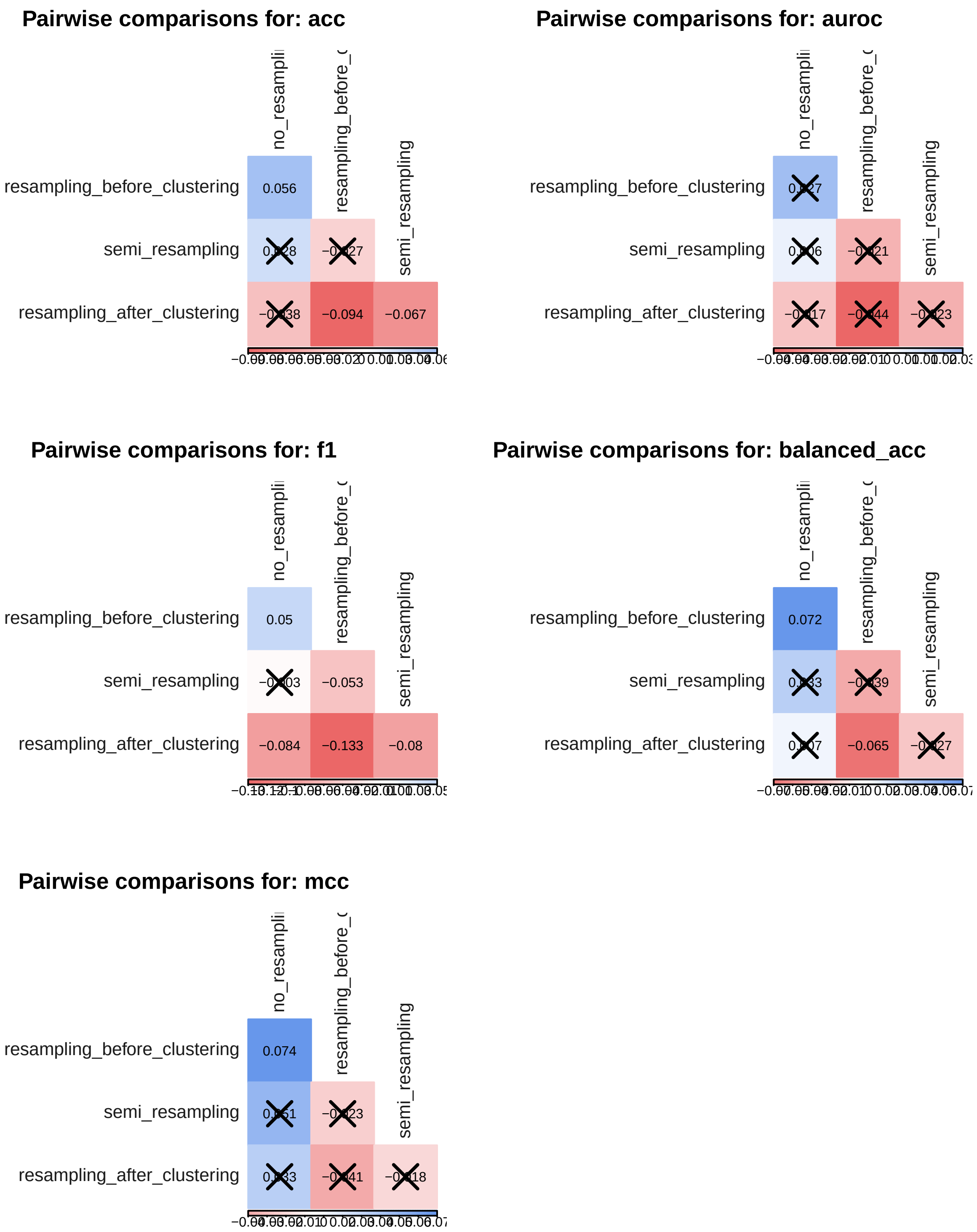

Figure 13: Pairwise comparison of strategy adjusted performance using Tukey method.

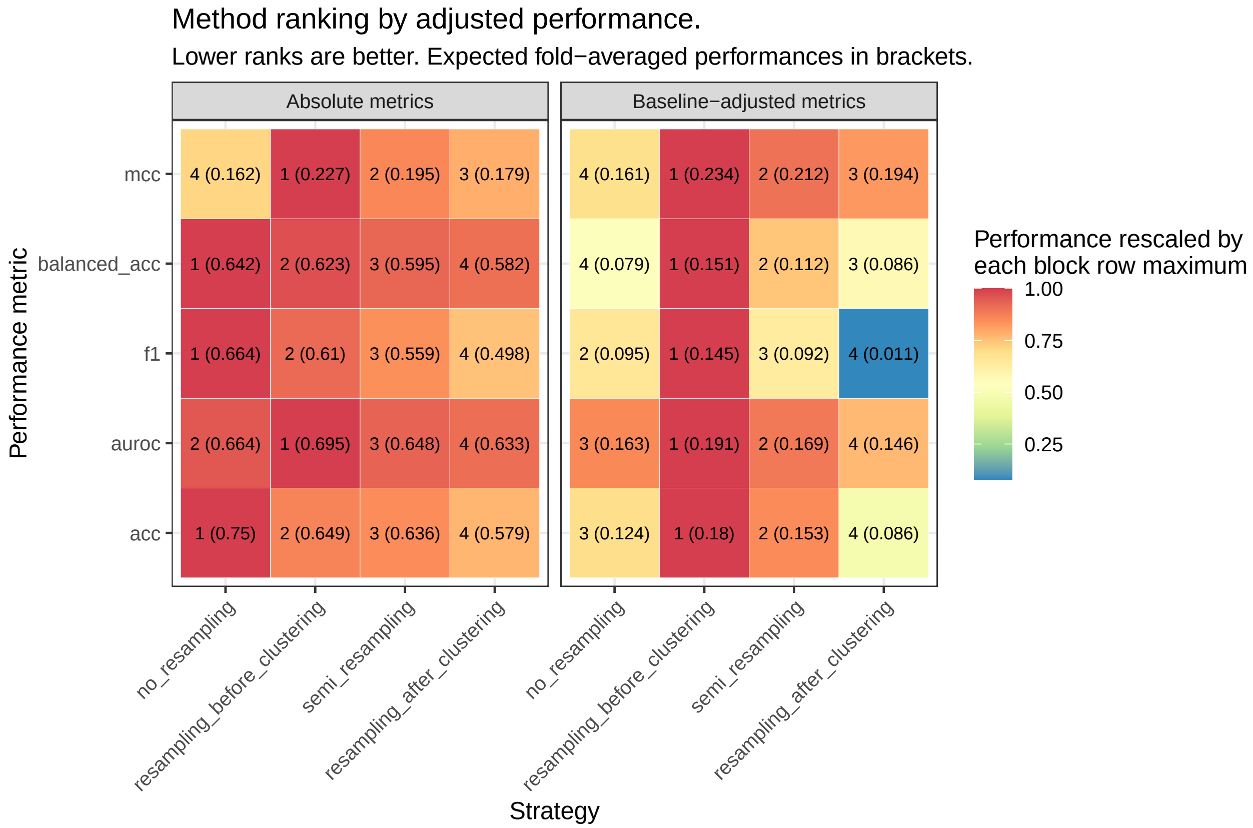

Figure 14: Method ranking according to the linear model predicted adjusted performances for each metric. Baseline metrics were taken into account in the adjustment. For a direct comparison, the same ranking using the absolute metrics was kept side by side.

Table 17: Expected adjusted performances, by metric and strategy, with 95% confidence intervals.

metric	strategy	emmean	SE	lower.CL	upper.CL
acc	no_resampling	0.124	1.338e-02	0.098	0.151
acc	resampling_before_clustering	0.180	1.353e-02	0.153	0.207
acc	semi_resampling	0.153	1.376e-02	0.126	0.180
acc	resampling_after_clustering	0.086	2.029e-02	0.046	0.126
auroc	no_resampling	0.163	1.507e-02	0.134	0.193
auroc	resampling_before_clustering	0.191	1.520e-02	0.161	0.221
auroc	semi_resampling	0.169	1.564e-02	0.139	0.200
auroc	resampling_after_clustering	0.146	2.090e-02	0.105	0.187
f1	no_resampling	0.095	1.333e-02	0.069	0.121
f1	resampling_before_clustering	0.145	1.348e-02	0.118	0.171
f1	semi_resampling	0.092	1.370e-02	0.065	0.119
f1	resampling_after_clustering	0.011	2.021e-02	-0.028	0.051
balanced_acc	no_resampling	0.079	1.356e-02	0.053	0.106
balanced_acc	resampling_before_clustering	0.151	1.371e-02	0.124	0.178
balanced_acc	semi_resampling	0.112	1.393e-02	0.085	0.140
balanced_acc	resampling_after_clustering	0.086	2.055e-02	0.045	0.126
mcc	no_resampling	0.161	1.790e-02	0.126	0.196
mcc	resampling_before_clustering	0.234	1.810e-02	0.199	0.270
mcc	semi_resampling	0.212	1.840e-02	0.176	0.248
mcc	resampling_after_clustering	0.194	2.713e-02	0.140	0.247

6 Reproducibility

- R version 3.6.3 (2020-02-29), `x86_64-pc-linux-gnu`
- Locale: `LC_CTYPE=en_US.UTF-8`, `LC_NUMERIC=C`, `LC_TIME=es_ES.UTF-8`, `LC_COLLATE=en_US.UTF-8`, `LC_MONETARY=es_ES.UTF-8`, `LC_MESSAGES=en_US.UTF-8`, `LC_PAPER=es_ES.UTF-8`, `LC_NAME=C`, `LC_ADDRESS=C`, `LC_TELEPHONE=C`, `LC_MEASUREMENT=es_ES.UTF-8`, `LC_IDENTIFICATION=C`
- Running under: `Ubuntu 16.04.7 LTS`
- Matrix products: default
- BLAS: `/usr/lib/atlas-base/atlas/libblas.so.3.0`
- LAPACK: `/usr/lib/atlas-base/atlas/liblapack.so.3.0`
- Base packages: base, datasets, graphics, grDevices, methods, stats, utils
- Other packages: corrplot 0.84, dplyr 1.0.5, forcats 0.5.1, ggplot2 3.3.3, gsubfn 0.7, kableExtra 1.3.4, magrittr 2.0.1, proto 1.0.0, purrr 0.3.4, readr 1.4.0, stargazer 5.2.2, stringr 1.4.0, tibble 3.1.0, tidyr 1.1.3, tidyverse 1.3.0
- Loaded via a namespace (and not attached): abind 1.4-5, assertthat 0.2.1, backports 1.2.1, bookdown 0.21, broom 0.7.5, car 3.0-10, carData 3.0-4, cellranger 1.1.0, cli 2.3.1, codetools 0.2-16, colorspace 2.0-0, compiler 3.6.3, crayon 1.4.1, curl 4.3, data.table 1.14.0, DBI 1.1.1, dbplyr 2.1.0, digest 0.6.27, ellipsis 0.3.1, emmeans 1.5.4, estimability 1.3, evaluate 0.14, fansi 0.4.2, farver 2.1.0, foreign 0.8-76, fs 1.5.0, generics 0.1.0, glue 1.4.2, grid 3.6.3, gtable 0.3.0, haven 2.3.1, highr 0.8, hms 1.0.0, htmltools 0.5.1.1, httr 1.4.2, jsonlite 1.7.2, knitr 1.31, labeling 0.4.2, lattice 0.20-41, lifecycle 1.0.0, lubridate 1.7.10, MASS 7.3-53, Matrix 1.2-18, mgcv 1.8-33, modelr 0.1.8, multcomp 1.4-16, munsell 0.5.0, mvtnorm 1.1-1, nlme 3.1-149, openxlsx 4.2.3, pillar 1.5.1,

pkgconfig 2.0.3, plyr 1.8.6, R6 2.5.0, RColorBrewer 1.1-2, Rcpp 1.0.6, readxl 1.3.1, reprex 1.0.0, reshape2 1.4.4, rio 0.5.26, rlang 0.4.10, rmarkdown 2.7, rstudioapi 0.13, rvest 0.3.6, sandwich 3.0-0, scales 1.1.1, splines 3.6.3, stringi 1.5.3, survival 3.2-7, svglite 2.0.0, systemfonts 1.0.1, tcltk 3.6.3, TH.data 1.0-10, tidyselect 1.1.0, tools 3.6.3, utf8 1.1.4, vctrs 0.3.6, viridisLite 0.3.0, webshot 0.5.2, withr 2.4.1, xfun 0.21, xml2 1.3.2, xtable 1.8-4, yaml 2.2.1, zip 2.1.1, zoo 1.8-8

Appendix 5: model predictions and performance (PRs)

Angela Lopez-del Rio   Sergio Picart-Armada   Alexandre Perera-Lluna

01/03/2021

Contents

1 Overview **1**

2 Description of data balance **2**
2.1 Distributions of the actives ratio . . . 2
2.2 Comparing training and test imbalance . . . 2
2.3 Other covariates . . . 2

3 Linear models on predicted proportions **5**
3.1 Distributions of the predicted ratios . . . 5
3.2 Predicted ratios against training ratios . . . 6
3.3 Linear models . . . 6
3.4 Conclusions . . . 8

4 Description of baseline performance **11**
4.1 Descriptive plot . . . 11
4.2 Linear models . . . 12

5 Description of deep learning model performance **12**
5.1 Absolute, baseline-naive performance . . . 14
5.2 Baseline-adjusted performance . . . 18

6 Reproducibility **23**

1 Overview

This supplement describes the behaviour of the proteochemometrics (PCM) deep learning model to predict protein-compound bioactivity for **PRs**. Specifically, this includes the descriptive statistics of data imbalance: the proportion of actives per protein in the training and test sets during the model fitting and the predicted proportion of actives. The model performance per protein was also described, pinpointing the most influential factors and characterising the proteins with the most extreme performances.

Four strategies (no_resampling, resampling_before_clustering, semi_resampling, resampling_after_clustering) were considered. For each of those, 10 folds of repeated holdout were run, and 5 performance metrics were computed: acc, auroc, f1, balanced_acc, mcc. This led to a total of 5904 values of performance. Since some strategies involved the upsampling method SMOTE, proteins whose sample sizes did not allow upsampling were excluded (table 1).

Table 1: Number of proteins for which performance metrics were computed. The resampling after clustering was the most stringent strategy regarding eligible proteins, since the resampling was carried out after the clustering, which introduced more imbalance.

Strategy	Fold 0	Fold 1	Fold 2	Fold 3	Fold 4	Fold 5	Fold 6	Fold 7	Fold 8	Fold 9
no_resampling	187	178	210	198	206	171	184	192	205	165
resampling_before_clustering	144	144	141	149	154	152	149	140	150	150
semi_resampling	187	178	210	198	206	171	184	192	205	165
resampling_after_clustering	63	53	74	62	75	57	59	68	71	57

2 Description of data balance

The data balancing strategy had an impact on the actual data balance, defined as the proportion of active molecules for a protein. Furthermore, specific trends were observed in the original data in the training and test sets, as well as in the values predicted by the deep learning model.

2.1 Distributions of the actives ratio

The histograms in figure 1 revealed trends:

- `no_resampling` **keeps similar data imbalance in training and test.**
- `resampling_before_clustering` and `semi_resampling` **lead to a more balanced training set, but not so much for the test set.**
- `resampling_after_clustering` **kept balanced proteins** in both training and test sets.

In addition, **test sets with imbalance tended to magnify** it and create extreme cases (all actives or all inactives), probably due to the combination of the clustering and the lower sample sizes in the test sets compared to training.

2.2 Comparing training and test imbalance

Figure 2 revealed both positive, negative and null trends between the training and test set protein balances.

- `no_resampling` showed a **positive relation** between both, i.e. proteins were prone to keep their (im)balance in train and test.
- `resampling_before_clustering` showed an **inverse relationship** instead. This was expected since this strategy started from globally balanced proteins, and after the clustering, an imbalance in one direction in the training set entailed an inverse imbalance in the test set.
- `semi_resampling` led to **independent train and test balances, expected since the train set was resampled, breaking any correlation with the test set balance.**
- `resampling_after_clustering` always **kept balanced proteins**, by design.

Table 2 displays the Pearson correlation estimate, 95% confidence interval and p-value for each strategy (except `resampling_after_clustering`, where ratios are constant), further confirming the claims above.

2.3 Other covariates

The effect of the number of interactions of each protein in its corresponding set and fold (figure 3) and the protein length in amino acids (figure 4) on the test set imbalance was investigated:

- **Proteins with greatest imbalance** (i.e. where `(0.5 - ratio_test)^2` was greatest) **tended to be among those with the least interactions. Linear correlations were significant** (table 3).

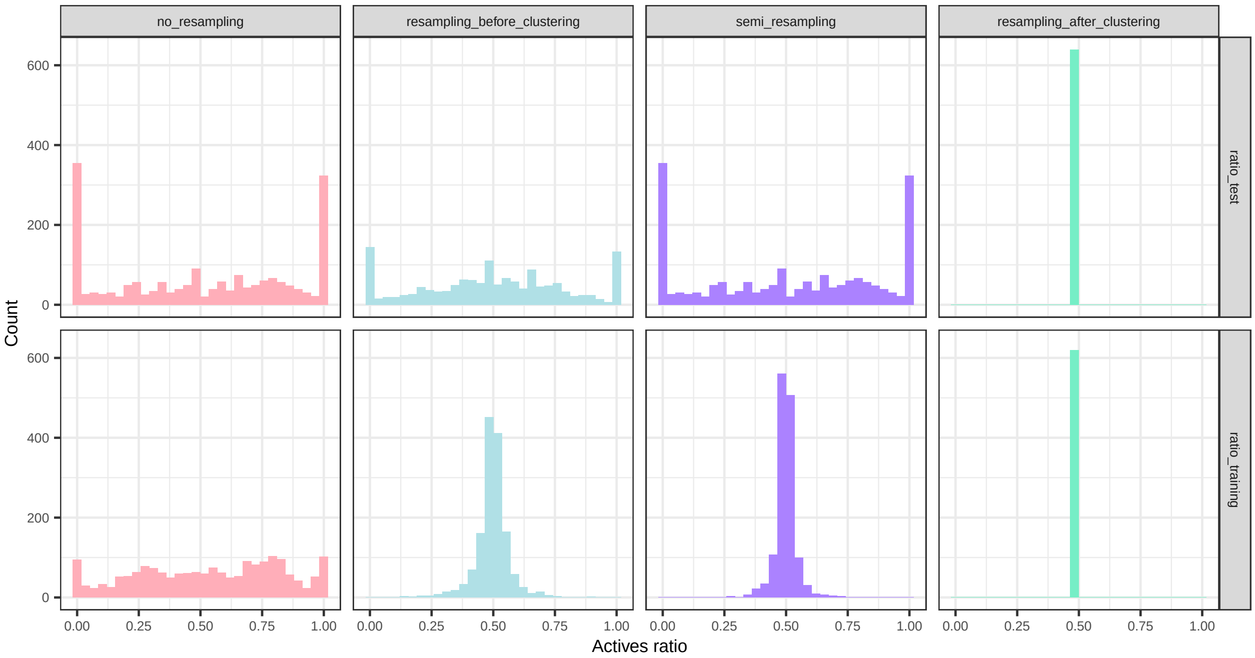

Figure 1: Distributios of the actives ratio in the training set and in the test set (both original and predicted by the deep learning model).

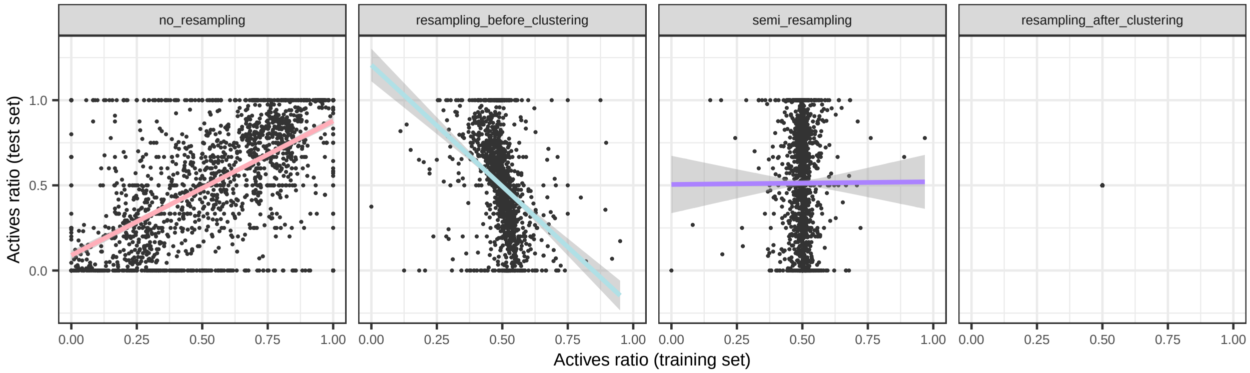

Figure 2: Comparison of the training and test ratios, by resampling strategy. A linear fit line was added per strategy.

Table 2: Correlations between train and test active ratios. 95% confidence intervals and p-values are shown.

strategy	cor	ci_lower	ci_upper	alternative	pvalue
no_resampling	0.630	0.602	0.657	two.sided	5.40e-206
resampling_before_clustering	-0.359	-0.403	-0.314	two.sided	1.58e-45
semi_resampling	0.002	-0.050	0.055	two.sided	9.26e-01
resampling_after_clustering	NA	NA	NA	two.sided	NA

- **The sequence length had no obvious effect on the protein imbalance. Linear correlations were not significant** (`resampling_before_clustering`) or significant but low (`no_resampling`, `semi_resampling`).

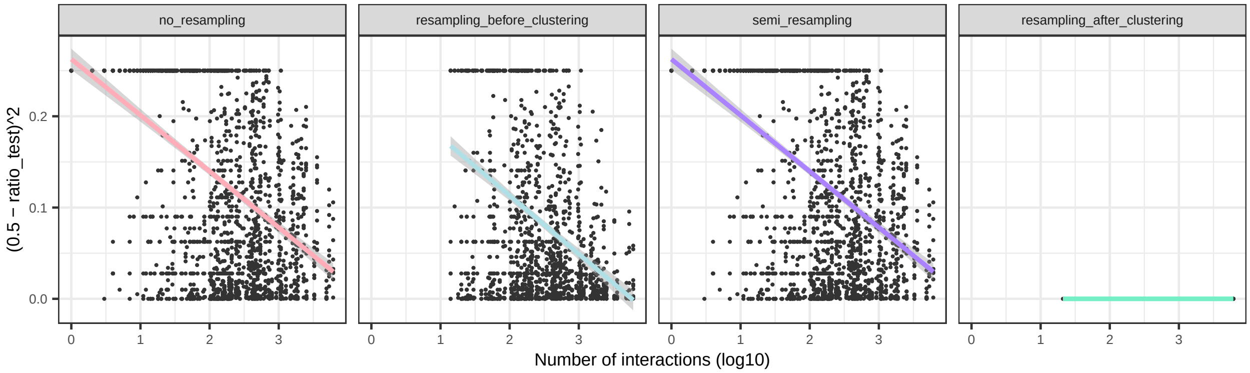

Figure 3: Data imbalance in the test set as a function of the number of available interactions for each protein.

Table 3: Correlations between imbalance (as defined above) and number of interactions. 95% confidence intervals and p-values are shown.

strategy	cor	ci_lower	ci_upper	alternative	pvalue
no_resampling	-0.289	-0.330	-0.247	two.sided	7.19e-38
resampling_before_clustering	-0.294	-0.340	-0.246	two.sided	2.81e-30
semi_resampling	-0.289	-0.330	-0.247	two.sided	7.19e-38
resampling_after_clustering	NA	NA	NA	two.sided	NA

Table 4: Correlations between imbalance (as defined above) and sequence length. 95% confidence intervals and p-values are shown.

strategy	cor	ci_lower	ci_upper	alternative	pvalue
no_resampling	0.049	0.004	0.094	two.sided	3.15e-02
resampling_before_clustering	-0.035	-0.086	0.016	two.sided	1.81e-01
semi_resampling	0.049	0.004	0.094	two.sided	3.15e-02
resampling_after_clustering	NA	NA	NA	two.sided	NA

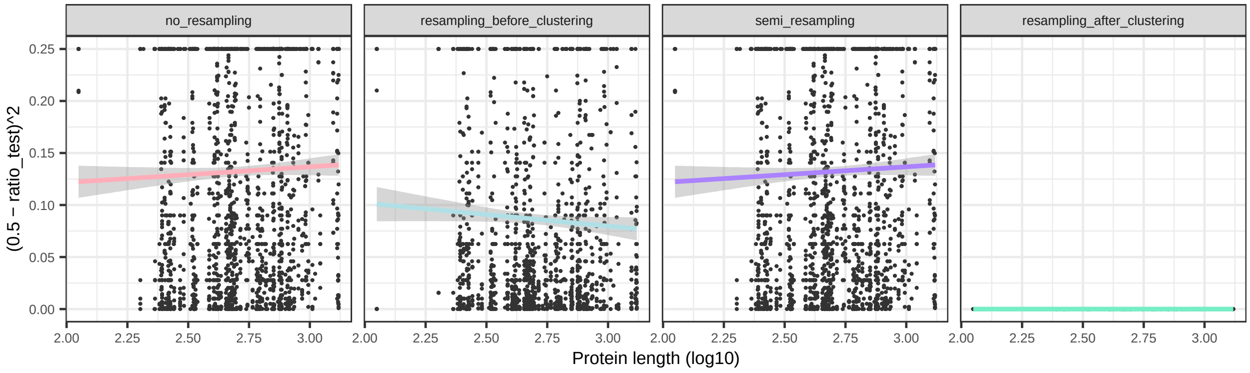

Figure 4: Data imbalance in the test set as a function of the protein length, in amino acids.

3 Linear models on predicted proportions

The next key question was to narrow down the factor driving the predicted proportion of actives. The main options under consideration were:

1. A constant, global imbalance that the model would learn from the whole dataset.
2. The protein-wise imbalance that the model would learn in the training set.
3. A test set-driven imbalance, based on its actual imbalance.

3.1 Distributions of the predicted ratios

After the model predictions in the test set were binarized (actives were those whose probabilities exceeded 0.5), the ratio of predicted actives was computed by protein. This ratio, shown in figure 5, suggested that:

- `no_resampling` was noticeably **inclined to predict more positives than negatives, and in general more extreme ratios than the real ones**.
- `resampling_before_clustering` and `semi_resampling` **alleviated the imbalance in the predictions, being was quite close to the actual distribution**.
- `resampling_after_clustering` **kept a wide and symmetric distribution of predicted actives**.

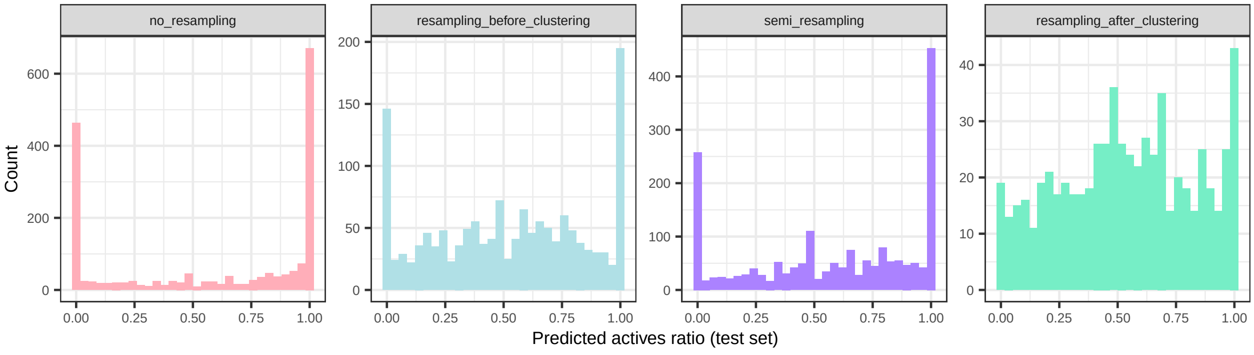

Figure 5: Ratios of the prediction values, after binarization.

Now, representing together (1) the original training and test ratios, and (2) the predicted ratios in test (figure 6) eased a general qualitative assessment: **the distribution was most resemblant to that of the test proportions to that of the training ones** (except `resampling_after_clustering`, since

those proportions are constant). Table 5 displays how `no_resampling` was **inclined to predict more extreme cases (all positives or all negatives)**, `resampling_before_clustering` and `semi_resampling` **alleviated this phenomenon**, and `resampling_after_clustering` was **essentially balanced**.

Table 5: Percentage of extreme cases, i.e. proteins with all actives or inactives.

strategy	RatioSet	all_inactives	all_actives	all_extremes
no_resampling	ratio_test	18.4	16.9	35.3
no_resampling	ratio_test_predicted	23.8	33.6	57.5
no_resampling	ratio_training	4.8	5.3	10.1
resampling_before_clustering	ratio_test	10.0	9.2	19.2
resampling_before_clustering	ratio_test_predicted	9.6	12.8	22.4
resampling_before_clustering	ratio_training	0.1	0.0	0.1
semi_resampling	ratio_test	18.4	16.9	35.3
semi_resampling	ratio_test_predicted	13.5	23.4	36.9
semi_resampling	ratio_training	0.1	0.0	0.1
resampling_after_clustering	ratio_test	0.0	0.0	0.0
resampling_after_clustering	ratio_test_predicted	2.7	4.5	7.2
resampling_after_clustering	ratio_training	0.0	0.0	0.0

3.2 Predicted ratios against training ratios

Figure 7 puts the predicted ratios in context of the training ratios, elucidating a variety of trends:

- `no_resampling`: **positive trend between the training and the predicted ratio**, but since the training and the test ratio also positively correlated (figure 2), the latter could be the one driving the predicted ratio of positives.
- `resampling_after_clustering` had a **constant training ratio**, meaning that the predicted ratio was not explainable by differences in training ratios.
- `resampling_before_clustering` showed a **negative but not significant coefficient in PRs (p=0.073)**.
- `semi_resampling` showed **no apparent correlation** between the predicted ratio and the training ratio.

The significance of the linear correlation backs up all the claims above (table 6).

Table 6: Correlations between train and predicted test active ratios. 95% confidence intervals and p-values are shown.

strategy	cor	ci_lower	ci_upper	alternative	pvalue
no_resampling	0.732	0.711	0.753	two.sided	3.35e-312
resampling_before_clustering	-0.047	-0.098	0.004	two.sided	7.29e-02
semi_resampling	-0.017	-0.069	0.035	two.sided	5.20e-01
resampling_after_clustering	NA	NA	NA	two.sided	NA

3.3 Linear models

The predicted ratio of actives r_{pred} was modelled through the following quasibinomial generalized linear models, stratified by strategy:

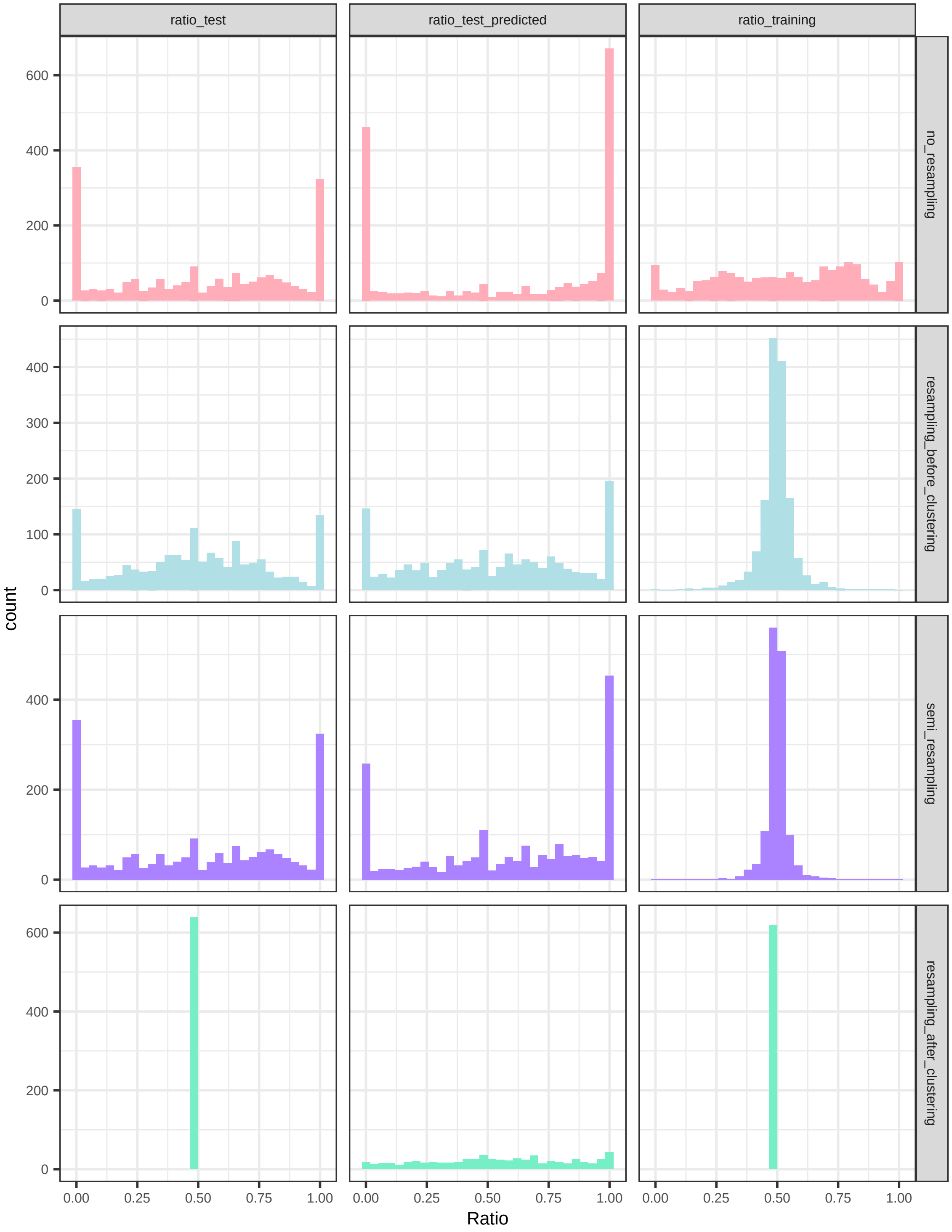

Figure 6: Distributios of the actives ratio in the training set and in the test set (both original and predicted by the deep learning model).

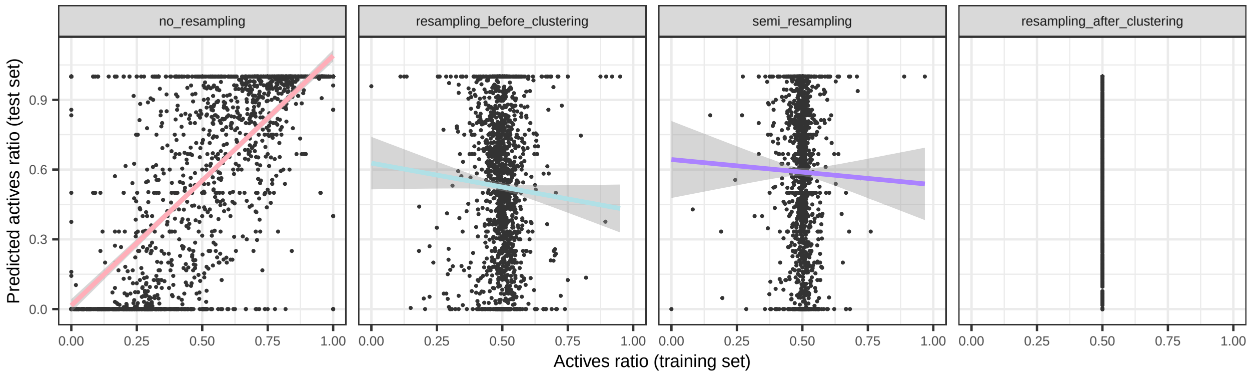

Figure 7: Predicted ratios, as a function of training ratios.

$$r_{pred} \sim r_{training} + r_{test} + \log10(n_{int}) + \log10(n_{seq}) + k_{fold}$$

The main variables of interest are the actual ratios in the training $r_{training}$ and in test r_{test}, both numeric between 0 and 1. As additional covariates, the number of interactions n_{int} and the sequence length n_{seq} (numerical) and the fold number k_{fold} (categorical) were also included.

3.3.1 In semi_resampling or resampling_before_clustering

Due to the lack of correlation between training and test ratios (figure 2), the `semi_resampling` strategy is the **ideal scenario to disentangle their effects** on the predicted ratio of actives (see model in table 7). This additive model suggests:

- The **test ratio is driving the predicted proportions, rather than the training ratio**.
- `n_interactions`: **the term is not significant in PRs**.

Table 7 also shows the additive model for `resampling_before_clustering`. This strategy showed negative correlation between training and test ratios, also providing a reasonably good scenario to distinguish their effects.

- **This model confirms both conclusions from the model** in the `semi_resampling` strategy. **The coefficient for ratio test is similar and significant in both models; the coefficient of ratio training is non-significant in both models.**

3.3.2 In no_resampling

The explanatory linear model under the `no_resampling` strategy (table 8) **suffers from the positive correlation between training and test ratios, which can be confounded**.

- Both `training_ratio` and `test_ratio` show a **positive effect** on the predicted fraction of actives.
- Although the **estimate is larger and more significant** for `training_ratio`, the **confounding effect of the predicted ratios deems this model inconclusive**. **For PRs the distribution of predicted ratios is not very skewed**.

3.4 Conclusions

- Data imbalance exists in all strategies but in `resampling_after_clustering`, where balance is enforced.

Table 7: Linear models to describe the predicted active ratio for the semi_resampling and the resampling_before_clustering strategies. Significance and 95% confidence intervals are included.

	semi_resampling	resampling_before_clustering
	(1)	(2)
ratio_training	−0.193 (−1.563, 1.177)	0.872 (−0.109, 1.853)
	p = 0.783	p = 0.082
ratio_test	1.149 (0.931, 1.368)***	1.178 (0.928, 1.428)***
	p = 5.340e-24	p = 9.248e-20
log10(n_interactions)	−0.044 (−0.174, 0.086)	−7.026e-03 (−0.123, 0.109)
	p = 0.51	p = 0.905
log10(len_seq)	−0.451 (−0.804, −0.097)*	−0.163 (−0.5, 0.173)
	p = 0.013	p = 0.342
fold1	0.324 (0.014, 0.635)*	0.491 (0.19, 0.792)*
	p = 0.041	p = 1.418e-03
fold2	0.421 (0.115, 0.726)*	0.297 (−2.457e-03, 0.597)
	p = 6.999e-03	p = 0.052
fold3	0.857 (0.537, 1.177)***	0.914 (0.606, 1.221)***
	p = 1.839e-07	p = 6.974e-09
fold4	0.011 (−0.291, 0.312)	0.213 (−0.08, 0.505)
	p = 0.945	p = 0.155
fold5	−0.182 (−0.486, 0.123)	0.015 (−0.278, 0.309)
	p = 0.243	p = 0.92
fold6	−0.516 (−0.815, −0.216)**	−0.343 (−0.642, −0.045)*
	p = 7.708e-04	p = 0.024
fold7	−0.509 (−0.81, −0.208)**	0.164 (−0.136, 0.463)
	p = 9.439e-04	p = 0.285
fold8	0.2 (−0.102, 0.501)	0.086 (−0.208, 0.381)
	p = 0.195	p = 0.567
fold9	0.413 (0.092, 0.733)*	0.07 (−0.236, 0.376)
	p = 0.012	p = 0.655
Constant	1.12 (−0.08, 2.32)	−0.641 (−1.757, 0.475)
	p = 0.068	p = 0.26
Observations	1405	1452
Note:		*p<0.05; **p<1.000e-03; ***p<1e-06

Table 8: Linear models to describe the predicted active ratio for the no_resampling strategy. Significance and 95% confidence intervals are included.

	no_resampling
ratio_training	6.124 (5.564, 6.684)***
	p = 3.380e-91
ratio_test	1.491 (1.131, 1.851)***
	p = 8.721e-16
log10(n_interactions)	−0.547 (−0.706, −0.388)***
	p = 2.010e-11
log10(len_seq)	−0.298 (−0.831, 0.236)
	p = 0.275
fold1	0.585 (0.111, 1.06)*
	p = 0.016
fold2	0.494 (0.039, 0.948)*
	p = 0.033
fold3	1.851 (1.363, 2.34)***
	p = 1.682e-13
fold4	0.522 (0.07, 0.974)*
	p = 0.024
fold5	−0.317 (−0.795, 0.161)
	p = 0.193
fold6	−0.367 (−0.831, 0.097)
	p = 0.121
fold7	0.486 (0.026, 0.946)*
	p = 0.038
fold8	0.383 (−0.071, 0.838)
	p = 0.099
fold9	0.363 (−0.113, 0.84)
	p = 0.135
Constant	−1.794 (−3.288, −0.301)*
	p = 0.019
Observations	1858
Note:	*p<0.05; **p<1.000e-03; ***p<1e-06

- The correlation between a protein's ratio in train and test is positive in `no_resampling`, negative in `resampling_before_clustering` and null in `semi_resampling` and `resampling_after_clustering`.
- The main factor driving the ratio of actives in the model predictions, per protein, is the actual ratio of positives in the test set. Their distributions are resemblant, and linear models confirm the association.

All of them apply to PRs as well, except for the negative correlation in resampling before clustering, which is non-significant in PRs.

4 Description of baseline performance

Before evaluating the deep learning model, the performance metrics of the baselines were characterised, in order to pinpoint imbalance-sensitive and insensitive metrics. Metrics were called imbalance-sensitive if the imbalance-aware random baseline exhibited different performances between resampling strategies.

4.1 Descriptive plot

Figure 8 shows a fold-averaged picture of the metrics by protein. **Visual inspection suggested that accuracy, F1 and possibly balanced accuracy were affected by the data imbalance**. **accuracy is the most apparent case in PRs**, see the quartiles in table 9.

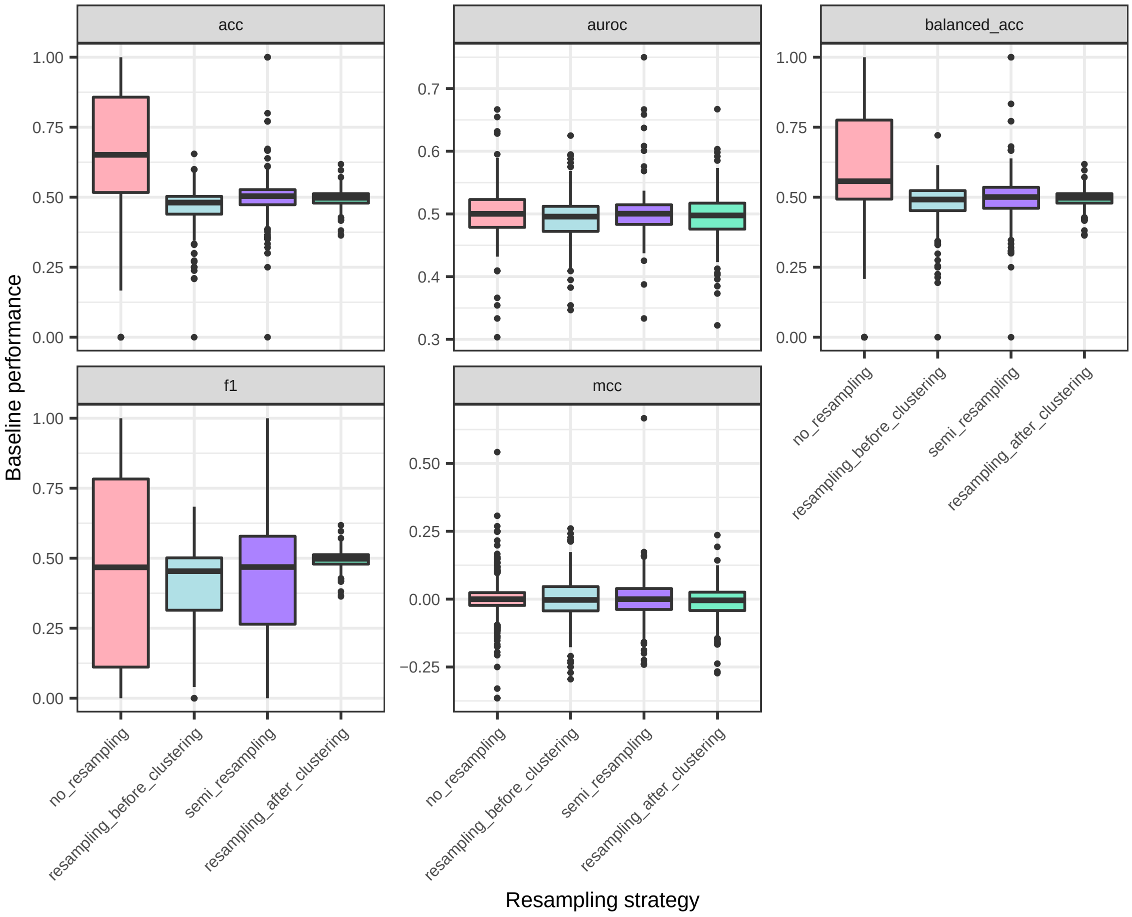

Figure 8: Performance metrics for imbalance-aware random baselines. Data points correspond to proteins, averaged over folds.

Table 9: Quartiles for the baseline F1-scores.

strategy	Min.	1st Qu.	Median	Mean	3rd Qu.	Max.
no_resampling	0.000	0.111	0.468	0.455	0.783	1.000
resampling_before_clustering	0.000	0.314	0.454	0.406	0.501	0.684
semi_resampling	0.000	0.264	0.469	0.424	0.579	1.000
resampling_after_clustering	0.364	0.479	0.498	0.493	0.513	0.618

4.2 Linear models

Formally, each performance metric was described with the following linear model:

$$\text{metric} \sim \text{strategy} + \log10(n_{int}) + \log10(n_{seq}) + k_{fold}$$

The response was the quantitative metric of interest (one model per metric), while `strategy` was categorical with the following possibilities: `no_resampling`, `resampling_after_clustering`, `resampling_before_clustering`, `semi_resampling`. Additional covariates included the number of interactions n_{int} and the sequence length n_{seq} (numerical) and the fold number k_{fold} (categorical). The `strategy` variable was tested with a type 3 ANOVA, being **significant** with $p < 0.05$ for `acc`, `f1` and `balanced_acc` (table 10).

Table 10: ANOVA p-values for including the resampling strategy as a regressor. Significant p-values imply that differences exist between resampling strategies.

strategy	variable	Sum Sq	Df	F value	Pr(>F)
acc	strategy	26.3568423	3	2.18e+02	3.44e-133
auroc	strategy	0.0655018	3	6.69e-01	5.71e-01
balanced_acc	strategy	7.6709749	3	5.67e+01	4.59e-36
f1	strategy	5.5240885	3	2.42e+01	1.56e-15
mcc	strategy	0.0668456	3	4.14e-01	7.43e-01

Based on this, metrics were divided in two types:

- Those where the baseline was different between strategies, i.e. imbalance-sensitive: `acc`, `f1` and `balanced_acc`. Therefore, before comparing strategies, the baseline performance needed to be accounted for.
- Those where the baseline was constant, i.e. imbalance-insensitive: `auroc`, `mcc`. Here we could compare strategies directly.

All applies to PRs as well.

5 Description of deep learning model performance

An overview of fold-averaged performances is displayed in figure 9, where strategies are paired with their baselines. This illustrates the **issue of direct strategy comparison** with imbalance-sensitive metrics, which was especially visible for the F1-score. Some metrics are undefined in edge cases (e.g. AUROC when only actives or only inactives are available); table 11 summarizes the number of proteins, added over folds, whose metrics were computable.

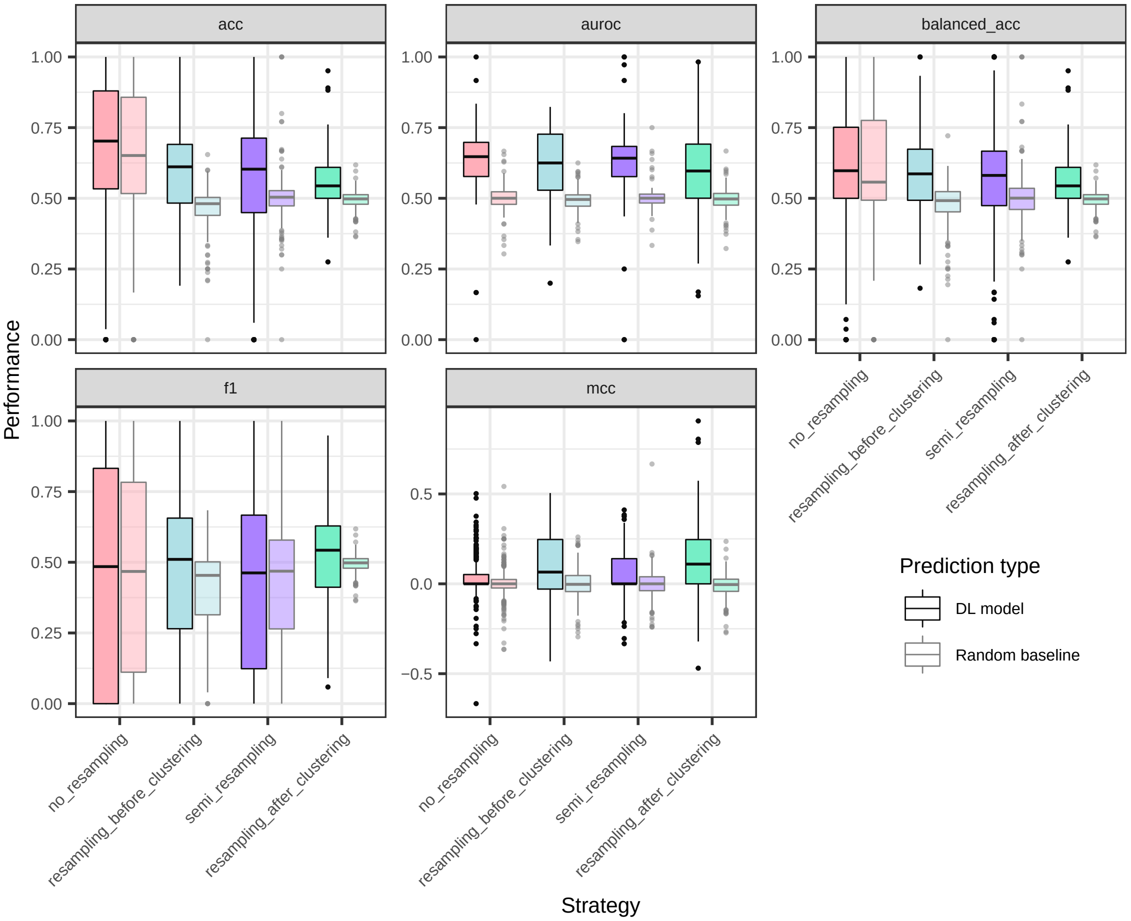

Figure 9: Performance metrics for balancing strategies and their corresponding imbalance-aware random baselines. Data points correspond to proteins, averaged over folds.

Table 11: Number of computable performance measures. AUROC was undefined for proteins with all actives or unactives in the test set, hence its lower counts.

strategy	acc	auroc	f1	balanced_acc	mcc
no_resampling	1896	1227	1896	1896	1896
resampling_before_clustering	1473	1199	1473	1473	1473
semi_resampling	1896	1227	1896	1896	1896
resampling_after_clustering	639	639	639	639	639

5.1 Absolute, baseline-naive performance

Anologous to the baseline performance models, absolute metric models (not accounting for baselines) were fitted:

$$\text{metric} \sim \text{strategy} + \log10(n_{int}) + \log10(n_{seq}) + k_{fold}$$

The `strategy` covariate was **always significant** in a type 3 ANOVA (table 12). The models, summarized in 13, showed **different behaviour in imbalance-sensitive and insensitive metrics**. Pairwise comparisons of the strategy coefficients using Tukey's method would point to **two different pictures** (figure 10), further confirmed when prioritizing the strategies according to their expected performance through the linear models (table 14 and figure 11):

- **Accuracy and balanced accuracy** suggested that `no_resampling` was the **best strategy**, but this was confounded by the fact that it also held the **highest baselines**.
- **In PRs, MCC suggested resampling before clustering and semi resampling as the best strategies**.
- **In PRs, F1 and AUROC also suggested the no resampling strategy as the best one, but its baselines were not higher**.

Table 12: ANOVA p-values for including the resampling strategy as a regressor in the performance models.

strategy	variable	Sum Sq	Df	F value	Pr(>F)
acc	strategy	17.362539	3	7.55e+01	6.14e-48
auroc	strategy	1.090834	3	6.37e+00	2.65e-04
f1	strategy	2.170764	3	5.85e+00	5.54e-04
balanced_acc	strategy	1.524648	3	7.34e+00	6.61e-05
mcc	strategy	1.027279	3	5.16e+00	1.46e-03

Table 13: Linear models to describe each performance metric. Standard deviations in parentheses.

	acc	auroc	f1	balanced_acc	mcc
	(1)	(2)	(3)	(4)	(5)
strategyresampling_before_clustering	-0.109^{***}	-0.011	-0.045^{**}	-0.023^{*}	0.028^{*}
	(9.727e-03)	(9.718e-03)	(0.012)	(9.248e-03)	(9.053e-03)
	p = 9.079e-29	p = 0.262	p = 2.456e-04	p = 0.013	p = 2.249e-03
strategysemi_resampling	-0.095^{***}	-5.248e-03	-0.012	-0.026^{*}	0.028^{**}
	(8.990e-03)	(9.647e-03)	(0.011)	(8.548e-03)	(8.367e-03)
	p = 5.466e-26	p = 0.586	p = 0.311	p = 1.999e-03	p = 7.070e-04
strategyresampling_after_clustering	-0.164^{***}	-0.05^{**}	-0.048^{*}	-0.054^{**}	6.680e-03
	(0.013)	(0.012)	(0.017)	(0.012)	(0.012)
	p = 1.868e-35	p = 2.966e-05	p = 4.330e-03	p = 1.362e-05	p = 0.585
log10(n_interactions)	0.04^{***}	0.068^{***}	0.121^{***}	6.244e-03	0.082^{***}
	(5.085e-03)	(6.200e-03)	(6.463e-03)	(4.834e-03)	(4.732e-03)
	p = 9.245e-15	p = 1.322e-27	p = 1.553e-75	p = 0.197	p = 2.538e-66
log10(len_seq)	0.059^{*}	0.011	-0.044	0.031	-0.017
	(0.018)	(0.019)	(0.023)	(0.017)	(0.017)
	p = 1.028e-03	p = 0.55	p = 0.055	p = 0.07	p = 0.31
fold1	0.044^{*}	-4.954e-03	0.035	0.031	-0.019
	(0.016)	(0.017)	(0.021)	(0.016)	(0.015)
	p = 7.992e-03	p = 0.769	p = 0.09	p = 0.05	p = 0.206
fold2	-0.035^{*}	-0.012	0.013	-0.035^{*}	-7.381e-03
	(0.016)	(0.016)	(0.02)	(0.015)	(0.015)
	p = 0.029	p = 0.463	p = 0.513	p = 0.022	p = 0.618
fold3	-0.036^{*}	7.250e-03	0.057^{*}	-0.036^{*}	2.073e-03
	(0.016)	(0.016)	(0.02)	(0.015)	(0.015)
	p = 0.024	p = 0.654	p = 5.524e-03	p = 0.02	p = 0.89
fold4	1.550e-03	8.425e-03	0.016	-0.015	3.890e-03
	(0.016)	(0.016)	(0.02)	(0.015)	(0.015)
	p = 0.922	p = 0.593	p = 0.417	p = 0.314	p = 0.792
fold5	0.024	0.037^{*}	9.275e-03	0.013	0.037^{*}
	(0.016)	(0.017)	(0.021)	(0.016)	(0.015)
	p = 0.148	p = 0.024	p = 0.658	p = 0.408	p = 0.016
fold6	0.039^{*}	0.022	-0.017	0.03	0.012
	(0.016)	(0.016)	(0.021)	(0.015)	(0.015)
	p = 0.017	p = 0.183	p = 0.404	p = 0.053	p = 0.438
fold7	1.006e-03	0.021	7.738e-03	-7.655e-04	0.015
	(0.016)	(0.016)	(0.021)	(0.015)	(0.015)
	p = 0.95	p = 0.193	p = 0.706	p = 0.96	p = 0.316
fold8	0.025	0.015	0.043^{*}	0.01	0.011
	(0.016)	(0.016)	(0.02)	(0.015)	(0.015)
	p = 0.122	p = 0.354	p = 0.035	p = 0.501	p = 0.471
fold9	0.04^{*}	0.039^{*}	0.058^{*}	0.023	0.034^{*}
	(0.017)	(0.017)	(0.021)	(0.016)	(0.015)
	p = 0.016	p = 0.019	p = 6.344e-03	p = 0.137	p = 0.027
Constant	0.438^{***}	0.403^{***}	0.331^{***}	0.505^{***}	-0.082
	(0.051)	(0.054)	(0.065)	(0.048)	(0.047)
	p = 1.107e-17	p = 6.987e-14	p = 3.247e-07	p = 3.037e-25	p = 0.085
Observations	5904	4292	5904	5904	5904
R^2	0.052	0.034	0.061	0.012	0.06
Adjusted R^2	0.05	0.03	0.059	9.687e-03	0.058

Note: *p<0.05; **p<1.000e-03; ***p<1e-06

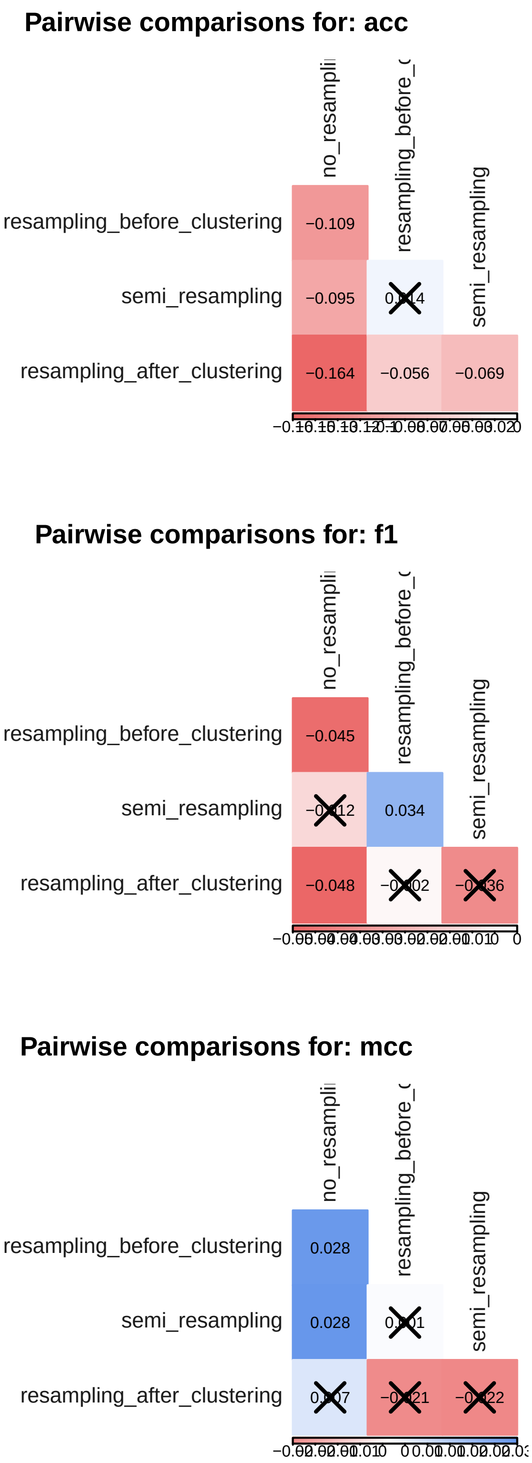

Figure 10: Pairwise comparison of strategy performance using Tukey method.

Table 14: Expected absolute performances, by metric and strategy, with 95% confidence intervals.

metric	strategy	emmean	SE	lower.CL	upper.CL
acc	no_resampling	0.720	7.179e-03	0.706	0.734
acc	resampling_before_clustering	0.611	7.476e-03	0.596	0.625
acc	semi_resampling	0.624	7.179e-03	0.610	0.638
acc	resampling_after_clustering	0.555	1.099e-02	0.534	0.577
auroc	no_resampling	0.634	7.122e-03	0.621	0.648
auroc	resampling_before_clustering	0.624	7.105e-03	0.610	0.637
auroc	semi_resampling	0.629	7.122e-03	0.615	0.643
auroc	resampling_after_clustering	0.585	9.500e-03	0.566	0.604
f1	no_resampling	0.566	9.125e-03	0.548	0.584
f1	resampling_before_clustering	0.520	9.503e-03	0.502	0.539
f1	semi_resampling	0.554	9.125e-03	0.536	0.572
f1	resampling_after_clustering	0.518	1.396e-02	0.491	0.546
balanced_acc	no_resampling	0.610	6.826e-03	0.597	0.623
balanced_acc	resampling_before_clustering	0.587	7.109e-03	0.573	0.601
balanced_acc	semi_resampling	0.584	6.826e-03	0.570	0.597
balanced_acc	resampling_after_clustering	0.556	1.045e-02	0.535	0.576
mcc	no_resampling	0.108	6.682e-03	0.095	0.121
mcc	resampling_before_clustering	0.136	6.959e-03	0.122	0.150
mcc	semi_resampling	0.137	6.682e-03	0.123	0.150
mcc	resampling_after_clustering	0.115	1.023e-02	0.095	0.135

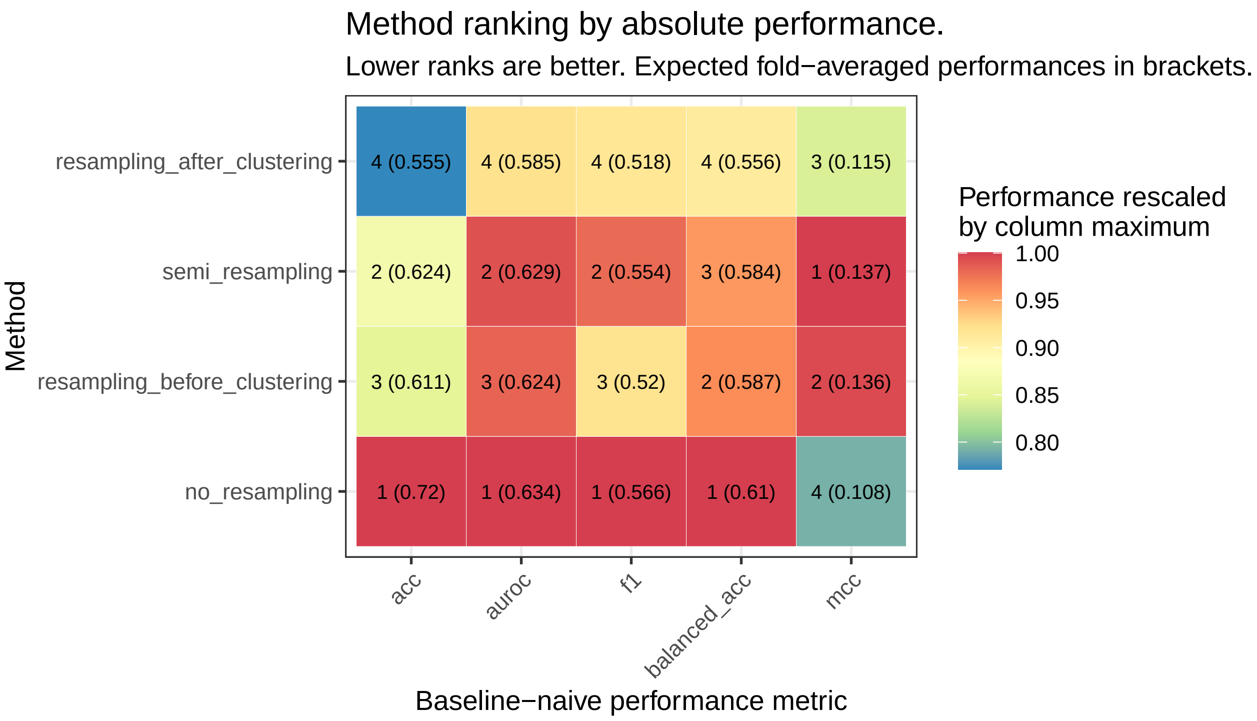

Figure 11: Method ranking according to the linear model predicted performances for each metric. Baseline metrics were ignored.

5.2 Baseline-adjusted performance

To address the pitfalls of the direct comparison of metrics whose baselines may differ, baseline-adjusted performance metrics were defined and modelled analogously. Specifically:

$$\text{adj\_metric} = \text{metric} - \text{baseline}$$

A descriptive plot of the the adjusted metrics (figure 12) pointed to a **scenario different than that of unadjusted ones** (figure 9).

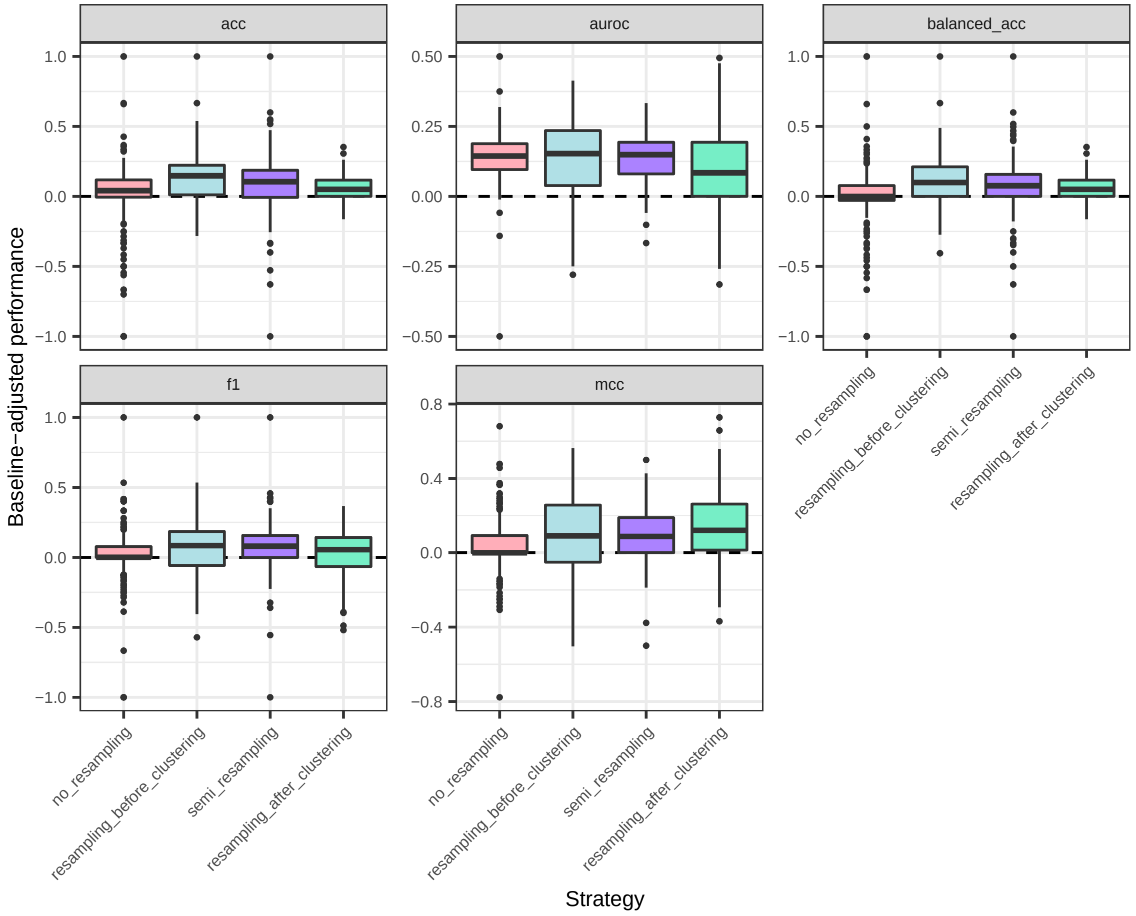

Figure 12: Baseline-adjusted performance metrics for balancing strategies. Data points correspond to proteins, averaged over folds.

Adjusted performance metrics were described with the following linear model:

$$\text{adj\_metric} \sim \text{strategy} + \log10(n_{int}) + \log10(n_{seq}) + k_{fold}$$

Note that while all metrics but `mcc` were non-negative, the adjusted metrics could show negative values when the performance of the DL model was lower than that of the baseline.

Again, `strategy` covariate was **always significant** in a type 3 ANOVA (table 15). **Baseline adjustment brought a uniform behaviour across the models, except for AUROC still suggesting no resampling as the best strategy in PRs** (table 16), further confirmed in pairwise coefficient comparison (Tukey's method, figure 13) and in their expected performance (table 17 and figure 14):

- **In PRs, resampling before clustering was the best performer, closely followed by semi resampling, then no resampling, and resampling after clustering**.
- **In PRs the general picture of resampling before clustering and semi resampling being the best was already present in unadjusted MCC, and is mostly kept in ajdusted metrics**.

Table 15: ANOVA p-values for including the resampling strategy as a regressor in the adjusted performance models.

strategy	variable	Sum Sq	Df	F value	Pr(>F)
acc	strategy	4.591602	3	1.82e+01	1.02e-11
auroc	strategy	1.116042	3	4.09e+00	6.55e-03
f1	strategy	2.165202	3	1.01e+01	1.30e-06
balanced_acc	strategy	4.523000	3	1.80e+01	1.30e-11
mcc	strategy	1.024287	3	2.82e+00	3.77e-02

Conclusions drawn from the baseline-adjusted performance analysis:

- **In PRs, resampling before clustering performed similarly to semi resampling, and better than no resampling and resampling after clustering. Therefore, augmenting the test set was not the largest performance driver**.
- `semi_resampling` outperformed `no_resampling` in **two out of five metrics, and non-significant in the remaining three** (Tukey's method, $p < 0.05$, figure 13), which **supports data augmentation usefulness** even if the data balance in the test set differs from that of the training set. This was **consistent with the observation that the predicted proportion of positives of the PCM model was mainly driven by the actual data balance in the test set, rather than that of the training set. Combined with the healthier distributions of predicted active ratios** of `semi_resampling` against `no_resampling`, this made a **case in favour of the former**.
- In **five** out of five metrics, **proteins with more interactions were better predicted** (table 16).

Table 16: Linear models to describe each adjusted performance metric. Standard deviations in parentheses.

	acc	auroc	f1	balanced_acc	mcc
	(1)	(2)	(3)	(4)	(5)
strategyresampling_before_clustering	0.056^{***}	$-6.020e\text{-}03$	0.027^{*}	0.069^{***}	0.033^{*}
	(0.01)	(0.012)	(9.460e-03)	(0.01)	(0.012)
	p = 3.898e-08	p = 0.624	p = 4.670e-03	p = 1.650e-11	p = 8.010e-03
strategysemi_resampling	0.027^{*}	−0.011	0.041^{**}	0.045^{**}	0.023
	(0.01)	(0.013)	(9.629e-03)	(0.01)	(0.013)
	p = 0.011	p = 0.376	p = 1.816e-05	p = 1.482e-05	p = 0.062
strategyresampling_after_clustering	-0.035^{*}	-0.051^{**}	−0.016	6.630e-03	4.841e-03
	(0.014)	(0.015)	(0.013)	(0.014)	(0.017)
	p = 0.014	p = 7.690e-04	p = 0.23	p = 0.639	p = 0.776
log10(n_interactions)	0.061^{***}	0.073^{***}	0.032^{***}	0.044^{***}	0.093^{***}
	(6.353e-03)	(8.505e-03)	(5.859e-03)	(6.335e-03)	(7.619e-03)
	p = 1.528e-21	p = 8.457e-18	p = 6.161e-08	p = 2.983e-12	p = 7.318e-34
log10(len_seq)	0.051^{*}	3.223e-03	-0.037^{*}	0.021	−0.025
	(0.02)	(0.024)	(0.018)	(0.02)	(0.024)
	p = 0.011	p = 0.894	p = 0.045	p = 0.281	p = 0.297
fold1	0.036^{*}	0.02	0.041^{*}	0.018	−2.052e-03
	(0.018)	(0.022)	(0.017)	(0.018)	(0.022)
	p = 0.045	p = 0.355	p = 0.013	p = 0.316	p = 0.924
fold2	−0.032	0.014	0.026	−0.025	0.015
	(0.018)	(0.02)	(0.016)	(0.017)	(0.021)
	p = 0.071	p = 0.506	p = 0.105	p = 0.145	p = 0.474
fold3	−0.015	0.042^{*}	0.093^{***}	−0.015	0.032
	(0.018)	(0.021)	(0.016)	(0.018)	(0.021)
	p = 0.381	p = 0.042	p = 1.039e-08	p = 0.382	p = 0.129
fold4	0.028	0.028	0.04^{*}	0.013	0.022
	(0.017)	(0.02)	(0.016)	(0.017)	(0.021)
	p = 0.104	p = 0.163	p = 0.014	p = 0.456	p = 0.285
fold5	0.033	0.046^{*}	0.012	0.023	0.043^{*}
	(0.018)	(0.021)	(0.017)	(0.018)	(0.022)
	p = 0.069	p = 0.031	p = 0.474	p = 0.192	p = 0.046
fold6	0.063^{**}	0.049^{*}	9.638e-03	0.061^{**}	0.043^{*}
	(0.018)	(0.021)	(0.016)	(0.018)	(0.021)
	p = 4.240e-04	p = 0.019	p = 0.556	p = 6.183e-04	p = 0.043
fold7	8.429e-04	0.031	0.015	−6.795e-04	0.018
	(0.018)	(0.021)	(0.016)	(0.018)	(0.021)
	p = 0.962	p = 0.143	p = 0.353	p = 0.969	p = 0.405
fold8	0.028	0.028	0.039^{*}	0.02	0.04
	(0.017)	(0.02)	(0.016)	(0.017)	(0.021)
	p = 0.111	p = 0.168	p = 0.017	p = 0.259	p = 0.057
fold9	0.031	0.044^{*}	0.041^{*}	0.014	0.037
	(0.018)	(0.021)	(0.017)	(0.018)	(0.022)
	p = 0.09	p = 0.039	p = 0.014	p = 0.453	p = 0.085
Constant	-0.235^{**}	−0.106	0.025	-0.144^{*}	−0.1
	(0.057)	(0.07)	(0.052)	(0.057)	(0.068)
	p = 3.387e-05	p = 0.127	p = 0.628	p = 0.011	p = 0.14
Observations	5355	4134	5355	5355	5355
R^2	0.038	0.021	0.021	0.028	0.035
Adjusted R^2	0.035	0.018	0.018	0.026	0.033

Note: *p<0.05; **p<1.000e-03; ***p<1e-06

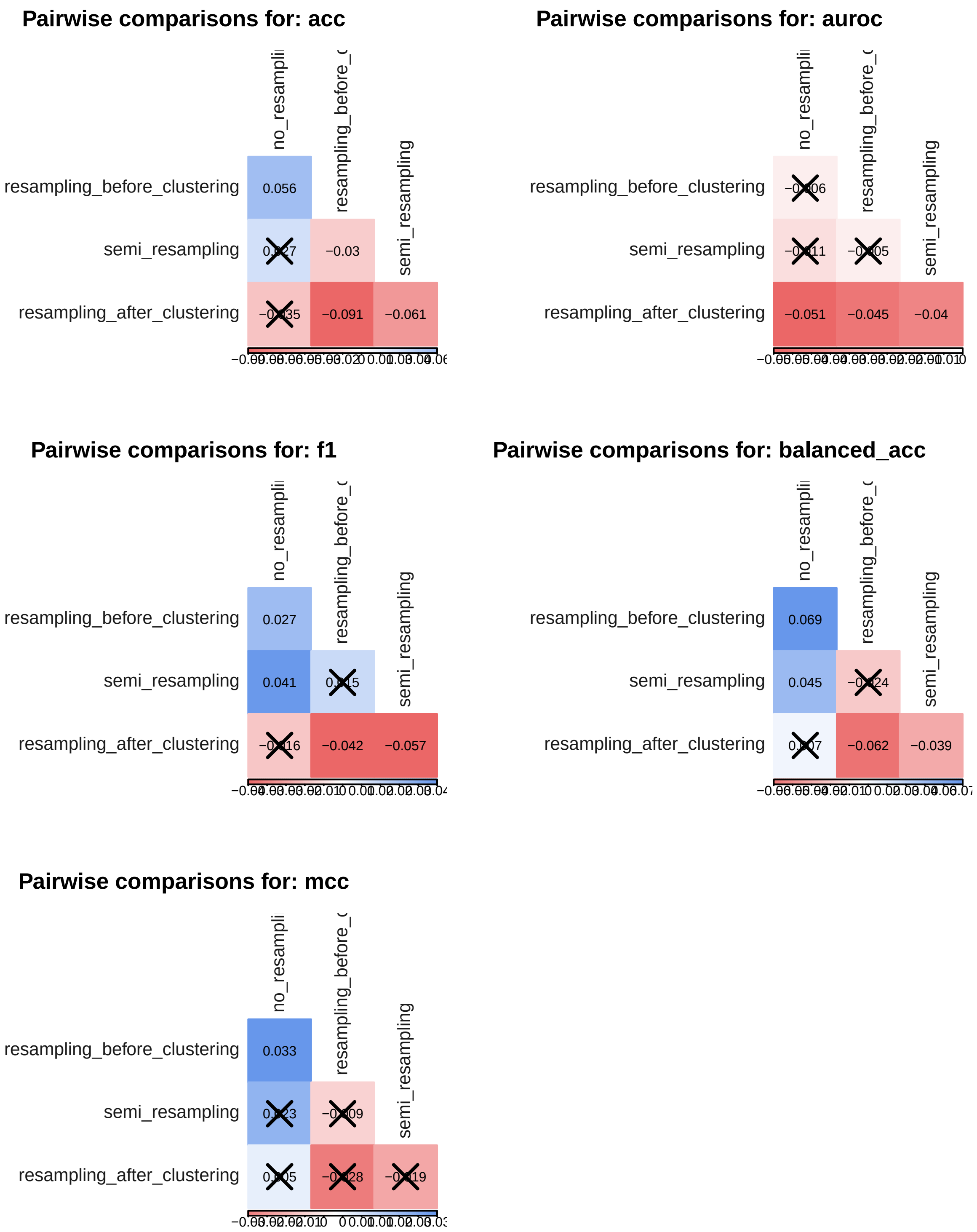

Figure 13: Pairwise comparison of strategy adjusted performance using Tukey method.

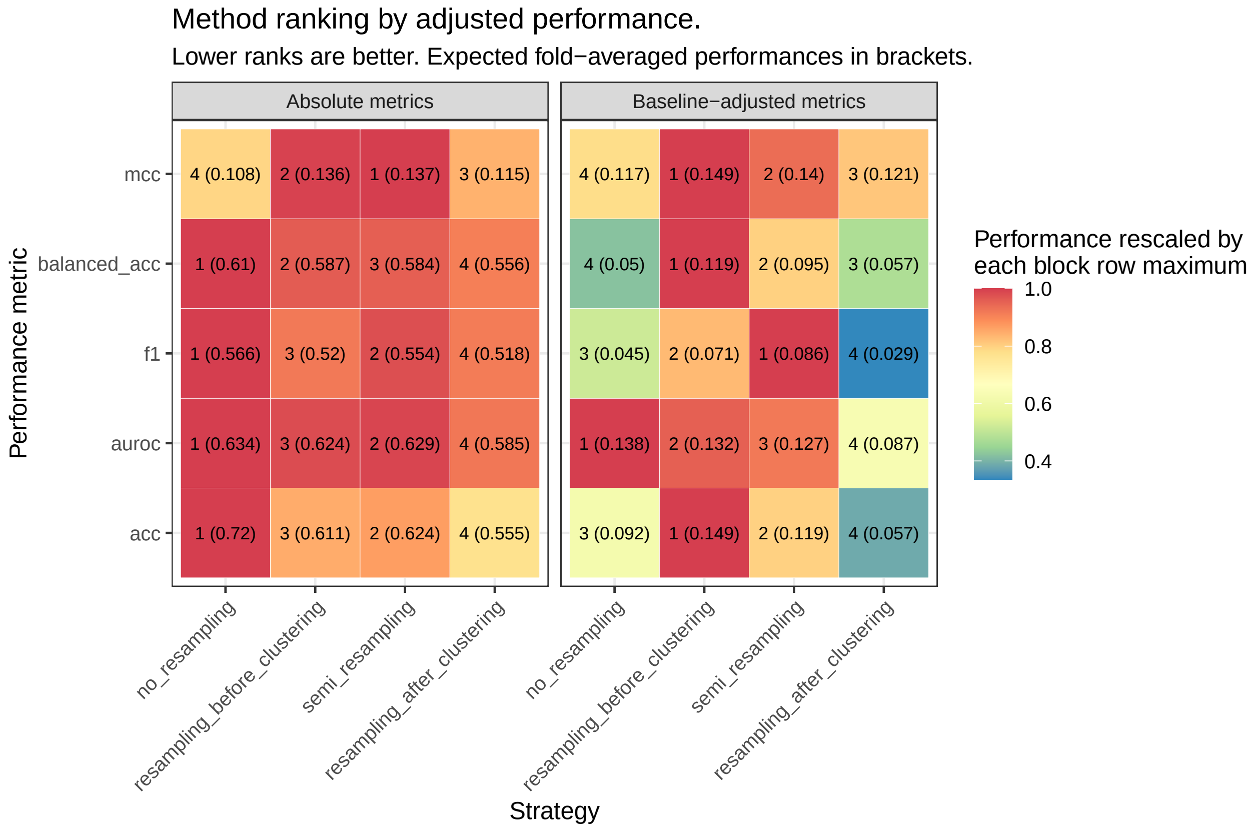

Figure 14: Method ranking according to the linear model predicted adjusted performances for each metric. Baseline metrics were taken into account in the adjustment. For a direct comparison, the same ranking using the absolute metrics was kept side by side.

Table 17: Expected adjusted performances, by metric and strategy, with 95% confidence intervals.

metric	strategy	emmean	SE	lower.CL	upper.CL
acc	no_resampling	0.092	7.925e-03	0.077	0.108
acc	resampling_before_clustering	0.149	8.018e-03	0.133	0.165
acc	semi_resampling	0.119	8.091e-03	0.103	0.135
acc	resampling_after_clustering	0.057	1.171e-02	0.035	0.080
auroc	no_resampling	0.138	9.147e-03	0.121	0.156
auroc	resampling_before_clustering	0.132	9.093e-03	0.115	0.150
auroc	semi_resampling	0.127	9.347e-03	0.109	0.146
auroc	resampling_after_clustering	0.087	1.218e-02	0.063	0.111
f1	no_resampling	0.045	7.308e-03	0.030	0.059
f1	resampling_before_clustering	0.071	7.394e-03	0.057	0.086
f1	semi_resampling	0.086	7.461e-03	0.071	0.100
f1	resampling_after_clustering	0.029	1.080e-02	0.008	0.050
balanced_acc	no_resampling	0.050	7.903e-03	0.035	0.066
balanced_acc	resampling_before_clustering	0.119	7.996e-03	0.103	0.135
balanced_acc	semi_resampling	0.095	8.068e-03	0.079	0.111
balanced_acc	resampling_after_clustering	0.057	1.168e-02	0.034	0.080
mcc	no_resampling	0.117	9.504e-03	0.098	0.135
mcc	resampling_before_clustering	0.149	9.616e-03	0.130	0.168
mcc	semi_resampling	0.140	9.703e-03	0.121	0.159
mcc	resampling_after_clustering	0.121	1.404e-02	0.094	0.149

6 Reproducibility

- R version 3.6.3 (2020-02-29), `x86_64-pc-linux-gnu`
- Locale: `LC_CTYPE=en_US.UTF-8`, `LC_NUMERIC=C`, `LC_TIME=es_ES.UTF-8`, `LC_COLLATE=en_US.UTF-8`, `LC_MONETARY=es_ES.UTF-8`, `LC_MESSAGES=en_US.UTF-8`, `LC_PAPER=es_ES.UTF-8`, `LC_NAME=C`, `LC_ADDRESS=C`, `LC_TELEPHONE=C`, `LC_MEASUREMENT=es_ES.UTF-8`, `LC_IDENTIFICATION=C`
- Running under: `Ubuntu 16.04.7 LTS`
- Matrix products: default
- BLAS: `/usr/lib/atlas-base/atlas/libblas.so.3.0`
- LAPACK: `/usr/lib/atlas-base/atlas/liblapack.so.3.0`
- Base packages: base, datasets, graphics, grDevices, methods, stats, utils
- Other packages: corrplot 0.84, dplyr 1.0.5, forcats 0.5.1, ggplot2 3.3.3, gsubfn 0.7, kableExtra 1.3.4, magrittr 2.0.1, proto 1.0.0, purrr 0.3.4, readr 1.4.0, rmarkdown 2.7, stargazer 5.2.2, stringr 1.4.0, tibble 3.1.0, tidyr 1.1.3, tidyverse 1.3.0
- Loaded via a namespace (and not attached): abind 1.4-5, assertthat 0.2.1, backports 1.2.1, bookdown 0.21, broom 0.7.5, car 3.0-10, carData 3.0-4, cellranger 1.1.0, cli 2.3.1, codetools 0.2-16, colorspace 2.0-0, compiler 3.6.3, crayon 1.4.1, curl 4.3, data.table 1.14.0, DBI 1.1.1, dbplyr 2.1.0, digest 0.6.27, ellipsis 0.3.1, emmeans 1.5.4, estimability 1.3, evaluate 0.14, fansi 0.4.2, farver 2.1.0, foreign 0.8-76, fs 1.5.0, generics 0.1.0, glue 1.4.2, grid 3.6.3, gtable 0.3.0, haven 2.3.1, highr 0.8, hms 1.0.0, htmltools 0.5.1.1, httr 1.4.2, jsonlite 1.7.2, knitr 1.31, labeling 0.4.2, lattice 0.20-41, lifecycle 1.0.0, lubridate 1.7.10, MASS 7.3-53, Matrix 1.2-18, mgcv 1.8-33, modelr 0.1.8, multcomp 1.4-16, munsell 0.5.0, mvtnorm 1.1-1, nlme 3.1-149, openxlsx 4.2.3, pillar 1.5.1,

pkgconfig 2.0.3, plyr 1.8.6, ps 1.6.0, R6 2.5.0, RColorBrewer 1.1-2, Rcpp 1.0.6, readxl 1.3.1, reprex 1.0.0, reshape2 1.4.4, rio 0.5.26, rlang 0.4.10, rstudioapi 0.13, rvest 0.3.6, sandwich 3.0-0, scales 1.1.1, splines 3.6.3, stringi 1.5.3, survival 3.2-7, svglite 2.0.0, systemfonts 1.0.1, tcltk 3.6.3, TH.data 1.0-10, tidyselect 1.1.0, tools 3.6.3, utf8 1.1.4, vctrs 0.3.6, viridisLite 0.3.0, webshot 0.5.2, withr 2.4.1, xfun 0.21, xml2 1.3.2, xtable 1.8-4, yaml 2.2.1, zip 2.1.1, zoo 1.8-8
